# Beech latewood density as a proxy for temperature reconstruction

**DOI:** 10.1126/sciadv.aee1305

**Published:** 2026-07-03

**Authors:** Louis Verschuren, Vladimir Matskovsky, Matthieu N. Boone, Pieter De Frenne, Tom De Mil, Kristof Haneca, Shashidhara Marathe, Charlotte Pearson, Christoph Rau, Ute Sass-Klaassen, Joris Van Acker, Kris Vandekerkhove, Kaz Wanelik, Julia Weidemüller, Valerie Trouet, Jan Van den Bulcke

**Affiliations:** ^1^ UGent-Woodlab, Department of Environment, Faculty of Bioscience Engineering, Ghent University, Coupure links 653, 9000 Ghent, Belgium.; ^2^ UGCT-Centre for X-ray Tomography, Ghent University, Proeftuinstraat 86/N12, 9000 Ghent, Belgium.; ^3^ Forest & Nature Lab, Department of Environment, Faculty of Bioscience Engineering, Ghent University, Geraardsbergsesteenweg 267, 9090 Melle, Belgium.; ^4^ Department of Physics and Astronomy–Radiation Physics, Faculty of Sciences, Ghent University, Proeftuinstraat 86, 9000 Ghent, Belgium.; ^5^ Forest is life, TERRA Teaching and Research Centre, Gembloux Agro-Bio Tech., University of Liège, Passage des Déportés 2, 5030 Gembloux, Belgium.; ^6^ Flanders Heritage Agency, Havenlaan 88 box 5, 1000 Brussels, Belgium.; ^7^ Diamond Light Source, Harwell Science and Innovation Campus, Didcot OX11 0DE, UK.; ^8^ Laboratory of Tree Ring Research, University of Arizona, 1215 E Lowell St., Tucson, AZ 85721, USA.; ^9^ Forest Ecology and Forest Management Group, Wageningen University, P.O. Box 47, 6700 AA Wageningen, Netherlands.; ^10^ Sustainable Forest Management, Van Hall Larenstein University of Applied Sciences, Larensteinselaan 26-A, 6882 CT Velp, Netherlands.; ^11^ Research Group on Forest Ecology and Management, Research Institute for Nature and Forest (INBO), Gaverstraat 4, 9500 Geraardsbergen, Belgium.; ^12^ Dendroarchaeological Laboratory, Bavarian State Office for the Preservation of Monuments and Sites, Thierhaupten 86672, Germany.

## Abstract

Maximum latewood density of conifers is the most widely used annual-resolution summer temperature proxy. Regions with few conifers, however, remain underrepresented in global paleoclimate records. Here, we use x-ray micro–computed tomography (micro-CT) to show that latewood density measurements of European beech (*Fagus sylvatica* L.) in a temperate lowland forest exhibit a strong summer (May to September) temperature signal (*r* = 0.73; 1833 to 2022 CE). Complementary wood anatomical analyses using deep learning segmentation reveal that both vessel and fiber anatomy are key drivers of latewood density variability and its temperature sensitivity. By integrating these anatomical responses, x-ray micro-CT–based latewood density measurements generate a robust and temporally stable summer temperature signal. Our results highlight the untapped potential of broad-leaved tree species for density-based climate reconstructions in temperate regions and open previously unidentified avenues for high-resolution paleoclimatology beyond the use of conifers.

## Teaser

Beech latewood density provides a robust summer temperature signal, expanding climate reconstructions beyond conifers.

## INTRODUCTION

**Fig. 1.**
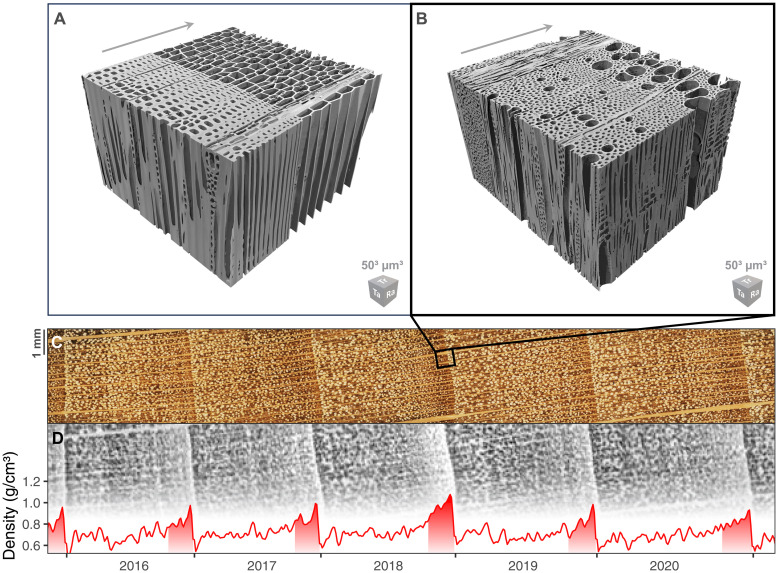
Wood anatomy of conifers versus broadleaves and latewood density of beech. High-resolution micro-CT scans of (**A**) Norway spruce (*P. abies*) and (**B**) European beech (*F. sylvatica*). These scans illustrate the simpler, more uniform wood anatomy of conifers (spruce) versus the complex, heterogeneous wood anatomy of broadleaves (beech). This complicates the interpretation of wood density profiles in tree-ring densitometry studies from broad-leaved species. The arrow shows the growth direction. Cubes (50^3^ μm^3^) show the orientation (transverse, tangential, and radial planes) and serve as a scale. This is also the voxel size of the beech increment core CT scans. These scans were made with a voxel size of 0.325^3^ μm^3^ using the I13-2 beamline at the Diamond Light Source synchrotron. See fig. S1 for additional species. (**C**) Optical scan from a section of a beech increment core used in this study. (**D**) Micro-CT scan (voxel size of 50^3^ μm^3^) from the same increment core. This shows a single 2D slice from the 3D stack. The grayscale represents the density (darker values being lower wood density), with the red line showing the density profile through the core. In this study, we define latewood density as the average density in the final 20% of the radial width of the ring (red shading). The 2018 ring exhibits a notably high latewood density, corresponding to the warmest summer on record in Belgium.

Proxy-based climate reconstructions are key to advancing our understanding of Earth’s climate history, contextualizing anthropogenic climate change, and guiding climate-related policies and actions (*1*). Tree-ring maximum latewood density (MXD) of conifers has emerged as the gold standard for high-resolution summer temperature reconstructions (*2*–*4*). This is because the thickness of tracheid cell walls formed at the end of the growing season is highly sensitive to summer temperature (*4*, *5*). The complex wood anatomy of broad-leaved species, however, complicates the interpretation of wood density variation across tree rings (Fig. 1 and fig. S1), which has limited their use in tree-ring densitometry studies. As a result, MXD temperature reconstructions are exclusively based on conifers, usually from high-latitude or high-elevation environments (*3*). This makes regions that have few conifers, often with a long history of human activity (*6*), underrepresented in the paleoclimatic temperature record. Anthropogenic climate change and climate extremes have high societal impacts in such (often temperate) regions, and so, developing broadleaf-based annual-resolution temperature proxies addresses the urgent requirement to fill these gaps.

**Fig. 2.**
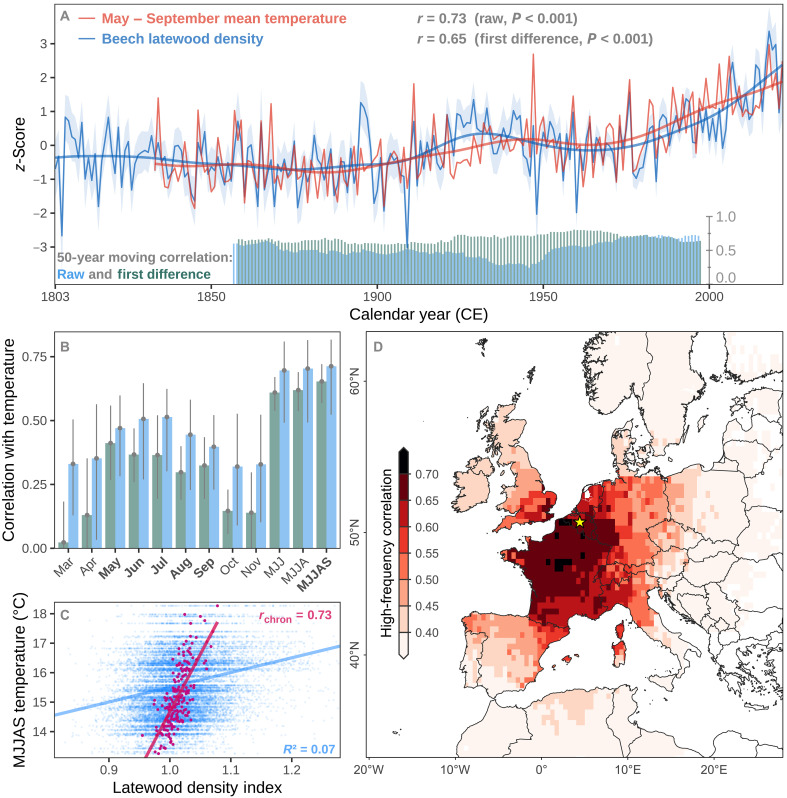
Beech latewood density correlation with temperature. (**A**) Latewood density chronology from the studied beech site near Brussels with 95% confidence interval (blue) plotted against instrumental May to September mean temperature data (red) from a synoptic weather station in Uccle (7 km from the study site) with 50-year smoothing splines showing the trends. The 50-year moving correlation between these two variables is plotted below; both raw (blue) and high-frequency (first difference, green) correlations are shown. *Z*-scores are calculated with 1833 to 2022 as the reference period. (**B**) Correlation between the latewood density chronology and instrumental temperature of individual months and month combinations (1833 to 2022). MJJAS (May, June, July, August, and September) is on the right. Both raw (blue) and high-frequency (first difference, green) correlations are shown with 95% percentile range. (**C**) Scatterplot comparing individual detrended micro-CT latewood density measurements with mean May to September temperatures. The fitted marginal regression with its marginal *R*^2^ is in blue. Chronology values (annual mean), with the corresponding linear regression and correlation coefficient, are shown in red. (**D**) High-frequency (first difference) spatial correlation between the latewood density chronology and 1901 to 2022 gridded May to September mean temperature (CRU TS 4.08). Only significant (*P* < 0.05) correlations are shown. The study site is indicated with a yellow star. See fig. S5 for spatial correlation of the raw chronology and climate data.

Here, we present a latewood density chronology (1803 to 2022 CE) of European beech (*Fagus sylvatica* L.), a widely distributed and often dominant tree species in midlatitude regions in Europe. Various wood density characteristics in beech tree rings are influenced by climate factors (*7*–*10*), yet the potential of using these wood density–based time series for paleoclimate reconstruction remains unexplored. We conducted three-dimensional (3D) measurements of wood density with x-ray micro–computed tomography (micro-CT) (*11*) on 242 cores of 121 beech trees from a single temperate lowland forest site near Brussels, Belgium (Fig. 2D). From the density profile across each tree ring, we tested different density variables. Among them, latewood density—defined as the average density in the final 20% of the tree ring (Fig. 1D and fig. S2), representing the wood formed at the end of each growing season—proved to be the most stable. This variable enables us to investigate climate correlations despite the complex wood anatomy of beech compared to conifers (*10*).

## RESULTS

To calculate the relation between the beech latewood density chronology and contemporary climate, we used one of the longest continuous instrumental records of temperature and precipitation in Europe (1833 to present), which is from the Uccle weather station located 7 km from the study site. We found a strong and temporally stable positive correlation (1833 to 2022 CE) with monthly temperatures from May to September, with the strongest correlations in June (*r* = 0.52, *P* < 0.01) and July (*r* = 0.52, *P* < 0.01) (Fig. 2B, fig. S3, and table S1). The wood density-temperature relationship was the strongest when considering the average temperature across the entire May to September window (*r* = 0.73, *P* < 0.001; Fig. 2), with a strong high-frequency temperature signal (first difference, *r* = 0.65, *P* < 0.001). Latewood density generated the strongest temperature signal, while tree-ring characteristics, such as ring width and other relevant tree-ring density parameters, showed only weak climate correlations (fig. S4). The May to September temperature signal in our latewood density chronology has a broad spatial coverage extending across much of Western Europe’s temperate climate regions (Fig. 2D and fig. S5). Spectral analysis shows good coherence between the chronology and the temperature target in the high (2- to 4- and 7-year periods) and low frequency (50+-year period) range (fig. S6).

The latewood density chronology exhibits an average pairwise correlation between series (rbar) of 0.14 and an overall interseries correlation (average correlation between the series and a master chronology as in COFECHA) of 0.33 (table S2). The large final sample size (119 trees) enables a sensitivity analysis of chronology statistics and climate correlation to a reduced sample size. The subsampling analysis (fig. S7) shows that an expressed population signal (EPS) of 0.85—commonly considered the minimum threshold in dendrochronology (*12*)—is reached with an average of 19 trees. Chronologies based on this sample size capture 65% of the May to September temperature signal represented in the full dataset. Increasing the sample size leads to a gradual strengthening of the climate signal: Subsets of 55 trees capture 90% of the full-dataset temperature signal, corresponding to fractions of explained variance of 0.48 and 0.53 for the reduced and full datasets, respectively.

**Fig. 3.**
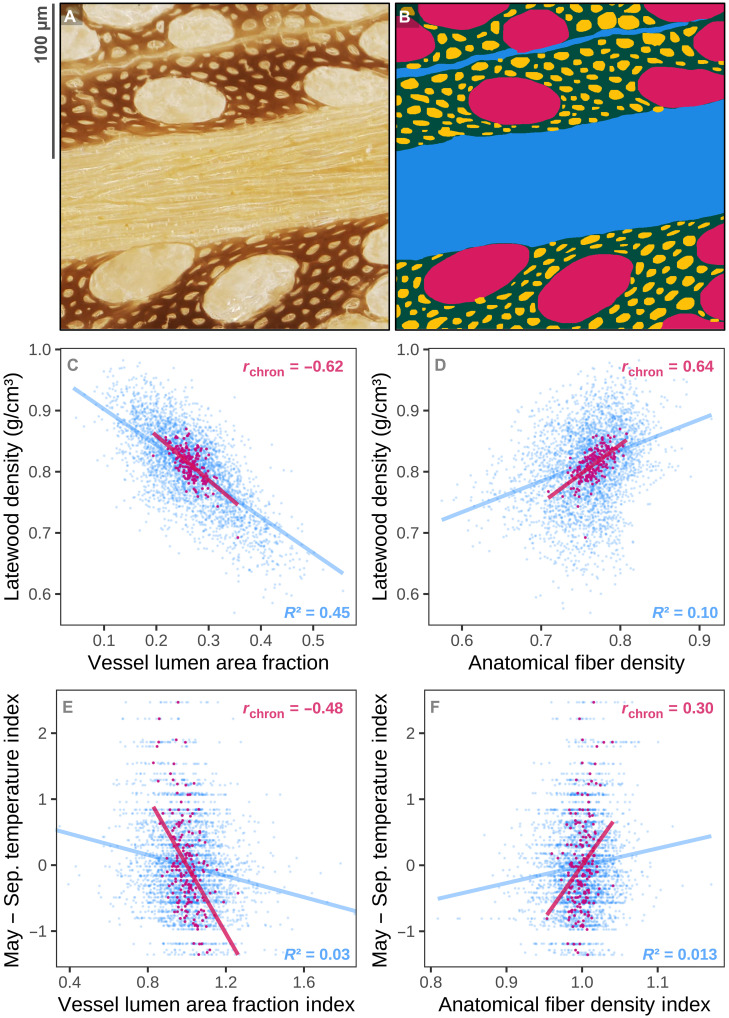
Anatomical explanation of latewood density and its temperature signal. (**A**) Section of an optically scanned increment core at a 0.2-μm pixel size. (**B**) Anatomical segmentation using a YOLOv8l deep learning model: vessel lumens (red), rays (blue), cell walls (green), and fiber lumens (yellow). (**C** and **D**) Scatterplots comparing micro-CT latewood density and two optically measured anatomical parameters: vessel lumen area fraction and anatomical fiber density (anatomical density of the cell walls and fiber lumen). Both are unitless. All calculations were corrected to exclude the ray area. The fitted marginal regression with its marginal *R*^2^ is in blue. Chronology values (annual mean), with the corresponding linear regression and correlation coefficient, are shown in red. (**E** and **F**) Scatterplots comparing detrended mean May to September temperatures with detrended anatomical parameters (both detrended with 30-year splines, unitless indices). An extended version of this plot is available as fig. S8.

**Fig. 4.**
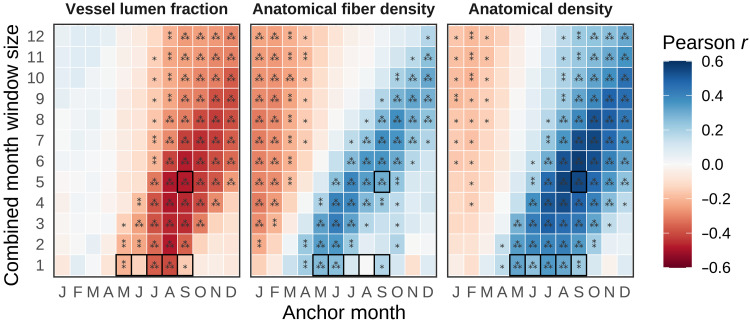
Correlations between latewood anatomy and temperature. Correlation between monthly and averaged month combination temperature data from the Uccle weather station (1834 to 2021) and three anatomical latewood chronologies: vessel lumen area fraction, anatomical fiber density (anatomical density of the cell walls and fiber lumen), and anatomical density (1 − porosity). The *y* axis represents different month combination sizes of averaged monthly temperature, the anchor month being the last month in the month combination [for instance, anchor month J(une) with a combined month window size of 6 thus means that temperatures were calculated over a period of January to June]. Single-month correlations with May to September temperature, as well as average May to September temperature, are highlighted, as these are the sensitive months used in Figs. 2 and 3. Significant correlations are indicated with one, two, or three asterisks, corresponding to *P* < 0.05, *P* < 0.01, and *P* < 0.001, respectively. All tree-ring and temperature data for this figure were detrended with a 30-year smoothing spline. An extended version of this plot is available as fig. S9.

To deepen our understanding of possible tree-functional aspects behind the association between the beech latewood density and the temperature signal, the wood anatomical composition was investigated in more detail using high-resolution optical scans (Fig. 3A) and deep learning segmentation (Fig. 3B) (*13*) on 41 increment cores. Two key wood anatomical features proved to be good predictors of micro-CT latewood density and May to September temperature variation: vessel lumen area fraction (*r* = −0.62 and *r* = −0.48, respectively; *P* < 0.001) and the anatomical density of the fiber tissue (*r* = 0.64 and *r* = 0.3, respectively; *P* < 0.001) (Fig. 3 and fig. S8). Traits of vessels in the latewood of beech are known to respond to climate conditions of the current year (*14*), whereas the sensitivity of anatomical fiber density to temperature mirrors the response of latewood anatomical tracheid density in many conifers (*4*, *5*). We found that the vessel lumen area fraction in the latewood is mainly related to temperature in July and August, whereas the latewood anatomical fiber density is more sensitive to May, June, and September temperature variability (Fig. 4 and fig. S9). Temperature sensitivity across the entire May to September period is achieved by combining vessel- and fiber-related anatomical traits within micro-CT density. This joint expression of wood anatomical effects in micro-CT latewood density results in a robust and physiologically grounded proxy for summer temperature, facilitating its use in paleoclimate reconstructions.

## DISCUSSION

The strong and robust temperature signal in our beech latewood density chronology supports expansion of latewood density measurements across the dense and well-established European beech tree-ring network (*15*). A network of temperature-sensitive beech latewood density chronologies will extend and improve the spatial coverage of paleoclimate datasets in regions with short instrumental records. Moreover, the broad spatial extent of the temperature signal in our chronology (Fig. 2D) suggests that millennium-length growing season temperature reconstructions can be developed for Western and Central Europe on the basis of beech latewood density measurements of archeological wood. This has a reconstruction potential of 2000+ years (*16*–*19*). Developing annual-resolution temperature reconstructions in regions with a long history of human activity and written documentation will allow for a detailed investigation of past human-climate interactions (*20*, *21*). Beyond *F. sylvatica*, other *Fagus* species outside Europe (e.g., *Fagus grandifolia* in North America or *Fagus crenata* and *Fagus engleriana* in East Asia) may also prove suitable for latewood density–based temperature reconstructions, extending the potential reconstruction range into much of the lower latitudes of the Northern Hemisphere.

The average pairwise correlation between series (rbar) of 0.14 and overall interseries correlation of 0.33 for beech latewood density is considerably lower than values typically reported for conifer latewood density chronologies [0.25 to 0.6 (*22*–*24*)]. This difference likely reflects the more complex wood anatomy of broad-leaved species, as well as the comparatively less climatically extreme growing conditions at the study site relative to classical climate reconstruction settings. Low interseries correlation values, however, are a common feature of quantitative wood anatomy studies (*25*–*28*), many of which nevertheless exhibit strong and coherent climate signals. The sensitivity analysis of chronology statistics and climate correlations shows that while a relatively modest number of trees is sufficient to meet commonly applied chronology quality thresholds, substantially larger sample sizes are required to fully capture the strength of the climate signal present in the complete latewood density dataset. Together, these results underscore that low interseries correlation values do not necessarily preclude robust climate sensitivity, but they do imply a greater reliance on large sample sizes to reliably extract the shared climate signal.

In summary, we demonstrate that latewood density in a broad-leaved species can serve as a reliable proxy for growing season and summer temperature, with the potential to boost existing climate reconstructions to temperate regions and refine information on local changes in temperature. Our results provide a clear demonstration of how density-based temperature reconstruction can be successfully derived from nonconifer species in lowland regions. This opens up exciting new potential and capacity to fill key regional data gaps for informing studies of past human/climate interactions.

## MATERIALS AND METHODS

### Study area and fieldwork

This study was conducted in the Joseph Zwaenepoel Forest Reserve (50.75°N, 4.42°E), located within the Sonian forest in Belgium (Fig. 2D). This reserve exemplifies a lowland old-growth beech forest containing many overmature beech trees up to 250 years old and holds significance as part of the UNESCO World Heritage Site “Ancient and Primeval Beech Forests of the Carpathians and Other Regions of Europe” (*29*). The site was selected because it combines environmental conditions traditionally considered unfavorable for strong tree-ring climate signals with good instrumental data. It lies ~7 km from the Uccle synoptic weather station, which provides one of the longest continuous instrumental temperature records in Europe (since 1833 CE), enabling a robust assessment of proxy-climate relationships and their temporal stability. In addition, the high abundance of mature beech trees exceeding 200 years in age allows the construction of long and well-replicated chronologies.

The sampled area is on a relatively flat plateau at a mean elevation of 113 m above sea level. The soil is a dry loamy luvisol consisting of Tertiary calcium-rich sandstone substrate, covered with a 0.5- to 4-m-thick layer of Quaternary niveo-aeolic loess deposits of the Weichselian glaciation (*30*). The climate is temperate and characterized by a total annual rainfall of 849 mm and a mean annual temperature of 10.7°C (1991 to 2020, as measured in the Uccle weather station at a 7-km distance from the study site) (*31*). In total, 121 trees with a diameter at breast height above 25 cm were randomly selected and sampled in October 2021 and May 2023 for a dendroecological study (*32*). For each tree, two perpendicular cores were collected with a two-threaded 5.15-mm Haglöf increment borer. The mean diameter at breast height of the sampled trees is 68.8 cm (SD = 32.4), and the estimated ages range between 63 and 246 years. About half of the trees were planted around 1774, while the other half were planted or naturally regenerated between 1914 and 1959 (fig. S10C).

### Micro-CT and tree-ring density measurements

The 242 increment cores were soaked for 20 hours in a heated 80°C water bath and subsequently extracted with Soxhlet extraction for 6 hours using a 0.43:1 toluene:ethanol mixture (*33*). The samples were then dried, stored in paper straws, and conditioned in a climate chamber at 65% relative humidity and 20°C. We used x-ray micro-CT to measure 3D density variation within each core. Micro-CT is a novel, nondestructive, 3D densitometry technique enabling the simultaneous study of tree-ring width and wood density while requiring little sample preparation (*11*, *34*). The cores were scanned using the HECTOR (*35*) and TESCAN CoreTOM micro-CT scanners at UGCT (Centre for X-ray Computed Tomography of Ghent University) at a voxel size of 50^3^ μm^3^ (scan settings: circular cone beam scans; 100 kV; 10 W; 0.5-mm aluminum filter; 2201 projections per turn; 1000 ms per projection; source-to-detector distance, 1166 mm; source-to-object distance, 295.8 mm). Reconstruction was performed using Octopus Reconstruction (*36*, *37*). All tree-ring boundaries were indicated and visually cross-dated using the XCT toolchain (software packages available at www.dendrochronomics.ugent.be) (*11*, *34*, *38*–*43*). An additional check of the cross-dating quality was performed with COFECHA software (see data S1 to S3) (*44*) and in R (*45*) using the dplR package (*46*). Six of the 242 cores were removed from the dataset because of cross-dating issues, and from a further seven, the density data were removed because of excessive rot. Increment core density profiles were calculated using RingIndicator software (*43*) with the highest 50% value filter per 50-μm slice to more accurately handle the density of latewood near the (often wavy) tree-ring borders.

### Quantitative wood anatomy

We selected 41 increment cores, free from rot and defects, and sanded them to a 4000-grit finish using a robotic sander (*13*). A 0.5-mm-wide strip of each core was then optically scanned at a resolution of 0.2 μm using a Hirox HR-5000E digital microscope. Three hundred images of 640 by 640 pixels were randomly cropped from the images of the scanned cores to be annotated as training data for a deep learning segmentation model. These images were annotated in Roboflow (*47*) to identify fiber lumens, vessel lumens, and rays. The annotated dataset was divided into 240 images for training and 60 images for validation. A YOLOv8l deep learning segmentation model (*48*) was then trained for 300 epochs. Detailed training and performance metrics of the segmentation model are provided in figs. S11 to S13. Subsequently, the trained model was applied to the full-sized images of each core using a moving window approach (*13*), producing binary masks for vessel lumens, fiber lumens, rays, and cell walls.

### Density chronology development

We here defined beech latewood density as the average density in the final 20% of the radial width of each ring. According to our experiments, this portion of the tree-ring captures the strongest temperature signal and outperforms the maximum density (MXD), which aligns with previous findings for beech (*10*). This value was calculated from density profiles using the XCT.read function (*42*) in R, applying the highest 90% density value filter to exclude accidental inclusion of earlywood from the following growth ring. The latewood density series were detrended with an age-dependent smoothing spline with an initial stiffness of 50 years (*49*) using the detrend function from dplR version 1.7.8 (*46*). [Everywhere we mention *N*-year splines, we mean smoothing cubic splines with a 50% frequency response at the *N*-year wavelength (*50*).] This dataset is not well suited to more complex detrending approaches like regional curve standardization that are generally better at retaining low-frequency signals (*51*). The main reasons are that we only have two separate age classes with none in between (fig. S10) and that this dataset lacks any historical samples. All rings displaying rot or important defects, like small ingrown branches, were removed from the dataset. Rings narrower than 1 mm were excluded from the density dataset because of the anatomical challenges in defining the latewood of narrow rings in diffuse-porous broad-leaved species. This threshold also ensures that the latewood of each ring is adequately represented by a minimum of four micro-CT slices, each 50 μm in thickness. Then, Tukey’s biweight robust mean (*52*, *53*) was used for the averaging of the obtained dimensionless indices into the final latewood density chronology. Stationary and 50-year running average pairwise correlation between series (rbar), overall interseries correlation (average correlation between the series and a master chronology as in COFECHA), EPS, and subsample signal strength (*54*) were calculated using dplR (*46*). The chronology was cut off in 1803, as the subsample signal strength drops below 0.85 thereafter (*12*). In addition to latewood density, we developed chronologies for ring width, MXD (defined as the highest value in the density profile for that ring), and mean density in the remaining four 20% wide sectors of the ring using the same methodology as applied to the latewood density chronology.

### Anatomical chronology development

The XCT toolchain software (*42*, *43*) was used to calculate area percentages of vessel lumens, fiber lumens, rays, and cell walls in the final 20% of the radial width of each ring. Area fractions for vessel lumens, fiber lumens, and cell walls were adjusted by excluding the regions occupied by rays. From these, we calculated four anatomical parameters that were used in our analysis: anatomical density (1 − porosity), vessel lumen area fraction, fiber lumen area fraction, and anatomical fiber density. The anatomical fiber density was determined by calculating the ratio of cell wall area to the total fiber area: the sum of cell wall and fiber lumen areas, similar to anatomical density calculations in conifers (*5*). In addition, we calculated the specific hydraulic conductivity (*K*_s_) of the vessels in this latewood area. Theoretical hydraulic conductance [*K*_H_ in m^4^/(MPa·s)] was determined according to the Hagen-Poiseuille law using individual vessel diameters calculated in Python (*55*–*57*). The *K*_H_ of the latewood was then divided by the area of the latewood in that ring to obtain the specific theoretical hydraulic conductivity [*K*_s_ in m^2^/(s·MPa)], which is independent of ring size. All individual time series were then detrended using a 30-year smoothing spline. Again, only rings wider than 1 mm were retained. All series were then averaged to yearly chronologies using Tukey’s biweight robust mean.

### Climate data

Monthly instrumental temperature and precipitation data since 1833 are available from a synoptic weather station in Uccle, located 7 km from the study area. In addition, we used the CRU TS 4.08 temperature dataset, obtained through the KNMI Climate Explorer (*58*), for field correlations.

### Climate correlation

All density chronologies were correlated (Pearson correlation coefficient) with monthly temperature and precipitation data from Uccle over the 1833 to 2022 instrumental period, as well as with a 50-year sliding window using the dcc function from the treeclim R package (*59*, *60*). Similarly, correlations were calculated with the first difference of both chronology and climate data. We used this transformation to separate high-frequency (year-to-year) common variability independently from the long-term trends. The detrended anatomical chronologies were fitted to 30-year spline detrended May to September Uccle temperature data from 1833 to 2021 (*61*).

To assess the skill of the latewood density temperature reconstruction, we performed split calibration-validation tests with May to September mean temperature using various calibration periods (table S1). We used a linear regression as a transfer function for our reconstruction. We calculated correlation in the calibration (*R*_c_), correlation in the validation (*R*_v_) periods, reduction of error, and coefficient of efficiency of the model (*62*) using the skills function from the treeclim R package (*59*, *60*). Then, we used the full instrumental period (1833 to 2022 CE) for the final calibration of the model.

Using the KNMI Climate Explorer (*58*), a field correlation map was generated between our latewood density chronology and the CRU TS 4.08 temperature dataset from 1901 to 2022 (the overlap of the temperature and the tree-ring datasets). We did this for both raw and first difference–transformed chronology and temperature data.

### Spectral analysis and coherence

Spectral characteristics of the latewood density chronology and mean May to September temperature series were analyzed using Fourier analysis with the REDFIT (*63*) implementation in dplR. Both series were restricted to their common period (1833 to 2022 CE). Red-noise background spectra were parameterized, and statistical significance was assessed using 1000 Monte Carlo simulations. Spectral coherence between the latewood density chronology and the May to September temperature was assessed using multitaper analysis (*64*) using the multitaper R package (*65*, *66*).

### Micro-CT versus anatomy

For the 41 cores used in the anatomical analysis, a 1.5-mm-wide section of latewood, avoiding large rays, was indicated on each ring in the corresponding micro-CT scans. This removes the effect of large rays on the density profiles. No minimum density filter was used for these density profiles. Again, only rings wider than 1 mm were retained. The resulting dataset consists of 3319 rings with coupled micro-CT latewood density and anatomical parameter measurements. Raw and 30-year spline detrended anatomical measurements were fitted to raw and 30-year spline detrended micro-CT latewood density measurements using linear mixed-effects models with the lmer function from the lme4 R package (*67*) with core ID included as a random effect. To evaluate model performance, marginal *R*^2^ values were calculated using the r.squaredGLMM function from the MuMIn R package (*68*). The same analysis was also performed between the detrended anatomical data and the 30-year smoothing spline detrended average May to September temperature data.

### Latewood definition

The profile measurements of micro-CT density, anatomical density, anatomical fiber density, fiber lumen fraction, and vessel lumen fraction from the 41 anatomically analyzed cores were used to create a comprehensive set of 125 chronologies. These varied in latewood definitions (e.g., final 10, 20, 30, 40, and 50% of the radial width of each ring) and applied different minimum ring-width filters (0, 0.25, 0.5, 0.75, and 1 mm). All series were detrended using a 30-year smoothing spline. The resulting chronologies were correlated with monthly Uccle temperature data (1834 to 2021) using varying month combination lengths (1 to 12 months) in a moving window approach. Temperature series were likewise detrended. This analysis allowed us to systematically explore how different latewood definitions influence the climate signal and to pinpoint which anatomical features are most sensitive to temperature during specific periods of the year.

### Sample-size sensitivity analysis

To quantify the robustness of chronology statistics and climate correlations to reduced sample size, we conducted a subsampling-based sensitivity analysis on the detrended latewood density dataset. Starting from the full pool of sampled trees, we repeatedly generated subsets containing increasing numbers of trees (5 to 100 trees). For each subset size, trees were randomly selected without replacement. Within each subsample, detrended latewood density series were averaged into a site chronology using Tukey’s biweight robust mean, following the same procedure as for the full dataset. rbar and EPS were calculated using the dplR package. Climate sensitivity was evaluated by correlating the resulting chronology with mean May to September instrumental temperature from Uccle using both raw and first difference–transformed series. For each sample size, the subsampling procedure was repeated 1000 times to generate a bootstrap distribution of each statistic. From these distributions, the mean and the central 95% bootstrap percentile interval were calculated.

### Synchrotron micro-CT

To illustrate the contrasting wood anatomy of conifers and broadleaves, we performed high-resolution micro-CT scans of Norway spruce (*Picea abies*), Scots pine (*Pinus sylvestris*), European beech (*F. sylvatica*), and pedunculate oak (*Quercus robur*). Scanning was conducted at the I13-2 beamline (*69*) of the Diamond Light Source synchrotron, enabling a higher spatial resolution than what is achievable with laboratory-based micro-CT systems. A pink beam centered around 27 keV was used, and samples were mounted on the Aerotech slip ring rotation stage with a hexapod base. All scans used a pco.edge camera paired with a 10× objective, resulting in a voxel size of 0.325^3^ μm^3^. A Paganin phase retrieval filter (*70*) was applied before reconstruction. Image reconstruction was carried out using Octopus Reconstruction (*36*, *37*), followed by denoising with a bilateral filter in Octopus Analysis to preserve edge sharpness. The final visualizations were generated using VGStudio MAX.
