# Supplementary material for: Beech latewood density as a proxy for temperature reconstruction
Source: Sci Adv. 2026 Jul 3;12(27):eaee1305. doi: 10.1126/sciadv.aee1305 (PMC13330825; doi:10.1126/sciadv.aee1305)
Supplement: Supplementary file 1 — Tables S1 and S2 Figs. S1 to S13 Data S1 to S3 [file sciadv.aee1305_sm.pdf]

Science Advances
AAAS

Supplementary Materials for

Beech latewood density as a proxy for temperature reconstruction

Louis Verschuren *et al.*

Corresponding author: Louis Verschuren, louis.verschuren@ugent.be

Sci. Adv. **12**, eaee1305 (2026)
DOI: 10.1126/sciadv.aee1305

This PDF file includes:

Tables S1 and S2
Figs. S1 to S13
Data S1 to S3

Table S1: **Split calibration test.** Reconstruction statistics of the mean May–September temperature using the beech latewood density chronology. r_c: correlation in the calibration period, r_v: correlation in the validation period, RE: Reduction of Error, CE: Coefficient of Efficiency.

Calibration	Validation	r_c	r_v	RE	CE
1833–1928	1929–2022	0.52	0.68	0.73	0.30
1929–2022	1833–1928	0.68	0.52	0.70	-0.05
1833–1972	1973–2022	0.54	0.72	0.80	0.30
1883–2022	1833–1882	0.70	0.59	0.68	0.30
1883–1972	1833–1882 and 1973–2022	0.48	0.82	0.68	0.64
1833–2022		0.73			

Table S2: **Overview of the developed tree-ring chronologies.** Chronology statistics for the main latewood density chronology (D 80 - 100) and all other chronologies used in fig. S4 and fig. S8. The shown statistics are the first and last year of the chronology, the number of increment cores and rings these chronologies were constructed from, and the average pairwise correlation between series (rbar), overall interseries correlation (average correlation between the series and a master chronology as in COFECHA), expressed population signal (EPS), and signal-to-noise ratio (SNR). The first seven chronologies were created using the full original micro-CT dataset, here all the individual tree-ring series were detrended using a 50-year age-dependent spline with an initial stiffness of 50 years before calculating the chronologies. The last 13 chronologies were constructed using the smaller dataset for anatomical analysis. The last seven chronologies were detrended using a 30-year spline before calculating the chronologies.

Chronology	Detrending	From (CE)	To (CE)	Samples	Rings	rbar	Overall interseries correlation	EPS	SNR
RWI	50-year age-dep. spline	1803	2022	236	29355	0.14	0.41	0.98	39.08
D 0-20	50-year age-dep. spline	1803	2022	229	20627	0.09	0.27	0.95	17.58
D 20-40	50-year age-dep. spline	1803	2022	229	20627	0.10	0.31	0.96	25.03
D 40-60	50-year age-dep. spline	1803	2022	229	20627	0.10	0.31	0.96	25.70
D 60-80	50-year age-dep. spline	1803	2022	229	20627	0.11	0.32	0.96	26.84
**D 80-100 (latewood density)**	50-year age-dep. spline	1803	2022	229	20627	0.14	0.33	0.97	35.96
MXD	50-year age-dep. spline	1803	2022	229	20627	0.13	0.30	0.97	30.56
micro-CT latewood density	None	1833	2021	41	3319	0.17	0.34	0.89	8.01
Anatomical density	None	1833	2021	41	3319	0.14	0.31	0.86	6.07
Vessel lumen area fraction	None	1833	2021	41	3319	0.14	0.24	0.87	6.51
Ks	None	1833	2021	41	3319	0.14	0.24	0.86	6.08
Fiber lumen area fraction	None	1833	2021	41	3319	0.19	0.33	0.90	8.98
Anatomical fiber density	None	1833	2021	41	3319	0.18	0.38	0.90	8.61
micro-CT latewood density	30-year spline	1833	2021	41	3319	0.12	0.34	0.84	5.22
Anatomical density	30-year spline	1833	2021	41	3319	0.11	0.29	0.83	4.77
Vessel lumen area fraction	30-year spline	1833	2021	41	3319	0.10	0.25	0.81	4.36
Ks	30-year spline	1833	2021	41	3319	0.10	0.24	0.81	4.17
Fiber lumen area fraction	30-year spline	1833	2021	41	3319	0.15	0.31	0.88	6.98
Anatomical fiber density	30-year spline	1833	2021	41	3319	0.15	0.34	0.88	7.06
RWI (limited samples)	30-year spline	1833	2021	41	5221	0.21	0.32	0.92	11.18

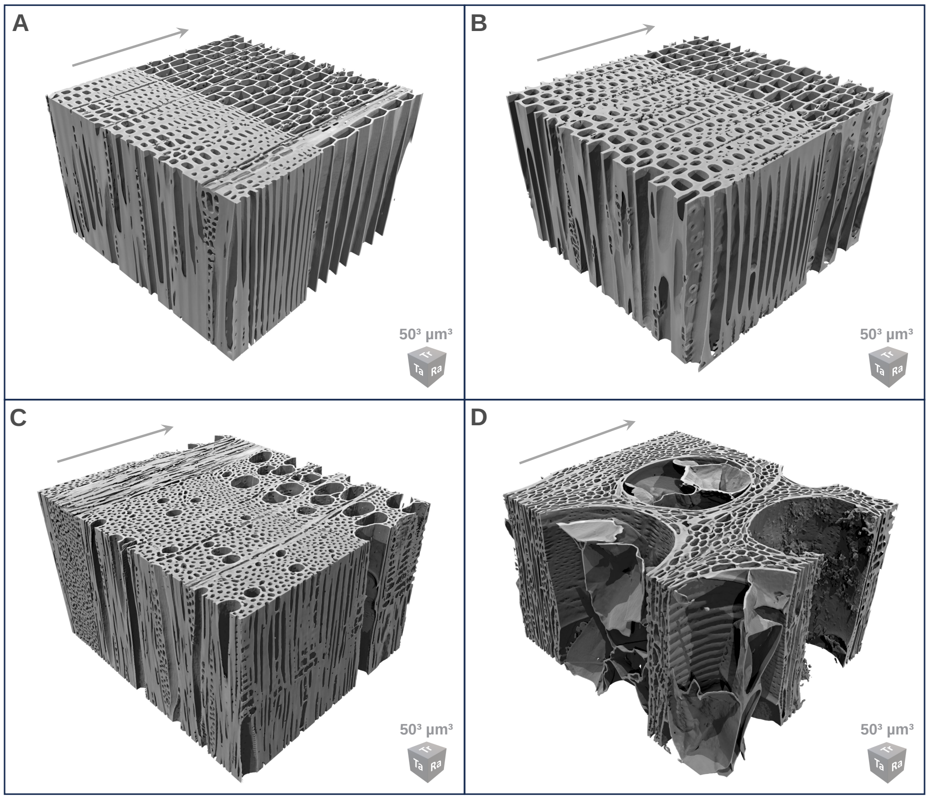

Figure S1: **Wood anatomy of conifers versus broadleaves (extended).** High-resolution micro-CT scans of (**A**) Norway spruce (*Picea abies*, same as Fig. 1A), (**B**) Scots pine (*Pinus sylvestris*), (**C**) European beech (*Fagus sylvatica*, same as Fig. 1B), and (**D**) pedunculate oak (*Quercus robur*). These scans illustrate the simpler, more uniform wood anatomy of conifers (spruce and pine) versus the complex, heterogeneous wood anatomy of broadleaves (beech and oak). This complicates the interpretation of wood density profiles in tree-ring densitometry studies from broad-leaved species. The arrow shows the growth direction. 50^3 μm^3 cubes show the orientation (transverse, tangential, and radial plane) and serve as a scale. This is also the voxel size of the beech increment core CT-scans. These scans were made with a voxel size of 0.325^3 μm^3 using the I13-2 beamline at the *Diamond Light Source* synchrotron.

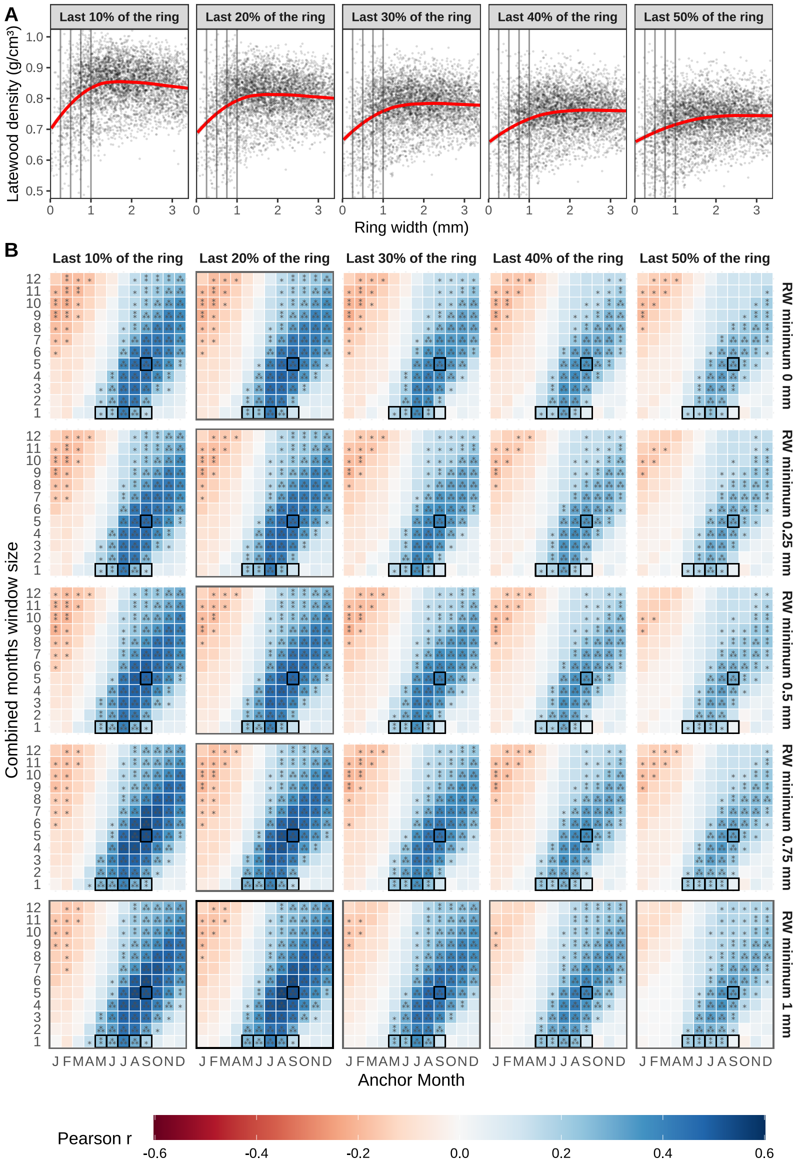

Figure S2: **Characterization and optimization of different beech latewood definitions.** (**A**) Relationship between ring width and latewood density, using five different definitions of latewood. Each panel shows the average density calculated over the last 10%, 20%, 30%, 40%, or 50% of the ring width. Individual points represent raw data from individual rings, and the red line indicates a smoothed (loess) fit to the data. The vertical lines represent the different minimum ring width filters: 0, 0.25, 0.5, 0.75, and 1 mm. (**B**) Correlation between monthly and averaged month combination temperature data from the Uccle weather station (1834–2021) and micro-CT latewood density chronologies with varying latewood area sizes and minimum ring width filters. The y-axis represents different averaged month combination sizes of averaged monthly temperature, the anchor month being the last month in the month combination. The columns represent differently sized sections of the ring as a definition for the latewood, with the 20% wide latewood results highlighted, as this is the definition of latewood used for the main analysis in this paper. The rows represent different minimum ring width filters, with the minimum of 1 mm highlighted, as this is the filter used for the main analysis in this paper. Single-month correlations with May–September temperature, as well as average May–September temperature, are also highlighted, as these are the most sensitive months used in this paper. All density and temperature data for this figure were detrended with a 30-year spline. Significant correlations are indicated with one, two, or three asterisks, corresponding to $p < 0.05$, $p < 0.01$, and $p < 0.001$, respectively.

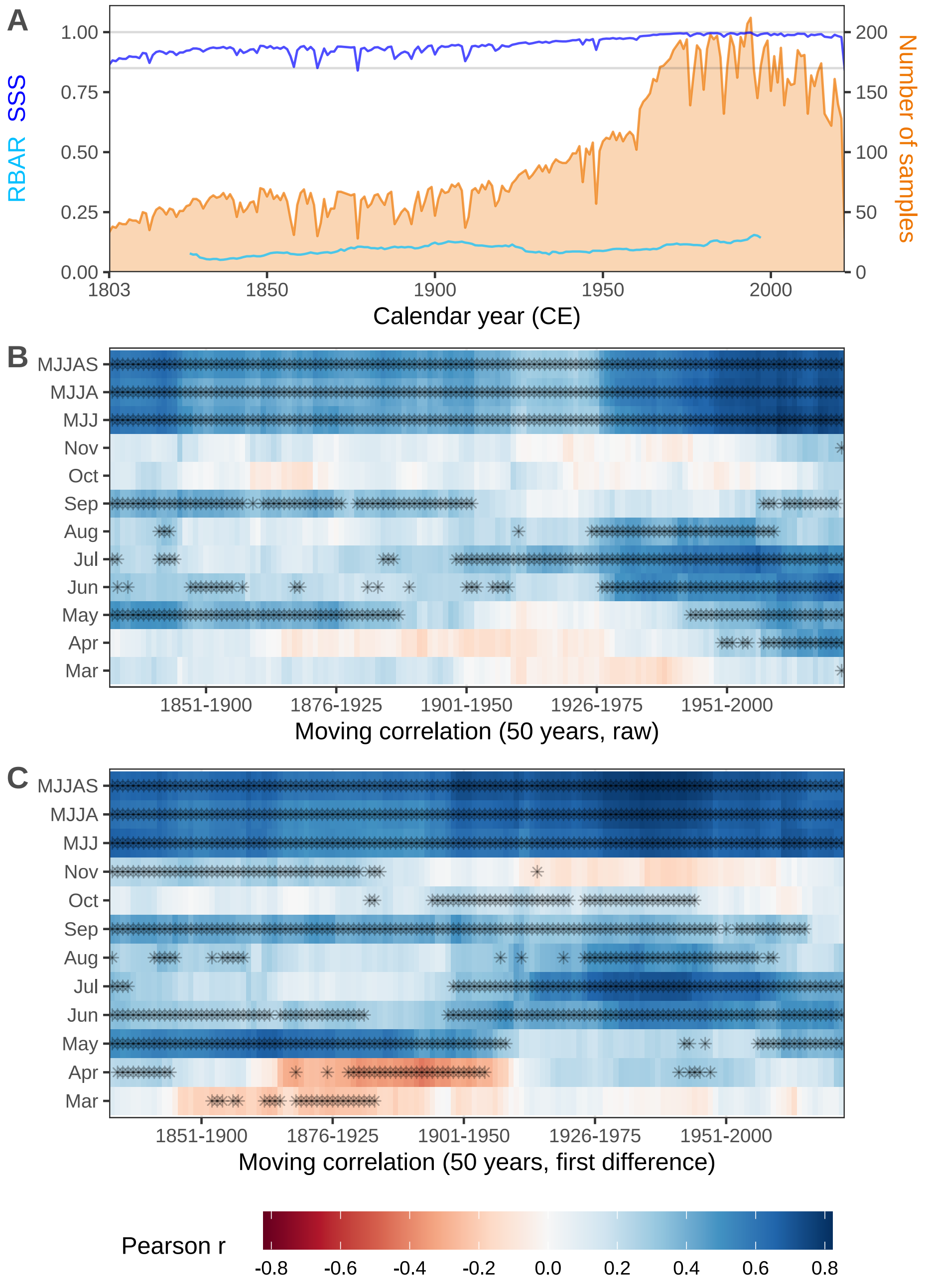

Figure S3: **50-year moving correlation between the latewood density chronology and growing season temperature.** (**A**) Subsample signal strength (SSS, dark blue), 50-year moving average pairwise correlation between series (rbar, light blue), and yearly sample depth (orange) of the latewood density chronology (1803–2022). SSS of 0.85 and 1 are indicated with gray lines. (**B**) 50-year moving correlation between monthly climate data from the Uccle weather station (1833–2022) and the latewood density chronology. Correlation with average May – September temperature (MJJAS) is shown at the top of each figure. Significant correlations ($p < 0.05$) are indicated with an asterisk. (**C**) Same as (B), but for high-frequency correlations where the chronology and instrumental data were first-difference transformed.

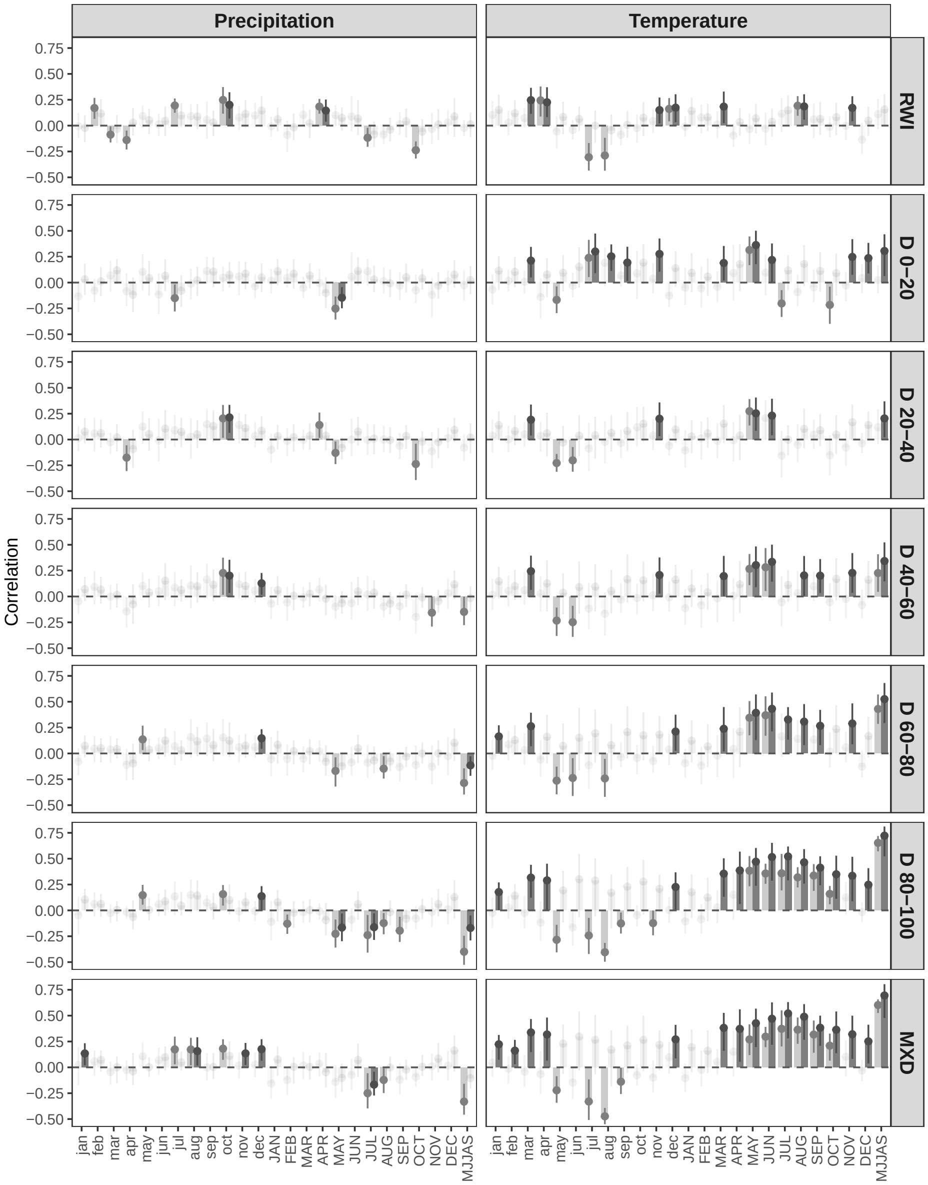

Figure S4: **Overview of correlations between climate and density in different ring sectors.** Correlation between monthly climate data from the Uccle weather station (1834–2022) and several micro-CT-based chronologies: ring width index (RWI), mean density in each of the five 20% wide ring sectors (D 0-20, D 20-40, D 40-60, D 60-80, and D 80-100, with D 80-100 representing latewood density in this study), and maximum latewood density (MXD). This both for the original chronologies (dark grey) and the first difference transformed chronologies (light grey). The whiskers represent the 95% confidence interval, with insignificant correlations being semi-transparent. Months written in all capital letters indicate the current growing season, while those in lowercase refer to the previous growing season. Chronology statistics can be found in table S2.

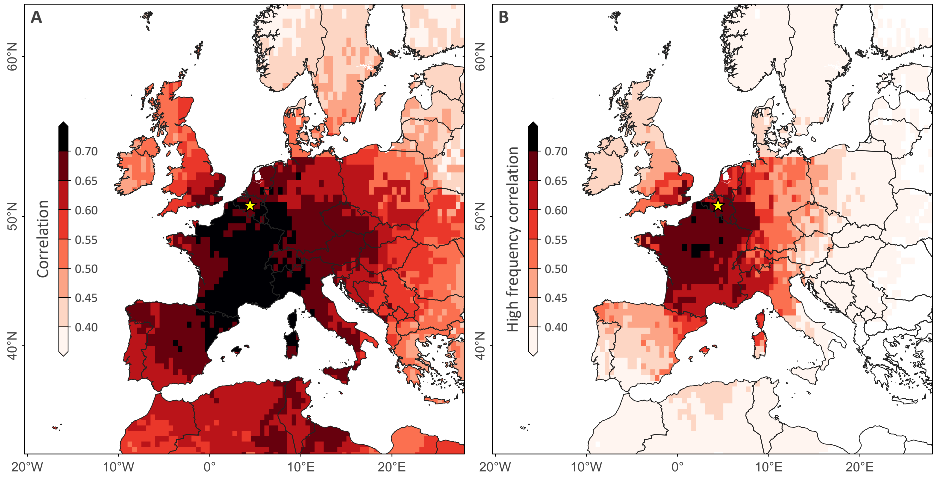

Figure S5: **Spatial correlation of the raw and detrended series.** (**A**) Spatial correlation between the latewood density chronology and 1901–2022 May–September mean temperature (CRU TS 4.08), only significant correlations are shown ($p < 0.05$). The study site is indicated with a yellow star. Raw chronology and climate data, not detrended. (**B**) The same as (A), but for the first-difference detrended time series.

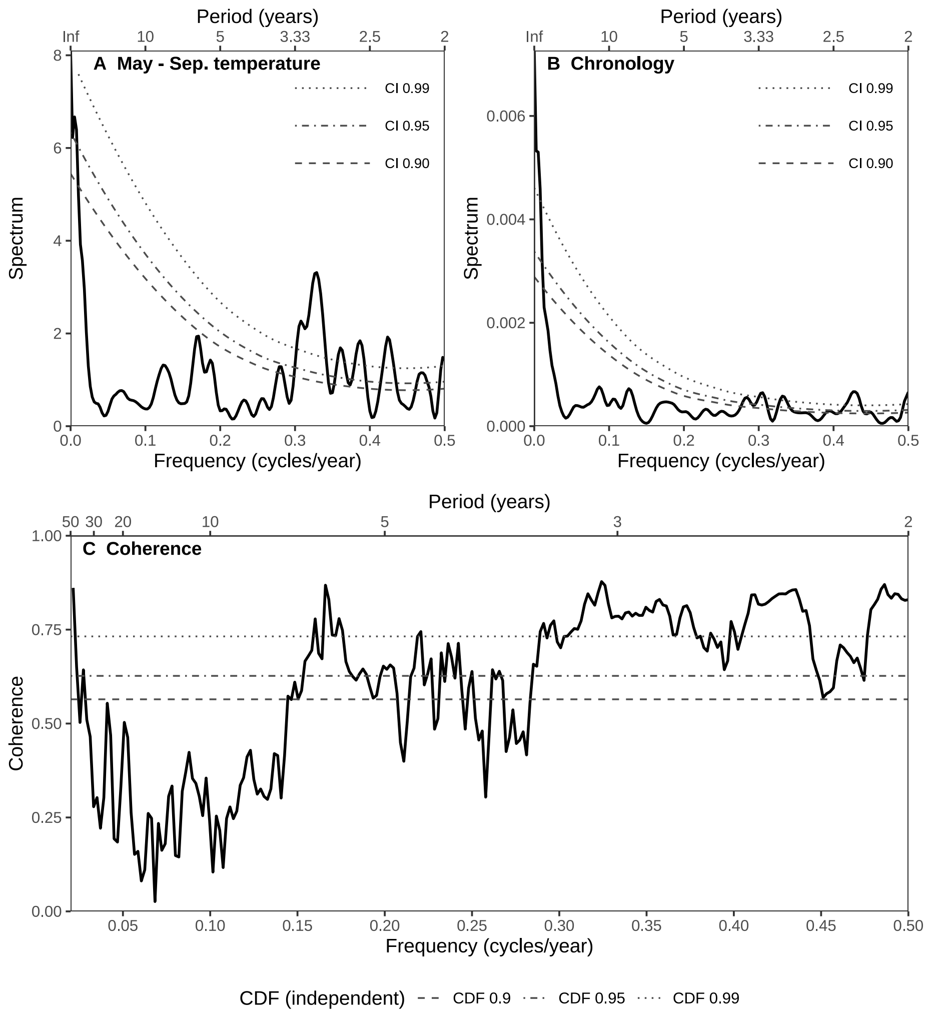

Figure S6: **Spectral characteristics and coherence between latewood density and growing-season temperature.** Spectra of (**A**) mean May–September temperature (1833–2022) and (**B**) the latewood density chronology (1833–2022) are shown as a function of frequency (bottom x-axis) and corresponding period in years (top x-axis). Solid black lines indicate the observed spectrum, while dashed lines denote the 90%, 95%, and 99% confidence levels against a red-noise null hypothesis. (**C**) Multitaper spectral coherence between the latewood density chronology and May–September temperature (1833–2022). Horizontal dashed lines indicate theoretical coherence thresholds corresponding to the 90%, 95%, and 99% cumulative distribution levels under the null hypothesis of independence.

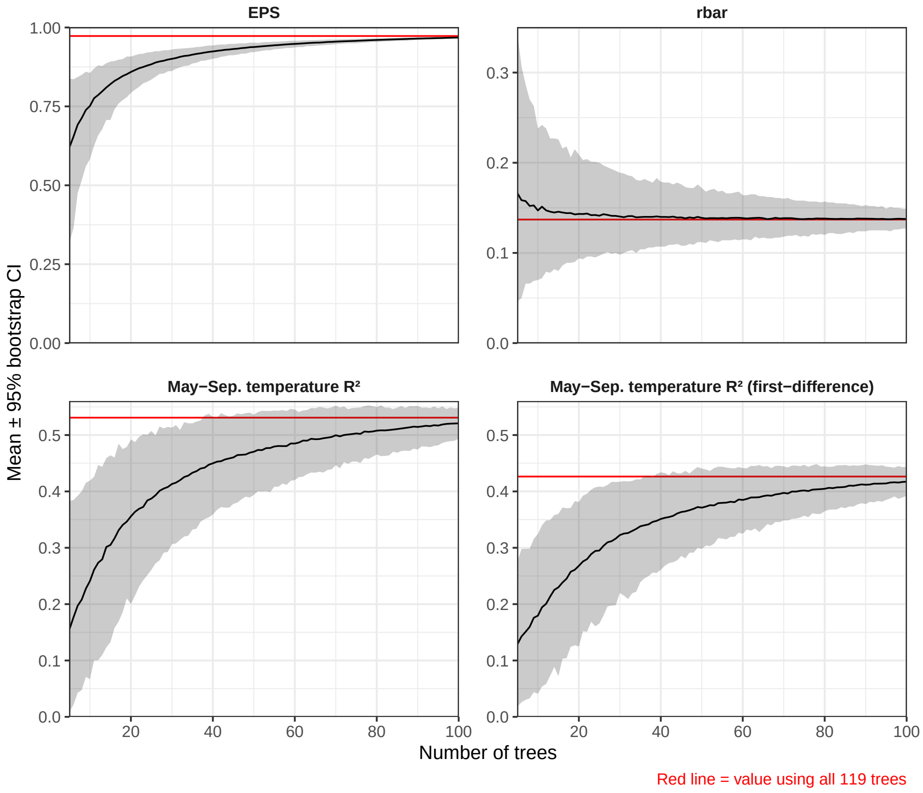

Figure S7: **Sample-size sensitivity of chronology statistics and climate correlations.** Mean chronology statistics (EPS and rbar) and May–September temperature variance explained by the chronology (R^2) as a function of the number of trees included in the analysis. Lines represent the mean across 1,000 bootstrap subsamples per sample size, and shaded areas indicate 95% bootstrap intervals. Red lines show values obtained from the full dataset (119 trees).

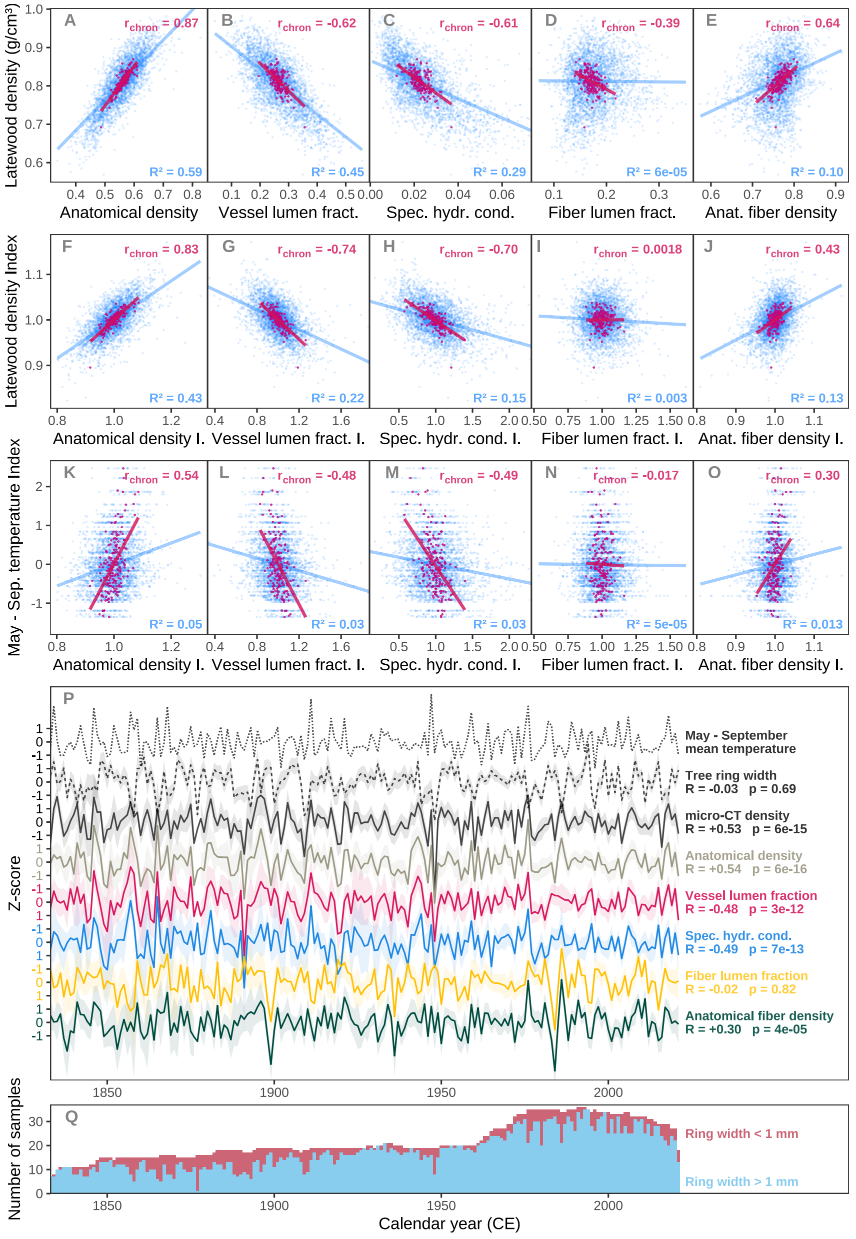

Figure S8: **Extended anatomical explanation of latewood density and its temperature signal.** (**a-e**) Scatter plots comparing micro-CT latewood density and optically measured anatomical parameters: anatomical density (1-porosity), vessel lumen area fraction, specific hydraulic conductivity (m^2 / (s × MPa), all others unitless), fiber lumen area fraction, and anatomical fiber density (anatomical density of only the cell walls and fiber lumen). All calculations were corrected to exclude the ray area. The fitted marginal regression with its marginal R^2 is in blue. Chronology values (annual mean), with the corresponding linear regression and correlation coefficient, are shown in red. (**f-j**) Same as in (a-e), but all parameters are detrended using a 30-year spline. (**k-o**) Scatter plots comparing detrended mean May–September temperature with detrended anatomical parameters (both detrended with 30-year smoothing splines). (**p**) The mean chronologies and 95% confidence intervals of all detrended parameters during the 1833–2021 instrumental period. May–September mean temperature (also detrended) is shown at the top. The correlation and p-value of each chronology to temperature is shown on the right. Vessel lumen area fraction, specific hydraulic conductivity, and fiber lumen area fraction are inverted for easier interpretation. Z-scores are calculated with 1833–2021 as the reference period. Chronology statistics can be found in table S2. (**q**) Yearly sample depth of the anatomical dataset, with rings narrower than 1 mm indicated in red.

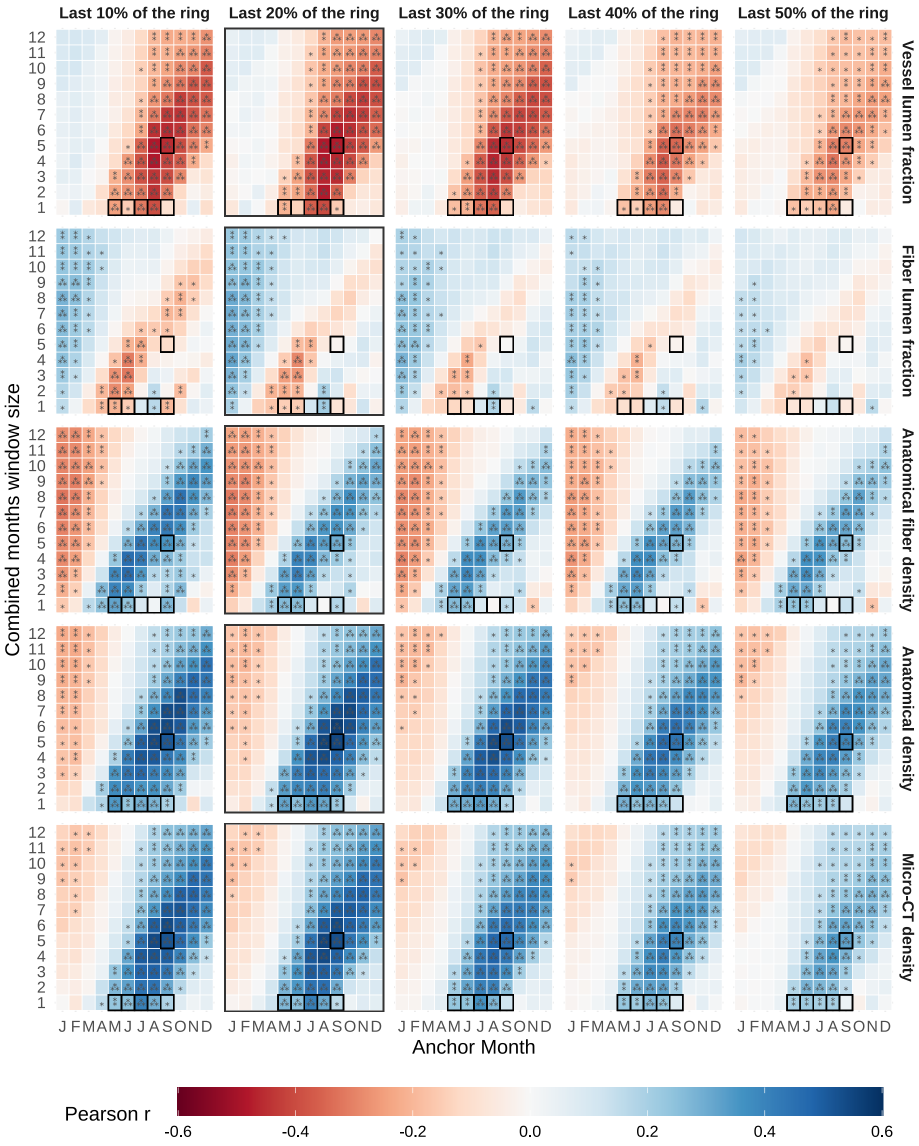

Figure S9: **Overview of correlations between temperature and latewood parameters.** Correlation between monthly and averaged month combination temperature data from the Uccle weather station (1834–2021) and the various latewood chronologies: vessel lumen area fraction, fiber lumen area fraction, anatomical fiber density (anatomical density of only the cell walls and fiber lumen), anatomical density (1-porosity), and micro-CT density. The y-axis represents different month combination sizes of averaged monthly temperature, the anchor month being the last month in the month combination. The columns represent differently sized sections of the ring as a definition for the latewood, with the 20% wide latewood results highlighted, as this is the definition of latewood used in the rest of this paper. Single-month correlations with May–September temperature, as well as average May–September temperature, are also highlighted, as these are the most sensitive months used in the rest of this paper. All tree-ring and temperature data for this figure were detrended with a 30-year smoothing spline. All chronologies were made with a minimum 1 mm ring width filter. Significant correlations are indicated with one, two, or three asterisks, corresponding to $p < 0.05$, $p < 0.01$, and $p < 0.001$, respectively.

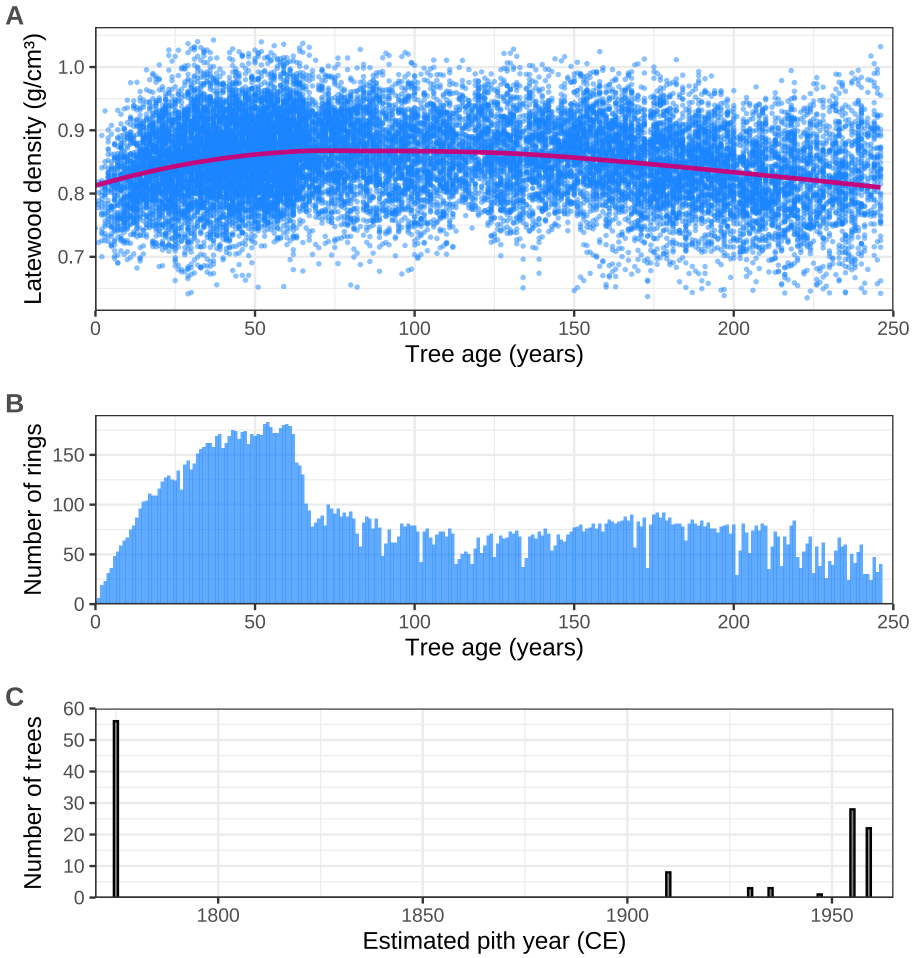

Figure S10: **Age-aligned density.** (**A**) Relationship between tree age and latewood density (g/cm^3). Blue points represent individual annual rings, and the red solid line shows a LOESS-smoothed trend highlighting the overall age-related pattern. (**B**) Frequency distribution of ring ages included in the analysis, showing the number of annual rings available for each tree age. (**C**) Frequency distribution of estimated pith years (CE) for all sampled trees, illustrating the temporal distribution of tree establishment within the dataset. Pith years were determined through a combination of estimating the number of missing rings to the pith and using known plantation years for this stand.

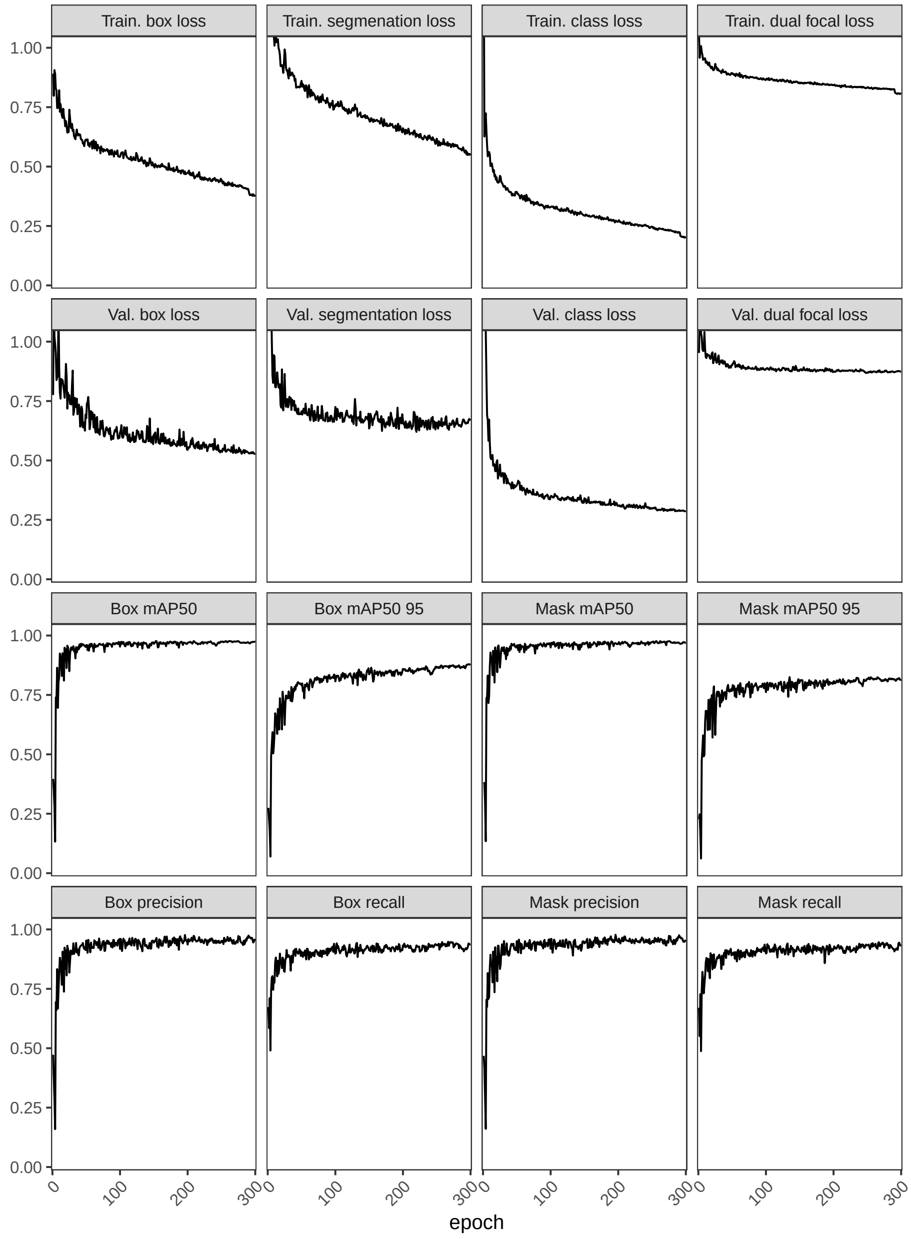

Figure S11: **YOLOv8l training statistics.** Performance statistics of the YOLOv8l segmentation model during training.

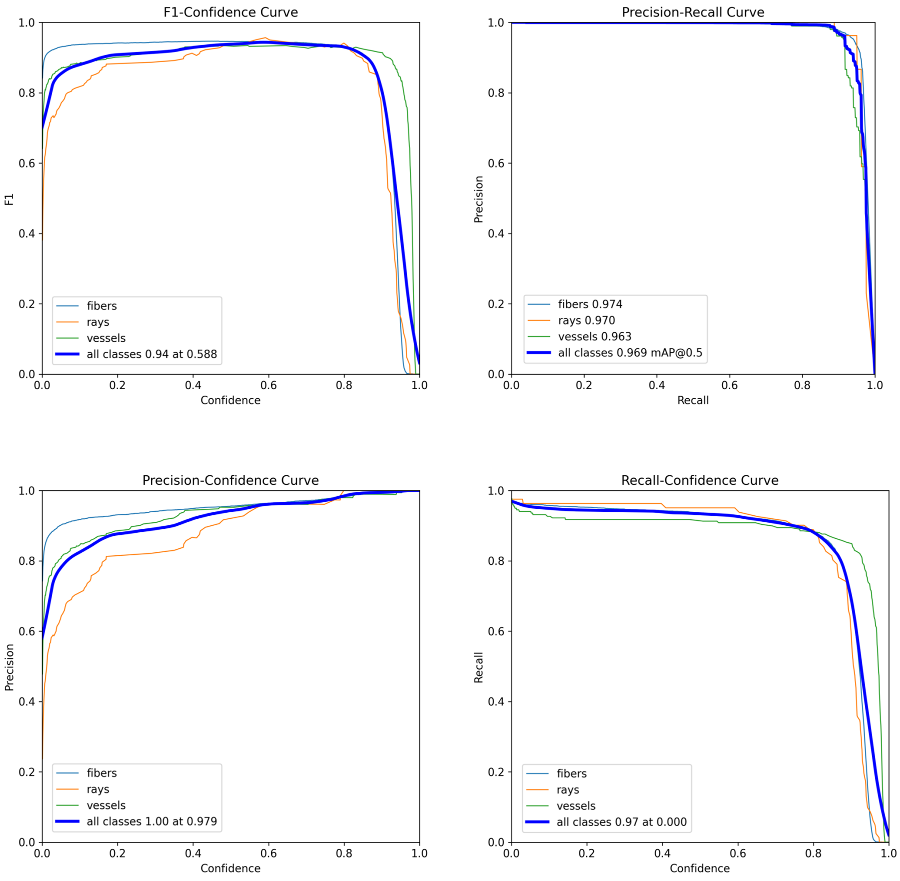

Figure S12: **YOLOv8l segmentation statistics.** Validation statistics of the YOLOv8l segmentation model masks.

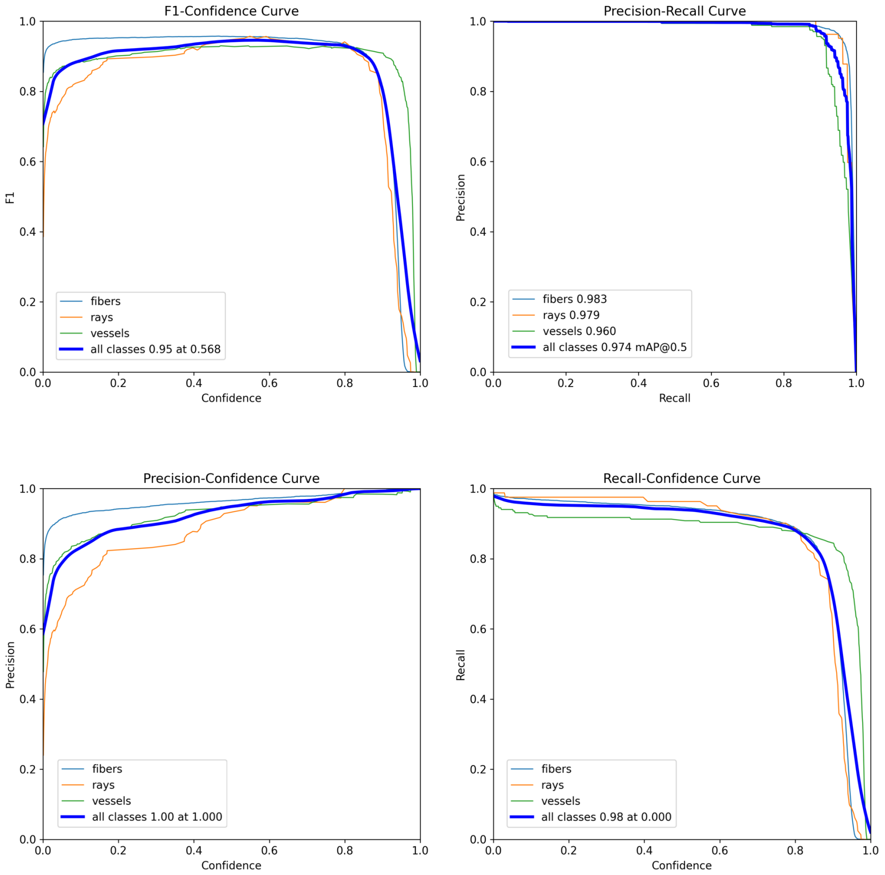

Figure S13: **YOLOv8l box statistics.** Validation statistics of the YOLOv8l segmentation model bounding boxes.

Data S1: COFECHA output of tree ring width from all used increment cores. The dating statistics were calculated by COFECHA for all the increment cores used in this study. This is the full raw output of the program using the default settings.

```
[]  Dendrochronology Program Library                                        Run ALL    Program COF  00:00   00     0000  Page   1
[]
[]  P R O G R A M      C O F E C H A                                                                      Version 6.06P      -30
-------------------------------------------------------------------------------------------------------------------------------------
QUALITY CONTROL AND DATING CHECK OF TREE-RING MEASUREMENTS
File of DATED series:   TRWALL.rwl
CONTENTS:
Part 1:  Title page, options selected, summary, absent rings by series
Part 2:  Histogram of time spans
Part 3:  Master series with sample depth and absent rings by year
Part 4:  Bar plot of Master Dating Series
Part 5:  Correlation by segment of each series with Master
Part 6:  Potential problems: low correlation, divergent year-to-year changes, absent rings, outliers
Part 7:  Descriptive statistics
RUN CONTROL OPTIONS SELECTED                             VALUE
1  Cubic smoothing spline 50                                                      32 years
2  Segments examined are                           50 years lagged successively by  25 years
3  Autoregressive model applied                     A  Residuals are used in master dating series and testing
4  Series transformed to logarithms                 Y  Each series log-transformed for master dating series and testing
5  CORRELATION is Pearson (parametric, quantitative)
Critical correlation, 99         6  Master dating series saved                      N
7  Ring measurements listed                         N
8  Parts printed                               1234567
9  Absent rings are omitted from master series and segment correlations  (Y)
Time span of Master dating series is  1774 to  2022   249 years
Continuous time span is              1774 to  2022   249 years
Portion with two or more series is    1776 to  2022   247 years
****************************************
*C* Number of dated series       237 *C*
*O* Master series 1774 2022  249 yrs *O*
*F* Total rings in all series  29807 *F*
*E* Total dated rings checked  29805 *E*
*C* Series intercorrelation    0.517 *C*
*H* Average mean sensitivity   0.307 *H*
*A* Segments, possible problems  187 *A*
*** Mean length of series      125.8 ***
****************************************
ABSENT RINGS listed by SERIES:            (See Master Dating Series for absent rings listed by year)
No ring measurements of zero value
PART 2:  TIME PLOT OF TREE-RING SERIES:                                                      00:00   00     0000  Page   2
-------------------------------------------------------------------------------------------------------------------------------------
1050 1100 1150 1200 1250 1300 1350 1400 1450 1500 1550 1600 1650 1700 1750 1800 1850 1900 1950 2000 2050 Ident     Seq Time-span  Yrs
:    :    :    :    :    :    :    :    :    :    :    :    :    :    :    :    :    :    :    :    :    -------- --- ---- ---- ----
.    .    .    .    .    .    .    .    .    .    .    .    .    .    .    <===================>    . L10011     1 1804 2005  202
.    .    .    .    .    .    .    .    .    .    .    .    .    .    .   <>    .    .    .    .    . L10012     2 1797 1807   11
.    .    .    .    .    .    .    .    .    .    .    .    .    .    .    .<===================>   . L10012     3 1810 2013  204
.    .    .    .    .    .    .    .    .    .    .    .    .    .    .    .<===================>   . L10021     4 1818 2018  201
.    .    .    .    .    .    .    .    .    .    .    .    .    .    .    . <==================>   . L10022     5 1826 2019  194
.    .    .    .    .    .    .    .    .    .    .    .    .    .    .   <=====================>   . L10061     6 1799 2019  221
.    .    .    .    .    .    .    .    .    .    .    .    .    .    .    .<===================>   . L10062     7 1812 2019  208
.    .    .    .    .    .    .    .    .    .    .    .    .    .    .    . <==================>   . L10071     8 1822 2018  197
.    .    .    .    .    .    .    .    .    .    .    .    .    .    .    <====================>   . L10072     9 1803 2018  216
.    .    .    .    .    .    .    .    .    .    .    .    .    .    .    .   <=================>  . L10081    10 1846 2020  175
.    .    .    .    .    .    .    .    .    .    .    .    .    .    .    <=====================>  . L10082    11 1808 2020  213
.    .    .    .    .    .    .    .    .    .    .    .    .    .    .    . <===================>  . L10101    12 1825 2020  196
.    .    .    .    .    .    .    .    .    .    .    .    .    .    .    .<====================>  . L10102    13 1814 2020  207
.    .    .    .    .    .    .    .    .    .    .    .    .    .    .    <====================>   . L10131    14 1804 2018  215
.    .    .    .    .    .    .    .    .    .    .    .    .    .    .    .    .  <============>   . L10132    15 1881 2018  138
.    .    .    .    .    .    .    .    .    .    .    .    .    .    .    <====================>   . L10141    16 1809 2018  210
.    .    .    .    .    .    .    .    .    .    .    .    .    .    .    . <==================>   . L10142    17 1822 2017  196
.    .    .    .    .    .    .    .    .    .    .    .    .    .    .   <=====================>   . L10151    18 1796 2019  224
.    .    .    .    .    .    .    .    .    .    .    .    .    .    .  <======================>   . L10152    19 1788 2019  232
.    .    .    .    .    .    .    .    .    .    .    .    .    .    .    .   <================>   . L10171    20 1847 2018  172
.    .    .    .    .    .    .    .    .    .    .    .    .    .    .    .  <=================>   . L10172    21 1834 2018  185
.    .    .    .    .    .    .    .    .    .    .    .    .    .    .    .    .    .<=========>   . L10181    22 1916 2019  104
.    .    .    .    .    .    .    .    .    .    .    .    .    .    .    <====================>   . L10182    23 1800 2019  220
.    .    .    .    .    .    .    .    .    .    .    .    .    .    .    .<===================>   . L10183    24 1818 2019  202
.    .    .    .    .    .    .    .    .    .    .    .    .    .    .    <====================>   . L10331    25 1808 2018  211
.    .    .    .    .    .    .    .    .    .    .    .    .    .    .    .    .    .    . <===>   . L10332    26 1975 2018   44
.    .    .    .    .    .    .    .    .    .    .    .    .    .    .    <===================>    . L7221     27 1800 2001  202
.    .    .    .    .    .    .    .    .    .    .    .    .    .    .   <===================>.    . L7222     28 1794 1997  204
.    .    .    .    .    .    .    .    .    .    .    .    .    .    .   <===================>.    . L7231     29 1792 1995  204
.    .    .    .    .    .    .    .    .    .    .    .    .    .    .    .    .    .<========>    . L7271     30 1916 2003   88
.    .    .    .    .    .    .    .    .    .    .    .    .    .    .    .<=================>.    . L7272     31 1815 1992  178
.    .    .    .    .    .    .    .    .    .    .    .    .    .    .   <====================>    . L7273     32 1798 2007  210
.    .    .    .    .    .    .    .    .    .    .    .    .    .    .    <====================>   . L7301     33 1809 2016  208
.    .    .    .    .    .    .    .    .    .    .    .    .    .    .   <=====================>   . L7302     34 1799 2012  214
.    .    .    .    .    .    .    .    .    .    .    .    .    .    .   <======================>  . SD1330    35 1792 2021  230
.    .    .    .    .    .    .    .    .    .    .    .    .    .    .    <=====================>  . SD1331    36 1804 2021  218
.    .    .    .    .    .    .    .    .    .    .    .    .    .    .    .    . <==============>  . SD1401    37 1870 2021  152
.    .    .    .    .    .    .    .    .    .    .    .    .    .    .    .   <=================>  . SD1402    38 1845 2021  177
.    .    .    .    .    .    .    .    .    .    .    .    .    .    .  <=======================>  . SD1621    39 1786 2021  236
.    .    .    .    .    .    .    .    .    .    .    .    .    .    .  <=======================>  . SD1622    40 1786 2021  236
.    .    .    .    .    .    .    .    .    .    .    .    .    .    .    <=====================>  . SD1650    41 1804 2021  218
.    .    .    .    .    .    .    .    .    .    .    .    .    .    .   <======================>  . SD1651    42 1798 2021  224
.    .    .    .    .    .    .    .    .    .    .    .    .    .    .    .    .    .  <========>  . SD1720    43 1937 2021   85
.    .    .    .    .    .    .    .    .    .    .    .    .    .    .    .    .    .  <========>  . SD1721    44 1934 2021   88
```

```
.    .    .    .    .    .    .    .    .    .    .    .    .    .    .   <======================>  . SD1750    45 1792 2021  230
.    .    .    .    .    .    .    .    .    .    .    .    .    .    .    .<====================>  . SD1751    46 1813 2021  209
.    .    .    .    .    .    .    .    .    .    .    .    .    .    .    .    .   <============>  . SD1752    47 1899 2021  123
.    .    .    .    .    .    .    .    .    .    .    .    .    .    .    <=====================>  . SD1753    48 1806 2021  216
.    .    .    .    .    .    .    .    .    .    .    .    .    .    .    <=====================>  . SD1754    49 1805 2021  217
.    .    .    .    .    .    .    .    .    .    .    .    .    .    .  <=======================>  . SD1755    50 1783 2021  239
:    :    :    :    :    :    :    :    :    :    :    :    :    :    :    :    :    :    :    :    :
1050 1100 1150 1200 1250 1300 1350 1400 1450 1500 1550 1600 1650 1700 1750 1800 1850 1900 1950 2000 2050
PART 2:  TIME PLOT OF TREE-RING SERIES:                                                  00:00   00     0000  Page   3
--------------------------------------------------------------------------------------------------------------------------------
1050 1100 1150 1200 1250 1300 1350 1400 1450 1500 1550 1600 1650 1700 1750 1800 1850 1900 1950 2000 2050 Ident   Seq Time-span  Yrs
:    :    :    :    :    :    :    :    :    :    :    :    :    :    :    :    :    :    :    :    : -------- --- ---- ---- ----
.    .    .    .    .    .    .    .    .    .    .    .    .    .    .    .   <=================>  . SD1920    51 1841 2021  181
.    .    .    .    .    .    .    .    .    .    .    .    .    .    .   <======================>  . SD1921    52 1798 2021  224
.    .    .    .    .    .    .    .    .    .    .    .    .    .    .    .  <==================>  . SD1930    53 1835 2021  187
.    .    .    .    .    .    .    .    .    .    .    .    .    .    .   <======================>  . SD1931    54 1798 2021  224
.    .    .    .    .    .    .    .    .    .    .    .    .    .    . <========================>  . SD1970    55 1774 2021  248
.    .    .    .    .    .    .    .    .    .    .    .    .    .    .  <=======================>  . SD1971    56 1788 2021  234
.    .    .    .    .    .    .    .    .    .    .    .    .    .    .    <=====================>  . SD1990    57 1800 2021  222
.    .    .    .    .    .    .    .    .    .    .    .    .    .    .    <=====================>  . SD1991    58 1802 2021  220
.    .    .    .    .    .    .    .    .    .    .    .    .    .    .   <======================>  . SD2210    59 1796 2021  226
.    .    .    .    .    .    .    .    .    .    .    .    .    .    .    <====================>   . SD2211    60 1800 2018  219
.    .    .    .    .    .    .    .    .    .    .    .    .    .    . <========================>  . SD2230    61 1776 2021  246
.    .    .    .    .    .    .    .    .    .    .    .    .    .    .   <======================>  . SD2231    62 1798 2021  224
.    .    .    .    .    .    .    .    .    .    .    .    .    .    .   <======================>  . SD2240    63 1795 2021  227
.    .    .    .    .    .    .    .    .    .    .    .    .    .    . <========================>  . SD2241    64 1779 2021  243
.    .    .    .    .    .    .    .    .    .    .    .    .    .    . <========================>  . SD2311    65 1777 2021  245
.    .    .    .    .    .    .    .    .    .    .    .    .    .    .   <======================>  . SD2312    66 1794 2021  228
.    .    .    .    .    .    .    .    .    .    .    .    .    .    .    . <===================>  . SD2501    67 1825 2021  197
.    .    .    .    .    .    .    .    .    .    .    .    .    .    .   <======================>  . SD2502    68 1797 2021  225
.    .    .    .    .    .    .    .    .    .    .    .    .    .    .    <=====================>  . SD2560    69 1802 2021  220
.    .    .    .    .    .    .    .    .    .    .    .    .    .    .    .<====================>  . SD2561    70 1813 2021  209
.    .    .    .    .    .    .    .    .    .    .    .    .    .    .    <=====================>  . SD2580    71 1804 2021  218
.    .    .    .    .    .    .    .    .    .    .    .    .    .    .   <======================>  . SD2581    72 1792 2021  230
.    .    .    .    .    .    .    .    .    .    .    .    .    .    .   <======================>  . SD2760    73 1793 2021  229
.    .    .    .    .    .    .    .    .    .    .    .    .    .    .  <=======================>  . SD2761    74 1788 2021  234
.    .    .    .    .    .    .    .    .    .    .    .    .    .    .    .<====================>  . SD2780    75 1816 2021  206
.    .    .    .    .    .    .    .    .    .    .    .    .    .    .    .  <=================>   . SD2781    76 1833 2014  182
.    .    .    .    .    .    .    .    .    .    .    .    .    .    .    <=====================>  . SD2791    77 1804 2021  218
.    .    .    .    .    .    .    .    .    .    .    .    .    .    .    .  <==================>  . SD2792    78 1833 2021  189
.    .    .    .    .    .    .    .    .    .    .    .    .    .    .    . <===================>  . SD2801    79 1824 2021  198
.    .    .    .    .    .    .    .    .    .    .    .    .    .    .    . <===================>  . SD2802    80 1823 2021  199
.    .    .    .    .    .    .    .    .    .    .    .    .    .    .    . <===================>  . SD2840    81 1827 2021  195
.    .    .    .    .    .    .    .    .    .    .    .    .    .    .    . <===================>  . SD2841    82 1822 2021  200
.    .    .    .    .    .    .    .    .    .    .    .    .    .    .   <======================>  . SD2851    83 1795 2021  227
.    .    .    .    .    .    .    .    .    .    .    .    .    .    .    .<====================>  . SD2852    84 1815 2021  207
.    .    .    .    .    .    .    .    .    .    .    .    .    .    .    .  <==================>  . SD2930    85 1833 2021  189
.    .    .    .    .    .    .    .    .    .    .    .    .    .    .    <=====================>  . SD2931    86 1805 2021  217
.    .    .    .    .    .    .    .    .    .    .    .    .    .    .    .<====================>  . SD3771    87 1818 2021  204
.    .    .    .    .    .    .    .    .    .    .    .    .    .    .  <=======================>  . SD3772    88 1785 2021  237
.    .    .    .    .    .    .    .    .    .    .    .    .    .    .    .   <=================>  . SD4361    89 1846 2021  176
.    .    .    .    .    .    .    .    .    .    .    .    .    .    .    .    . <==============>  . SD4362    90 1878 2021  144
.    .    .    .    .    .    .    .    .    .    .    .    .    .    .    .<====================>  . SD4521    91 1813 2021  209
.    .    .    .    .    .    .    .    .    .    .    .    .    .    .   <======================>  . SD4522    92 1797 2021  225
.    .    .    .    .    .    .    .    .    .    .    .    .    .    .    . <===================>  . SD4531    93 1829 2021  193
.    .    .    .    .    .    .    .    .    .    .    .    .    .    .    .    . <==============>  . SD4532    94 1871 2021  151
.    .    .    .    .    .    .    .    .    .    .    .    .    .    .    .<===================>   . SD4551    95 1819 2017  199
.    .    .    .    .    .    .    .    .    .    .    .    .    .    .   <=====================>   . SD4581    96 1797 2017  221
.    .    .    .    .    .    .    .    .    .    .    .    .    .    .    <====================>   . SD5341    97 1803 2017  215
.    .    .    .    .    .    .    .    .    .    .    .    .    .    .    .    <===============>   . SD6311    98 1856 2016  161
.    .    .    .    .    .    .    .    .    .    .    .    .    .    .    .    <================>  . SD6681    99 1851 2021  171
.    .    .    .    .    .    .    .    .    .    .    .    .    .    .    .   <=================>  . SD6682   100 1848 2021  174
:    :    :    :    :    :    :    :    :    :    :    :    :    :    :    :    :    :    :    :    :
1050 1100 1150 1200 1250 1300 1350 1400 1450 1500 1550 1600 1650 1700 1750 1800 1850 1900 1950 2000 2050
PART 2:  TIME PLOT OF TREE-RING SERIES:                                                  00:00   00     0000  Page   4
--------------------------------------------------------------------------------------------------------------------------------
1050 1100 1150 1200 1250 1300 1350 1400 1450 1500 1550 1600 1650 1700 1750 1800 1850 1900 1950 2000 2050 Ident   Seq Time-span  Yrs
:    :    :    :    :    :    :    :    :    :    :    :    :    :    :    :    :    :    :    :    : -------- --- ---- ---- ----
.    .    .    .    .    .    .    .    .    .    .    .    .    .    .   <======================>  . SD7221   101 1795 2021  227
.    .    .    .    .    .    .    .    .    .    .    .    .    .    .  <=======================>  . SD7222   102 1780 2021  242
.    .    .    .    .    .    .    .    .    .    .    .    .    .    .    .    . <==============>  . SD81     103 1873 2021  149
.    .    .    .    .    .    .    .    .    .    .    .    .    .    .    .   <=================>  . SD82     104 1841 2021  181
.    .    .    .    .    .    .    .    .    .    .    .    .    .    .    .   <================>   . SD83     105 1843 2017  175
.    .    .    .    .    .    .    .    .    .    .    .    .    .    . <=======================>   . SD8601   106 1777 2017  241
.    .    .    .    .    .    .    .    .    .    .    .    .    .    .    .<====================>  . SD9531   107 1811 2021  211
.    .    .    .    .    .    .    .    .    .    .    .    .    .    .    .   <=================>  . SD9532   108 1849 2021  173
.    .    .    .    .    .    .    .    .    .    .    .    .    .    .    .    .    .   <=======>  . NG1070   109 1941 2021   81
.    .    .    .    .    .    .    .    .    .    .    .    .    .    .    .    .    .   <=======>  . NG1071   110 1941 2021   81
.    .    .    .    .    .    .    .    .    .    .    .    .    .    .    .    .    .    <======>  . NG1110   111 1956 2021   66
.    .    .    .    .    .    .    .    .    .    .    .    .    .    .    .    .    .    .<=====>  . NG1111   112 1965 2021   57
.    .    .    .    .    .    .    .    .    .    .    .    .    .    .    .    .    .    <======>  . NG1120   113 1952 2021   70
.    .    .    .    .    .    .    .    .    .    .    .    .    .    .    .    .    .   <=======>  . NG1121   114 1943 2021   79
.    .    .    .    .    .    .    .    .    .    .    .    .    .    .    .    .    .    .<=====>  . NG1180   115 1961 2021   61
.    .    .    .    .    .    .    .    .    .    .    .    .    .    .    .    .    .    .<=====>  . NG1181   116 1963 2021   59
.    .    .    .    .    .    .    .    .    .    .    .    .    .    .    .    .    .    .<=====>  . NG1182   117 1963 2021   59
.    .    .    .    .    .    .    .    .    .    .    .    .    .    .    .    .    .    .<=====>  . NG1183   118 1968 2021   54
.    .    .    .    .    .    .    .    .    .    .    .    .    .    .    .    .    .    .<=====>  . NG1190   119 1964 2021   58
.    .    .    .    .    .    .    .    .    .    .    .    .    .    .    .    .    .    <======>  . NG1191   120 1957 2021   65
.    .    .    .    .    .    .    .    .    .    .    .    .    .    .    .    .    .    . <====>  . NG1280   121 1971 2021   51
.    .    .    .    .    .    .    .    .    .    .    .    .    .    .    .    .    .    . <====>  . NG1281   122 1972 2021   50
.    .    .    .    .    .    .    .    .    .    .    .    .    .    .    .    .    .    .<=====>  . NG1282   123 1962 2022   61
.    .    .    .    .    .    .    .    .    .    .    .    .    .    .    .    .    .    . <====>  . NG1283   124 1976 2022   47
```

```
.    .    .    .    .    .    .    .    .    .    .    .    .    .    .    .    .    .    .<=====>  . NG1290   125 1961 2022   62
.    .    .    .    .    .    .    .    .    .    .    .    .    .    .    .    .    .    .<=====>  . NG1291   126 1965 2022   58
.    .    .    .    .    .    .    .    .    .    .    .    .    .    .    .    .    .    .<=====>  . NG1320   127 1964 2021   58
.    .    .    .    .    .    .    .    .    .    .    .    .    .    .    .    .    .    .<=====>  . NG1321   128 1966 2021   56
.    .    .    .    .    .    .    .    .    .    .    .    .    .    .    .    .    .    .<=====>  . NG1400   129 1968 2022   55
.    .    .    .    .    .    .    .    .    .    .    .    .    .    .    .    .    .    .<=====>  . NG1401   130 1967 2022   56
.    .    .    .    .    .    .    .    .    .    .    .    .    .    .    .    .    .    .<=====>  . NG1420   131 1968 2022   55
.    .    .    .    .    .    .    .    .    .    .    .    .    .    .    .    .    .    .<=====>  . NG1421   132 1969 2022   54
.    .    .    .    .    .    .    .    .    .    .    .    .    .    .    .    .    .    .<=====>  . NG1422   133 1969 2022   54
.    .    .    .    .    .    .    .    .    .    .    .    .    .    .    .    .    .    . <====>  . NG1423   134 1971 2022   52
.    .    .    .    .    .    .    .    .    .    .    .    .    .    .    .    .    .    .<=====>  . NG1430   135 1968 2022   55
.    .    .    .    .    .    .    .    .    .    .    .    .    .    .    .    .    .    <======>  . NG1431   136 1958 2022   65
.    .    .    .    .    .    .    .    .    .    .    .    .    .    .    .    .    .    <======>  . NG1440   137 1959 2022   64
.    .    .    .    .    .    .    .    .    .    .    .    .    .    .    .    .    .    .<=====>  . NG1441   138 1968 2022   55
.    .    .    .    .    .    .    .    .    .    .    .    .    .    .    .    .    .    . <====>  . NG1470   139 1975 2022   48
.    .    .    .    .    .    .    .    .    .    .    .    .    .    .    .    .    .    .<=====>  . NG1471   140 1966 2022   57
.    .    .    .    .    .    .    .    .    .    .    .    .    .    .    .    .    .    .<====>   . NG151    141 1968 2017   50
.    .    .    .    .    .    .    .    .    .    .    .    .    .    .    .    .    .    .<=====>  . NG1580   142 1962 2021   60
.    .    .    .    .    .    .    .    .    .    .    .    .    .    .    .    .    .    .<=====>  . NG1581   143 1967 2021   55
.    .    .    .    .    .    .    .    .    .    .    .    .    .    .    .    .    .    <======>  . NG2640   144 1958 2021   64
.    .    .    .    .    .    .    .    .    .    .    .    .    .    .    .    .    .    <======>  . NG2641   145 1959 2021   63
.    .    .    .    .    .    .    .    .    .    .    .    .    .    .    .    .    .    <======>  . NG2650   146 1952 2021   70
.    .    .    .    .    .    .    .    .    .    .    .    .    .    .    .    .    .    .<=====>  . NG2651   147 1962 2021   60
.    .    .    .    .    .    .    .    .    .    .    .    .    .    .    .    .    .    .<=====>  . NG2710   148 1968 2021   54
.    .    .    .    .    .    .    .    .    .    .    .    .    .    .    .    .    .    .<=====>  . NG2711   149 1967 2021   55
.    .    .    .    .    .    .    .    .    .    .    .    .    .    .    .    .    .    . <====>  . NG2712   150 1970 2021   52
:    :    :    :    :    :    :    :    :    :    :    :    :    :    :    :    :    :    :    :    :
1050 1100 1150 1200 1250 1300 1350 1400 1450 1500 1550 1600 1650 1700 1750 1800 1850 1900 1950 2000 2050
PART 2:  TIME PLOT OF TREE-RING SERIES:                                                   00:00   00     0000  Page   5
------------------------------------------------------------------------------------------------------------------------
1050 1100 1150 1200 1250 1300 1350 1400 1450 1500 1550 1600 1650 1700 1750 1800 1850 1900 1950 2000 2050 Ident   Seq Time-span  Yrs
:    :    :    :    :    :    :    :    :    :    :    :    :    :    :    :    :    :    :    :    : -------- --- ---- ---- ----
.    .    .    .    .    .    .    .    .    .    .    .    .    .    .    .    .    .    .<=====>  . NG2713   151 1965 2021   57
.    .    .    .    .    .    .    .    .    .    .    .    .    .    .    .    .    .    .<=====>  . NG2800   152 1967 2021   55
.    .    .    .    .    .    .    .    .    .    .    .    .    .    .    .    .    .    <======>  . NG2801   153 1958 2021   64
.    .    .    .    .    .    .    .    .    .    .    .    .    .    .    .    .    .   <=======>  . NG2900   154 1948 2021   74
.    .    .    .    .    .    .    .    .    .    .    .    .    .    .    .    .    .    .<=====>  . NG2901   155 1960 2021   62
.    .    .    .    .    .    .    .    .    .    .    .    .    .    .    .    .    .    <======>  . NG2940   156 1957 2021   65
.    .    .    .    .    .    .    .    .    .    .    .    .    .    .    .    .    .    <======>  . NG2941   157 1957 2020   64
.    .    .    .    .    .    .    .    .    .    .    .    .    .    .    .    .    .    <======>  . NG3020   158 1955 2021   67
.    .    .    .    .    .    .    .    .    .    .    .    .    .    .    .    .    .    <======>  . NG3021   159 1951 2021   71
.    .    .    .    .    .    .    .    .    .    .    .    .    .    .    .    .    .    .<=====>  . NG3040   160 1960 2021   62
.    .    .    .    .    .    .    .    .    .    .    .    .    .    .    .    .    .    . <====>  . NG3041   161 1972 2021   50
.    .    .    .    .    .    .    .    .    .    .    .    .    .    .    .    .    .    .<=====>  . NG3051   162 1960 2021   62
.    .    .    .    .    .    .    .    .    .    .    .    .    .    .    .    .    .    <======>  . NG3052   163 1953 2021   69
.    .    .    .    .    .    .    .    .    .    .    .    .    .    .    .    .    .    <======>  . NG3101   164 1951 2021   71
.    .    .    .    .    .    .    .    .    .    .    .    .    .    .    .    .    .    <======>  . NG3102   165 1956 2021   66
.    .    .    .    .    .    .    .    .    .    .    .    .    .    .    .    .    .    .  <===>  . NG3571   166 1983 2021   39
.    .    .    .    .    .    .    .    .    .    .    .    .    .    .    .    .    .    .   <==>  . NG3572   167 1991 2021   31
.    .    .    .    .    .    .    .    .    .    .    .    .    .    .    .    .    .    . <====>  . NG3581   168 1970 2021   52
.    .    .    .    .    .    .    .    .    .    .    .    .    .    .    .    .    .    . <====>  . NG3582   169 1975 2021   47
.    .    .    .    .    .    .    .    .    .    .    .    .    .    .    .    .    .   <=======>  . NG4141   170 1946 2020   75
.    .    .    .    .    .    .    .    .    .    .    .    .    .    .    .    .    .    <======>  . NG4142   171 1950 2021   72
.    .    .    .    .    .    .    .    .    .    .    .    .    .    .    .    .    .    .<=====>  . WG11     172 1964 2022   59
.    .    .    .    .    .    .    .    .    .    .    .    .    .    .    .    .    .    .<=====>  . WG12     173 1968 2022   55
.    .    .    .    .    .    .    .    .    .    .    .    .    .    .    .    .    .    .<=====>  . WG101    174 1964 2022   59
.    .    .    .    .    .    .    .    .    .    .    .    .    .    .    .    .    .    .<=====>  . WG102    175 1961 2022   62
.    .    .    .    .    .    .    .    .    .    .    .    .    .    .    .    .    . <=========>  . WG111    176 1928 2022   95
.    .    .    .    .    .    .    .    .    .    .    .    .    .    .    .    .    .  <========>  . WG112    177 1933 2022   90
.    .    .    .    .    .    .    .    .    .    .    .    .    .    .    .    .    .    .<=====>  . WG121    178 1962 2022   61
.    .    .    .    .    .    .    .    .    .    .    .    .    .    .    .    .    .    . <====>  . WG122    179 1973 2022   50
.    .    .    .    .    .    .    .    .    .    .    .    .    .    .    .    .    .    .<=====>  . WG131    180 1966 2022   57
.    .    .    .    .    .    .    .    .    .    .    .    .    .    .    .    .    .    <======>  . WG132    181 1959 2022   64
.    .    .    .    .    .    .    .    .    .    .    .    .    .    .    .    .    .    . <====>  . WG141    182 1971 2022   52
.    .    .    .    .    .    .    .    .    .    .    .    .    .    .    .    .    .    .<=====>  . WG142    183 1963 2022   60
.    .    .    .    .    .    .    .    .    .    .    .    .    .    .    .    .    .    <======>  . WG151    184 1959 2022   64
.    .    .    .    .    .    .    .    .    .    .    .    .    .    .    .    .    .    .<=====>  . WG152    185 1961 2022   62
.    .    .    .    .    .    .    .    .    .    .    .    .    .    .    .    .    .    . <====>  . WG161    186 1971 2022   52
.    .    .    .    .    .    .    .    .    .    .    .    .    .    .    .    .    .    .<=====>  . WG162    187 1963 2022   60
.    .    .    .    .    .    .    .    .    .    .    .    .    .    .    .    .    .    <======>  . WG171    188 1957 2022   66
.    .    .    .    .    .    .    .    .    .    .    .    .    .    .    .    .    .    .<=====>  . WG172    189 1963 2022   60
.    .    .    .    .    .    .    .    .    .    .    .    .    .    .    .    .    . <=========>  . WG181    190 1927 2022   96
.    .    .    .    .    .    .    .    .    .    .    .    .    .    .    .    .    . <=========>  . WG182    191 1921 2022  102
.    .    .    .    .    .    .    .    .    .    .    .    .    .    .    .    .    .    .<=====>  . WG191    192 1964 2022   59
.    .    .    .    .    .    .    .    .    .    .    .    .    .    .    .    .    .    .<=====>  . WG192    193 1967 2022   56
.    .    .    .    .    .    .    .    .    .    .    .    .    .    .    .    .    .    <======>  . WG21     194 1959 2022   64
.    .    .    .    .    .    .    .    .    .    .    .    .    .    .    .    .    .    <======>  . WG22     195 1959 2022   64
.    .    .    .    .    .    .    .    .    .    .    .    .    .    .    .    .    .    . <====>  . WG201    196 1972 2022   51
.    .    .    .    .    .    .    .    .    .    .    .    .    .    .    .    .    .    .<=====>  . WG202    197 1960 2022   63
.    .    .    .    .    .    .    .    .    .    .    .    .    .    .    .    .    .    <======>  . WG211    198 1957 2022   66
.    .    .    .    .    .    .    .    .    .    .    .    .    .    .    .    .    .    <======>  . WG212    199 1957 2022   66
.    .    .    .    .    .    .    .    .    .    .    .    .    .    .    .    .    . <=========>  . WG221    200 1927 2022   96
:    :    :    :    :    :    :    :    :    :    :    :    :    :    :    :    :    :    :    :    :
1050 1100 1150 1200 1250 1300 1350 1400 1450 1500 1550 1600 1650 1700 1750 1800 1850 1900 1950 2000 2050
PART 2:  TIME PLOT OF TREE-RING SERIES:                                                   00:00   00     0000  Page   6
------------------------------------------------------------------------------------------------------------------------
1050 1100 1150 1200 1250 1300 1350 1400 1450 1500 1550 1600 1650 1700 1750 1800 1850 1900 1950 2000 2050 Ident   Seq Time-span  Yrs
:    :    :    :    :    :    :    :    :    :    :    :    :    :    :    :    :    :    :    :    : -------- --- ---- ---- ----
.    .    .    .    .    .    .    .    .    .    .    .    .    .    .    .    .    .   <=======>  . WG222    201 1946 2022   77
.    .    .    .    .    .    .    .    .    .    .    .    .    .    .    .    .    .    .<=====>  . WG231    202 1961 2022   62
.    .    .    .    .    .    .    .    .    .    .    .    .    .    .    .    .    .    .<=====>  . WG232    203 1962 2022   61
.    .    .    .    .    .    .    .    .    .    .    .    .    .    .    .    .    .<==========>  . WG241    204 1916 2022  107
```

```
.    .    .    .    .    .    .    .    .    .    .    .    .    .    .    .    .    .<==========>  . WG242    205 1914 2022  109
.    .    .    .    .    .    .    .    .    .    .    .    .    .    .    .    .    .    <======>  . WG251    206 1954 2022   69
.    .    .    .    .    .    .    .    .    .    .    .    .    .    .    .    .    .  <========>  . WG252    207 1938 2022   85
.    .    .    .    .    .    .    .    .    .    .    .    .    .    .    .    .    .    .<=====>  . WG261    208 1962 2022   61
.    .    .    .    .    .    .    .    .    .    .    .    .    .    .    .    .    .    .<=====>  . WG262    209 1964 2022   59
.    .    .    .    .    .    .    .    .    .    .    .    .    .    .    .    .    .    <======>  . WG271    210 1953 2022   70
.    .    .    .    .    .    .    .    .    .    .    .    .    .    .    .    .    .    <======>  . WG272    211 1954 2022   69
.    .    .    .    .    .    .    .    .    .    .    .    .    .    .    .    .    .    .<=====>  . WG281    212 1960 2022   63
.    .    .    .    .    .    .    .    .    .    .    .    .    .    .    .    .    .    .<=====>  . WG282    213 1964 2022   59
.    .    .    .    .    .    .    .    .    .    .    .    .    .    .    .    .    . <=========>  . WG291    214 1920 2022  103
.    .    .    .    .    .    .    .    .    .    .    .    .    .    .    .    .    .  <========>  . WG292    215 1933 2022   90
.    .    .    .    .    .    .    .    .    .    .    .    .    .    .    .    .    .    .<=====>  . WG31     216 1967 2022   56
.    .    .    .    .    .    .    .    .    .    .    .    .    .    .    .    .    .    .<=====>  . WG32     217 1961 2022   62
.    .    .    .    .    .    .    .    .    .    .    .    .    .    .    .    .    .    .<=====>  . WG301    218 1969 2022   54
.    .    .    .    .    .    .    .    .    .    .    .    .    .    .    .    .    .    .<=====>  . WG302    219 1960 2022   63
.    .    .    .    .    .    .    .    .    .    .    .    .    .    .    .    .    .    .<=====>  . WG311    220 1964 2022   59
.    .    .    .    .    .    .    .    .    .    .    .    .    .    .    .    .    .    .<=====>  . WG312    221 1963 2022   60
.    .    .    .    .    .    .    .    .    .    .    .    .    .    .    .    .    .    .<=====>  . WG321    222 1967 2022   56
.    .    .    .    .    .    .    .    .    .    .    .    .    .    .    .    .    .    <======>  . WG322    223 1959 2022   64
.    .    .    .    .    .    .    .    .    .    .    .    .    .    .    .    .    .   <=======>  . WG331    224 1942 2022   81
.    .    .    .    .    .    .    .    .    .    .    .    .    .    .    .    .    .   <=======>  . WG332    225 1945 2022   78
.    .    .    .    .    .    .    .    .    .    .    .    .    .    .    .    .    .    .<=====>  . WG41     226 1963 2022   60
.    .    .    .    .    .    .    .    .    .    .    .    .    .    .    .    .    .    .<=====>  . WG42     227 1961 2022   62
.    .    .    .    .    .    .    .    .    .    .    .    .    .    .    .    .    .    <======>  . WG51     228 1958 2022   65
.    .    .    .    .    .    .    .    .    .    .    .    .    .    .    .    .    .    .<=====>  . WG52     229 1965 2022   58
.    .    .    .    .    .    .    .    .    .    .    .    .    .    .    .    .    . <=========>  . WG61     230 1925 2022   98
.    .    .    .    .    .    .    .    .    .    .    .    .    .    .    .    .    .  <========>  . WG62     231 1933 2022   90
.    .    .    .    .    .    .    .    .    .    .    .    .    .    .    .    .    .   <=======>  . WG71     232 1940 2022   83
.    .    .    .    .    .    .    .    .    .    .    .    .    .    .    .    .    .   <=======>  . WG72     233 1941 2022   82
.    .    .    .    .    .    .    .    .    .    .    .    .    .    .    .    .    .    .<=====>  . WG81     234 1963 2022   60
.    .    .    .    .    .    .    .    .    .    .    .    .    .    .    .    .    .    <======>  . WG82     235 1958 2022   65
.    .    .    .    .    .    .    .    .    .    .    .    .    .    .    .    .    .   <=======>  . WG91     236 1940 2022   83
.    .    .    .    .    .    .    .    .    .    .    .    .    .    .    .    .    .   <=======>  . WG92     237 1941 2022   82
:    :    :    :    :    :    :    :    :    :    :    :    :    :    :    :    :    :    :    :    :
1050 1100 1150 1200 1250 1300 1350 1400 1450 1500 1550 1600 1650 1700 1750 1800 1850 1900 1950 2000 2050
```

```
PART 3:  Master Dating Series:                                                           00:00   00     0000  Page   7
-------------------------------------------------------------------------------------------------------------------------------------
Year  Value  No Ab    Year  Value  No Ab    Year  Value  No Ab    Year  Value  No Ab    Year  Value  No Ab    Year  Value  No Ab
------------------    ------------------    ------------------    ------------------    ------------------    ------------------
1800 -0.039  40       1850  0.396  94       1900 -1.389 102       1950  0.876 131       2000 -0.526 233
1801  0.612  40       1851  1.430  95       1901 -0.047 102       1951  0.884 133       2001  0.074 233
1802 -4.190  42       1852  0.149  95       1902  0.248 102       1952  0.747 135       2002 -0.296 232
1803 -0.367  44       1853  0.790  95       1903  0.139 102       1953  1.206 137       2003  0.816 232
1804  1.342  50       1854  0.070  95       1904  0.539 102       1954  0.597 139       2004 -0.753 231
1805  0.313  52       1855  0.718  95       1905  1.093 102       1955  1.266 140       2005 -0.233 231
1806  0.117  53       1856  0.367  96       1906  0.842 102       1956 -0.386 142       2006 -0.219 230
1807  1.072  53       1857 -1.351  96       1907  1.154 102       1957 -0.411 148       2007 -0.834 230
1808  1.089  54       1858 -2.619  96       1908  0.157 102       1958  0.180 153       2008  0.684 229
1809  0.690  56       1859  0.414  96       1909 -2.554 102       1959 -0.770 160       2009  0.850 229
1810  0.482  57       1860  0.848  96       1910 -1.812 102       1960 -1.600 166       2010  0.578 229
1811  1.085  58       1861  1.275  96       1911  0.503 102       1961 -0.093 173       2011 -0.787 229
1812 -0.191  59       1862  0.269  96       1912  0.065 102       1962  0.625 179       2012  0.235 229
1813  0.671  62       1863  1.244  96       1913  0.252 102       1963 -0.139 187       2013 -0.336 228
1814 -0.285  63       1864 -0.027  96       1914  0.861 103       1964  0.303 195       2014  0.541 227
1815 -3.689  65       1865 -2.404  96       1915 -0.048 103       1965  0.570 199       2015  0.689 226
1816 -1.140  66       1866 -1.019  96       1916  0.815 106       1966  0.463 202       2016 -1.140 226
1817  0.845  66       1867  0.564  96       1917  0.047 106       1967  0.474 209       2017 -0.811 224
1818  0.113  69       1868 -0.984  96       1918 -1.218 106       1968  0.411 217       2018 -0.805 217
1819 -0.027  70       1869 -0.201  96       1919 -1.118 106       1969  0.241 220       2019  1.024 206
1820 -1.066  70       1870 -0.042  97       1920  0.312 107       1970 -0.167 222       2020  0.447 198
1821  0.298  70       1871  0.872  98       1921 -0.610 108       1971  0.421 226       2021  0.622 192
1822 -0.294  73       1872  0.891  98       1922 -0.744 108       1972 -0.188 229       2022  0.083  82
1823 -1.348  74       1873  0.629  99       1923 -0.719 108       1973  0.731 230
1774  0.180   1       1824 -0.031  75       1874  0.233  99       1924 -0.444 108       1974  0.060 230
1775 -5.876   1       1825  0.438  77       1875  1.296  99       1925  0.437 109       1975  0.769 233
1776  0.781   2       1826  0.464  78       1876  0.598  99       1926  0.343 109       1976 -2.219 234
1777  1.268   4       1827  0.508  79       1877 -2.887  99       1927  0.703 111       1977 -0.875 234
1778  0.277   4       1828  1.171  79       1878  0.222 100       1928 -0.002 112       1978 -0.056 234
1779 -1.005   5       1829  0.965  80       1879  0.716 100       1929  0.532 112       1979 -0.396 234
1780 -0.932   6       1830  0.634  80       1880 -0.292 100       1930  0.128 112       1980 -1.253 234
1781 -0.295   6       1831 -0.868  80       1881 -0.551 101       1931  1.525 112       1981 -0.174 234
1782  0.156   6       1832 -0.489  80       1882  0.555 101       1932  0.431 112       1982  0.995 234
1783  0.764   7       1833  0.317  83       1883  0.631 101       1933  0.779 115       1983  0.440 235
1784  0.370   7       1834  1.337  84       1884  0.614 101       1934 -0.372 116       1984  0.642 235
1785  0.587   8       1835 -0.073  85       1885 -0.294 101       1935  0.023 116       1985 -0.224 235
1786  0.244  10       1836  0.275  85       1886  0.779 101       1936  0.437 116       1986 -1.860 235
1787  0.436  10       1837  1.216  85       1887  0.713 101       1937 -0.131 117       1987 -0.611 235
1788 -0.292  13       1838 -1.046  85       1888 -1.250 101       1938 -0.162 118       1988  0.945 235
1789 -0.502  13       1839  0.293  85       1889 -0.792 101       1939  0.449 118       1989  0.642 235
1790  0.657  13       1840  0.085  85       1890 -0.616 101       1940 -0.099 120       1990 -0.127 235
1791 -0.184  13       1841 -2.598  87       1891 -0.687 101       1941  0.201 124       1991  0.023 236
1792  0.379  17       1842  0.038  87       1892 -0.236 101       1942 -0.226 125       1992  0.643 236
1793 -0.155  18       1843 -1.152  88       1893 -1.705 101       1943  1.315 126       1993  1.021 235
1794 -0.474  20       1844 -0.445  88       1894  0.413 101       1944 -2.310 126       1994  1.672 235
1795 -0.738  23       1845 -0.217  89       1895  1.631 101       1945 -0.403 127       1995 -0.343 235
1796  1.218  25       1846  0.181  91       1896 -0.780 101       1946 -0.165 129       1996 -1.793 234
1797  1.024  29       1847 -1.154  92       1897  0.534 101       1947  0.206 129       1997 -0.024 234
1798  0.833  34       1848  0.883  93       1898  0.829 101       1948 -2.892 130       1998  0.092 233
1799 -1.313  36       1849  0.903  94       1899  0.892 102       1949 -0.341 130       1999  1.152 233
-------------------------------------------------------------------------------------------------------------------------------------
PART 4:  Master Bar Plot:                                                                00:00   00     0000  Page   8
-------------------------------------------------------------------------------------------------------------------------------------
```

```
Year Rel value  Year Rel value  Year Rel value  Year Rel value  Year Rel value  Year Rel value  Year Rel value  Year Rel value
1800----@       1850------B     1900f           1950---------D  2000--b
1801-------B    1851----------F 1901----@       1951---------D  2001----@
1802q           1852-----A      1902-----A      1952--------C   2002---a
1803--a         1853---------C  1903-----A      1953----------E 2003---------C
1804----------E 1854----@       1904-------B    1954-------B    2004-c
1805------A     1855--------C   1905----------D 1955----------E 2005---a
1806-----@      1856------A     1906---------C  1956--b         2006---a
1807----------D 1857-e          1907----------E 1957--b         2007-c
1808----------D 1858j           1908-----A      1958-----A      2008--------C
1809--------C   1859------B     1909j           1959-c          2009---------C
1810-------B    1860---------C  1910g           1960f           2010-------B
1811----------D 1861----------E 1911-------B    1961----@       2011-c
1812---a        1862-----A      1912----@       1962--------C   2012-----A
1813--------C   1863----------E 1913-----A      1963---a        2013--a
1814---a        1864----@       1914---------C  1964------A     2014-------B
1815o           1865j           1915----@       1965-------B    2015--------C
1816-e          1866-d          1916---------C  1966-------B    2016-e
1817---------C  1867-------B    1917----@       1967-------B    2017-c
1818-----@      1868-d          1918-e          1968------B     2018-c
1819----@       1869---a        1919-d          1969-----A      2019---------D
1820-d          1870----@       1920------A     1970---a        2020------B
1821------A     1871---------C  1921--b         1971------B     2021--------B
1822---a        1872---------D  1922-c          1972---a        2022-----@
1823-e          1873--------C   1923-c          1973--------C
1774-----A      1824----@       1874-----A      1924--b         1974----@
1775x           1825------B     1875----------E 1925------B     1975--------C
1776--------C   1826-------B    1876-------B    1926------A     1976i
1777----------E 1827-------B    1877l           1927--------C   1977-c
1778-----A      1828----------E 1878-----A      1928----@       1978----@
1779-d          1829---------D  1879--------C   1929-------B    1979--b
1780-d          1830--------C   1880---a        1930-----A      1980-e
1781---a        1831-c          1881--b         1931----------F 1981---a
1782-----A      1832--b         1882-------B    1932------B     1982---------D
1783--------C   1833------A     1883--------C   1933--------C   1983------B
1784------A     1834----------E 1884-------B    1934--a         1984--------C
1785-------B    1835----@       1885---a        1935----@       1985---a
1786-----A      1836-----A      1886--------C   1936------B     1986g
1787------B     1837----------E 1887--------C   1937---a        1987--b
1788---a        1838-d          1888-e          1938---a        1988---------D
1789--b         1839------A     1889-c          1939------B     1989--------C
1790--------C   1840-----@      1890--b         1940----@       1990---a
1791---a        1841j           1891-c          1941-----A      1991----@
1792------B     1842----@       1892---a        1942---a        1992--------C
1793---a        1843-e          1893g           1943----------E 1993---------D
1794--b         1844--b         1894------B     1944i           1994----------G
1795-c          1845---a        1895----------G 1945--b         1995--a
1796----------E 1846-----A      1896-c          1946---a        1996g
1797---------D  1847-e          1897-------B    1947-----A      1997----@
1798---------C  1848---------D  1898---------C  1948l           1998-----@
1799-e          1849---------D  1899---------D  1949--a         1999----------E
```

PART 5: CORRELATION OF SERIES BY SEGMENTS: 00:00 00 0000 Page 5

Correlations of 50-year dated segments, lagged 25 years

Flags: A = correlation under 0.3281 but highest as dated; B = correlation higher at other than dated position

Seq	Series	Time span	1775 1824	1800 1849	1825 1874	1850 1899	1875 1924	1900 1949	1925 1974	1950 1999	1975 2024
1	L10011	1804 2005		.59	.69	.74	.70	.50	.43	.48	.39
2	L10012	1797 1807	.74								
3	L10012	1810 2013		.63	.65	.78	.74	.57	.52	.69	.64
4	L10021	1818 2018		.50	.52	.63	.56	.68	.72	.71	.65
5	L10022	1826 2019			.59	.50	.41	.68	.72	.29A	.29A
6	L10061	1799 2019	.40	.37	.43	.32B	.47	.69	.65	.60	.58
7	L10062	1812 2019		.32A	.35	.74	.81	.64	.57	.59	.64
8	L10071	1822 2018		.58	.56	.60	.55	.77	.77	.63	.58
9	L10072	1803 2018		.26B	.34	.72	.73	.72	.56	.20B	.15B
10	L10081	1846 2020			.73	.71	.71	.65	.52	.53	.57
11	L10082	1808 2020		.59	.64	.77	.82	.69	.56	.40	.15B
12	L10101	1825 2020			.62	.48	.40	.48	.45	.59	.40
13	L10102	1814 2020		.64	.69	.70	.59	.62	.63	.59	.32B
14	L10131	1804 2018		.59	.52	.65	.66	.69	.56	.63	.63
15	L10132	1881 2018					.75	.77	.75	.78	.76
16	L10141	1809 2018		.58	.36	.58	.67	.69	.68	.65	.70
17	L10142	1822 2017		.41	.41	.59	.70	.48	.42	.76	.66
18	L10151	1796 2019	.71	.72	.61	.79	.79	.58	.46	.75	.70
19	L10152	1788 2019	.64	.69	.67	.82	.73	.71	.66	.63	.68
20	L10171	1847 2018			.83	.82	.76	.61	.44B	.56	.72
21	L10172	1834 2018			.60	.54	.48	.54	.51	.76	.81
22	L10181	1916 2019						.70	.69	.65	.69
23	L10182	1800 2019		.60	.60	.75	.78	.71	.77	.73	.73
24	L10183	1818 2019		.59	.64	.78	.82	.71	.67	.67	.71
25	L10331	1808 2018		.63	.76	.81	.79	.77	.70	.63	.65
26	L10332	1975 2018									.69
27	L7221	1800 2001		.54	.58	.72	.76	.71	.67	.81	.82
28	L7222	1794 1997	.76	.76	.68	.76	.76	.61	.58	.72	
29	L7231	1792 1995	.71	.73	.62	.52	.33	.18B	.23A	.40	
30	L7271	1916 2003						.63	.58	.33B	.18B
31	L7272	1815 1992		.05B	.09B	.18B	.49	.70	.75	.65	
32	L7273	1798 2007	.23B	.33B	.10B	.10B	.49	.74	.76	.48	.26A
33	L7301	1809 2016		.68	.75	.67	.43	.42	.61	.66	.64
34	L7302	1799 2012	.72	.71	.80	.76	.53	.43	.54	.76	.76

35	SD1330	1792	2021	.45	.44	.59	.48	.53	.64	.59	.59	.65
36	SD1331	1804	2021		.43	.63	.52	.50	.52	.53	.57	.68
37	SD1401	1870	2021				.35	.36	.57	.63	.63	.50
38	SD1402	1845	2021			.59	.61	.56	.46	.52	.62	.66
39	SD1621	1786	2021	.44	.58	.55	.55	.67	.65	.56	.67	.67
40	SD1622	1786	2021	.40	.55	.44	.31A	.48	.51	.54	.65	.62
41	SD1650	1804	2021		.66	.69	.66	.50	.51	.60	.62	.67
42	SD1651	1798	2021	.76	.74	.76	.67	.64	.73	.78	.76	.76
43	SD1720	1937	2021							.73	.63	.59
44	SD1721	1934	2021							.81	.74	.71
45	SD1750	1792	2021	.58	.62	.70	.66	.62	.66	.77	.79	.72
46	SD1751	1813	2021		.54	.66	.75	.73	.69	.69	.71	.62
47	SD1752	1899	2021					.61	.60	.65	.72	.63
48	SD1753	1806	2021		.58	.58	.42	.47	.60	.61	.74	.63
49	SD1754	1805	2021		.65	.71	.71	.60	.56	.59	.71	.76
50	SD1755	1783	2021	.56	.67	.76	.74	.70	.57	.40	.42	.58
51	SD1920	1841	2021			.64	.69	.79	.69	.63	.77	.74
52	SD1921	1798	2021	.69	.69	.61	.76	.70	.51	.43B	.72	.75
53	SD1930	1835	2021			.71	.73	.63	.62	.50	.48	.55
54	SD1931	1798	2021	.63	.66	.60	.78	.71	.52	.57	.82	.72
55	SD1970	1774	2021	.58	.72	.56	.61	.68	.73	.68	.73	.73
56	SD1971	1788	2021	.60	.66	.55	.23B	.14B	.53	.62	.79	.75
57	SD1990	1800	2021		.46	.36	.41	.42	.63	.56	.59	.53
58	SD1991	1802	2021		.50	.44	.41	.47	.75	.76	.72	.60
59	SD2210	1796	2021	.53	.55	.58	.69	.80	.65	.60	.71	.65
60	SD2211	1800	2018		.54	.69	.75	.76	.59	.50	.66	.42
61	SD2230	1776	2021	.55	.71	.45	.60	.57	.56	.72	.70	.55
62	SD2231	1798	2021	.64	.68	.45	.63	.53	.53	.67	.69	.65
63	SD2240	1795	2021	.80	.77	.58	.60	.43	.61	.75	.51	.38
64	SD2241	1779	2021	.68	.79	.62	.71	.52	.36B	.51	.45	.49
65	SD2311	1777	2021	.63	.68	.74	.83	.83	.75	.65	.71	.72
66	SD2312	1794	2021	.73	.72	.75	.68	.67	.78	.73	.74	.68
67	SD2501	1825	2021			.62	.68	.57	.49	.62	.45	.30A
68	SD2502	1797	2021	.26A	.33	.72	.68	.55	.60	.60	.56	.53
69	SD2560	1802	2021		.68	.66	.68	.54	.60	.68	.69	.66
70	SD2561	1813	2021		.68	.68	.60	.70	.71	.52	.65	.69
71	SD2580	1804	2021		.55	.57	.73	.65	.46	.30A	.67	.78
72	SD2581	1792	2021	.27B	.30B	.56	.53	.45	.69	.70	.74	.71
73	SD2760	1793	2021	.42	.49	.74	.57	.25A	.31A	.32B	.54	.68
74	SD2761	1788	2021	.22B	.40	.48	.14B	.23B	.39	.37	.56	.72
75	SD2780	1816	2021		.77	.74	.62	.55	.72	.79	.55	.40
76	SD2781	1833	2014			.62	.63	.58	.73	.81	.72	.66
77	SD2791	1804	2021		.66	.60	.40	.38	.61	.59	.62	.52
78	SD2792	1833	2021			.66	.52	.58	.69	.59	.62	.61
79	SD2801	1824	2021		.58	.54	.46	.40	.67	.69	.64	.77
80	SD2802	1823	2021		.52	.55	.43	.54	.41	.07B	.37	.64
81	SD2840	1827	2021			.73	.68	.50	.37	.52	.79	.74
82	SD2841	1822	2021		.73	.77	.79	.69	.67	.71	.77	.71
83	SD2851	1795	2021	.68	.67	.57	.48	.56	.69	.67	.62	.66
84	SD2852	1815	2021		.54	.59	.58	.64	.72	.67	.53	.41
85	SD2930	1833	2021			.54	.69	.69	.55	.54	.75	.78
86	SD2931	1805	2021		.59	.61	.59	.57	.58	.58	.64	.68
87	SD3771	1818	2021		-.20B	-.17B	-.05B	-.06B	.29B	.67	.55	.63
88	SD3772	1785	2021	.37B	.16B	-.03B	.05B	.05B	.31A	.35	.29A	.38
89	SD4361	1846	2021			.64	.67	.57	.50	.60	.71	.77
90	SD4362	1878	2021					.33A	.42	.57	.72	.68
91	SD4521	1813	2021		.62	.62	.71	.82	.75	.60	.61	.48
92	SD4522	1797	2021	.60	.60	.64	.70	.72	.66	.60	.53	.59
93	SD4531	1829	2021			.00B	.13B	.36	.59	.62	.45	.56
94	SD4532	1871	2021				.41	.44	.38B	.40	.66	.80
95	SD4551	1819	2017		.73	.73	.68	.74	.76	.72	.74	.71
96	SD4581	1797	2017	.31B	.33B	.57	.62	.41	-.03B	.07B	.46	.63
97	SD5341	1803	2017		.28A	.17B	.33	.45	.65	.54	.63	.70
98	SD6311	1856	2016				.68	.62	.74	.80	.64	.66
99	SD6681	1851	2021				.86	.85	.77	.68	.72	.75
100	SD6682	1848	2021			.71	.71	.71	.72	.67	.68	.69
101	SD7221	1795	2021	.68	.65	.59	.53	.23A	.25A	.19B	.46	.53
102	SD7222	1780	2021	.76	.70	.54	.65	.58	.60	.64	.62	.63
103	SD81	1873	2021				.59	.59	.80	.78	.63	.60
104	SD82	1841	2021			.32B	.38	.53	.72	.71	.66	.67
105	SD83	1843	2017			.38	.52	.58	.61	.53	.68	.72
106	SD8601	1777	2017	.68	.65	.31B	.10B	.03B	.07B	.12B	.31A	.38
107	SD9531	1811	2021		.30A	.46	.48	.26B	.41	.63	.72	.68
108	SD9532	1849	2021			.68	.66	.47	.37	.54	.66	.65
109	NG1070	1941	2021							.47	.48	.61
110	NG1071	1941	2021							.58	.42	.53
111	NG1110	1956	2021								.37	.35
112	NG1111	1965	2021								-.03B	-.01B
113	NG1120	1952	2021								.37	.31A
114	NG1121	1943	2021							.41	.26A	.25A
115	NG1180	1961	2021								.24B	.20B
116	NG1181	1963	2021								.25A	.17B
117	NG1182	1963	2021								.27B	.28A
118	NG1183	1968	2021								.34	.33A
119	NG1190	1964	2021								.49	.57
120	NG1191	1957	2021								.42	.49
121	NG1280	1971	2021								.32B	.31B
122	NG1281	1972	2021								.26B	
123	NG1282	1962	2022								.27A	.27A
124	NG1283	1976	2022									.33
125	NG1290	1961	2022								.52	.69
126	NG1291	1965	2022								.49	.56

127	NG1320	1964	2021			.53	.63
128	NG1321	1966	2021			.36	.23B
129	NG1400	1968	2022			.18B	.17B
130	NG1401	1967	2022			.36B	.38
131	NG1420	1968	2022			.32A	.30A
132	NG1421	1969	2022			.49	.50
133	NG1422	1969	2022			.41	.45
134	NG1423	1971	2022			.37	.37
135	NG1430	1968	2022			.49	.54
136	NG1431	1958	2022			.28A	.47
137	NG1440	1959	2022			.33B	.24B
138	NG1441	1968	2022			.32A	.32A
139	NG1470	1975	2022				.16B
140	NG1471	1966	2022			.24B	.28A
141	NG151	1968	2017			.20B	
142	NG1580	1962	2021			.16B	.27A
143	NG1581	1967	2021			.21B	.20B
144	NG2640	1958	2021			.45	.36
145	NG2641	1959	2021			.45	.42
146	NG2650	1952	2021			.36	.25B
147	NG2651	1962	2021			.56	.41
148	NG2710	1968	2021			.37	.34
149	NG2711	1967	2021			.43	.41
150	NG2712	1970	2021			.36	.34
151	NG2713	1965	2021			.43	.49
152	NG2800	1967	2021			.33	.34B
153	NG2801	1958	2021			.41	.34B
154	NG2900	1948	2021		.13B	.28B	.35B
155	NG2901	1960	2021			.35	.40
156	NG2940	1957	2021			.21B	.24A
157	NG2941	1957	2020			.38	.28A
158	NG3020	1955	2021			.50	.46
159	NG3021	1951	2021			.34	.41
160	NG3040	1960	2021			.40	.19A
161	NG3041	1972	2021			.17B	
162	NG3051	1960	2021			.41	.42
163	NG3052	1953	2021			.39	.40
164	NG3101	1951	2021			.44	.25B
165	NG3102	1956	2021			.43	.43
166	NG3571	1983	2021				.37
167	NG3572	1991	2021				.27A
168	NG3581	1970	2021			.40	.39
169	NG3582	1975	2021				.19B
170	NG4141	1946	2020		.65	.51	.39B
171	NG4142	1950	2021			.48	.37
172	WG11	1964	2022			.50	.53
173	WG12	1968	2022			.36	.43
174	WG101	1964	2022			.33	.41
175	WG102	1961	2022			.21A	.30A
176	WG111	1928	2022		.40	.43	.45
177	WG112	1933	2022		.15B	.38	.47
178	WG121	1962	2022			.35B	.38
179	WG122	1973	2022			.42	
180	WG131	1966	2022			.43	.45
181	WG132	1959	2022			.38B	.44
182	WG141	1971	2022			.24A	.18B
183	WG142	1963	2022			.28B	.23B
184	WG151	1959	2022			.42	.40
185	WG152	1961	2022			.35B	.38
186	WG161	1971	2022			.20B	.20B
187	WG162	1963	2022			.45	.45
188	WG171	1957	2022			.36B	.44
189	WG172	1963	2022			.38	.46
190	WG181	1927	2022		.59	.54	.74
191	WG182	1921	2022	.26B	.27B	.32A	.68
192	WG191	1964	2022			.30B	.36B
193	WG192	1967	2022			.36	.38
194	WG21	1959	2022			.16B	.13B
195	WG22	1959	2022			.45	.36B
196	WG201	1972	2022			.53	.53
197	WG202	1960	2022			.31A	.44
198	WG211	1957	2022			.12B	.10B
199	WG212	1957	2022			.45	.36
200	WG221	1927	2022		.27A	.44	.59
201	WG222	1946	2022		.41	.51	.63
202	WG231	1961	2022			.58	.67
203	WG232	1962	2022			.64	.56
204	WG241	1916	2022	.16B	.21A	.31A	.55
205	WG242	1914	2022	.16B	.18B	.50	.62
206	WG251	1954	2022			.34B	.43
207	WG252	1938	2022		.25A	.20B	.44
208	WG261	1962	2022			.08B	.20B
209	WG262	1964	2022			.29B	.24B
210	WG271	1953	2022			.29B	.31A
211	WG272	1954	2022			.30B	.49
212	WG281	1960	2022			.55	.52
213	WG282	1964	2022			.35	.28A
214	WG291	1920	2022	.33B	.35	.41	.57
215	WG292	1933	2022		.36	.38	.55
216	WG31	1967	2022			.45	.47
217	WG32	1961	2022			.33	.38
218	WG301	1969	2022			.34	.32A

```
219 WG302    1960 2022                                      .32A .55
220 WG311    1964 2022                                      .38  .32A
221 WG312    1963 2022                                      .48  .35
222 WG321    1967 2022                                      .28A .28A
223 WG322    1959 2022                                      .05B .21A
224 WG331    1942 2022                                -.02B .35  .48
225 WG332    1945 2022                                 .36  .36  .38B
226 WG41     1963 2022                                      .34B .35
227 WG42     1961 2022                                      .37  .35
228 WG51     1958 2022                                      .49  .48
229 WG52     1965 2022                                      .34  .28A
230 WG61     1925 2022                                 .48  .54  .75
231 WG62     1933 2022                                 .41  .49  .57
232 WG71     1940 2022                                 .30B .19B .36B
233 WG72     1941 2022                                 .34  .25A .29A
234 WG81     1963 2022                                      .48  .37
235 WG82     1958 2022                                      .30A .30A
236 WG91     1940 2022                                 .16B .20B .40
237 WG92     1941 2022                                 .32A .37  .50
Av segment correlation  0.57 0.56 0.57 0.59 0.57 0.57 0.54 0.48 0.49
PART 6:  POTENTIAL PROBLEMS:                                                                       00:00   00     0000  Page   9
------------------------------------------------------------------------------------------------------------------------------------
For each series with potential problems the following diagnostics may appear:
[A] Correlations with master dating series of flagged  50-year segments of series filtered with  32-year spline,
at every point from ten years earlier (-10) to ten years later (+10) than dated
[B] Effect of those data values which most lower or raise correlation with master series
Symbol following year indicates value in series is greater (>) or lesser (<) than master series value
[C] Year-to-year changes very different from the mean change in other series
[D] Absent rings (zero values)
[E] Values which are statistical outliers from mean for the year
====================================================================================================================================
L10011    1804 to  2005     202 years                                                                                  Series   1
[B] Entire series, effect on correlation ( 0.565) is:
Lower   1994<-0.020   1843<-0.019   1823>-0.011   1944>-0.010   1980>-0.009   1887<-0.007  Higher   1976 0.019   1877 0.013
[E] Outliers     1   3.0 SD above or -4.5 SD below mean for year
1844 -5.0 SD
====================================================================================================================================
L10012    1797 to  1807      11 years                                                                                  Series   2
[B] Entire series, effect on correlation ( 0.740) is:
Lower   1803<-0.056   1807<-0.044   1805>-0.036   1806>-0.035   1804<-0.009   1798>-0.009  Higher   1802 0.331   1799 0.041
====================================================================================================================================
L10012    1810 to  2013     204 years                                                                                  Series   3
[B] Entire series, effect on correlation ( 0.650) is:
Lower   1919<-0.011   1843<-0.010   1824<-0.009   1980>-0.008   1820>-0.007   1961<-0.007  Higher   1815 0.023   1976 0.014
[E] Outliers     1   3.0 SD above or -4.5 SD below mean for year
1919 -4.8 SD
====================================================================================================================================
L10021    1818 to  2018     201 years                                                                                  Series   4
[B] Entire series, effect on correlation ( 0.595) is:
Lower   1841>-0.028   1899<-0.023   1984<-0.010   2007>-0.009   1843>-0.009   1893>-0.009  Higher   1877 0.028   1976 0.022
[E] Outliers     1   3.0 SD above or -4.5 SD below mean for year
1899 -4.7 SD
====================================================================================================================================
L10022    1826 to  2019     194 years                                                                                  Series   5
[A] Segment   High   -10   -9   -8   -7   -6   -5   -4   -3   -2   -1    0   +1   +2   +3   +4   +5   +6   +7   +8   +9  +10
---------  ----   ---  ---  ---  ---  ---  ---  ---  ---  ---  ---  ---  ---  ---  ---  ---  ---  ---  ---  ---  ---  ---
1950 1999    0    .27 -.09  .08 -.11 -.05  .06 -.07 -.10 -.22 -.01  .29* .22  .20 -.11 -.06  .07  .00 -.26 -.26  .00 -.03
1970 2019    0    .22  .06  .13 -.04 -.08  .25  .11 -.12 -.27 -.15  .29* .13  .16 -.31    -    -    -    -    -    -    -
[B] Entire series, effect on correlation ( 0.483) is:
Lower   1984<-0.055   1975<-0.018   1841>-0.015   1980>-0.011   1893>-0.010   2007>-0.010  Higher   1948 0.046   1944 0.020
1950 to 1999 segment:
Lower   1984<-0.181   1975<-0.055   1980>-0.044   1985>-0.016   1977>-0.011   1958<-0.008  Higher   1976 0.079   1986 0.043
1970 to 2019 segment:
Lower   1984<-0.160   1975<-0.053   1980>-0.037   2007>-0.030   2009<-0.016   2013>-0.014  Higher   1976 0.060   2016 0.041
====================================================================================================================================
L10061    1799 to  2019     221 years                                                                                  Series   6
[A] Segment   High   -10   -9   -8   -7   -6   -5   -4   -3   -2   -1    0   +1   +2   +3   +4   +5   +6   +7   +8   +9  +10
---------  ----   ---  ---  ---  ---  ---  ---  ---  ---  ---  ---  ---  ---  ---  ---  ---  ---  ---  ---  ---  ---  ---
1850 1899    8   -.13 -.05  .02  .04 -.16  .15  .33  .07 -.33  .08  .32|-.19 -.23 -.01 -.02 -.04 -.14  .21  .38*-.18 -.24
[B] Entire series, effect on correlation ( 0.493) is:
Lower   1802>-0.031   1877>-0.019   1825<-0.012   1823>-0.011   1888>-0.009   1988<-0.008  Higher   1815 0.021   1944 0.017
1850 to 1899 segment:
Lower   1877>-0.076   1888>-0.040   1868>-0.027   1897<-0.018   1872<-0.018   1882<-0.014  Higher   1857 0.033   1893 0.027
[E] Outliers     1   3.0 SD above or -4.5 SD below mean for year
1802 +3.1 SD
====================================================================================================================================
L10062    1812 to  2019     208 years                                                                                  Series   7
[A] Segment   High   -10   -9   -8   -7   -6   -5   -4   -3   -2   -1    0   +1   +2   +3   +4   +5   +6   +7   +8   +9  +10
---------  ----   ---  ---  ---  ---  ---  ---  ---  ---  ---  ---  ---  ---  ---  ---  ---  ---  ---  ---  ---  ---  ---
1812 1861    0    .11  .12 -.17 -.04  .11  .07 -.13  .08  .07 -.15  .32*-.07 -.22 -.18 -.23 -.01  .16  .03  .01  .01  .15
[B] Entire series, effect on correlation ( 0.559) is:
Lower   1825<-0.046   1925<-0.023   1826<-0.013   1996>-0.012   1841>-0.009   1823>-0.007  Higher   1877 0.023   1944 0.020
1812 to 1861 segment:
Lower   1825<-0.142   1826<-0.036   1823>-0.020   1848<-0.017   1841>-0.016   1859<-0.011  Higher   1847 0.039   1858 0.034
[C] Year-to-year changes diverging by over 4.0 std deviations:
1824 1825  -4.4 SD
[E] Outliers     2   3.0 SD above or -4.5 SD below mean for year
1825 -5.5 SD;    1826 -5.6 SD
====================================================================================================================================
L10071    1822 to  2018     197 years                                                                                  Series   8
[B] Entire series, effect on correlation ( 0.615) is:
Lower   1847<-0.021   1841>-0.013   1918>-0.009   1923<-0.009   1875<-0.008   2018>-0.007  Higher   1944 0.027   1948 0.018
```

```
[E] Outliers     1   3.0 SD above or -4.5 SD below mean for year
1847 -5.8 SD
====================================================================================================================================
L10072    1803 to  2018     216 years                                                                                    Series   9
[A] Segment   High   -10   -9   -8   -7   -6   -5   -4   -3   -2   -1    0   +1   +2   +3   +4   +5   +6   +7   +8   +9  +10
---------  ----   ---  ---  ---  ---  ---  ---  ---  ---  ---  ---  ---  ---  ---  ---  ---  ---  ---  ---  ---  ---  ---
1803 1852    3    .19 -.25 -.04 -.02 -.20 -.19  .12  .25 -.14  .05  .26|-.05  .05  .26* .08 -.03 -.09 -.11  .10  .05 -.15
- - - - - - - - - - - - - - - - - - - - - - - - - - - - - - - - - - - - - - - - - - - - - - - - - - - - - - - - - - - - - -
1950 1999    4    .13 -.12  .04 -.14  .00 -.12 -.15 -.04  .05  .06  .20| .00 -.11  .11  .40*-.13  .19 -.06  .16 -.19  .04
1969 2018    4    .07  .02 -.36 -.09 -.04  .06 -.13  .18  .07  .05  .15|-.01 -.08  .05  .22*   -    -    -    -    -    -
[B] Entire series, effect on correlation ( 0.482) is:
Lower   1847>-0.024   1841>-0.015   1815>-0.014   1960>-0.014   1859<-0.010   1888>-0.010  Higher   1877 0.042   1944 0.026
1803 to 1852 segment:
Lower   1847>-0.100   1841>-0.043   1826<-0.033   1815>-0.029   1831>-0.019   1820>-0.014  Higher   1843 0.038   1823 0.032
1950 to 1999 segment:
Lower   1960>-0.069   1955<-0.037   1992<-0.024   1995>-0.024   1973<-0.021   1999<-0.019  Higher   1994 0.051   1956 0.050
1969 to 2018 segment:
Lower   2009<-0.033   1992<-0.031   1973<-0.026   1995>-0.022   1999<-0.022   2018>-0.018  Higher   1994 0.068   2008 0.052
[E] Outliers     1   3.0 SD above or -4.5 SD below mean for year
1847 +3.6 SD
====================================================================================================================================
L10081    1846 to  2020     175 years                                                                                    Series  10
[B] Entire series, effect on correlation ( 0.629) is:
Lower   1992<-0.012   1859<-0.011   1896>-0.010   1885<-0.009   1947<-0.009   1959>-0.009  Higher   1877 0.035   1944 0.013
====================================================================================================================================
L10082    1808 to  2020     213 years                                                                                    Series  11
[A] Segment   High   -10   -9   -8   -7   -6   -5   -4   -3   -2   -1    0   +1   +2   +3   +4   +5   +6   +7   +8   +9  +10
---------  ----   ---  ---  ---  ---  ---  ---  ---  ---  ---  ---  ---  ---  ---  ---  ---  ---  ---  ---  ---  ---  ---
1971 2020    1   -.01  .03 -.07 -.02 -.17  .11  .10 -.22  .05  .11  .15| .17* .02    -    -    -    -    -    -    -    -
[B] Entire series, effect on correlation ( 0.549) is:
Lower   2007>-0.021   1995>-0.013   1986>-0.010   1996>-0.010   1997<-0.009   2013>-0.008  Higher   1877 0.033   1976 0.020
1971 to 2020 segment:
Lower   2007>-0.067   1995>-0.045   1986>-0.033   1996>-0.032   1997<-0.028   2004>-0.025  Higher   1976 0.195   2019 0.033
[E] Outliers     1   3.0 SD above or -4.5 SD below mean for year
2007 +3.2 SD
====================================================================================================================================
L10101    1825 to  2020     196 years                                                                                    Series  12
[B] Entire series, effect on correlation ( 0.473) is:
Lower   1900>-0.021   1897<-0.020   2016>-0.020   2019<-0.011   1898<-0.011   1956>-0.010  Higher   1841 0.024   1877 0.024
[C] Year-to-year changes diverging by over 4.0 std deviations:
1899 1900   5.0 SD
[E] Outliers     1   3.0 SD above or -4.5 SD below mean for year
1831 -5.1 SD
====================================================================================================================================
L10102    1814 to  2020     207 years                                                                                    Series  13
[A] Segment   High   -10   -9   -8   -7   -6   -5   -4   -3   -2   -1    0   +1   +2   +3   +4   +5   +6   +7   +8   +9  +10
---------  ----   ---  ---  ---  ---  ---  ---  ---  ---  ---  ---  ---  ---  ---  ---  ---  ---  ---  ---  ---  ---  ---
1971 2020  -10    .36*-.05 -.12 -.14  .07  .00 -.14 -.05 -.09  .14  .32| .12 -.14    -    -    -    -    -    -    -    -
[B] Entire series, effect on correlation ( 0.557) is:
Lower   2016>-0.017   1847<-0.016   2019<-0.012   2011>-0.009   1871<-0.009   1956>-0.008  Higher   1841 0.022   1944 0.021
1971 to 2020 segment:
Lower   2016>-0.074   2019<-0.057   2011>-0.038   2012<-0.027   2010<-0.026   2003<-0.020  Higher   1986 0.057   1976 0.051
[E] Outliers     1   3.0 SD above or -4.5 SD below mean for year
1847 -5.2 SD
====================================================================================================================================
L10131    1804 to  2018     215 years                                                                                    Series  14
[B] Entire series, effect on correlation ( 0.634) is:
Lower   1834<-0.013   1969<-0.010   1848<-0.008   1838>-0.006   1946<-0.005   1996>-0.004  Higher   1815 0.021   1976 0.019
====================================================================================================================================
L10132    1881 to  2018     138 years                                                                                    Series  15
[B] Entire series, effect on correlation ( 0.753) is:
Lower   1980>-0.012   1884<-0.007   1905<-0.006   1956>-0.006   1932>-0.005   1923>-0.005  Higher   1976 0.020   1909 0.018
====================================================================================================================================
L10141    1809 to  2018     210 years                                                                                    Series  16
[B] Entire series, effect on correlation ( 0.612) is:
Lower   1841>-0.022   1963<-0.013   1847>-0.009   1980>-0.007   1871<-0.007   2007>-0.006  Higher   1944 0.028   1976 0.015
====================================================================================================================================
L10142    1822 to  2017     196 years                                                                                    Series  17
[B] Entire series, effect on correlation ( 0.540) is:
Lower   1936<-0.034   1825<-0.019   1841>-0.016   2009<-0.014   1944>-0.010   2007>-0.007  Higher   1976 0.030   1865 0.013
[E] Outliers     1   3.0 SD above or -4.5 SD below mean for year
1870 -4.8 SD
====================================================================================================================================
L10151    1796 to  2019     224 years                                                                                    Series  18
[B] Entire series, effect on correlation ( 0.668) is:
Lower   1799>-0.010   1848<-0.008   1948>-0.006   2000>-0.006   1840>-0.006   1822>-0.006  Higher   1802 0.044   1976 0.013
====================================================================================================================================
L10152    1788 to  2019     232 years                                                                                    Series  19
[B] Entire series, effect on correlation ( 0.672) is:
Lower   1950<-0.010   1843>-0.008   1799>-0.007   2018<-0.006   1788>-0.006   1980>-0.006  Higher   1802 0.024   1944 0.017
====================================================================================================================================
L10171    1847 to  2018     172 years                                                                                    Series  20
[A] Segment   High   -10   -9   -8   -7   -6   -5   -4   -3   -2   -1    0   +1   +2   +3   +4   +5   +6   +7   +8   +9  +10
---------  ----   ---  ---  ---  ---  ---  ---  ---  ---  ---  ---  ---  ---  ---  ---  ---  ---  ---  ---  ---  ---  ---
1925 1974   10    .21 -.06 -.19 -.16  .04 -.20 -.04 -.07  .07 -.03  .44|-.16  .20 -.04  .11 -.32  .11 -.19 -.06 -.17  .44*
[B] Entire series, effect on correlation ( 0.674) is:
Lower   1966<-0.046   1944>-0.012   2007>-0.007   1958<-0.005   1920<-0.005   2013>-0.005  Higher   1877 0.029   1858 0.007
1925 to 1974 segment:
Lower   1966<-0.179   1958<-0.016   1944>-0.011   1972>-0.007   1938<-0.005   1969>-0.004  Higher   1943 0.036   1948 0.036
====================================================================================================================================
L10172    1834 to  2018     185 years                                                                                    Series  21
[B] Entire series, effect on correlation ( 0.632) is:
```

```
Lower   1882<-0.047   1944>-0.023   1966<-0.015   2007>-0.009   1909>-0.008   1933<-0.008  Higher   1948 0.021   1976 0.020
[C] Year-to-year changes diverging by over 4.0 std deviations:
1881 1882  -4.0 SD
[E] Outliers     1   3.0 SD above or -4.5 SD below mean for year
1882 -5.0 SD
====================================================================================================================================
L10181    1916 to  2019     104 years                                                                                    Series  22
[B] Entire series, effect on correlation ( 0.682) is:
Lower   1961<-0.010   1928<-0.010   1985>-0.010   2007>-0.010   1931<-0.007   1944>-0.006  Higher   1948 0.058   1986 0.014
====================================================================================================================================
L10182    1800 to  2019     220 years                                                                                    Series  23
[B] Entire series, effect on correlation ( 0.690) is:
Lower   1829<-0.015   1815>-0.014   1919>-0.010   1900>-0.010   1871<-0.007   1847>-0.007  Higher   1877 0.018   1841 0.013
====================================================================================================================================
L10183    1818 to  2019     202 years                                                                                    Series  24
[B] Entire series, effect on correlation ( 0.699) is:
Lower   1858>-0.012   1829<-0.011   2007>-0.009   1933<-0.008   1967<-0.008   1823>-0.007  Higher   1877 0.021   1841 0.014
====================================================================================================================================
L10331    1808 to  2018     211 years                                                                                    Series  25
[B] Entire series, effect on correlation ( 0.706) is:
Lower   1823>-0.010   1847>-0.008   1828<-0.007   1980>-0.007   1959>-0.007   2007>-0.004  Higher   1877 0.022   1948 0.020
====================================================================================================================================
L10332    1975 to  2018      44 years                                                                                    Series  26
[B] Entire series, effect on correlation ( 0.693) is:
Lower   2007>-0.053   1980>-0.040   2017>-0.026   1985>-0.015   2016>-0.014   1990<-0.013  Higher   1976 0.098   1986 0.025
====================================================================================================================================
L7221     1800 to  2001     202 years                                                                                    Series  27
[B] Entire series, effect on correlation ( 0.671) is:
Lower   1823<-0.028   1815>-0.018   1948>-0.013   1841>-0.013   1869<-0.007   1896>-0.006  Higher   1802 0.027   1944 0.016
[C] Year-to-year changes diverging by over 4.0 std deviations:
1822 1823  -4.2 SD
[E] Outliers     1   3.0 SD above or -4.5 SD below mean for year
1823 -5.6 SD
====================================================================================================================================
L7222     1794 to  1997     204 years                                                                                    Series  28
[B] Entire series, effect on correlation ( 0.717) is:
Lower   1823<-0.015   1936<-0.013   1920<-0.007   1871<-0.005   1953<-0.005   1841>-0.005  Higher   1802 0.038   1877 0.018
[C] Year-to-year changes diverging by over 4.0 std deviations:
1823 1824   4.4 SD
[E] Outliers     1   3.0 SD above or -4.5 SD below mean for year
1823 -5.6 SD
====================================================================================================================================
L7231     1792 to  1995     204 years                                                                                    Series  29
[A] Segment   High   -10   -9   -8   -7   -6   -5   -4   -3   -2   -1    0   +1   +2   +3   +4   +5   +6   +7   +8   +9  +10
---------  ----   ---  ---  ---  ---  ---  ---  ---  ---  ---  ---  ---  ---  ---  ---  ---  ---  ---  ---  ---  ---  ---
1900 1949   -7   -.06 -.15  .08  .24* .21 -.01  .14  .02 -.14 -.09  .18| .04 -.01 -.25 -.14 -.02  .17  .16  .00  .06  .10
1925 1974    0    .07 -.16 -.03  .13 -.22 -.01 -.08  .15 -.05 -.07  .23* .03 -.02 -.30  .15 -.06  .16  .06  .12 -.03  .14
[B] Entire series, effect on correlation ( 0.506) is:
Lower   1877>-0.043   1916<-0.019   1944>-0.018   1976>-0.018   1948>-0.010   1799>-0.008  Higher   1802 0.079   1865 0.011
1900 to 1949 segment:
Lower   1916<-0.081   1944>-0.048   1949<-0.022   1919>-0.017   1918>-0.012   1925<-0.012  Higher   1909 0.061   1905 0.037
1925 to 1974 segment:
Lower   1944>-0.070   1960>-0.032   1948>-0.019   1949<-0.019   1955<-0.019   1951<-0.018  Higher   1956 0.032   1959 0.032
[C] Year-to-year changes diverging by over 4.0 std deviations:
1801 1802  -4.6 SD
[E] Outliers     1   3.0 SD above or -4.5 SD below mean for year
1877 +4.0 SD
====================================================================================================================================
L7271     1916 to  2003      88 years                                                                                    Series  30
[A] Segment   High   -10   -9   -8   -7   -6   -5   -4   -3   -2   -1    0   +1   +2   +3   +4   +5   +6   +7   +8   +9  +10
---------  ----   ---  ---  ---  ---  ---  ---  ---  ---  ---  ---  ---  ---  ---  ---  ---  ---  ---  ---  ---  ---  ---
1950 1999    6   -.08 -.07  .12 -.19 -.42 -.18  .33 -.04 -.15  .18  .33|-.02  .20 -.14 -.15 -.09  .34*-.25 -.06  .12  .13
1954 2003    6   -.09 -.06  .11 -.26 -.24 -.03  .21  .00 -.19  .26  .18| .02  .04 -.01 -.18 -.12  .40*-.30 -.05  .10  .24
[B] Entire series, effect on correlation ( 0.371) is:
Lower   1994<-0.055   1936<-0.055   1986>-0.036   2000>-0.033   2001<-0.031   1996>-0.012  Higher   1948 0.053   1944 0.043
1950 to 1999 segment:
Lower   1994<-0.118   1986>-0.075   1996>-0.022   1984<-0.014   1999<-0.013   1979>-0.013  Higher   1976 0.059   1960 0.054
1954 to 2003 segment:
Lower   1994<-0.093   1986>-0.059   2000>-0.053   2001<-0.040   1996>-0.016   1984<-0.011  Higher   1976 0.066   1960 0.054
[E] Outliers     1   3.0 SD above or -4.5 SD below mean for year
1936 -4.9 SD
====================================================================================================================================
L7272     1815 to  1992     178 years                                                                                    Series  31
[A] Segment   High   -10   -9   -8   -7   -6   -5   -4   -3   -2   -1    0   +1   +2   +3   +4   +5   +6   +7   +8   +9  +10
---------  ----   ---  ---  ---  ---  ---  ---  ---  ---  ---  ---  ---  ---  ---  ---  ---  ---  ---  ---  ---  ---  ---
1815 1864   -3   -.25  .20 -.07 -.10  .19 -.12  .07  .59*-.02 -.20  .05|-.13  .16  .08 -.21  .10 -.12 -.12 -.05 -.04 -.06
1825 1874   -3   -.05  .25 -.13 -.12  .29 -.17 -.04  .48* .10 -.07  .09|-.20  .15  .07 -.36 -.08  .01 -.12 -.16  .19  .08
1850 1899   -3   -.02  .05 -.01 -.29  .05 -.03 -.11  .43* .05  .13  .18|-.38  .08 -.04 -.20 -.04  .09 -.09  .08  .11  .04
[B] Entire series, effect on correlation ( 0.356) is:
Lower   1818<-0.024   1865>-0.017   1894<-0.016   1858>-0.014   1815>-0.013   1986>-0.013  Higher   1944 0.032   1948 0.024
1815 to 1864 segment:
Lower   1858>-0.029   1857>-0.027   1828<-0.020   1829<-0.018   1860<-0.016   1818<-0.013  Higher   1847 0.035   1838 0.030
1825 to 1874 segment:
Lower   1865>-0.047   1858>-0.038   1857>-0.036   1871<-0.025   1828<-0.023   1829<-0.021  Higher   1868 0.070   1847 0.044
1850 to 1899 segment:
Lower   1865>-0.052   1894<-0.045   1858>-0.042   1857>-0.038   1871<-0.025   1877>-0.022  Higher   1888 0.063   1868 0.053
[E] Outliers     1   3.0 SD above or -4.5 SD below mean for year
1818 -5.4 SD
====================================================================================================================================
L7273     1798 to  2007     210 years                                                                                    Series  32
[A] Segment   High   -10   -9   -8   -7   -6   -5   -4   -3   -2   -1    0   +1   +2   +3   +4   +5   +6   +7   +8   +9  +10
---------  ----   ---  ---  ---  ---  ---  ---  ---  ---  ---  ---  ---  ---  ---  ---  ---  ---  ---  ---  ---  ---  ---
```

```
1798 1847   -3    .00 -.03 -.04 -.13 -.03  .03 -.13  .38*-.16 -.07  .23| .17 -.02  .09  .13  .04 -.09 -.04 -.08 -.25 -.03
1800 1849   -3   -.05 -.05 -.04 -.18 -.02  .01 -.15  .37*-.13 -.04  .33| .10  .01  .20 -.10  .06 -.01 -.03 -.11 -.27 -.01
1825 1874   -3    .02  .09 -.02 -.01  .10  .09 -.05  .57*-.06 -.15  .10| .13  .00 -.08 -.16  .05 -.14 -.19  .03  .01 -.04
1850 1899   -3   -.02  .02  .04 -.06 -.18  .12  .02  .29*-.01 -.11  .10|-.12  .01 -.05 -.01  .11 -.16 -.08  .16  .14 -.17
- - - - - - - - - - - - - - - - - - - - - - - - - - - - - - - - - - - - - - - - - - - - - - - - - - - - - - - - - - - - - -
1958 2007    0    .09  .07  .10  .00 -.25 -.16 -.02  .13 -.13  .02  .26* .09  .12 -.07 -.05 -.31  .26 -.18  .05  .03  .22
[B] Entire series, effect on correlation ( 0.353) is:
Lower   1846<-0.021   1798<-0.020   1877>-0.018   1858>-0.016   1802>-0.015   1865>-0.013  Higher   1948 0.043   1815 0.035
1798 to 1847 segment:
Lower   1846<-0.062   1798<-0.058   1802>-0.035   1813<-0.031   1817<-0.023   1799>-0.022  Higher   1815 0.132   1804 0.022
1800 to 1849 segment:
Lower   1846<-0.085   1802>-0.053   1813<-0.037   1817<-0.028   1823>-0.020   1818<-0.014  Higher   1815 0.132   1838 0.023
1825 to 1874 segment:
Lower   1858>-0.057   1846<-0.047   1865>-0.042   1861<-0.037   1872<-0.028   1835>-0.011  Higher   1868 0.052   1838 0.046
1850 to 1899 segment:
Lower   1858>-0.058   1877>-0.054   1865>-0.043   1861<-0.040   1872<-0.031   1882<-0.022  Higher   1893 0.091   1888 0.063
1958 to 2007 segment:
Lower   1994<-0.074   2003<-0.043   2007>-0.041   1996>-0.037   2000>-0.034   1968<-0.022  Higher   1976 0.141   1986 0.047
[C] Year-to-year changes diverging by over 4.0 std deviations:
1798 1799   4.6 SD
[E] Outliers     3   3.0 SD above or -4.5 SD below mean for year
1818 -4.9 SD;    1846 -4.5 SD;    1858 +3.2 SD
==================================================================================================================================
L7301     1809 to  2016     208 years                                                                                  Series  33
[B] Entire series, effect on correlation ( 0.613) is:
Lower   1907<-0.046   1926<-0.017   2007>-0.011   1852<-0.006   1823>-0.006   1910>-0.005  Higher   1948 0.028   1841 0.016
[E] Outliers     1   3.0 SD above or -4.5 SD below mean for year
1907 -4.7 SD
==================================================================================================================================
L7302     1799 to  2012     214 years                                                                                  Series  34
[B] Entire series, effect on correlation ( 0.658) is:
Lower   1802>-0.015   1933<-0.012   1908<-0.011   1896>-0.011   1909>-0.006   1907<-0.006  Higher   1976 0.012   1815 0.012
==================================================================================================================================
SD1330    1792 to  2021     230 years                                                                                  Series  35
[B] Entire series, effect on correlation ( 0.529) is:
Lower   1873<-0.028   1802>-0.023   1877>-0.011   1815>-0.010   1805<-0.009   1967<-0.008  Higher   1948 0.026   1841 0.017
[E] Outliers     1   3.0 SD above or -4.5 SD below mean for year
1888 -4.7 SD
==================================================================================================================================
SD1331    1804 to  2021     218 years                                                                                  Series  36
[B] Entire series, effect on correlation ( 0.525) is:
Lower   1815>-0.032   1873<-0.020   1944>-0.016   1877>-0.014   1847>-0.008   1824<-0.006  Higher   1948 0.037   1976 0.016
[C] Year-to-year changes diverging by over 4.0 std deviations:
1814 1815   4.2 SD
[E] Outliers     2   3.0 SD above or -4.5 SD below mean for year
1815 +3.3 SD;    1948 -5.0 SD
==================================================================================================================================
SD1401    1870 to  2021     152 years                                                                                  Series  37
[B] Entire series, effect on correlation ( 0.468) is:
Lower   1877>-0.027   2007>-0.020   2021<-0.014   1914<-0.012   1882<-0.010   1893>-0.010  Higher   1944 0.021   1976 0.017
==================================================================================================================================
SD1402    1845 to  2021     177 years                                                                                  Series  38
[B] Entire series, effect on correlation ( 0.567) is:
Lower   1909>-0.019   1919<-0.018   1858>-0.014   1860<-0.012   1944>-0.011   2007>-0.010  Higher   1976 0.028   1877 0.019
==================================================================================================================================
SD1621    1786 to  2021     236 years                                                                                  Series  39
[B] Entire series, effect on correlation ( 0.573) is:
Lower   1862<-0.011   1798<-0.011   1824<-0.008   1943<-0.007   1823<-0.006   1841>-0.006  Higher   1802 0.018   1877 0.017
[E] Outliers     1   3.0 SD above or -4.5 SD below mean for year
1824 -5.0 SD
==================================================================================================================================
SD1622    1786 to  2021     236 years                                                                                  Series  40
[A] Segment   High   -10   -9   -8   -7   -6   -5   -4   -3   -2   -1    0   +1   +2   +3   +4   +5   +6   +7   +8   +9  +10
---------  ----   ---  ---  ---  ---  ---  ---  ---  ---  ---  ---  ---  ---  ---  ---  ---  ---  ---  ---  ---  ---  ---
1850 1899    0   -.15  .15  .04 -.24  .03  .05  .01 -.04 -.30  .15  .31*-.30  .10  .04  .08 -.24 -.02  .28  .28 -.39  .05
[B] Entire series, effect on correlation ( 0.487) is:
Lower   1824<-0.013   1865>-0.010   1798<-0.009   1883<-0.008   1787<-0.008   1868>-0.008  Higher   1802 0.018   1909 0.013
1850 to 1899 segment:
Lower   1865>-0.035   1883<-0.035   1868>-0.033   1875<-0.029   1862<-0.024   1850<-0.019  Higher   1877 0.087   1858 0.057
[C] Year-to-year changes diverging by over 4.0 std deviations:
1822 1823  -4.3 SD
[E] Outliers     1   3.0 SD above or -4.5 SD below mean for year
1823 -5.3 SD
==================================================================================================================================
SD1650    1804 to  2021     218 years                                                                                  Series  41
[B] Entire series, effect on correlation ( 0.609) is:
Lower   1905<-0.040   1823>-0.016   2007>-0.008   1887<-0.005   1900>-0.005   1929<-0.005  Higher   1948 0.020   1815 0.015
==================================================================================================================================
SD1651    1798 to  2021     224 years                                                                                  Series  42
[B] Entire series, effect on correlation ( 0.725) is:
Lower   1923<-0.013   1887<-0.013   1802>-0.012   1918>-0.009   1823>-0.007   1838>-0.006  Higher   1815 0.017   1948 0.014
==================================================================================================================================
SD1720    1937 to  2021      85 years                                                                                  Series  43
[B] Entire series, effect on correlation ( 0.666) is:
Lower   2003<-0.022   1980>-0.018   2004>-0.015   2007>-0.010   2002>-0.010   1968<-0.009  Higher   1948 0.063   1976 0.025
==================================================================================================================================
SD1721    1934 to  2021      88 years                                                                                  Series  44
[B] Entire series, effect on correlation ( 0.719) is:
Lower   1990<-0.030   2003<-0.022   1994<-0.016   1996>-0.016   1935<-0.011   2007>-0.009  Higher   1976 0.042   1948 0.015
==================================================================================================================================
SD1750    1792 to  2021     230 years                                                                                  Series  45
[B] Entire series, effect on correlation ( 0.645) is:
```

```
Lower   1795<-0.015   1821<-0.009   1805<-0.009   2016>-0.008   1910>-0.008   2007>-0.006  Higher   1944 0.022   1841 0.016
====================================================================================================================================
SD1751    1813 to  2021     209 years                                                                                    Series  46
[B] Entire series, effect on correlation ( 0.651) is:
Lower   2007>-0.014   1815>-0.011   1921>-0.011   2016>-0.010   1821<-0.007   1823>-0.007  Higher   1877 0.020   1944 0.017
[E] Outliers     1   3.0 SD above or -4.5 SD below mean for year
1841 -5.9 SD
====================================================================================================================================
SD1752    1899 to  2021     123 years                                                                                    Series  47
[B] Entire series, effect on correlation ( 0.616) is:
Lower   1944>-0.021   2007>-0.015   2016>-0.015   1923<-0.014   1918>-0.011   1986>-0.010  Higher   1976 0.047   1948 0.024
====================================================================================================================================
SD1753    1806 to  2021     216 years                                                                                    Series  48
[B] Entire series, effect on correlation ( 0.561) is:
Lower   2016>-0.013   1879<-0.011   2007>-0.009   1837<-0.007   1838>-0.007   1882<-0.007  Higher   1841 0.025   1976 0.025
====================================================================================================================================
SD1754    1805 to  2021     217 years                                                                                    Series  49
[B] Entire series, effect on correlation ( 0.662) is:
Lower   1943<-0.011   1878<-0.011   2007>-0.008   1907<-0.007   1922>-0.005   1936<-0.005  Higher   1976 0.014   1944 0.011
[E] Outliers     1   3.0 SD above or -4.5 SD below mean for year
1858 -4.7 SD
====================================================================================================================================
SD1755    1783 to  2021     239 years                                                                                    Series  50
[B] Entire series, effect on correlation ( 0.592) is:
Lower   1820<-0.033   1944>-0.011   1980>-0.009   1789<-0.009   1786<-0.005   1946>-0.005  Higher   1802 0.044   1877 0.011
[C] Year-to-year changes diverging by over 4.0 std deviations:
1819 1820  -4.4 SD
[E] Outliers     1   3.0 SD above or -4.5 SD below mean for year
1820 -6.5 SD
====================================================================================================================================
SD1920    1841 to  2021     181 years                                                                                    Series  51
[B] Entire series, effect on correlation ( 0.687) is:
Lower   1841>-0.019   1868>-0.011   1944>-0.010   1943<-0.008   1992<-0.006   1880>-0.006  Higher   1976 0.019   1877 0.016
====================================================================================================================================
SD1921    1798 to  2021     224 years                                                                                    Series  52
[A] Segment   High   -10   -9   -8   -7   -6   -5   -4   -3   -2   -1    0   +1   +2   +3   +4   +5   +6   +7   +8   +9  +10
---------  ----   ---  ---  ---  ---  ---  ---  ---  ---  ---  ---  ---  ---  ---  ---  ---  ---  ---  ---  ---  ---  ---
1925 1974   10    .10  .02 -.11 -.09 -.04 -.34  .04 -.03  .17 -.17  .43|-.08  .12 -.28  .05 -.19  .11 -.09  .02 -.01  .45*
[B] Entire series, effect on correlation ( 0.661) is:
Lower   1944>-0.019   1931<-0.009   1918>-0.009   1966<-0.007   1930<-0.007   1838>-0.006  Higher   1815 0.023   1877 0.016
1925 to 1974 segment:
Lower   1944>-0.095   1931<-0.045   1966<-0.033   1930<-0.028   1943<-0.013   1958<-0.005  Higher   1948 0.119   1960 0.026
====================================================================================================================================
SD1930    1835 to  2021     187 years                                                                                    Series  53
[B] Entire series, effect on correlation ( 0.572) is:
Lower   1841>-0.015   1918>-0.012   2006<-0.010   1919>-0.010   1960>-0.009   1898<-0.009  Higher   1877 0.032   1944 0.022
[E] Outliers     1   3.0 SD above or -4.5 SD below mean for year
1958 +3.0 SD
====================================================================================================================================
SD1931    1798 to  2021     224 years                                                                                    Series  54
[B] Entire series, effect on correlation ( 0.664) is:
Lower   1820<-0.017   2007>-0.013   1847>-0.009   1910>-0.008   1902<-0.007   1840<-0.006  Higher   1802 0.033   1976 0.015
[E] Outliers     1   3.0 SD above or -4.5 SD below mean for year
1820 -5.3 SD
====================================================================================================================================
SD1970    1774 to  2021     248 years                                                                                    Series  55
[*] Early part of series cannot be checked from 1774 to 1775 -- not matched by another series
[B] Entire series, effect on correlation ( 0.643) is:
Lower   1791<-0.017   1799>-0.011   2007>-0.010   1875<-0.009   1865>-0.006   1815>-0.006  Higher   1802 0.042   1948 0.013
====================================================================================================================================
SD1971    1788 to  2021     234 years                                                                                    Series  56
[A] Segment   High   -10   -9   -8   -7   -6   -5   -4   -3   -2   -1    0   +1   +2   +3   +4   +5   +6   +7   +8   +9  +10
---------  ----   ---  ---  ---  ---  ---  ---  ---  ---  ---  ---  ---  ---  ---  ---  ---  ---  ---  ---  ---  ---  ---
1850 1899   -8    .12 -.03  .28*-.01 -.19 -.16 -.08 -.22 -.10  .15  .23| .08 -.03 -.02  .15 -.12 -.12 -.07 -.01 -.08  .06
1875 1924   -8    .20  .19  .21*-.09 -.08 -.04  .18 -.10 -.28  .11  .14|-.14 -.11  .09  .11 -.11  .00  .05 -.07  .03 -.05
[B] Entire series, effect on correlation ( 0.536) is:
Lower   1877>-0.031   1799>-0.011   1909>-0.011   1878<-0.010   1815>-0.010   2007>-0.009  Higher   1802 0.064   1976 0.022
1850 to 1899 segment:
Lower   1877>-0.126   1878<-0.043   1893>-0.031   1888>-0.029   1875<-0.026   1867<-0.016  Higher   1896 0.039   1851 0.038
1875 to 1924 segment:
Lower   1877>-0.122   1878<-0.041   1893>-0.031   1888>-0.030   1909>-0.030   1910>-0.024  Higher   1900 0.069   1918 0.045
[C] Year-to-year changes diverging by over 4.0 std deviations:
1876 1877   4.3 SD    1877 1878  -4.4 SD
[E] Outliers     1   3.0 SD above or -4.5 SD below mean for year
1877 +3.0 SD
====================================================================================================================================
SD1990    1800 to  2021     222 years                                                                                    Series  57
[B] Entire series, effect on correlation ( 0.497) is:
Lower   1822<-0.022   1867<-0.018   1823>-0.012   1841>-0.011   1888>-0.009   1842<-0.008  Higher   1815 0.029   1802 0.017
[C] Year-to-year changes diverging by over 4.0 std deviations:
1822 1823   4.6 SD
====================================================================================================================================
SD1991    1802 to  2021     220 years                                                                                    Series  58
[B] Entire series, effect on correlation ( 0.565) is:
Lower   1823>-0.014   2007>-0.011   1841>-0.011   1877>-0.011   1867<-0.008   1930<-0.008  Higher   1944 0.031   1948 0.019
====================================================================================================================================
SD2210    1796 to  2021     226 years                                                                                    Series  59
[B] Entire series, effect on correlation ( 0.619) is:
Lower   1801<-0.048   1799>-0.008   1850<-0.007   1916<-0.005   2019<-0.005   1869<-0.004  Higher   1877 0.022   1815 0.014
[C] Year-to-year changes diverging by over 4.0 std deviations:
1800 1801  -4.2 SD
====================================================================================================================================
```

```
SD2211    1800 to  2018     219 years                                                                                    Series  60
[B] Entire series, effect on correlation ( 0.570) is:
Lower   1823<-0.028   1802>-0.012   2008<-0.010   2007>-0.009   2014<-0.009   2013>-0.008  Higher   1877 0.027   1976 0.015
[C] Year-to-year changes diverging by over 4.0 std deviations:
1822 1823  -4.8 SD
[E] Outliers     1   3.0 SD above or -4.5 SD below mean for year
1823 -5.7 SD
====================================================================================================================================
SD2230    1776 to  2021     246 years                                                                                    Series  61
[B] Entire series, effect on correlation ( 0.567) is:
Lower   1788<-0.018   1865>-0.014   1924<-0.013   1780>-0.011   2007>-0.009   1859<-0.008  Higher   1802 0.048   1976 0.020
[E] Outliers     1   3.0 SD above or -4.5 SD below mean for year
1924 -4.8 SD
====================================================================================================================================
SD2231    1798 to  2021     224 years                                                                                    Series  62
[B] Entire series, effect on correlation ( 0.608) is:
Lower   1924<-0.022   2016>-0.008   1865>-0.008   1859<-0.007   1910>-0.007   2007>-0.007  Higher   1802 0.055   1948 0.016
[E] Outliers     1   3.0 SD above or -4.5 SD below mean for year
1924 -5.5 SD
====================================================================================================================================
SD2240    1795 to  2021     227 years                                                                                    Series  63
[B] Entire series, effect on correlation ( 0.594) is:
Lower   2016>-0.012   2006<-0.012   1904<-0.008   1909>-0.008   1937<-0.008   1992<-0.007  Higher   1948 0.025   1944 0.024
====================================================================================================================================
SD2241    1779 to  2021     243 years                                                                                    Series  64
[A] Segment   High   -10   -9   -8   -7   -6   -5   -4   -3   -2   -1    0   +1   +2   +3   +4   +5   +6   +7   +8   +9  +10
---------  ----   ---  ---  ---  ---  ---  ---  ---  ---  ---  ---  ---  ---  ---  ---  ---  ---  ---  ---  ---  ---  ---
1900 1949   -4   -.02 -.12  .02 -.05  .25  .08  .37* .01  .06 -.13  .36|-.35 -.07 -.02  .15 -.34  .23 -.12  .01 -.09  .06
[B] Entire series, effect on correlation ( 0.571) is:
Lower   1779>-0.036   1909>-0.009   1926<-0.006   2004>-0.006   1918>-0.006   1914<-0.006  Higher   1802 0.061   1815 0.014
1900 to 1949 segment:
Lower   1926<-0.038   1914<-0.033   1918>-0.033   1909>-0.032   1919>-0.024   1927<-0.019  Higher   1944 0.120   1948 0.083
[E] Outliers     2   3.0 SD above or -4.5 SD below mean for year
1779 +4.1 SD;    1802 -5.3 SD
====================================================================================================================================
SD2311    1777 to  2021     245 years                                                                                    Series  65
[B] Entire series, effect on correlation ( 0.704) is:
Lower   1996>-0.008   1837<-0.007   1791>-0.005   1782<-0.005   1804<-0.004   1847>-0.004  Higher   1877 0.010   1802 0.010
====================================================================================================================================
SD2312    1794 to  2021     228 years                                                                                    Series  66
[B] Entire series, effect on correlation ( 0.687) is:
Lower   1893<-0.023   1822<-0.009   2007>-0.008   1841>-0.005   1985>-0.005   2009<-0.005  Higher   1802 0.014   1976 0.011
[E] Outliers     1   3.0 SD above or -4.5 SD below mean for year
1893 -6.2 SD
====================================================================================================================================
SD2501    1825 to  2021     197 years                                                                                    Series  67
[A] Segment   High   -10   -9   -8   -7   -6   -5   -4   -3   -2   -1    0   +1   +2   +3   +4   +5   +6   +7   +8   +9  +10
---------  ----   ---  ---  ---  ---  ---  ---  ---  ---  ---  ---  ---  ---  ---  ---  ---  ---  ---  ---  ---  ---  ---
1972 2021    0   -.04  .01 -.06  .00  .04 -.03  .30 -.33  .10  .02  .30*-.21    -    -    -    -    -    -    -    -    -
[B] Entire series, effect on correlation ( 0.535) is:
Lower   1994<-0.017   1906<-0.013   2008<-0.012   2007>-0.012   2016>-0.012   1843>-0.011  Higher   1877 0.046   1841 0.021
1972 to 2021 segment:
Lower   1994<-0.073   2008<-0.052   2007>-0.047   2016>-0.043   1996>-0.041   1995>-0.027  Higher   1976 0.133   2019 0.035
====================================================================================================================================
SD2502    1797 to  2021     225 years                                                                                    Series  68
[A] Segment   High   -10   -9   -8   -7   -6   -5   -4   -3   -2   -1    0   +1   +2   +3   +4   +5   +6   +7   +8   +9  +10
---------  ----   ---  ---  ---  ---  ---  ---  ---  ---  ---  ---  ---  ---  ---  ---  ---  ---  ---  ---  ---  ---  ---
1797 1846    0    .13 -.06  .08 -.15 -.11 -.08 -.12 -.04  .06 -.21  .26* .01  .10  .07  .05  .18 -.12 -.10  .03  .09  .16
[B] Entire series, effect on correlation ( 0.498) is:
Lower   1802>-0.052   1804<-0.015   1918>-0.009   1964<-0.008   1893>-0.008   2016>-0.008  Higher   1944 0.038   1877 0.020
1797 to 1846 segment:
Lower   1802>-0.163   1804<-0.057   1797<-0.027   1810<-0.019   1843>-0.012   1821<-0.005  Higher   1841 0.093   1838 0.024
[C] Year-to-year changes diverging by over 4.0 std deviations:
1801 1802   4.7 SD
[E] Outliers     2   3.0 SD above or -4.5 SD below mean for year
1802 +4.1 SD;    1944 -4.9 SD
====================================================================================================================================
SD2560    1802 to  2021     220 years                                                                                    Series  69
[B] Entire series, effect on correlation ( 0.650) is:
Lower   1830<-0.009   1823>-0.009   1882<-0.009   1906<-0.008   1910>-0.007   1996>-0.006  Higher   1815 0.024   1976 0.012
====================================================================================================================================
SD2561    1813 to  2021     209 years                                                                                    Series  70
[B] Entire series, effect on correlation ( 0.644) is:
Lower   1868<-0.018   1858>-0.016   2007>-0.014   1888>-0.013   1823>-0.013   1959>-0.007  Higher   1815 0.022   1841 0.016
[E] Outliers     1   3.0 SD above or -4.5 SD below mean for year
1868 -5.1 SD
====================================================================================================================================
SD2580    1804 to  2021     218 years                                                                                    Series  71
[A] Segment   High   -10   -9   -8   -7   -6   -5   -4   -3   -2   -1    0   +1   +2   +3   +4   +5   +6   +7   +8   +9  +10
---------  ----   ---  ---  ---  ---  ---  ---  ---  ---  ---  ---  ---  ---  ---  ---  ---  ---  ---  ---  ---  ---  ---
1925 1974    0    .09  .06  .02  .01 -.18 -.14 -.04  .05 -.12  .23  .30*-.08  .21  .26  .10 -.26 -.10  .01 -.16 -.12 -.02
[B] Entire series, effect on correlation ( 0.604) is:
Lower   1945<-0.026   1944>-0.017   1841>-0.016   1960>-0.009   1923>-0.007   1840<-0.007  Higher   1815 0.020   1865 0.014
1925 to 1974 segment:
Lower   1945<-0.091   1944>-0.047   1960>-0.038   1947<-0.017   1973<-0.008   1963>-0.007  Higher   1948 0.038   1943 0.027
[C] Year-to-year changes diverging by over 4.0 std deviations:
1840 1841   4.3 SD    1944 1945  -4.7 SD
====================================================================================================================================
SD2581    1792 to  2021     230 years                                                                                    Series  72
[A] Segment   High   -10   -9   -8   -7   -6   -5   -4   -3   -2   -1    0   +1   +2   +3   +4   +5   +6   +7   +8   +9  +10
---------  ----   ---  ---  ---  ---  ---  ---  ---  ---  ---  ---  ---  ---  ---  ---  ---  ---  ---  ---  ---  ---  ---
1792 1841    1    .11 -.18 -.09  .24 -.12 -.30 -.10  .00  .16 -.09  .27| .49*-.15  .00 -.11 -.03 -.09 -.17  .01  .20 -.06
```

```
1800 1849    1    .09 -.19  .01  .14 -.13 -.28 -.12 -.03  .20 -.20  .30| .47*-.15  .10 -.03  .01 -.18 -.05 -.01  .11 -.11
[B] Entire series, effect on correlation ( 0.517) is:
Lower   1801<-0.027   1802>-0.025   1895<-0.011   1896>-0.010   2007>-0.010   1799>-0.009  Higher   1948 0.022   1976 0.022
1792 to 1841 segment:
Lower   1801<-0.089   1802>-0.044   1799>-0.023   1823>-0.023   1822<-0.017   1795>-0.015  Higher   1815 0.086   1837 0.021
1800 to 1849 segment:
Lower   1801<-0.090   1802>-0.050   1823>-0.023   1847>-0.021   1822<-0.019   1840<-0.015  Higher   1815 0.083   1848 0.020
[C] Year-to-year changes diverging by over 4.0 std deviations:
1801 1802   4.9 SD
===================================================================================================================================
SD2760    1793 to  2021     229 years                                                                                  Series  73
[A] Segment   High   -10   -9   -8   -7   -6   -5   -4   -3   -2   -1    0   +1   +2   +3   +4   +5   +6   +7   +8   +9  +10
---------  ----   ---  ---  ---  ---  ---  ---  ---  ---  ---  ---  ---  ---  ---  ---  ---  ---  ---  ---  ---  ---  ---
1875 1924    0   -.09 -.04 -.08 -.03 -.22 -.07  .03  .14  .06  .17  .25* .18 -.14  .00  .07 -.20 -.17 -.05 -.10  .02 -.05
1900 1949    0    .03 -.13 -.13 -.12  .09 -.04 -.03  .16  .15 -.16  .31* .11 -.06 -.17  .27  .05 -.04  .10 -.07 -.02  .20
1925 1974    4    .08 -.11  .16  .11 -.03 -.19  .10  .08 -.16 -.35  .32|-.05 -.20 -.11  .34* .21  .01  .18  .04  .12  .02
[B] Entire series, effect on correlation ( 0.466) is:
Lower   1801<-0.037   1900>-0.018   1802>-0.016   1799>-0.010   1955<-0.008   1888>-0.008  Higher   1815 0.031   1841 0.024
1875 to 1924 segment:
Lower   1900>-0.117   1888>-0.045   1897<-0.027   1885>-0.021   1911<-0.015   1884<-0.014  Higher   1877 0.109   1909 0.028
1900 to 1949 segment:
Lower   1900>-0.105   1939<-0.022   1911<-0.018   1925<-0.017   1931<-0.016   1916<-0.015  Higher   1944 0.133   1943 0.028
1925 to 1974 segment:
Lower   1955<-0.053   1952<-0.025   1931<-0.021   1971<-0.021   1939<-0.021   1928>-0.018  Higher   1944 0.155   1956 0.026
[C] Year-to-year changes diverging by over 4.0 std deviations:
1801 1802   5.2 SD
===================================================================================================================================
SD2761    1788 to  2021     234 years                                                                                  Series  74
[A] Segment   High   -10   -9   -8   -7   -6   -5   -4   -3   -2   -1    0   +1   +2   +3   +4   +5   +6   +7   +8   +9  +10
---------  ----   ---  ---  ---  ---  ---  ---  ---  ---  ---  ---  ---  ---  ---  ---  ---  ---  ---  ---  ---  ---  ---
1788 1837    1    .01  .02 -.06 -.08  .08 -.21 -.10  .09 -.02 -.24  .22| .53*-.23 -.17  .04  .07 -.12 -.14  .29 -.04 -.16
- - - - - - - - - - - - - - - - - - - - - - - - - - - - - - - - - - - - - - - - - - - - - - - - - - - - - - - - - - - - - -
1850 1899   -7   -.13 -.05 -.02  .31*-.17 -.04 -.12 -.04  .00  .00  .14| .27 -.02  .12  .00 -.10  .03 -.16 -.23  .14 -.04
1875 1924    1    .04 -.18 -.12  .19 -.17 -.30  .08  .21  .05  .07  .23| .25*-.11 -.01  .12 -.08 -.15 -.12 -.06  .13 -.18
[B] Entire series, effect on correlation ( 0.382) is:
Lower   1801<-0.037   1802>-0.022   1799>-0.013   1900>-0.012   1863<-0.011   1858>-0.010  Higher   1841 0.032   1815 0.018
1788 to 1837 segment:
Lower   1801<-0.109   1802>-0.061   1799>-0.039   1823>-0.026   1795>-0.017   1794<-0.012  Higher   1815 0.090   1834 0.025
1850 to 1899 segment:
Lower   1863<-0.055   1858>-0.042   1868>-0.036   1871<-0.031   1884<-0.030   1895<-0.029  Higher   1865 0.064   1877 0.032
1875 to 1924 segment:
Lower   1900>-0.079   1884<-0.045   1895<-0.033   1893>-0.027   1885>-0.021   1911<-0.012  Higher   1909 0.045   1905 0.030
[C] Year-to-year changes diverging by over 4.0 std deviations:
1794 1795   4.1 SD    1801 1802   5.1 SD
[E] Outliers     1   3.0 SD above or -4.5 SD below mean for year
1794 -4.6 SD
===================================================================================================================================
SD2780    1816 to  2021     206 years                                                                                  Series  75
[B] Entire series, effect on correlation ( 0.605) is:
Lower   2013<-0.018   2016>-0.013   1980>-0.011   1890<-0.009   1867<-0.009   1823>-0.009  Higher   1944 0.023   1841 0.022
===================================================================================================================================
SD2781    1833 to  2014     182 years                                                                                  Series  76
[B] Entire series, effect on correlation ( 0.673) is:
Lower   1867<-0.015   1980>-0.011   1878<-0.011   1837<-0.010   1900>-0.007   1848<-0.007  Higher   1976 0.021   1841 0.020
===================================================================================================================================
SD2791    1804 to  2021     218 years                                                                                  Series  77
[B] Entire series, effect on correlation ( 0.532) is:
Lower   2007>-0.013   2016>-0.010   1960>-0.009   1859<-0.009   1888>-0.008   1868>-0.008  Higher   1944 0.034   1815 0.030
===================================================================================================================================
SD2792    1833 to  2021     189 years                                                                                  Series  78
[B] Entire series, effect on correlation ( 0.600) is:
Lower   1893<-0.020   1877>-0.011   1985>-0.011   1875<-0.010   1996>-0.008   1847>-0.006  Higher   1909 0.020   1944 0.012
===================================================================================================================================
SD2801    1824 to  2021     198 years                                                                                  Series  79
[B] Entire series, effect on correlation ( 0.603) is:
Lower   1886<-0.013   1874<-0.010   1865>-0.009   1875<-0.009   1961<-0.008   1960>-0.007  Higher   1944 0.035   1976 0.018
===================================================================================================================================
SD2802    1823 to  2021     199 years                                                                                  Series  80
[A] Segment   High   -10   -9   -8   -7   -6   -5   -4   -3   -2   -1    0   +1   +2   +3   +4   +5   +6   +7   +8   +9  +10
---------  ----   ---  ---  ---  ---  ---  ---  ---  ---  ---  ---  ---  ---  ---  ---  ---  ---  ---  ---  ---  ---  ---
1925 1974    5   -.07  .01  .11  .05 -.15 -.10 -.08 -.10 -.26 -.13  .07| .02  .00  .25  .11  .37* .11  .22  .03 -.07  .08
[B] Entire series, effect on correlation ( 0.466) is:
Lower   1948>-0.023   1971<-0.017   1960>-0.016   1879<-0.014   1823>-0.010   1871<-0.007  Higher   1841 0.018   2016 0.012
1925 to 1974 segment:
Lower   1960>-0.063   1948>-0.061   1971<-0.045   1943<-0.023   1972>-0.017   1959>-0.017  Higher   1944 0.073   1956 0.039
===================================================================================================================================
SD2840    1827 to  2021     195 years                                                                                  Series  81
[B] Entire series, effect on correlation ( 0.629) is:
Lower   1931<-0.015   1919>-0.012   1941<-0.012   1886<-0.010   1918>-0.010   1909>-0.010  Higher   1841 0.022   1976 0.017
===================================================================================================================================
SD2841    1822 to  2021     200 years                                                                                  Series  82
[B] Entire series, effect on correlation ( 0.706) is:
Lower   1824<-0.010   1900>-0.009   2007>-0.008   1894<-0.006   1843>-0.005   2009<-0.005  Higher   1976 0.015   1948 0.013
[E] Outliers     1   3.0 SD above or -4.5 SD below mean for year
1841 -4.8 SD
===================================================================================================================================
SD2851    1795 to  2021     227 years                                                                                  Series  83
[B] Entire series, effect on correlation ( 0.625) is:
Lower   1823<-0.028   1877>-0.009   1975<-0.007   1864>-0.006   1948>-0.006   1853<-0.005  Higher   1802 0.018   1944 0.018
[C] Year-to-year changes diverging by over 4.0 std deviations:
1822 1823  -4.7 SD
[E] Outliers     1   3.0 SD above or -4.5 SD below mean for year
```

```
1823 -6.6 SD
===================================================================================================================================
SD2852    1815 to  2021     207 years                                                                                    Series  84
[B] Entire series, effect on correlation ( 0.578) is:
Lower   1852<-0.021   1884<-0.017   1948>-0.014   1999<-0.012   1877>-0.011   2007>-0.011  Higher   1944 0.034   1976 0.019
===================================================================================================================================
SD2930    1833 to  2021     189 years                                                                                    Series  85
[B] Entire series, effect on correlation ( 0.612) is:
Lower   1841>-0.017   1838>-0.011   1842<-0.010   1865>-0.009   1918>-0.009   1944>-0.005  Higher   1877 0.036   1976 0.021
===================================================================================================================================
SD2931    1805 to  2021     217 years                                                                                    Series  86
[B] Entire series, effect on correlation ( 0.609) is:
Lower   1885<-0.023   2007>-0.010   1941<-0.010   1909>-0.009   1852<-0.009   1969<-0.008  Higher   1877 0.029   1944 0.014
===================================================================================================================================
SD3771    1818 to  2021     204 years                                                                                    Series  87
[A] Segment   High   -10   -9   -8   -7   -6   -5   -4   -3   -2   -1    0   +1   +2   +3   +4   +5   +6   +7   +8   +9  +10
---------  ----   ---  ---  ---  ---  ---  ---  ---  ---  ---  ---  ---  ---  ---  ---  ---  ---  ---  ---  ---  ---  ---
1818 1867    3    .07  .04  .13  .15  .04  .14 -.12  .00  .09  .02 -.20|-.01 -.14  .21*-.12 -.13  .12 -.02 -.19  .04  .08
1825 1874    3    .10 -.02  .09  .10 -.10  .17 -.04  .02  .09  .01 -.17| .03 -.15  .18*-.09 -.10  .11 -.01 -.19  .01  .05
1850 1899   -8    .05 -.12  .22* .02  .13  .01 -.04  .20 -.07 -.17 -.05| .07 -.18  .08  .06 -.06  .02  .09 -.15 -.10  .20
1875 1924   -3    .01 -.10  .02 -.12 -.01 -.07  .03  .47*-.07 -.13 -.06|-.03 -.25  .04  .07  .10  .05  .15 -.07  .01  .15
1900 1949   -4    .21 -.17 -.20 -.08 -.15 -.20  .37* .18  .07 -.25  .29|-.30 -.17 -.06  .21  .15  .11  .06  .08  .03 -.09
[B] Entire series, effect on correlation ( 0.189) is:
Lower   1899<-0.034   1877>-0.026   1851<-0.023   1841>-0.022   1909>-0.021   1848<-0.020  Higher   1944 0.059   1948 0.031
1818 to 1867 segment:
Lower   1851<-0.061   1841>-0.053   1848<-0.050   1858>-0.039   1857>-0.022   1821<-0.014  Higher   1843 0.043   1837 0.034
1825 to 1874 segment:
Lower   1851<-0.064   1841>-0.054   1848<-0.052   1858>-0.040   1857>-0.023   1829<-0.010  Higher   1843 0.048   1837 0.030
1850 to 1899 segment:
Lower   1899<-0.073   1851<-0.068   1877>-0.055   1858>-0.034   1857>-0.018   1895<-0.011  Higher   1896 0.084   1875 0.037
1875 to 1924 segment:
Lower   1899<-0.070   1877>-0.055   1909>-0.048   1900>-0.037   1913<-0.014   1895<-0.007  Higher   1896 0.069   1875 0.040
1900 to 1949 segment:
Lower   1909>-0.096   1900>-0.066   1939<-0.030   1912<-0.029   1913<-0.027   1924>-0.019  Higher   1944 0.214   1948 0.096
[C] Year-to-year changes diverging by over 4.0 std deviations:
1844 1845   4.6 SD    1847 1848  -4.0 SD    1899 1900   4.0 SD
[E] Outliers     4   3.0 SD above or -4.5 SD below mean for year
1858 +3.6 SD;    1877 +4.4 SD;    1909 +3.7 SD;    1913 -6.1 SD
===================================================================================================================================
SD3772    1785 to  2021     237 years                                                                                    Series  88
[A] Segment   High   -10   -9   -8   -7   -6   -5   -4   -3   -2   -1    0   +1   +2   +3   +4   +5   +6   +7   +8   +9  +10
---------  ----   ---  ---  ---  ---  ---  ---  ---  ---  ---  ---  ---  ---  ---  ---  ---  ---  ---  ---  ---  ---  ---
1785 1834    3    .06  .12  .02  .09 -.03 -.11 -.13 -.18 -.13 -.18  .37|-.02 -.08  .42*-.21 -.12  .03  .03  .04 -.08 -.01
1800 1849   10    .18  .22 -.04  .22  .04 -.11 -.26 -.02 -.26 -.31  .16|-.06 -.12  .08 -.07 -.04  .18  .05  .24  .00  .29*
1825 1874   -7    .10 -.06  .07  .23* .04 -.02 -.07 -.09  .17 -.29 -.03|-.15 -.11  .11  .07 -.09  .07 -.20  .13  .05  .22
1850 1899    3   -.01 -.04  .00  .09  .00 -.10  .09 -.02  .16 -.09  .05|-.09 -.12  .23* .09 -.10  .03  .04 -.18  .18  .10
1875 1924    7   -.12 -.03  .10 -.19 -.17 -.11  .09  .02  .05  .16  .05|-.01 -.19 -.10 -.10  .01  .11  .47* .00  .26  .03
1900 1949    0    .10 -.08  .13 -.13 -.17 -.26  .09 -.11  .10 -.22  .31*-.17 -.18 -.29  .17  .04  .10  .23  .22  .01  .07
- - - - - - - - - - - - - - - - - - - - - - - - - - - - - - - - - - - - - - - - - - - - - - - - - - - - - - - - - - - - - -
1950 1999    0    .09  .24  .22  .09  .05 -.09 -.04 -.15 -.17 -.17  .29*-.12  .06 -.14  .05 -.12 -.03  .10  .18 -.08 -.08
[B] Entire series, effect on correlation ( 0.231) is:
Lower   1877>-0.027   1841>-0.019   1858>-0.015   1857>-0.012   1911<-0.008   1918>-0.008  Higher   1944 0.029   1799 0.028
1785 to 1834 segment:
Lower   1823>-0.019   1809<-0.018   1811<-0.017   1815>-0.015   1820>-0.013   1790<-0.012  Higher   1799 0.064   1802 0.048
1800 to 1849 segment:
Lower   1841>-0.087   1809<-0.027   1811<-0.023   1837<-0.023   1843>-0.021   1823>-0.017  Higher   1802 0.186   1815 0.031
1825 to 1874 segment:
Lower   1841>-0.088   1858>-0.073   1857>-0.056   1843>-0.023   1867<-0.018   1837<-0.017  Higher   1865 0.053   1863 0.042
1850 to 1899 segment:
Lower   1877>-0.114   1858>-0.064   1857>-0.049   1896>-0.030   1867<-0.019   1886<-0.018  Higher   1865 0.041   1875 0.040
1875 to 1924 segment:
Lower   1877>-0.121   1911<-0.038   1918>-0.031   1896>-0.030   1886<-0.023   1900>-0.013  Higher   1875 0.047   1893 0.039
1900 to 1949 segment:
Lower   1911<-0.046   1931<-0.038   1918>-0.038   1929<-0.028   1909>-0.025   1900>-0.023  Higher   1944 0.150   1943 0.035
1950 to 1999 segment:
Lower   1959>-0.056   1980>-0.046   1955<-0.030   1965<-0.027   1995>-0.026   1984<-0.025  Higher   1996 0.057   1976 0.048
[E] Outliers     4   3.0 SD above or -4.5 SD below mean for year
1841 +3.5 SD;    1858 +3.8 SD;    1877 +3.7 SD;    1911 -4.8 SD
===================================================================================================================================
SD4361    1846 to  2021     176 years                                                                                    Series  89
[B] Entire series, effect on correlation ( 0.621) is:
Lower   1948>-0.029   1916<-0.009   1882<-0.009   1914<-0.008   1911<-0.007   1995>-0.007  Higher   1944 0.037   1976 0.021
[E] Outliers     1   3.0 SD above or -4.5 SD below mean for year
1944 -6.1 SD
===================================================================================================================================
SD4362    1878 to  2021     144 years                                                                                    Series  90
[A] Segment   High   -10   -9   -8   -7   -6   -5   -4   -3   -2   -1    0   +1   +2   +3   +4   +5   +6   +7   +8   +9  +10
---------  ----   ---  ---  ---  ---  ---  ---  ---  ---  ---  ---  ---  ---  ---  ---  ---  ---  ---  ---  ---  ---  ---
1878 1927    0   -.28 -.14  .07  .00 -.08  .05  .01 -.16 -.04  .02  .33*-.03  .16  .20 -.01  .01 -.06  .05 -.06 -.11  .07
[B] Entire series, effect on correlation ( 0.535) is:
Lower   1948>-0.030   1907<-0.018   1971<-0.012   2006<-0.011   1918>-0.010   1916<-0.010  Higher   1944 0.058   1986 0.019
1878 to 1927 segment:
Lower   1907<-0.056   1880>-0.032   1918>-0.031   1916<-0.030   1891>-0.024   1920<-0.022  Higher   1893 0.052   1888 0.050
===================================================================================================================================
SD4521    1813 to  2021     209 years                                                                                    Series  91
[B] Entire series, effect on correlation ( 0.639) is:
Lower   2016>-0.019   1968<-0.012   1860<-0.007   2011>-0.007   1864<-0.007   1896>-0.007  Higher   1877 0.028   1976 0.015
===================================================================================================================================
SD4522    1797 to  2021     225 years                                                                                    Series  92
[B] Entire series, effect on correlation ( 0.622) is:
Lower   1805<-0.016   1802>-0.015   1986>-0.011   1847>-0.008   1985>-0.006   1798<-0.006  Higher   1815 0.018   1976 0.011
===================================================================================================================================
```

```
SD4531    1829 to  2021     193 years                                                                                    Series  93
[A] Segment   High   -10   -9   -8   -7   -6   -5   -4   -3   -2   -1    0   +1   +2   +3   +4   +5   +6   +7   +8   +9  +10
---------  ----   ---  ---  ---  ---  ---  ---  ---  ---  ---  ---  ---  ---  ---  ---  ---  ---  ---  ---  ---  ---  ---
1829 1878    3   -.17  .25  .21 -.19  .11 -.07 -.09 -.09 -.04 -.26  .00|-.05 -.05  .38* .09  .11  .10 -.01 -.02  .06 -.06
1850 1899   -8   -.05  .13  .31*-.01  .00 -.16  .08  .07 -.11 -.27  .13| .10  .01  .21  .26  .09 -.16 -.01 -.05 -.21 -.15
[B] Entire series, effect on correlation ( 0.399) is:
Lower   1975<-0.037   1877>-0.019   1841>-0.016   1858>-0.013   1857>-0.012   1900>-0.011  Higher   1948 0.050   2016 0.017
1829 to 1878 segment:
Lower   1857>-0.038   1858>-0.031   1843>-0.026   1841>-0.025   1855<-0.021   1863<-0.021  Higher   1865 0.049   1875 0.041
1850 to 1899 segment:
Lower   1857>-0.050   1877>-0.045   1858>-0.044   1887<-0.034   1886<-0.028   1895<-0.027  Higher   1896 0.050   1865 0.038
[C] Year-to-year changes diverging by over 4.0 std deviations:
1974 1975  -4.4 SD
[E] Outliers     1   3.0 SD above or -4.5 SD below mean for year
1975 -4.6 SD
================================================================================================================================
SD4532    1871 to  2021     151 years                                                                                    Series  94
[A] Segment   High   -10   -9   -8   -7   -6   -5   -4   -3   -2   -1    0   +1   +2   +3   +4   +5   +6   +7   +8   +9  +10
---------  ----   ---  ---  ---  ---  ---  ---  ---  ---  ---  ---  ---  ---  ---  ---  ---  ---  ---  ---  ---  ---  ---
1900 1949    8   -.01 -.08  .00 -.29  .07 -.20  .04 -.08  .02 -.22  .38|-.07  .02 -.26  .33 -.11  .04 -.11  .38* .05 -.01
[B] Entire series, effect on correlation ( 0.499) is:
Lower   1940<-0.051   1900>-0.022   1877>-0.020   1944>-0.017   1959<-0.009   1918>-0.007  Higher   1909 0.034   1976 0.025
1900 to 1949 segment:
Lower   1940<-0.118   1900>-0.053   1944>-0.041   1918>-0.016   1919>-0.014   1930<-0.006  Higher   1909 0.112   1948 0.063
[C] Year-to-year changes diverging by over 4.0 std deviations:
1940 1941   4.1 SD
[E] Outliers     2   3.0 SD above or -4.5 SD below mean for year
1940 -6.4 SD;    1959 -4.9 SD
================================================================================================================================
SD4551    1819 to  2017     199 years                                                                                    Series  95
[B] Entire series, effect on correlation ( 0.721) is:
Lower   2007>-0.017   1888>-0.010   1855<-0.009   1883<-0.006   1857>-0.005   1858>-0.005  Higher   1877 0.017   1976 0.015
================================================================================================================================
SD4581    1797 to  2017     221 years                                                                                    Series  96
[A] Segment   High   -10   -9   -8   -7   -6   -5   -4   -3   -2   -1    0   +1   +2   +3   +4   +5   +6   +7   +8   +9  +10
---------  ----   ---  ---  ---  ---  ---  ---  ---  ---  ---  ---  ---  ---  ---  ---  ---  ---  ---  ---  ---  ---  ---
1797 1846    3    .25  .22  .14  .23 -.14 -.02 -.21  .01 -.19 -.25  .31| .04 -.25  .31* .19 -.12  .15  .03 -.12  .03 -.13
1800 1849    3    .18  .24  .16  .15 -.12 -.07 -.24  .02 -.12 -.25  .33| .01 -.22  .34* .07 -.05  .18  .04 -.09  .05 -.16
- - - - - - - - - - - - - - - - - - - - - - - - - - - - - - - - - - - - - - - - - - - - - - - - - - - - - - - - - - - - - -
1900 1949    4    .01 -.19 -.24  .00  .12 -.22 -.09 -.11 -.22 -.36 -.03|-.03  .05  .31  .32* .14  .23  .09 -.09  .11  .06
1925 1974    4   -.09  .08 -.02 -.06  .02 -.14  .17 -.05 -.35 -.08  .07|-.18 -.06  .10  .24* .21  .20  .17  .13 -.13 -.05
[B] Entire series, effect on correlation ( 0.387) is:
Lower   1802>-0.027   1948>-0.021   1809<-0.017   1811<-0.014   1906<-0.010   1798<-0.009  Higher   1877 0.047   1865 0.013
1797 to 1846 segment:
Lower   1802>-0.089   1809<-0.065   1811<-0.049   1798<-0.031   1846<-0.008   1840<-0.006  Higher   1838 0.037   1823 0.036
1800 to 1849 segment:
Lower   1802>-0.089   1809<-0.071   1811<-0.053   1846<-0.009   1840<-0.007   1847>-0.006  Higher   1838 0.039   1823 0.035
1900 to 1949 segment:
Lower   1948>-0.060   1906<-0.051   1943<-0.032   1918>-0.026   1907<-0.023   1921>-0.018  Higher   1944 0.093   1909 0.054
1925 to 1974 segment:
Lower   1948>-0.105   1959>-0.027   1964<-0.025   1943<-0.024   1952<-0.023   1956>-0.012  Higher   1944 0.059   1960 0.042
[E] Outliers     2   3.0 SD above or -4.5 SD below mean for year
1802 +3.4 SD;    1948 +3.5 SD
================================================================================================================================
SD5341    1803 to  2017     215 years                                                                                    Series  97
[A] Segment   High   -10   -9   -8   -7   -6   -5   -4   -3   -2   -1    0   +1   +2   +3   +4   +5   +6   +7   +8   +9  +10
---------  ----   ---  ---  ---  ---  ---  ---  ---  ---  ---  ---  ---  ---  ---  ---  ---  ---  ---  ---  ---  ---  ---
1803 1852    0    .09  .21  .17 -.02 -.13 -.21  .13  .11 -.38  .09  .28*-.17 -.15 -.13 -.04 -.13  .24  .09  .23  .03 -.16
1825 1874    6    .13  .13  .19  .15  .02  .01 -.08  .03 -.31 -.06  .17|-.18 -.09 -.06  .07 -.13  .29* .08  .08 -.08 -.21
[B] Entire series, effect on correlation ( 0.483) is:
Lower   1894<-0.014   1843>-0.012   1857>-0.011   1893>-0.010   1851<-0.010   1838>-0.010  Higher   1976 0.031   1815 0.022
1803 to 1852 segment:
Lower   1843>-0.046   1838>-0.037   1851<-0.033   1847>-0.032   1820>-0.030   1839<-0.027  Higher   1815 0.149   1823 0.059
1825 to 1874 segment:
Lower   1843>-0.052   1857>-0.043   1851<-0.041   1838>-0.038   1847>-0.034   1839<-0.034  Higher   1865 0.105   1868 0.050
================================================================================================================================
SD6311    1856 to  2016     161 years                                                                                    Series  98
[B] Entire series, effect on correlation ( 0.700) is:
Lower   1888>-0.020   1909>-0.015   2007>-0.013   1895<-0.008   1900>-0.008   1956>-0.007  Higher   1877 0.029   1948 0.027
================================================================================================================================
SD6681    1851 to  2021     171 years                                                                                    Series  99
[B] Entire series, effect on correlation ( 0.773) is:
Lower   1944>-0.017   1980>-0.010   1985>-0.007   2007>-0.006   1938<-0.005   1856<-0.004  Higher   1948 0.019   1865 0.009
================================================================================================================================
SD6682    1848 to  2021     174 years                                                                                    Series 100
[B] Entire series, effect on correlation ( 0.693) is:
Lower   1944>-0.014   1992<-0.011   1968<-0.008   1985>-0.006   1939<-0.005   1932<-0.005  Higher   1948 0.021   1976 0.020
================================================================================================================================
SD7221    1795 to  2021     227 years                                                                                    Series 101
[A] Segment   High   -10   -9   -8   -7   -6   -5   -4   -3   -2   -1    0   +1   +2   +3   +4   +5   +6   +7   +8   +9  +10
---------  ----   ---  ---  ---  ---  ---  ---  ---  ---  ---  ---  ---  ---  ---  ---  ---  ---  ---  ---  ---  ---  ---
1875 1924    0   -.01 -.23 -.03  .04 -.06 -.15  .05  .17  .08 -.09  .23* .17 -.30 -.10 -.03  .04 -.12  .12  .10  .06 -.34
1900 1949    0    .07 -.21 -.08 -.11  .00  .01  .02  .07  .14  .17  .25*-.14  .01 -.23  .00 -.23  .00  .10  .05 -.02  .03
1925 1974   -8   -.02 -.04  .25* .18 -.03  .09  .09  .06 -.21 -.07  .19|-.28  .10 -.03  .19 -.21  .08  .07  .12 -.07  .11
[B] Entire series, effect on correlation ( 0.487) is:
Lower   1900>-0.013   1960>-0.012   1823>-0.009   1831>-0.009   1918>-0.009   1911<-0.008  Higher   1815 0.036   1802 0.024
1875 to 1924 segment:
Lower   1900>-0.059   1911<-0.042   1918>-0.040   1895<-0.033   1888>-0.022   1884<-0.018  Higher   1877 0.062   1893 0.037
1900 to 1949 segment:
Lower   1900>-0.058   1911<-0.044   1918>-0.039   1949<-0.031   1927<-0.015   1919>-0.012  Higher   1948 0.034   1910 0.029
1925 to 1974 segment:
Lower   1960>-0.067   1973<-0.030   1949<-0.028   1959>-0.023   1951<-0.018   1927<-0.015  Higher   1948 0.057   1956 0.028
```

```
==================================================================================================================================
SD7222    1780 to  2021     242 years                                                                                    Series 102
[B] Entire series, effect on correlation ( 0.648) is:
Lower   1911<-0.015   1831>-0.010   1892<-0.009   2007>-0.009   1851<-0.007   1857>-0.007  Higher   1802 0.027   1815 0.023
==================================================================================================================================
SD81      1873 to  2021     149 years                                                                                    Series 103
[B] Entire series, effect on correlation ( 0.661) is:
Lower   1974<-0.020   1888>-0.018   1980>-0.010   2014<-0.010   1900>-0.009   1985>-0.007  Higher   1944 0.041   1948 0.026
==================================================================================================================================
SD82      1841 to  2021     181 years                                                                                    Series 104
[A] Segment   High   -10   -9   -8   -7   -6   -5   -4   -3   -2   -1    0   +1   +2   +3   +4   +5   +6   +7   +8   +9  +10
---------  ----   ---  ---  ---  ---  ---  ---  ---  ---  ---  ---  ---  ---  ---  ---  ---  ---  ---  ---  ---  ---  ---
1841 1890   -4   -.03 -.07  .00 -.19  .00 -.03  .33* .15  .06 -.09  .32|-.23 -.14 -.20 -.21 -.15 -.12  .02  .19  .28  .02
[B] Entire series, effect on correlation ( 0.568) is:
Lower   1990<-0.026   1865>-0.017   1888>-0.012   1848<-0.011   1841>-0.011   1922>-0.010  Higher   1944 0.040   1976 0.027
1841 to 1890 segment:
Lower   1848<-0.046   1865>-0.037   1849<-0.032   1888>-0.032   1860<-0.019   1864>-0.014  Higher   1877 0.055   1847 0.036
==================================================================================================================================
SD83      1843 to  2017     175 years                                                                                    Series 105
[B] Entire series, effect on correlation ( 0.570) is:
Lower   1948>-0.018   1848<-0.015   1843>-0.012   1865>-0.011   1922>-0.010   1966<-0.010  Higher   1976 0.031   1944 0.031
==================================================================================================================================
SD8601    1777 to  2017     241 years                                                                                    Series 106
[A] Segment   High   -10   -9   -8   -7   -6   -5   -4   -3   -2   -1    0   +1   +2   +3   +4   +5   +6   +7   +8   +9  +10
---------  ----   ---  ---  ---  ---  ---  ---  ---  ---  ---  ---  ---  ---  ---  ---  ---  ---  ---  ---  ---  ---  ---
1825 1874   -4    .00 -.01  .02  .08 -.18 -.31  .35* .22  .07 -.01  .31| .00  .16  .00 -.06  .08 -.09 -.32  .25  .02 -.36
1850 1899   -4   -.09 -.11  .16  .14 -.21 -.22  .35* .09  .07 -.09  .10| .10 -.19 -.03  .24  .03 -.07 -.04  .20  .27 -.40
1875 1924    4   -.16 -.18  .20 -.03 -.09  .14  .13 -.09  .25 -.02  .03| .11 -.35 -.16  .31* .03  .14  .28 -.01  .01 -.23
1900 1949   -2   -.16 -.16  .03 -.06 -.05  .15 -.04 -.06  .27*-.01  .07|-.01 -.16 -.29  .12 -.01 -.02  .17  .08  .08 -.02
1925 1974   -2    .02 -.11 -.01 -.18  .10  .02 -.20 -.19  .28*-.15  .12|-.02  .08 -.24  .06 -.02 -.06  .06  .04  .24  .08
1950 1999    0    .16  .10  .03 -.30  .18  .05 -.15 -.28  .16 -.25  .31* .11  .05 -.25  .11  .09  .15  .07 -.21  .07  .17
[B] Entire series, effect on correlation ( 0.349) is:
Lower   1877>-0.029   1944>-0.017   1823>-0.014   1858>-0.012   1900>-0.011   1960>-0.010  Higher   1815 0.056   1802 0.049
1825 to 1874 segment:
Lower   1858>-0.072   1857>-0.040   1861<-0.039   1839<-0.023   1856<-0.022   1872<-0.020  Higher   1865 0.056   1834 0.034
1850 to 1899 segment:
Lower   1877>-0.124   1858>-0.049   1857>-0.028   1861<-0.027   1884<-0.024   1872<-0.014  Higher   1875 0.064   1865 0.061
1875 to 1924 segment:
Lower   1877>-0.111   1900>-0.040   1911<-0.039   1909>-0.024   1884<-0.023   1921>-0.020  Higher   1875 0.077   1899 0.029
1900 to 1949 segment:
Lower   1944>-0.052   1911<-0.044   1900>-0.043   1909>-0.026   1921>-0.021   1935<-0.018  Higher   1948 0.067   1907 0.027
1925 to 1974 segment:
Lower   1944>-0.083   1960>-0.061   1965<-0.029   1950<-0.019   1935<-0.016   1971<-0.015  Higher   1948 0.078   1955 0.033
1950 to 1999 segment:
Lower   1960>-0.077   1965<-0.044   1994<-0.028   1990>-0.027   1977>-0.026   1950<-0.025  Higher   1996 0.044   1995 0.037
[E] Outliers     3   3.0 SD above or -4.5 SD below mean for year
1815 -4.9 SD;    1858 +3.1 SD;    1877 +3.5 SD
==================================================================================================================================
SD9531    1811 to  2021     211 years                                                                                    Series 107
[A] Segment   High   -10   -9   -8   -7   -6   -5   -4   -3   -2   -1    0   +1   +2   +3   +4   +5   +6   +7   +8   +9  +10
---------  ----   ---  ---  ---  ---  ---  ---  ---  ---  ---  ---  ---  ---  ---  ---  ---  ---  ---  ---  ---  ---  ---
1811 1860    0   -.17 -.05 -.18  .01  .07  .07  .01 -.09 -.24 -.01  .30*-.08 -.04  .20  .23  .24  .01 -.24 -.09 -.26 -.08
- - - - - - - - - - - - - - - - - - - - - - - - - - - - - - - - - - - - - - - - - - - - - - - - - - - - - - - - - - - - - -
1875 1924    3    .23  .07  .13  .00  .16 -.21 -.19 -.03 -.23 -.25  .26| .13 -.14  .33* .24 -.13  .01 -.18 -.17  .12 -.16
[B] Entire series, effect on correlation ( 0.465) is:
Lower   1906<-0.071   1944>-0.019   1893>-0.014   1837<-0.010   1887<-0.008   1921>-0.007  Higher   1948 0.037   1976 0.022
1811 to 1860 segment:
Lower   1837<-0.045   1853<-0.025   1843>-0.024   1811<-0.022   1814>-0.018   1823>-0.018  Higher   1815 0.100   1838 0.047
1875 to 1924 segment:
Lower   1906<-0.219   1893>-0.043   1887<-0.023   1921>-0.020   1910>-0.020   1876<-0.010  Higher   1909 0.091   1877 0.022
[C] Year-to-year changes diverging by over 4.0 std deviations:
1905 1906  -4.7 SD    1906 1907   4.1 SD
[E] Outliers     1   3.0 SD above or -4.5 SD below mean for year
1906 -7.1 SD
==================================================================================================================================
SD9532    1849 to  2021     173 years                                                                                    Series 108
[B] Entire series, effect on correlation ( 0.565) is:
Lower   1944>-0.023   1906<-0.013   1914<-0.010   1934>-0.010   1888>-0.009   1857>-0.008  Higher   1948 0.041   1877 0.016
==================================================================================================================================
NG1070    1941 to  2021      81 years                                                                                    Series 109
[B] Entire series, effect on correlation ( 0.494) is:
Lower   2007<-0.026   1952<-0.021   1951<-0.014   1969<-0.011   1965<-0.010   1956>-0.010  Higher   1976 0.020   2016 0.018
[E] Outliers     1   3.0 SD above or -4.5 SD below mean for year
2007 -4.9 SD
==================================================================================================================================
NG1071    1941 to  2021      81 years                                                                                    Series 110
[B] Entire series, effect on correlation ( 0.566) is:
Lower   1976>-0.034   2009<-0.025   1965<-0.019   2007>-0.015   2002>-0.013   1960>-0.011  Higher   1944 0.059   1948 0.049
==================================================================================================================================
NG1110    1956 to  2021      66 years                                                                                    Series 111
[B] Entire series, effect on correlation ( 0.372) is:
Lower   1986>-0.041   2000>-0.039   2013>-0.021   1973<-0.021   1959>-0.020   1975<-0.018  Higher   1960 0.054   1976 0.044
==================================================================================================================================
NG1111    1965 to  2021      57 years                                                                                    Series 112
[A] Segment   High   -10   -9   -8   -7   -6   -5   -4   -3   -2   -1    0   +1   +2   +3   +4   +5   +6   +7   +8   +9  +10
---------  ----   ---  ---  ---  ---  ---  ---  ---  ---  ---  ---  ---  ---  ---  ---  ---  ---  ---  ---  ---  ---  ---
1965 2014    3   -.03 -.02 -.19 -.05 -.06  .09  .01  .26 -.19  .00 -.03| .04  .12  .32*-.10 -.12  .09  .20 -.13    -    -
1972 2021   -3   -.05 -.02 -.17 -.06 -.06  .10  .02  .13*-.13 -.02 -.01| .08    -    -    -    -    -    -    -    -    -    -
[B] Entire series, effect on correlation (-0.001) is:
Lower   2011>-0.038   2015<-0.025   1995>-0.023   2001<-0.020   2007>-0.020   1973<-0.019  Higher   2008 0.035   2019 0.034
1965 to 2014 segment:
Lower   2011>-0.042   1995>-0.026   2007>-0.022   1973<-0.021   1986>-0.019   2001<-0.019  Higher   2008 0.041   1976 0.033
```

```
1972 to 2021 segment:
Lower   2011>-0.040   2015<-0.028   1995>-0.025   2001<-0.024   2007>-0.022   1986>-0.021  Higher   2008 0.041   2019 0.040
====================================================================================================================================
NG1120    1952 to  2021      70 years                                                                                  Series 113
[A] Segment   High   -10   -9   -8   -7   -6   -5   -4   -3   -2   -1    0   +1   +2   +3   +4   +5   +6   +7   +8   +9  +10
---------  ----   ---  ---  ---  ---  ---  ---  ---  ---  ---  ---  ---  ---  ---  ---  ---  ---  ---  ---  ---  ---  ---
1972 2021    0    .16  .03 -.31 -.06 -.15  .08  .14 -.06 -.02  .06  .31*-.05    -    -    -    -    -    -    -    -    -    -
[B] Entire series, effect on correlation ( 0.349) is:
Lower   1973<-0.067   2016>-0.036   1996>-0.018   1981<-0.017   2013>-0.015   1989<-0.015  Higher   1976 0.067   1982 0.022
1972 to 2021 segment:
Lower   1973<-0.088   2016>-0.044   1981<-0.023   1996>-0.021   1989<-0.020   2013>-0.018  Higher   1976 0.094   1982 0.034
====================================================================================================================================
NG1121    1943 to  2021      79 years                                                                                  Series 114
[A] Segment   High   -10   -9   -8   -7   -6   -5   -4   -3   -2   -1    0   +1   +2   +3   +4   +5   +6   +7   +8   +9  +10
---------  ----   ---  ---  ---  ---  ---  ---  ---  ---  ---  ---  ---  ---  ---  ---  ---  ---  ---  ---  ---  ---  ---
1950 1999    0    .01 -.07 -.17 -.06 -.14 -.02  .12  .25 -.03 -.12  .26*-.07 -.02 -.03 -.24 -.06  .26  .25 -.17 -.06  .06
1972 2021    0    .00  .22 -.14 -.10 -.08  .14  .02  .10 -.03 -.12  .25*-.07    -    -    -    -    -    -    -    -    -    -
[B] Entire series, effect on correlation ( 0.341) is:
Lower   1964<-0.025   1948>-0.023   2000>-0.022   1993<-0.021   1962<-0.020   1943<-0.019  Higher   1944 0.107   1960 0.017
1950 to 1999 segment:
Lower   1996>-0.037   1964<-0.035   1993<-0.034   1962<-0.029   1973<-0.024   1951<-0.013  Higher   1960 0.040   1976 0.035
1972 to 2021 segment:
Lower   1993<-0.043   2000>-0.039   1973<-0.032   1996>-0.031   2001<-0.021   1981<-0.017  Higher   1976 0.048   1982 0.031
====================================================================================================================================
NG1180    1961 to  2021      61 years                                                                                  Series 115
[A] Segment   High   -10   -9   -8   -7   -6   -5   -4   -3   -2   -1    0   +1   +2   +3   +4   +5   +6   +7   +8   +9  +10
---------  ----   ---  ---  ---  ---  ---  ---  ---  ---  ---  ---  ---  ---  ---  ---  ---  ---  ---  ---  ---  ---  ---
1961 2010   -3    .19  .13 -.13 -.04 -.06  .02  .01  .25*-.01  .16  .24| .07 -.11 -.10 -.17 -.17  .17 -.09 -.03 -.11  .05
1972 2021  -10    .22* .19 -.05 -.07 -.12  .19 -.01  .12 -.13  .14  .20| .02    -    -    -    -    -    -    -    -    -    -
[B] Entire series, effect on correlation ( 0.199) is:
Lower   2021<-0.060   2004>-0.037   1962<-0.037   1996>-0.023   2011>-0.018   1988<-0.018  Higher   1986 0.044   1980 0.030
1961 to 2010 segment:
Lower   2004>-0.052   1962<-0.052   1996>-0.032   1988<-0.024   2001<-0.023   1999<-0.022  Higher   1986 0.057   1980 0.039
1972 to 2021 segment:
Lower   2021<-0.076   2004>-0.040   1996>-0.025   2001<-0.020   1988<-0.020   2005<-0.019  Higher   1986 0.048   1976 0.033
====================================================================================================================================
NG1181    1963 to  2021      59 years                                                                                  Series 116
[A] Segment   High   -10   -9   -8   -7   -6   -5   -4   -3   -2   -1    0   +1   +2   +3   +4   +5   +6   +7   +8   +9  +10
---------  ----   ---  ---  ---  ---  ---  ---  ---  ---  ---  ---  ---  ---  ---  ---  ---  ---  ---  ---  ---  ---  ---
1963 2012    0   -.25  .00 -.04  .13  .03 -.06  .08  .21 -.06 -.15  .25* .11 -.05  .03  .11 -.13 -.02 -.23 -.05 -.07 -.02
1972 2021   -5   -.03  .00  .08 -.01  .01  .19* .02  .07 -.17 -.11  .17| .12    -    -    -    -    -    -    -    -    -    -
[B] Entire series, effect on correlation ( 0.185) is:
Lower   2021<-0.092   1996>-0.040   2011>-0.031   1999<-0.020   2003<-0.019   1995>-0.015  Higher   1976 0.088   1994 0.016
1963 to 2012 segment:
Lower   1996>-0.056   2011>-0.044   1999<-0.030   2003<-0.029   1995>-0.020   2001<-0.020  Higher   1976 0.120   1986 0.020
1972 to 2021 segment:
Lower   2021<-0.123   1996>-0.044   2011>-0.034   1999<-0.023   2003<-0.023   1995>-0.016  Higher   1976 0.105   1994 0.021
====================================================================================================================================
NG1182    1963 to  2021      59 years                                                                                  Series 117
[A] Segment   High   -10   -9   -8   -7   -6   -5   -4   -3   -2   -1    0   +1   +2   +3   +4   +5   +6   +7   +8   +9  +10
---------  ----   ---  ---  ---  ---  ---  ---  ---  ---  ---  ---  ---  ---  ---  ---  ---  ---  ---  ---  ---  ---  ---
1963 2012   -4   -.14 -.22 -.07 -.02 -.04 -.02  .28* .20 -.03  .10  .27|-.18 -.04 -.02  .06 -.13  .21 -.05 -.24  .06 -.15
1972 2021    0   -.03 -.24  .03 -.10 -.07  .06  .19  .01 -.06  .14  .28*-.14    -    -    -    -    -    -    -    -    -    -
[B] Entire series, effect on correlation ( 0.291) is:
Lower   1976>-0.048   1995>-0.037   1996>-0.024   1984<-0.022   1988<-0.019   2008<-0.014  Higher   1980 0.052   2007 0.032
1963 to 2012 segment:
Lower   1976>-0.057   1995>-0.043   1996>-0.029   1984<-0.023   1988<-0.020   2008<-0.015  Higher   1980 0.065   2007 0.039
1972 to 2021 segment:
Lower   1976>-0.052   1995>-0.043   1996>-0.027   1984<-0.027   1988<-0.022   2008<-0.016  Higher   1980 0.054   1994 0.037
====================================================================================================================================
NG1183    1968 to  2021      54 years                                                                                  Series 118
[A] Segment   High   -10   -9   -8   -7   -6   -5   -4   -3   -2   -1    0   +1   +2   +3   +4   +5   +6   +7   +8   +9  +10
---------  ----   ---  ---  ---  ---  ---  ---  ---  ---  ---  ---  ---  ---  ---  ---  ---  ---  ---  ---  ---  ---  ---
1972 2021    0    .13 -.23 -.17 -.13  .11  .13  .23 -.06 -.21  .25  .33*-.02    -    -    -    -    -    -    -    -    -    -
[B] Entire series, effect on correlation ( 0.331) is:
Lower   2001<-0.035   1986>-0.033   1995>-0.029   2011>-0.025   2004>-0.016   2021<-0.015  Higher   1980 0.045   1976 0.044
1972 to 2021 segment:
Lower   2001<-0.036   1986>-0.034   1995>-0.030   2011>-0.026   2004>-0.017   2021<-0.015  Higher   1980 0.045   1976 0.044
====================================================================================================================================
NG1190    1964 to  2021      58 years                                                                                  Series 119
[B] Entire series, effect on correlation ( 0.525) is:
Lower   2005<-0.044   2004>-0.028   1976>-0.028   1965<-0.028   1990>-0.013   1966<-0.012  Higher   2016 0.029   1994 0.022
====================================================================================================================================
NG1191    1957 to  2021      65 years                                                                                  Series 120
[B] Entire series, effect on correlation ( 0.436) is:
Lower   1965<-0.026   2009<-0.026   2004>-0.023   1973<-0.020   1961<-0.016   2018<-0.013  Higher   1976 0.068   1986 0.019
====================================================================================================================================
NG1280    1971 to  2021      51 years                                                                                  Series 121
[A] Segment   High   -10   -9   -8   -7   -6   -5   -4   -3   -2   -1    0   +1   +2   +3   +4   +5   +6   +7   +8   +9  +10
---------  ----   ---  ---  ---  ---  ---  ---  ---  ---  ---  ---  ---  ---  ---  ---  ---  ---  ---  ---  ---  ---  ---
1971 2020   -4   -.03 -.07 -.20 -.11 -.06  .09  .40*-.11 -.10  .02  .32|-.10 -.17    -    -    -    -    -    -    -    -    -
1972 2021   -4   -.07 -.08 -.20 -.11 -.06  .03  .39*-.14 -.08  .03  .31|-.10    -    -    -    -    -    -    -    -    -    -
[B] Entire series, effect on correlation ( 0.322) is:
Lower   1986>-0.052   2016>-0.041   1976>-0.034   2008<-0.021   2001<-0.019   2002>-0.015  Higher   1980 0.037   2019 0.023
1971 to 2020 segment:
Lower   1986>-0.053   2016>-0.042   1976>-0.034   2008<-0.021   2001<-0.019   2004>-0.016  Higher   1980 0.038   2019 0.024
1972 to 2021 segment:
Lower   1986>-0.054   2016>-0.043   1976>-0.035   2008<-0.021   2001<-0.020   2004>-0.016  Higher   1980 0.038   2019 0.025
[E] Outliers     1   3.0 SD above or -4.5 SD below mean for year
1980 -4.7 SD
====================================================================================================================================
NG1281    1972 to  2021      50 years                                                                                  Series 122
```

```
[A] Segment   High   -10   -9   -8   -7   -6   -5   -4   -3   -2   -1    0   +1   +2   +3   +4   +5   +6   +7   +8   +9  +10
---------  ----   ---  ---  ---  ---  ---  ---  ---  ---  ---  ---  ---  ---  ---  ---  ---  ---  ---  ---  ---  ---  ---
1972 2021   -4   -.06 -.16  .00  .04  .02 -.07  .35* .03 -.10 -.02  .26| .00    -    -    -    -    -    -    -    -    -
[B] Entire series, effect on correlation ( 0.258) is:
Lower   2016>-0.066   1986>-0.046   1996>-0.022   1994<-0.015   1993<-0.013   2011>-0.013  Higher   1980 0.062   2007 0.034
1972 to 2021 segment:
Lower   2016>-0.066   1986>-0.046   1996>-0.022   1994<-0.015   1993<-0.013   2011>-0.013  Higher   1980 0.062   2007 0.034
[E] Outliers     1   3.0 SD above or -4.5 SD below mean for year
1980 -4.7 SD
==================================================================================================================================
NG1282    1962 to  2022      61 years                                                                                  Series 123
[A] Segment   High   -10   -9   -8   -7   -6   -5   -4   -3   -2   -1    0   +1   +2   +3   +4   +5   +6   +7   +8   +9  +10
---------  ----   ---  ---  ---  ---  ---  ---  ---  ---  ---  ---  ---  ---  ---  ---  ---  ---  ---  ---  ---  ---  ---
1962 2011    0   -.19 -.02 -.14  .18  .11 -.12  .07  .24  .12 -.03  .27*-.16 -.02 -.10  .12 -.13 -.14 -.07 -.07  .19 -.16
1973 2022    0   -.14 -.05  .00  .23  .05 -.05 -.01  .05  .17  .05  .27*   -    -    -    -    -    -    -    -    -    -
[B] Entire series, effect on correlation ( 0.272) is:
Lower   1995>-0.038   1982<-0.033   2011>-0.019   1976>-0.017   2016>-0.016   1999<-0.016  Higher   2007 0.042   2004 0.027
1962 to 2011 segment:
Lower   1995>-0.045   1982<-0.037   1976>-0.023   2011>-0.023   1999<-0.017   2008<-0.015  Higher   2007 0.051   2004 0.030
1973 to 2022 segment:
Lower   1995>-0.044   1982<-0.043   2011>-0.021   1999<-0.020   2008<-0.017   2016>-0.016  Higher   2007 0.037   2004 0.032
[E] Outliers     1   3.0 SD above or -4.5 SD below mean for year
1963 -5.1 SD
==================================================================================================================================
NG1283    1976 to  2022      47 years                                                                                  Series 124
[B] Entire series, effect on correlation ( 0.333) is:
Lower   1984<-0.084   1976>-0.076   1995>-0.036   2005<-0.023   2015<-0.009   1979>-0.007  Higher   2007 0.030   1986 0.028
==================================================================================================================================
NG1290    1961 to  2022      62 years                                                                                  Series 125
[B] Entire series, effect on correlation ( 0.551) is:
Lower   1965<-0.107   1976>-0.040   1987<-0.017   1990>-0.011   1967<-0.011   2013>-0.011  Higher   1996 0.041   2016 0.035
==================================================================================================================================
NG1291    1965 to  2022      58 years                                                                                  Series 126
[B] Entire series, effect on correlation ( 0.519) is:
Lower   1976>-0.042   1965<-0.031   2000>-0.030   1991<-0.028   1993<-0.013   1990>-0.011  Higher   1986 0.046   2004 0.022
==================================================================================================================================
NG1320    1964 to  2021      58 years                                                                                  Series 127
[B] Entire series, effect on correlation ( 0.539) is:
Lower   1969<-0.041   2008<-0.033   1964<-0.026   1995>-0.020   2011>-0.015   2013>-0.012  Higher   1976 0.069   1986 0.048
==================================================================================================================================
NG1321    1966 to  2021      56 years                                                                                  Series 128
[A] Segment   High   -10   -9   -8   -7   -6   -5   -4   -3   -2   -1    0   +1   +2   +3   +4   +5   +6   +7   +8   +9  +10
---------  ----   ---  ---  ---  ---  ---  ---  ---  ---  ---  ---  ---  ---  ---  ---  ---  ---  ---  ---  ---  ---  ---
1972 2021  -10    .40*-.15 -.02 -.19  .14  .01 -.04 -.16 -.19  .15  .23| .06    -    -    -    -    -    -    -    -    -
[B] Entire series, effect on correlation ( 0.219) is:
Lower   2021<-0.106   2011>-0.034   2004>-0.022   1971<-0.016   1997<-0.016   2016>-0.016  Higher   1996 0.050   1976 0.046
1972 to 2021 segment:
Lower   2021<-0.126   2011>-0.035   2004>-0.022   1997<-0.018   2016>-0.016   1999<-0.015  Higher   1996 0.051   1976 0.048
==================================================================================================================================
NG1400    1968 to  2022      55 years                                                                                  Series 129
[A] Segment   High   -10   -9   -8   -7   -6   -5   -4   -3   -2   -1    0   +1   +2   +3   +4   +5   +6   +7   +8   +9  +10
---------  ----   ---  ---  ---  ---  ---  ---  ---  ---  ---  ---  ---  ---  ---  ---  ---  ---  ---  ---  ---  ---  ---
1968 2017   -1    .20  .07 -.24  .01  .06  .15  .10 -.12 -.14  .29* .18|-.13 -.15 -.12 -.11  .27    -    -    -    -    -
1973 2022   -1    .22  .06 -.18  .00  .01  .16  .10 -.10 -.18  .29* .17|   -    -    -    -    -    -    -    -    -    -
[B] Entire series, effect on correlation ( 0.168) is:
Lower   1976>-0.103   1981<-0.030   2016>-0.025   1986>-0.023   1993<-0.022   2000>-0.017  Higher   1996 0.039   2011 0.037
1968 to 2017 segment:
Lower   1976>-0.113   1981<-0.034   2016>-0.028   1986>-0.026   1993<-0.023   2000>-0.019  Higher   1996 0.038   2011 0.037
1973 to 2022 segment:
Lower   1976>-0.106   1981<-0.032   2016>-0.026   1986>-0.023   1993<-0.023   2000>-0.017  Higher   1996 0.041   2011 0.038
[E] Outliers     1   3.0 SD above or -4.5 SD below mean for year
1976 +3.3 SD
==================================================================================================================================
NG1401    1967 to  2022      56 years                                                                                  Series 130
[A] Segment   High   -10   -9   -8   -7   -6   -5   -4   -3   -2   -1    0   +1   +2   +3   +4   +5   +6   +7   +8   +9  +10
---------  ----   ---  ---  ---  ---  ---  ---  ---  ---  ---  ---  ---  ---  ---  ---  ---  ---  ---  ---  ---  ---  ---
1967 2016   -5   -.04  .22 -.15 -.13 -.09  .43* .03 -.10 -.01  .04  .36|-.05 -.03 -.23 -.02  .19  .02    -    -    -    -
[B] Entire series, effect on correlation ( 0.372) is:
Lower   1981<-0.156   1976>-0.072   1986>-0.029   2011>-0.012   1975<-0.010   1972>-0.008  Higher   2016 0.064   2004 0.032
1967 to 2016 segment:
Lower   1981<-0.162   1976>-0.076   1986>-0.031   2011>-0.012   1975<-0.010   1972>-0.008  Higher   2016 0.067   2004 0.033
[C] Year-to-year changes diverging by over 4.0 std deviations:
1980 1981  -4.1 SD
[E] Outliers     1   3.0 SD above or -4.5 SD below mean for year
1981 -5.4 SD
==================================================================================================================================
NG1420    1968 to  2022      55 years                                                                                  Series 131
[A] Segment   High   -10   -9   -8   -7   -6   -5   -4   -3   -2   -1    0   +1   +2   +3   +4   +5   +6   +7   +8   +9  +10
---------  ----   ---  ---  ---  ---  ---  ---  ---  ---  ---  ---  ---  ---  ---  ---  ---  ---  ---  ---  ---  ---  ---
1968 2017    0    .19 -.10 -.18  .02  .14 -.01  .18 -.13 -.09  .18  .32*-.04 -.26 -.07  .17  .10    -    -    -    -    -
1973 2022    0    .23 -.09 -.08 -.03  .10  .06  .16 -.14 -.19  .18  .30*   -    -    -    -    -    -    -    -    -    -
[B] Entire series, effect on correlation ( 0.307) is:
Lower   2016>-0.051   2012<-0.046   1976>-0.035   2004>-0.034   2011>-0.023   2018>-0.016  Higher   1980 0.049   2000 0.026
1968 to 2017 segment:
Lower   2016>-0.057   2012<-0.050   1976>-0.042   2004>-0.038   2011>-0.025   1997<-0.016  Higher   1980 0.046   2000 0.026
1973 to 2022 segment:
Lower   2016>-0.054   2012<-0.049   2004>-0.036   1976>-0.035   2011>-0.024   2018>-0.018  Higher   1980 0.051   2000 0.027
==================================================================================================================================
NG1421    1969 to  2022      54 years                                                                                  Series 132
[B] Entire series, effect on correlation ( 0.494) is:
Lower   2011>-0.032   2008<-0.022   1976>-0.019   2014<-0.016   1990>-0.015   2007>-0.014  Higher   1996 0.057   2016 0.023
==================================================================================================================================
```

```
NG1422    1969 to  2022      54 years                                                                                    Series 133
[B] Entire series, effect on correlation ( 0.411) is:
Lower   1976>-0.050   2001<-0.043   1995>-0.024   2016>-0.023   1969<-0.017   1970>-0.016  Higher   1996 0.029   1980 0.027
====================================================================================================================================
NG1423    1971 to  2022      52 years                                                                                    Series 134
[B] Entire series, effect on correlation ( 0.372) is:
Lower   2014<-0.093   2016>-0.053   2008<-0.026   2013>-0.021   1997<-0.019   1974>-0.013  Higher   1996 0.060   1986 0.026
====================================================================================================================================
NG1430    1968 to  2022      55 years                                                                                    Series 135
[B] Entire series, effect on correlation ( 0.487) is:
Lower   1980>-0.034   1972<-0.031   2000>-0.028   2011>-0.027   1994<-0.021   1984<-0.019  Higher   2016 0.061   1976 0.032
====================================================================================================================================
NG1431    1958 to  2022      65 years                                                                                    Series 136
[A] Segment   High   -10   -9   -8   -7   -6   -5   -4   -3   -2   -1    0   +1   +2   +3   +4   +5   +6   +7   +8   +9  +10
---------  ----   ---  ---  ---  ---  ---  ---  ---  ---  ---  ---  ---  ---  ---  ---  ---  ---  ---  ---  ---  ---  ---
1958 2007    0    .15  .14 -.21  .08  .09  .23 -.13 -.17  .03 -.03  .28* .04  .13 -.19  .13 -.07 -.10 -.19 -.23  .05 -.02
[B] Entire series, effect on correlation ( 0.325) is:
Lower   1958<-0.040   1965<-0.031   1960>-0.030   2004>-0.024   1980>-0.016   1959>-0.016  Higher   1996 0.049   2011 0.029
1958 to 2007 segment:
Lower   1958<-0.049   1965<-0.039   1960>-0.036   2004>-0.029   1980>-0.019   1959>-0.019  Higher   1996 0.066   2007 0.033
====================================================================================================================================
NG1440    1959 to  2022      64 years                                                                                    Series 137
[A] Segment   High   -10   -9   -8   -7   -6   -5   -4   -3   -2   -1    0   +1   +2   +3   +4   +5   +6   +7   +8   +9  +10
---------  ----   ---  ---  ---  ---  ---  ---  ---  ---  ---  ---  ---  ---  ---  ---  ---  ---  ---  ---  ---  ---  ---
1959 2008   -9   -.05  .33*-.27  .03 -.01  .03  .02  .05 -.04 -.19  .33| .20 -.14 -.15 -.02  .00  .08 -.11 -.27 -.14  .09
1973 2022   -9    .02  .33*-.18  .00  .12  .06  .01 -.04 -.09 -.09  .24|  -    -    -    -    -    -    -    -    -    -
[B] Entire series, effect on correlation ( 0.276) is:
Lower   2011>-0.041   2004>-0.035   1996>-0.035   1999<-0.034   1992<-0.029   2016>-0.025  Higher   1986 0.035   1988 0.025
1959 to 2008 segment:
Lower   1996>-0.047   2004>-0.046   1985<-0.043   1999<-0.041   1992<-0.036   1960>-0.011  Higher   1986 0.037   1988 0.028
1973 to 2022 segment:
Lower   2011>-0.046   2004>-0.040   1996>-0.039   1999<-0.037   1992<-0.032   2016>-0.028  Higher   1986 0.041   1988 0.031
====================================================================================================================================
NG1441    1968 to  2022      55 years                                                                                    Series 138
[A] Segment   High   -10   -9   -8   -7   -6   -5   -4   -3   -2   -1    0   +1   +2   +3   +4   +5   +6   +7   +8   +9  +10
---------  ----   ---  ---  ---  ---  ---  ---  ---  ---  ---  ---  ---  ---  ---  ---  ---  ---  ---  ---  ---  ---  ---
1968 2017    0    .16  .27  .02 -.27  .17 -.20  .30  .05 -.10 -.22  .32* .06 -.23  .02 -.19 -.13   -    -    -    -    -
1973 2022    0    .16  .22  .02 -.26  .17 -.20  .26  .02 -.10 -.18  .32*  -    -    -    -    -    -    -    -    -    -
[B] Entire series, effect on correlation ( 0.322) is:
Lower   1976>-0.037   2010<-0.034   2011>-0.028   2000>-0.020   1986>-0.020   2007>-0.019  Higher   1980 0.046   1995 0.041
1968 to 2017 segment:
Lower   1976>-0.042   2010<-0.035   2011>-0.030   1986>-0.023   2000>-0.022   2007>-0.021  Higher   1980 0.048   1995 0.042
1973 to 2022 segment:
Lower   2010<-0.037   1976>-0.037   2011>-0.028   2000>-0.020   1986>-0.019   2007>-0.019  Higher   1980 0.046   1995 0.041
====================================================================================================================================
NG1470    1975 to  2022      48 years                                                                                    Series 139
[A] Segment   High   -10   -9   -8   -7   -6   -5   -4   -3   -2   -1    0   +1   +2   +3   +4   +5   +6   +7   +8   +9  +10
---------  ----   ---  ---  ---  ---  ---  ---  ---  ---  ---  ---  ---  ---  ---  ---  ---  ---  ---  ---  ---  ---  ---
1975 2022   -5    .12  .07 -.02 -.31 -.05  .28*-.06  .10 -.25  .13  .16|  -    -    -    -    -    -    -    -    -    -
[B] Entire series, effect on correlation ( 0.158) is:
Lower   2001<-0.056   2021<-0.046   2008<-0.043   1976>-0.043   1988<-0.039   2011>-0.027  Higher   2016 0.086   1994 0.043
1975 to 2022 segment:
Lower   2001<-0.056   2021<-0.046   2008<-0.043   1976>-0.043   1988<-0.039   2011>-0.027  Higher   2016 0.086   1994 0.043
====================================================================================================================================
NG1471    1966 to  2022      57 years                                                                                    Series 140
[A] Segment   High   -10   -9   -8   -7   -6   -5   -4   -3   -2   -1    0   +1   +2   +3   +4   +5   +6   +7   +8   +9  +10
---------  ----   ---  ---  ---  ---  ---  ---  ---  ---  ---  ---  ---  ---  ---  ---  ---  ---  ---  ---  ---  ---  ---
1966 2015    1    .00  .22 -.15 -.01  .24  .05 -.10 -.19 -.02 -.06  .24| .28*-.12 -.12  .12  .14 -.10 -.26   -    -    -
1973 2022    0    .06  .24 -.13 -.09  .16  .12 -.14 -.22 -.11 -.05  .28*  -    -    -    -    -    -    -    -    -    -
[B] Entire series, effect on correlation ( 0.268) is:
Lower   1975<-0.082   1985<-0.023   1976>-0.021   1966<-0.020   2011>-0.018   2004>-0.013  Higher   1994 0.042   2016 0.037
1966 to 2015 segment:
Lower   1975<-0.088   1976>-0.023   2011>-0.021   1966<-0.021   1985<-0.016   2004>-0.014  Higher   1994 0.048   1996 0.035
1973 to 2022 segment:
Lower   1975<-0.091   1985<-0.035   1976>-0.025   2011>-0.020   2004>-0.014   2021<-0.012  Higher   1994 0.045   2016 0.038
[C] Year-to-year changes diverging by over 4.0 std deviations:
1975 1976   4.1 SD
====================================================================================================================================
NG151     1968 to  2017      50 years                                                                                    Series 141
[A] Segment   High   -10   -9   -8   -7   -6   -5   -4   -3   -2   -1    0   +1   +2   +3   +4   +5   +6   +7   +8   +9  +10
---------  ----   ---  ---  ---  ---  ---  ---  ---  ---  ---  ---  ---  ---  ---  ---  ---  ---  ---  ---  ---  ---  ---
1968 2017    5    .08  .03 -.09 -.25  .15 -.03 -.06 -.27  .14 -.01  .20| .10 -.19 -.02  .08  .31*  -    -    -    -    -
[B] Entire series, effect on correlation ( 0.202) is:
Lower   2015<-0.079   1971<-0.049   2016>-0.032   1997<-0.028   2004>-0.025   1982<-0.021  Higher   1996 0.071   1986 0.053
1968 to 2017 segment:
Lower   2015<-0.079   1971<-0.049   2016>-0.032   1997<-0.028   2004>-0.025   1982<-0.021  Higher   1996 0.071   1986 0.053
====================================================================================================================================
NG1580    1962 to  2021      60 years                                                                                    Series 142
[A] Segment   High   -10   -9   -8   -7   -6   -5   -4   -3   -2   -1    0   +1   +2   +3   +4   +5   +6   +7   +8   +9  +10
---------  ----   ---  ---  ---  ---  ---  ---  ---  ---  ---  ---  ---  ---  ---  ---  ---  ---  ---  ---  ---  ---  ---
1962 2011   -6    .18  .21 -.20 -.21  .25* .15 -.18 -.25 -.11 -.10  .16| .10 -.09 -.26  .15  .15  .04 -.11 -.08 -.13  .12
1972 2021    0    .22  .26 -.22 -.22  .22  .26 -.10 -.25 -.22 -.10  .27* .09   -    -    -    -    -    -    -    -    -
[B] Entire series, effect on correlation ( 0.269) is:
Lower   1976>-0.075   2004>-0.031   2011>-0.026   2021<-0.023   1980>-0.016   1991<-0.012  Higher   2016 0.087   1996 0.080
1962 to 2011 segment:
Lower   1976>-0.072   2004>-0.033   2011>-0.028   1980>-0.016   1991<-0.013   1971<-0.013  Higher   1996 0.128   1986 0.027
1972 to 2021 segment:
Lower   1976>-0.078   2004>-0.031   2021<-0.027   2011>-0.026   1980>-0.017   1991<-0.016  Higher   2016 0.091   1996 0.083
====================================================================================================================================
NG1581    1967 to  2021      55 years                                                                                    Series 143
[A] Segment   High   -10   -9   -8   -7   -6   -5   -4   -3   -2   -1    0   +1   +2   +3   +4   +5   +6   +7   +8   +9  +10
---------  ----   ---  ---  ---  ---  ---  ---  ---  ---  ---  ---  ---  ---  ---  ---  ---  ---  ---  ---  ---  ---  ---
```

```
1967 2016   -9    .05  .34*-.05 -.15  .07  .29 -.09 -.38 -.16 -.16  .21| .09 -.02 -.32  .14  .20  .04    -    -    -    -
1972 2021   -9    .11  .35*-.06 -.17  .09  .34 -.04 -.36 -.24 -.17  .20| .15    -    -    -    -    -    -    -    -    -    -
[B] Entire series, effect on correlation ( 0.189) is:
Lower   2004>-0.032   1991<-0.022   1968<-0.019   2021<-0.018   2018>-0.014   1993<-0.014  Higher   1996 0.041   1995 0.023
1967 to 2016 segment:
Lower   2004>-0.035   1991<-0.026   1968<-0.021   1993<-0.015   1990>-0.013   1979>-0.011  Higher   1996 0.042   1995 0.024
1972 to 2021 segment:
Lower   2004>-0.033   1991<-0.029   2021<-0.022   1993<-0.016   2018>-0.015   1990>-0.012  Higher   1996 0.044   1995 0.025
=========================================================================================================================
NG2640    1958 to  2021     64 years                                                                            Series 144
[B] Entire series, effect on correlation ( 0.409) is:
Lower   2008<-0.036   1976>-0.025   2000>-0.024   1986>-0.021   2018>-0.014   2004>-0.011  Higher   1960 0.041   2007 0.021
[E] Outliers     1   3.0 SD above or -4.5 SD below mean for year
1960 -4.8 SD
=========================================================================================================================
NG2641    1959 to  2021     63 years                                                                            Series 145
[B] Entire series, effect on correlation ( 0.443) is:
Lower   1976>-0.062   1997<-0.039   1986>-0.039   2000>-0.020   2018>-0.015   1959>-0.009  Higher   1960 0.031   2004 0.020
=========================================================================================================================
NG2650    1952 to  2021     70 years                                                                            Series 146
[A] Segment   High   -10   -9   -8   -7   -6   -5   -4   -3   -2   -1    0   +1   +2   +3   +4   +5   +6   +7   +8   +9  +10
---------  ----   ---  ---  ---  ---  ---  ---  ---  ---  ---  ---  ---  ---  ---  ---  ---  ---  ---  ---  ---  ---  ---
1972 2021   -4   -.04  .05  .16 -.13 -.13 -.16  .28*-.11  .02 -.10  .25| .19    -    -    -    -    -    -    -    -    -    -
[B] Entire series, effect on correlation ( 0.198) is:
Lower   2015<-0.082   1953<-0.045   2011>-0.027   2016>-0.024   2007>-0.021   2003<-0.019  Higher   1980 0.042   1976 0.034
1972 to 2021 segment:
Lower   2015<-0.138   2011>-0.035   2016>-0.033   2007>-0.027   2003<-0.026   1995>-0.019  Higher   1980 0.047   1976 0.037
=========================================================================================================================
NG2651    1962 to  2021     60 years                                                                            Series 147
[B] Entire series, effect on correlation ( 0.401) is:
Lower   2016>-0.046   2013<-0.040   2012<-0.035   2007>-0.027   1995>-0.016   2011>-0.011  Higher   1976 0.042   1980 0.035
[E] Outliers     1   3.0 SD above or -4.5 SD below mean for year
2013 -5.9 SD
=========================================================================================================================
NG2710    1968 to  2021     54 years                                                                            Series 148
[B] Entire series, effect on correlation ( 0.344) is:
Lower   1995>-0.048   2019<-0.035   2011>-0.026   2000>-0.021   2002>-0.012   2010<-0.007  Higher   2004 0.048   2007 0.022
=========================================================================================================================
NG2711    1967 to  2021     55 years                                                                            Series 149
[B] Entire series, effect on correlation ( 0.397) is:
Lower   2014<-0.058   1976>-0.043   1993<-0.026   1986>-0.021   1968<-0.017   2018>-0.016  Higher   1996 0.041   2004 0.031
=========================================================================================================================
NG2712    1970 to  2021     52 years                                                                            Series 150
[B] Entire series, effect on correlation ( 0.336) is:
Lower   1976>-0.045   2011>-0.037   2019<-0.037   2004>-0.033   2021<-0.019   2018>-0.017  Higher   1996 0.065   1980 0.043
=========================================================================================================================
NG2713    1965 to  2021     57 years                                                                            Series 151
[B] Entire series, effect on correlation ( 0.478) is:
Lower   1976>-0.058   2004>-0.036   2008<-0.028   1988<-0.017   2000>-0.017   1995>-0.013  Higher   1996 0.059   2016 0.058
=========================================================================================================================
NG2800    1967 to  2021     55 years                                                                            Series 152
[A] Segment   High   -10   -9   -8   -7   -6   -5   -4   -3   -2   -1    0   +1   +2   +3   +4   +5   +6   +7   +8   +9  +10
---------  ----   ---  ---  ---  ---  ---  ---  ---  ---  ---  ---  ---  ---  ---  ---  ---  ---  ---  ---  ---  ---  ---
1972 2021   -4   -.04  .23 -.11 -.01 -.01 -.11  .35* .03  .11 -.37  .34|-.07    -    -    -    -    -    -    -    -    -    -
[B] Entire series, effect on correlation ( 0.321) is:
Lower   1976>-0.046   2004>-0.026   1969<-0.024   1996>-0.023   2017>-0.021   2009<-0.016  Higher   1980 0.047   2011 0.031
1972 to 2021 segment:
Lower   1976>-0.051   2004>-0.029   1996>-0.025   2017>-0.024   2009<-0.018   1990>-0.011  Higher   1980 0.039   2011 0.030
=========================================================================================================================
NG2801    1958 to  2021     64 years                                                                            Series 153
[A] Segment   High   -10   -9   -8   -7   -6   -5   -4   -3   -2   -1    0   +1   +2   +3   +4   +5   +6   +7   +8   +9  +10
---------  ----   ---  ---  ---  ---  ---  ---  ---  ---  ---  ---  ---  ---  ---  ---  ---  ---  ---  ---  ---  ---  ---
1972 2021   -4   -.01  .16 -.12  .09  .00 -.11  .34* .05  .05 -.20  .34|-.20    -    -    -    -    -    -    -    -    -    -
[B] Entire series, effect on correlation ( 0.394) is:
Lower   1973<-0.039   2020<-0.032   1995>-0.020   2016>-0.017   1988<-0.016   1996>-0.014  Higher   1960 0.052   1980 0.027
1972 to 2021 segment:
Lower   1973<-0.049   2020<-0.043   1995>-0.025   1988<-0.018   2016>-0.018   1984<-0.016  Higher   1980 0.036   2011 0.033
=========================================================================================================================
NG2900    1948 to  2021     74 years                                                                            Series 154
[A] Segment   High   -10   -9   -8   -7   -6   -5   -4   -3   -2   -1    0   +1   +2   +3   +4   +5   +6   +7   +8   +9  +10
---------  ----   ---  ---  ---  ---  ---  ---  ---  ---  ---  ---  ---  ---  ---  ---  ---  ---  ---  ---  ---  ---  ---
1948 1997   -8    .11 -.04  .32*-.05  .06 -.04  .01  .06 -.08 -.09  .13| .00 -.09 -.21  .02 -.02 -.12  .08  .04  .09 -.07
1950 1999   -8    .13 -.06  .34*-.06  .10 -.11  .12  .04 -.08 -.15  .28| .00 -.12 -.24  .02 -.06 -.13  .03  .06  .11 -.08
1972 2021   -9    .21  .44* .02 -.11  .02  .08 -.08 -.01 -.10 -.05  .35| .10    -    -    -    -    -    -    -    -    -    -
[B] Entire series, effect on correlation ( 0.188) is:
Lower   1948>-0.106   1952<-0.046   1980>-0.018   1988<-0.016   2018>-0.015   1962<-0.013  Higher   1996 0.039   2016 0.036
1948 to 1997 segment:
Lower   1948>-0.139   1952<-0.054   1980>-0.023   1988<-0.019   1962<-0.016   1971<-0.012  Higher   1996 0.053   1960 0.039
1950 to 1999 segment:
Lower   1952<-0.070   1980>-0.033   1976>-0.030   1988<-0.023   1962<-0.018   1957>-0.014  Higher   1996 0.054   1960 0.040
1972 to 2021 segment:
Lower   1988<-0.036   1980>-0.031   2018>-0.029   2015<-0.025   1984<-0.023   1989<-0.021  Higher   1996 0.056   2016 0.053
[E] Outliers     2   3.0 SD above or -4.5 SD below mean for year
1948 +3.9 SD;    1961 +3.1 SD
=========================================================================================================================
NG2901    1960 to  2021     62 years                                                                            Series 155
[B] Entire series, effect on correlation ( 0.293) is:
Lower   1967<-0.041   1988<-0.038   1960>-0.023   1984<-0.022   2004>-0.019   2015<-0.017  Higher   1976 0.053   1996 0.037
=========================================================================================================================
NG2940    1957 to  2021     65 years                                                                            Series 156
[A] Segment   High   -10   -9   -8   -7   -6   -5   -4   -3   -2   -1    0   +1   +2   +3   +4   +5   +6   +7   +8   +9  +10
---------  ----   ---  ---  ---  ---  ---  ---  ---  ---  ---  ---  ---  ---  ---  ---  ---  ---  ---  ---  ---  ---  ---
```

```
1957 2006    1    .12  .14  .02 -.04  .24  .05 -.08 -.09  .20 -.18  .21| .27*-.04  .02  .11  .01 -.25  .05 -.22 -.02  .06
1972 2021    0   -.01  .04 -.07  .00  .17 -.04  .00  .19  .05 -.09  .24* .05    -    -    -    -    -    -    -    -    -
[B] Entire series, effect on correlation ( 0.169) is:
Lower   1960>-0.046   2016>-0.041   1982<-0.024   1965<-0.023   1996>-0.018   2018>-0.017  Higher   1976 0.113   2007 0.060
1957 to 2006 segment:
Lower   1960>-0.075   1965<-0.044   1982<-0.042   1996>-0.027   1958<-0.024   1975<-0.024  Higher   1976 0.191   1999 0.027
1972 to 2021 segment:
Lower   2016>-0.054   1982<-0.029   1996>-0.026   2018>-0.022   1995>-0.018   1975<-0.017  Higher   1976 0.120   2007 0.045
[E] Outliers     1   3.0 SD above or -4.5 SD below mean for year
2007 -4.5 SD
======================================================================================================================================
NG2941    1957 to  2020      64 years                                                                                    Series 157
[A] Segment   High   -10   -9   -8   -7   -6   -5   -4   -3   -2   -1    0   +1   +2   +3   +4   +5   +6   +7   +8   +9  +10
---------  ----   ---  ---  ---  ---  ---  ---  ---  ---  ---  ---  ---  ---  ---  ---  ---  ---  ---  ---  ---  ---  ---
1971 2020    0   -.02  .02 -.05 -.16  .12  .12  .20 -.09 -.09  .10  .28*-.02 -.12    -    -    -    -    -    -    -    -
[B] Entire series, effect on correlation ( 0.253) is:
Lower   2008<-0.165   1960>-0.045   1982<-0.023   1990>-0.016   1975<-0.014   2020<-0.010  Higher   1976 0.035   1986 0.029
1971 to 2020 segment:
Lower   2008<-0.205   1982<-0.026   1990>-0.020   1975<-0.016   2020<-0.013   2017>-0.010  Higher   1976 0.035   1986 0.031
[C] Year-to-year changes diverging by over 4.0 std deviations:
2007 2008  -5.2 SD
[E] Outliers     1   3.0 SD above or -4.5 SD below mean for year
2008 -5.3 SD
======================================================================================================================================
NG3020    1955 to  2021      67 years                                                                                    Series 158
[B] Entire series, effect on correlation ( 0.420) is:
Lower   1955<-0.046   2016>-0.037   1976>-0.031   2011>-0.020   2019<-0.014   2001<-0.008  Higher   2004 0.019   1996 0.017
[E] Outliers     1   3.0 SD above or -4.5 SD below mean for year
1980 -5.4 SD
======================================================================================================================================
NG3021    1951 to  2021      71 years                                                                                    Series 159
[B] Entire series, effect on correlation ( 0.370) is:
Lower   2016>-0.037   1976>-0.036   1995>-0.022   1964<-0.018   1953<-0.016   1991<-0.015  Higher   1980 0.032   2004 0.022
======================================================================================================================================
NG3040    1960 to  2021      62 years                                                                                    Series 160
[A] Segment   High   -10   -9   -8   -7   -6   -5   -4   -3   -2   -1    0   +1   +2   +3   +4   +5   +6   +7   +8   +9  +10
---------  ----   ---  ---  ---  ---  ---  ---  ---  ---  ---  ---  ---  ---  ---  ---  ---  ---  ---  ---  ---  ---  ---
1972 2021    0   -.02 -.05 -.19 -.08  .01  .01 -.11  .08  .06  .15  .19*-.14    -    -    -    -    -    -    -    -    -
[B] Entire series, effect on correlation ( 0.230) is:
Lower   2019<-0.105   1976>-0.047   1986>-0.035   2008<-0.018   2011>-0.015   2016>-0.013  Higher   1996 0.057   2007 0.046
1972 to 2021 segment:
Lower   2019<-0.113   1976>-0.051   1986>-0.039   2008<-0.018   2011>-0.017   2016>-0.015  Higher   1996 0.064   2007 0.058
[C] Year-to-year changes diverging by over 4.0 std deviations:
2018 2019  -4.4 SD
======================================================================================================================================
NG3041    1972 to  2021      50 years                                                                                    Series 161
[A] Segment   High   -10   -9   -8   -7   -6   -5   -4   -3   -2   -1    0   +1   +2   +3   +4   +5   +6   +7   +8   +9  +10
---------  ----   ---  ---  ---  ---  ---  ---  ---  ---  ---  ---  ---  ---  ---  ---  ---  ---  ---  ---  ---  ---  ---
1972 2021   -1    .07 -.05  .10  .10 -.08  .19 -.24 -.08 -.06  .22* .17| .07    -    -    -    -    -    -    -    -    -
[B] Entire series, effect on correlation ( 0.173) is:
Lower   2012<-0.081   1984<-0.045   1976>-0.022   1995>-0.018   1980>-0.017   2004>-0.015  Higher   1996 0.076   1994 0.022
1972 to 2021 segment:
Lower   2012<-0.081   1984<-0.045   1976>-0.022   1995>-0.018   1980>-0.017   2004>-0.015  Higher   1996 0.076   1994 0.022
======================================================================================================================================
NG3051    1960 to  2021      62 years                                                                                    Series 162
[B] Entire series, effect on correlation ( 0.381) is:
Lower   1962<-0.068   2004>-0.033   2011>-0.023   1982<-0.019   1980>-0.012   1963>-0.011  Higher   1976 0.065   1960 0.024
======================================================================================================================================
NG3052    1953 to  2021      69 years                                                                                    Series 163
[B] Entire series, effect on correlation ( 0.370) is:
Lower   1962<-0.025   2011>-0.024   2007<-0.020   1954<-0.016   1953<-0.016   1982<-0.015  Higher   1976 0.032   1960 0.019
[C] Year-to-year changes diverging by over 4.0 std deviations:
2006 2007  -4.3 SD
[E] Outliers     1   3.0 SD above or -4.5 SD below mean for year
2007 -5.4 SD
======================================================================================================================================
NG3101    1951 to  2021      71 years                                                                                    Series 164
[A] Segment   High   -10   -9   -8   -7   -6   -5   -4   -3   -2   -1    0   +1   +2   +3   +4   +5   +6   +7   +8   +9  +10
---------  ----   ---  ---  ---  ---  ---  ---  ---  ---  ---  ---  ---  ---  ---  ---  ---  ---  ---  ---  ---  ---  ---
1972 2021   -5    .22 -.03 -.13 -.26  .08  .33*-.05  .03 -.19  .19  .25|-.03    -    -    -    -    -    -    -    -    -
[B] Entire series, effect on correlation ( 0.262) is:
Lower   2001<-0.075   2011>-0.035   2004>-0.022   2021<-0.020   1996>-0.019   1976>-0.013  Higher   1980 0.031   2000 0.030
1972 to 2021 segment:
Lower   2001<-0.103   2011>-0.040   2021<-0.027   2004>-0.025   1996>-0.021   1976>-0.015  Higher   1980 0.036   2000 0.035
[C] Year-to-year changes diverging by over 4.0 std deviations:
2001 2002   4.9 SD
[E] Outliers     1   3.0 SD above or -4.5 SD below mean for year
2001 -6.0 SD
======================================================================================================================================
NG3102    1956 to  2021      66 years                                                                                    Series 165
[B] Entire series, effect on correlation ( 0.416) is:
Lower   2004>-0.034   2012<-0.034   1976>-0.031   1965<-0.021   2011>-0.015   2002>-0.013  Higher   1986 0.046   1960 0.029
[E] Outliers     1   3.0 SD above or -4.5 SD below mean for year
2007 -4.8 SD
======================================================================================================================================
NG3571    1983 to  2021      39 years                                                                                    Series 166
[B] Entire series, effect on correlation ( 0.374) is:
Lower   2012<-0.123   2004>-0.032   2001<-0.031   1996>-0.023   1983<-0.014   2002>-0.010  Higher   2016 0.080   2007 0.040
======================================================================================================================================
NG3572    1991 to  2021      31 years                                                                                    Series 167
[A] Segment   High   -10   -9   -8   -7   -6   -5   -4   -3   -2   -1    0   +1   +2   +3   +4   +5   +6   +7   +8   +9  +10
---------  ----   ---  ---  ---  ---  ---  ---  ---  ---  ---  ---  ---  ---  ---  ---  ---  ---  ---  ---  ---  ---  ---
```

```
1991 2021    0    .01 -.13 -.21  .11  .13  .23 -.19 -.04 -.15  .10  .27*-.25    -    -    -    -    -    -    -    -    -    -
[B] Entire series, effect on correlation ( 0.274) is:
Lower   1993<-0.060   2012<-0.040   2004>-0.025   1995>-0.022   1991<-0.019   2018>-0.016  Higher   2007 0.089   1994 0.041
1991 to 2021 segment:
Lower   1993<-0.060   2012<-0.040   2004>-0.025   1995>-0.022   1991<-0.019   2018>-0.016  Higher   2007 0.089   1994 0.041
====================================================================================================================================
NG3581    1970 to  2021      52 years                                                                                    Series 168
[B] Entire series, effect on correlation ( 0.363) is:
Lower   1976>-0.038   2021<-0.032   2011>-0.030   2012<-0.029   2015<-0.023   1995>-0.020  Higher   1986 0.073   1996 0.062
====================================================================================================================================
NG3582    1975 to  2021      47 years                                                                                    Series 169
[A] Segment   High   -10   -9   -8   -7   -6   -5   -4   -3   -2   -1    0   +1   +2   +3   +4   +5   +6   +7   +8   +9  +10
---------  ----   ---  ---  ---  ---  ---  ---  ---  ---  ---  ---  ---  ---  ---  ---  ---  ---  ---  ---  ---  ---  ---
1975 2021  -10    .31* .00 -.12  .06  .15  .08 -.19 -.03 -.13  .16  .19| .03    -    -    -    -    -    -    -    -    -    -
[B] Entire series, effect on correlation ( 0.187) is:
Lower   1976>-0.119   2011>-0.038   1978<-0.034   1980>-0.024   1983<-0.024   2014<-0.024  Higher   2016 0.101   1986 0.058
1975 to 2021 segment:
Lower   1976>-0.119   2011>-0.038   1978<-0.034   1980>-0.024   1983<-0.024   2014<-0.024  Higher   2016 0.101   1986 0.058
[E] Outliers     1   3.0 SD above or -4.5 SD below mean for year
1976 +3.3 SD
====================================================================================================================================
NG4141    1946 to  2020      75 years                                                                                    Series 170
[A] Segment   High   -10   -9   -8   -7   -6   -5   -4   -3   -2   -1    0   +1   +2   +3   +4   +5   +6   +7   +8   +9  +10
---------  ----   ---  ---  ---  ---  ---  ---  ---  ---  ---  ---  ---  ---  ---  ---  ---  ---  ---  ---  ---  ---  ---
1971 2020  -10    .40* .04 -.05 -.11  .17  .08 -.10  .13 -.14  .14  .39| .05 -.41    -    -    -    -    -    -    -    -    -
[B] Entire series, effect on correlation ( 0.507) is:
Lower   2012<-0.027   2004>-0.025   1976>-0.018   1974>-0.014   2002>-0.011   1995>-0.010  Higher   1948 0.108   1986 0.039
1971 to 2020 segment:
Lower   2012<-0.042   2004>-0.034   1974>-0.017   2002>-0.013   1989<-0.013   2003<-0.011  Higher   1986 0.080   1980 0.030
====================================================================================================================================
NG4142    1950 to  2021      72 years                                                                                    Series 171
[B] Entire series, effect on correlation ( 0.367) is:
Lower   1953<-0.038   2002>-0.026   1997<-0.023   2004>-0.020   1989<-0.018   1974>-0.016  Higher   1976 0.064   1986 0.027
====================================================================================================================================
WG11      1964 to  2022      59 years                                                                                    Series 172
[B] Entire series, effect on correlation ( 0.521) is:
Lower   2008<-0.100   2004>-0.025   1987<-0.018   2000>-0.015   2006>-0.013   1995>-0.013  Higher   1976 0.120   1986 0.044
====================================================================================================================================
WG12      1968 to  2022      55 years                                                                                    Series 173
[B] Entire series, effect on correlation ( 0.380) is:
Lower   1969<-0.059   1976>-0.050   2016>-0.031   2000>-0.025   2004>-0.019   1979>-0.013  Higher   1996 0.056   1986 0.050
====================================================================================================================================
WG101     1964 to  2022      59 years                                                                                    Series 174
[B] Entire series, effect on correlation ( 0.307) is:
Lower   1971<-0.072   2015<-0.053   2010<-0.028   2004>-0.022   1984<-0.015   2011>-0.015  Higher   1996 0.039   1986 0.025
====================================================================================================================================
WG102     1961 to  2022      62 years                                                                                    Series 175
[A] Segment   High   -10   -9   -8   -7   -6   -5   -4   -3   -2   -1    0   +1   +2   +3   +4   +5   +6   +7   +8   +9  +10
---------  ----   ---  ---  ---  ---  ---  ---  ---  ---  ---  ---  ---  ---  ---  ---  ---  ---  ---  ---  ---  ---  ---
1961 2010    0   -.01 -.20 -.12  .04  .03  .10 -.02  .03 -.01  .14  .21* .03 -.08 -.02  .12 -.08  .00 -.16 -.15  .21  .01
1973 2022    0   -.05 -.09 -.06  .09 -.01  .15  .03 -.17 -.08  .18  .30*   -    -    -    -    -    -    -    -    -    -
[B] Entire series, effect on correlation ( 0.206) is:
Lower   1962<-0.091   2015<-0.035   2004>-0.028   2003<-0.027   2011>-0.027   1976>-0.013  Higher   2000 0.039   2019 0.025
1961 to 2010 segment:
Lower   1962<-0.119   2004>-0.035   2003<-0.034   1976>-0.016   1990>-0.013   1967<-0.011  Higher   2000 0.050   1994 0.020
1973 to 2022 segment:
Lower   2015<-0.060   2003<-0.040   2004>-0.036   2011>-0.035   1976>-0.021   2012<-0.015  Higher   2000 0.038   2019 0.030
====================================================================================================================================
WG111     1928 to  2022      95 years                                                                                    Series 176
[B] Entire series, effect on correlation ( 0.398) is:
Lower   1944>-0.027   1960>-0.025   2021<-0.021   2003<-0.019   1975<-0.013   1968<-0.013  Higher   1976 0.053   1948 0.045
====================================================================================================================================
WG112     1933 to  2022      90 years                                                                                    Series 177
[A] Segment   High   -10   -9   -8   -7   -6   -5   -4   -3   -2   -1    0   +1   +2   +3   +4   +5   +6   +7   +8   +9  +10
---------  ----   ---  ---  ---  ---  ---  ---  ---  ---  ---  ---  ---  ---  ---  ---  ---  ---  ---  ---  ---  ---  ---
1933 1982    8   -.07 -.18 -.10 -.05 -.04 -.18  .12  .04 -.02 -.11  .15|-.12 -.16 -.09  .09 -.02  .10 -.10  .17* .04  .12
[B] Entire series, effect on correlation ( 0.360) is:
Lower   1976>-0.050   2019<-0.035   1962<-0.031   2018>-0.019   1966<-0.019   2021<-0.019  Higher   2016 0.036   1996 0.023
1933 to 1982 segment:
Lower   1976>-0.067   1962<-0.059   1966<-0.034   1980>-0.022   1939<-0.017   1961<-0.013  Higher   1948 0.059   1944 0.052
====================================================================================================================================
WG121     1962 to  2022      61 years                                                                                    Series 178
[A] Segment   High   -10   -9   -8   -7   -6   -5   -4   -3   -2   -1    0   +1   +2   +3   +4   +5   +6   +7   +8   +9  +10
---------  ----   ---  ---  ---  ---  ---  ---  ---  ---  ---  ---  ---  ---  ---  ---  ---  ---  ---  ---  ---  ---  ---
1962 2011    9    .14  .05 -.08  .15 -.05  .18 -.03 -.09 -.18  .20  .35|-.23 -.11 -.12  .07 -.09  .01 -.28 -.19  .44* .21
[B] Entire series, effect on correlation ( 0.321) is:
Lower   2011>-0.055   1976>-0.054   1964<-0.023   1967<-0.021   2015<-0.011   1996>-0.011  Higher   1986 0.027   2007 0.022
1962 to 2011 segment:
Lower   1976>-0.067   2011>-0.062   1964<-0.027   1967<-0.024   1996>-0.013   1963>-0.011  Higher   1986 0.029   1994 0.021
====================================================================================================================================
WG122     1973 to  2022      50 years                                                                                    Series 179
[B] Entire series, effect on correlation ( 0.421) is:
Lower   1976>-0.073   2011>-0.034   1974<-0.025   2013>-0.014   2008<-0.013   2019<-0.010  Higher   1994 0.024   1980 0.019
====================================================================================================================================
WG131     1966 to  2022      57 years                                                                                    Series 180
[B] Entire series, effect on correlation ( 0.430) is:
Lower   2007<-0.039   1984<-0.035   1967<-0.019   2000>-0.016   1987<-0.015   2021<-0.011  Higher   1996 0.020   1994 0.020
====================================================================================================================================
WG132     1959 to  2022      64 years                                                                                    Series 181
[A] Segment   High   -10   -9   -8   -7   -6   -5   -4   -3   -2   -1    0   +1   +2   +3   +4   +5   +6   +7   +8   +9  +10
---------  ----   ---  ---  ---  ---  ---  ---  ---  ---  ---  ---  ---  ---  ---  ---  ---  ---  ---  ---  ---  ---  ---
1959 2008    9   -.04  .05 -.16  .09 -.11 -.02 -.24  .07  .03  .13  .38| .00 -.10 -.14  .10 -.05 -.13 -.14 -.15  .39* .03
```

```
[B] Entire series, effect on correlation ( 0.435) is:
Lower   1976>-0.061   2007<-0.020   1967<-0.013   1988<-0.011   2000>-0.011   1993<-0.010  Higher   2016 0.028   2019 0.018
1959 to 2008 segment:
Lower   1976>-0.068   1967<-0.015   2000>-0.012   1988<-0.012   1993<-0.012   2004>-0.010  Higher   1959 0.022   1996 0.016
==================================================================================================================================
WG141     1971 to  2022      52 years                                                                                    Series 182
[A] Segment   High   -10   -9   -8   -7   -6   -5   -4   -3   -2   -1    0   +1   +2   +3   +4   +5   +6   +7   +8   +9  +10
---------  ----   ---  ---  ---  ---  ---  ---  ---  ---  ---  ---  ---  ---  ---  ---  ---  ---  ---  ---  ---  ---  ---
1971 2020    0    .10  .20 -.14  .17  .15  .23 -.27 -.10 -.19  .09  .24*-.29 -.14    -    -    -    -    -    -    -    -
1973 2022   -5    .18  .13 -.07  .15  .07  .31*-.25 -.05 -.26  .12  .18|   -    -    -    -    -    -    -    -    -    -
[B] Entire series, effect on correlation ( 0.191) is:
Lower   1976>-0.075   2021<-0.039   1993<-0.023   1992<-0.022   1981<-0.021   2011>-0.018  Higher   1986 0.049   1996 0.047
1971 to 2020 segment:
Lower   1976>-0.083   1981<-0.027   1993<-0.027   1992<-0.026   2011>-0.019   2004>-0.019  Higher   1986 0.051   1996 0.049
1973 to 2022 segment:
Lower   1976>-0.075   2021<-0.041   1993<-0.024   1992<-0.023   1981<-0.022   2011>-0.018  Higher   1986 0.053   1996 0.051
==================================================================================================================================
WG142     1963 to  2022      60 years                                                                                    Series 183
[A] Segment   High   -10   -9   -8   -7   -6   -5   -4   -3   -2   -1    0   +1   +2   +3   +4   +5   +6   +7   +8   +9  +10
---------  ----   ---  ---  ---  ---  ---  ---  ---  ---  ---  ---  ---  ---  ---  ---  ---  ---  ---  ---  ---  ---  ---
1963 2012   -7    .05  .02 -.24  .31* .03  .19  .09  .14 -.27  .05  .28|-.26 -.09 -.11  .09 -.02 -.04 -.21 -.22  .14 -.18
1973 2022  -10    .31* .07 -.13  .15  .02  .28 -.07  .08 -.38  .09  .23|   -    -    -    -    -    -    -    -    -    -
[B] Entire series, effect on correlation ( 0.223) is:
Lower   1976>-0.084   2021<-0.043   2004>-0.025   2011>-0.022   2018>-0.020   1984<-0.017  Higher   1996 0.057   1986 0.050
1963 to 2012 segment:
Lower   1976>-0.110   2004>-0.031   2011>-0.027   1984<-0.023   1995>-0.017   1993<-0.016  Higher   1996 0.069   1986 0.060
1973 to 2022 segment:
Lower   1976>-0.093   2021<-0.059   2004>-0.028   2011>-0.024   2018>-0.022   1984<-0.021  Higher   1996 0.067   1986 0.059
==================================================================================================================================
WG151     1959 to  2022      64 years                                                                                    Series 184
[B] Entire series, effect on correlation ( 0.418) is:
Lower   2007<-0.069   2016>-0.033   2000>-0.022   1994<-0.022   1993<-0.012   2015<-0.011  Higher   1960 0.025   1976 0.023
[E] Outliers     1   3.0 SD above or -4.5 SD below mean for year
2007 -5.8 SD
==================================================================================================================================
WG152     1961 to  2022      62 years                                                                                    Series 185
[A] Segment   High   -10   -9   -8   -7   -6   -5   -4   -3   -2   -1    0   +1   +2   +3   +4   +5   +6   +7   +8   +9  +10
---------  ----   ---  ---  ---  ---  ---  ---  ---  ---  ---  ---  ---  ---  ---  ---  ---  ---  ---  ---  ---  ---  ---
1961 2010    9    .14 -.03 -.07  .16 -.03 -.12 -.21  .09  .12 -.04  .35|-.14  .04 -.15  .13 -.10 -.08 -.07 -.14  .37* .14
[B] Entire series, effect on correlation ( 0.329) is:
Lower   2004>-0.032   1999<-0.027   1967<-0.023   1990>-0.020   2015<-0.018   2011>-0.017  Higher   1976 0.090   1986 0.050
1961 to 2010 segment:
Lower   2004>-0.039   1999<-0.032   1967<-0.026   1990>-0.024   2002>-0.018   1978<-0.018  Higher   1976 0.103   1986 0.057
==================================================================================================================================
WG161     1971 to  2022      52 years                                                                                    Series 186
[A] Segment   High   -10   -9   -8   -7   -6   -5   -4   -3   -2   -1    0   +1   +2   +3   +4   +5   +6   +7   +8   +9  +10
---------  ----   ---  ---  ---  ---  ---  ---  ---  ---  ---  ---  ---  ---  ---  ---  ---  ---  ---  ---  ---  ---  ---
1971 2020    1    .18  .12  .02 -.11 -.05  .12 -.06 -.01 -.14  .00  .20| .25*-.11    -    -    -    -    -    -    -    -
1973 2022  -10    .24* .10  .06 -.07 -.05  .14 -.05  .02 -.16  .04  .20|   -    -    -    -    -    -    -    -    -    -
[B] Entire series, effect on correlation ( 0.197) is:
Lower   2015<-0.071   2011>-0.025   1976>-0.023   2002>-0.020   2003<-0.014   2013>-0.014  Higher   1986 0.033   1988 0.033
1971 to 2020 segment:
Lower   2015<-0.072   2011>-0.026   1976>-0.025   2002>-0.020   2003<-0.014   2013>-0.014  Higher   1988 0.034   1986 0.032
1973 to 2022 segment:
Lower   2015<-0.087   2011>-0.026   2002>-0.022   1976>-0.019   2003<-0.017   2013>-0.014  Higher   1986 0.039   1988 0.035
[E] Outliers     1   3.0 SD above or -4.5 SD below mean for year
2002 +3.2 SD
==================================================================================================================================
WG162     1963 to  2022      60 years                                                                                    Series 187
[B] Entire series, effect on correlation ( 0.433) is:
Lower   1976>-0.068   2021<-0.048   2000>-0.034   1965<-0.020   2005<-0.016   2004>-0.010  Higher   1986 0.057   1996 0.039
==================================================================================================================================
WG171     1957 to  2022      66 years                                                                                    Series 188
[A] Segment   High   -10   -9   -8   -7   -6   -5   -4   -3   -2   -1    0   +1   +2   +3   +4   +5   +6   +7   +8   +9  +10
---------  ----   ---  ---  ---  ---  ---  ---  ---  ---  ---  ---  ---  ---  ---  ---  ---  ---  ---  ---  ---  ---  ---
1957 2006   -9   -.09  .39* .12 -.09 -.10 -.15 -.01 -.04 -.12 -.04  .36|-.05  .19  .20 -.17 -.09 -.05  .06 -.18  .23 -.16
[B] Entire series, effect on correlation ( 0.399) is:
Lower   1993<-0.062   2011>-0.033   1957<-0.020   1986>-0.019   1978<-0.012   1960>-0.010  Higher   2004 0.034   2007 0.022
1957 to 2006 segment:
Lower   1993<-0.085   1957<-0.028   1986>-0.022   1978<-0.016   1960>-0.011   1990>-0.009  Higher   2004 0.051   1995 0.029
==================================================================================================================================
WG172     1963 to  2022      60 years                                                                                    Series 189
[B] Entire series, effect on correlation ( 0.403) is:
Lower   1976>-0.050   1978<-0.041   1967<-0.027   1993<-0.023   1990>-0.017   1963>-0.015  Higher   1995 0.020   1996 0.019
==================================================================================================================================
WG181     1927 to  2022      96 years                                                                                    Series 190
[B] Entire series, effect on correlation ( 0.611) is:
Lower   1948>-0.041   1956>-0.013   1966<-0.011   1951<-0.011   1953<-0.010   1965<-0.009  Higher   1944 0.093   1976 0.034
[E] Outliers     1   3.0 SD above or -4.5 SD below mean for year
1944 -5.6 SD
==================================================================================================================================
WG182     1921 to  2022     102 years                                                                                    Series 191
[A] Segment   High   -10   -9   -8   -7   -6   -5   -4   -3   -2   -1    0   +1   +2   +3   +4   +5   +6   +7   +8   +9  +10
---------  ----   ---  ---  ---  ---  ---  ---  ---  ---  ---  ---  ---  ---  ---  ---  ---  ---  ---  ---  ---  ---  ---
1921 1970   -9   -.03  .32* .03  .21  .12  .19  .20  .05  .03 -.36  .26|-.23 -.29 -.09  .25 -.18 -.08  .19  .13 -.10  .05
1925 1974    4   -.09  .26  .03  .13  .05  .14  .19  .04  .05 -.32  .27|-.19 -.26 -.16  .29* .00 -.10  .17  .18 -.04 -.05
1950 1999    0    .01  .28 -.11  .00 -.01  .25 -.05 -.07 -.17 -.25  .32*-.08 -.35 -.16  .13  .24  .15  .12 -.03  .30  .18
[B] Entire series, effect on correlation ( 0.447) is:
Lower   1953<-0.040   1971<-0.028   1960>-0.021   1951<-0.020   2007>-0.016   1980>-0.011  Higher   1944 0.067   2016 0.029
1921 to 1970 segment:
Lower   1953<-0.085   1951<-0.041   1960>-0.039   1927<-0.022   1921>-0.021   1942>-0.020  Higher   1944 0.266   1948 0.041
1925 to 1974 segment:
```

```
Lower   1953<-0.066   1960>-0.043   1971<-0.041   1951<-0.030   1942>-0.022   1959>-0.018  Higher   1944 0.217   1955 0.025
1950 to 1999 segment:
Lower   1953<-0.075   1971<-0.047   1960>-0.046   1951<-0.035   1980>-0.024   1959>-0.019  Higher   1976 0.061   1995 0.052
====================================================================================================================================
WG191     1964 to  2022      59 years                                                                                    Series 192
[A] Segment   High   -10   -9   -8   -7   -6   -5   -4   -3   -2   -1    0   +1   +2   +3   +4   +5   +6   +7   +8   +9  +10
---------  ----   ---  ---  ---  ---  ---  ---  ---  ---  ---  ---  ---  ---  ---  ---  ---  ---  ---  ---  ---  ---  ---
1964 2013   -9   -.04  .41*-.10 -.02 -.03  .05 -.01 -.23 -.15 -.06  .30| .24 -.03 -.03 -.11  .17 -.02 -.09 -.24 -.05    -
1973 2022   -9    .02  .45*-.11  .01 -.04  .05  .01 -.20 -.21 -.08  .36|  -    -    -    -    -    -    -    -    -    -    -
[B] Entire series, effect on correlation ( 0.350) is:
Lower   1985<-0.063   1986>-0.035   2000>-0.029   1973<-0.028   2004>-0.023   1965<-0.016  Higher   2016 0.045   1976 0.038
1964 to 2013 segment:
Lower   1985<-0.057   1986>-0.036   2000>-0.031   1973<-0.031   2004>-0.024   1965<-0.018  Higher   1976 0.052   1996 0.038
1973 to 2022 segment:
Lower   1985<-0.087   1986>-0.037   1973<-0.031   2000>-0.031   2004>-0.024   1993<-0.012  Higher   2016 0.047   1976 0.040
====================================================================================================================================
WG192     1967 to  2022      56 years                                                                                    Series 193
[B] Entire series, effect on correlation ( 0.390) is:
Lower   1985<-0.043   2004>-0.027   2007>-0.018   1995>-0.016   2015<-0.016   2003<-0.016  Higher   1996 0.077   2019 0.021
[E] Outliers     1   3.0 SD above or -4.5 SD below mean for year
1985 -4.8 SD
====================================================================================================================================
WG21      1959 to  2022      64 years                                                                                    Series 194
[A] Segment   High   -10   -9   -8   -7   -6   -5   -4   -3   -2   -1    0   +1   +2   +3   +4   +5   +6   +7   +8   +9  +10
---------  ----   ---  ---  ---  ---  ---  ---  ---  ---  ---  ---  ---  ---  ---  ---  ---  ---  ---  ---  ---  ---  ---
1959 2008    1    .10  .25  .04  .06  .06  .06 -.23 -.15 -.17 -.07  .16| .40*-.10 -.15 -.03  .16 -.05 -.16 -.19 -.07  .20
1973 2022   -9    .15  .31* .10 -.03  .17 -.05 -.06 -.03 -.24 -.13  .13|  -    -    -    -    -    -    -    -    -    -    -
[B] Entire series, effect on correlation ( 0.074) is:
Lower   1960>-0.035   2010<-0.034   2016>-0.024   1971<-0.023   2019<-0.021   2004>-0.020  Higher   1996 0.061   1959 0.024
1959 to 2008 segment:
Lower   1960>-0.048   1971<-0.038   2004>-0.027   1975<-0.025   1993<-0.024   2000>-0.017  Higher   1996 0.076   1959 0.029
1973 to 2022 segment:
Lower   2010<-0.049   2016>-0.031   2019<-0.025   2004>-0.025   1975<-0.020   1993<-0.019  Higher   1996 0.066   1999 0.024
====================================================================================================================================
WG22      1959 to  2022      64 years                                                                                    Series 195
[A] Segment   High   -10   -9   -8   -7   -6   -5   -4   -3   -2   -1    0   +1   +2   +3   +4   +5   +6   +7   +8   +9  +10
---------  ----   ---  ---  ---  ---  ---  ---  ---  ---  ---  ---  ---  ---  ---  ---  ---  ---  ---  ---  ---  ---  ---
1973 2022   -1    .28  .07 -.18 -.18  .06  .07 -.04 -.04 -.31  .38* .36|  -    -    -    -    -    -    -    -    -    -    -
[B] Entire series, effect on correlation ( 0.378) is:
Lower   2011>-0.037   1977<-0.035   2004>-0.022   2019<-0.018   2002>-0.014   1960>-0.014  Higher   1996 0.057   1994 0.021
1973 to 2022 segment:
Lower   1977<-0.050   2011>-0.047   2004>-0.027   2019<-0.020   2002>-0.018   2016>-0.015  Higher   1996 0.072   1994 0.029
====================================================================================================================================
WG201     1972 to  2022      51 years                                                                                    Series 196
[B] Entire series, effect on correlation ( 0.521) is:
Lower   2018<-0.086   2000>-0.030   1994<-0.019   1980>-0.019   1986>-0.017   2003<-0.014  Higher   1976 0.035   1995 0.019
====================================================================================================================================
WG202     1960 to  2022      63 years                                                                                    Series 197
[A] Segment   High   -10   -9   -8   -7   -6   -5   -4   -3   -2   -1    0   +1   +2   +3   +4   +5   +6   +7   +8   +9  +10
---------  ----   ---  ---  ---  ---  ---  ---  ---  ---  ---  ---  ---  ---  ---  ---  ---  ---  ---  ---  ---  ---  ---
1960 2009    0   -.09 -.02 -.26  .10 -.03  .26 -.03 -.19  .01  .10  .31*-.18  .15 -.12  .14  .28  .00 -.18 -.19  .24 -.19
[B] Entire series, effect on correlation ( 0.382) is:
Lower   1981<-0.069   1976>-0.043   1960>-0.038   1986>-0.030   1984<-0.013   1961<-0.012  Higher   1996 0.044   1995 0.020
1960 to 2009 segment:
Lower   1981<-0.077   1960>-0.044   1976>-0.044   1986>-0.033   1984<-0.015   1961<-0.013  Higher   1996 0.061   1995 0.028
====================================================================================================================================
WG211     1957 to  2022      66 years                                                                                    Series 198
[A] Segment   High   -10   -9   -8   -7   -6   -5   -4   -3   -2   -1    0   +1   +2   +3   +4   +5   +6   +7   +8   +9  +10
---------  ----   ---  ---  ---  ---  ---  ---  ---  ---  ---  ---  ---  ---  ---  ---  ---  ---  ---  ---  ---  ---  ---
1957 2006   -1    .11  .05 -.08 -.22  .07  .17 -.07 -.12 -.07  .22* .12|-.01 -.13  .16 -.02  .07  .05 -.05 -.16  .00  .08
1973 2022   -6    .12  .03 -.10 -.16  .22* .18  .01 -.21 -.05  .15  .10|  -    -    -    -    -    -    -    -    -    -    -
[B] Entire series, effect on correlation ( 0.166) is:
Lower   2001<-0.071   1996>-0.066   1976>-0.061   2004>-0.020   2016>-0.015   1978<-0.013  Higher   1986 0.076   1960 0.043
1957 to 2006 segment:
Lower   1996>-0.079   2001<-0.079   1976>-0.072   2004>-0.023   1978<-0.014   1993<-0.012  Higher   1986 0.101   1960 0.057
1973 to 2022 segment:
Lower   1996>-0.073   2001<-0.069   1976>-0.064   2004>-0.022   2016>-0.015   1978<-0.013  Higher   1986 0.093   2019 0.032
[C] Year-to-year changes diverging by over 4.0 std deviations:
2001 2002   4.2 SD
[E] Outliers     2   3.0 SD above or -4.5 SD below mean for year
1996 +3.4 SD;    2001 -5.0 SD
====================================================================================================================================
WG212     1957 to  2022      66 years                                                                                    Series 199
[B] Entire series, effect on correlation ( 0.407) is:
Lower   1978<-0.065   2004>-0.029   2012<-0.029   2002>-0.014   2018>-0.012   2008<-0.010  Higher   1960 0.048   1996 0.037
====================================================================================================================================
WG221     1927 to  2022      96 years                                                                                    Series 200
[A] Segment   High   -10   -9   -8   -7   -6   -5   -4   -3   -2   -1    0   +1   +2   +3   +4   +5   +6   +7   +8   +9  +10
---------  ----   ---  ---  ---  ---  ---  ---  ---  ---  ---  ---  ---  ---  ---  ---  ---  ---  ---  ---  ---  ---  ---
1927 1976    0    .10 -.12  .09 -.10  .01 -.09  .21 -.11 -.16 -.05  .27*-.02 -.01 -.06  .04  .13 -.20  .12  .09  .12 -.22
[B] Entire series, effect on correlation ( 0.438) is:
Lower   1944>-0.075   1933<-0.017   1980>-0.013   1960>-0.012   2007>-0.011   1975<-0.011  Higher   1948 0.047   2016 0.036
1927 to 1976 segment:
Lower   1944>-0.128   1933<-0.034   1975<-0.020   1959>-0.019   1960>-0.019   1934>-0.019  Higher   1948 0.162   1976 0.056
====================================================================================================================================
WG222     1946 to  2022      77 years                                                                                    Series 201
[B] Entire series, effect on correlation ( 0.508) is:
Lower   1948>-0.031   1980>-0.027   1965<-0.026   1975<-0.026   1959>-0.016   1950<-0.016  Higher   1976 0.033   2016 0.029
====================================================================================================================================
WG231     1961 to  2022      62 years                                                                                    Series 202
[B] Entire series, effect on correlation ( 0.585) is:
Lower   1971<-0.032   2004>-0.023   1961<-0.023   1970>-0.016   1990>-0.014   2006>-0.013  Higher   1976 0.115   1996 0.040
```

```
====================================================================================================================================
WG232     1962 to  2022      61 years                                                                                    Series 203
[B] Entire series, effect on correlation ( 0.567) is:
Lower   2008<-0.089   2022<-0.034   2004>-0.028   2016>-0.026   2000>-0.024   1994<-0.017  Higher   1976 0.129   1986 0.028
====================================================================================================================================
WG241     1916 to  2022     107 years                                                                                    Series 204
[A] Segment   High   -10   -9   -8   -7   -6   -5   -4   -3   -2   -1    0   +1   +2   +3   +4   +5   +6   +7   +8   +9  +10
---------  ----   ---  ---  ---  ---  ---  ---  ---  ---  ---  ---  ---  ---  ---  ---  ---  ---  ---  ---  ---  ---  ---
1916 1965   -7   -.06 -.23 -.12  .25*-.05  .05  .11  .03 -.22 -.13  .16| .01 -.22 -.07  .09  .15 -.03 -.02  .04  .02 -.01
1925 1974    0    .01 -.15 -.15  .19  .07  .07  .03  .07 -.17 -.13  .21*-.01 -.32 -.03  .13  .05 -.07 -.09  .03 -.02  .07
1950 1999    0    .03 -.07 -.16  .15 -.03  .12  .05  .10 -.14 -.02  .31*-.06 -.26 -.10  .02 -.10  .04 -.12 -.10 -.07  .30
[B] Entire series, effect on correlation ( 0.331) is:
Lower   1964<-0.043   1929<-0.031   1948>-0.019   1916<-0.016   1921>-0.013   2007>-0.013  Higher   1944 0.066   1976 0.053
1916 to 1965 segment:
Lower   1964<-0.065   1929<-0.050   1916<-0.026   1921>-0.024   1960>-0.023   1948>-0.019  Higher   1944 0.187   1931 0.032
1925 to 1974 segment:
Lower   1964<-0.071   1929<-0.052   1948>-0.031   1960>-0.028   1939<-0.019   1966<-0.018  Higher   1944 0.200   1931 0.030
1950 to 1999 segment:
Lower   1964<-0.110   1960>-0.031   1966<-0.027   1980>-0.021   1973<-0.018   1965<-0.013  Higher   1976 0.158   1986 0.028
[C] Year-to-year changes diverging by over 4.0 std deviations:
1963 1964  -4.0 SD
[E] Outliers     1   3.0 SD above or -4.5 SD below mean for year
1964 -6.0 SD
====================================================================================================================================
WG242     1914 to  2022     109 years                                                                                    Series 205
[A] Segment   High   -10   -9   -8   -7   -6   -5   -4   -3   -2   -1    0   +1   +2   +3   +4   +5   +6   +7   +8   +9  +10
---------  ----   ---  ---  ---  ---  ---  ---  ---  ---  ---  ---  ---  ---  ---  ---  ---  ---  ---  ---  ---  ---  ---
1914 1963    8   -.09 -.14  .06  .12  .06 -.15  .11 -.10 -.23 -.29  .16|-.09 -.30  .11  .06  .24  .12  .12  .26* .04  .03
1925 1974    5   -.15 -.03  .01 -.04  .06 -.02  .10 -.13 -.17 -.23  .18|-.11 -.29  .14  .13  .23* .14  .09  .13 -.03 -.11
[B] Entire series, effect on correlation ( 0.404) is:
Lower   1929<-0.021   1965<-0.021   1918>-0.021   1944>-0.019   1973<-0.017   1939<-0.017  Higher   1976 0.068   2016 0.037
1914 to 1963 segment:
Lower   1929<-0.054   1918>-0.045   1939<-0.043   1927<-0.022   1955<-0.020   1919>-0.017  Higher   1948 0.062   1960 0.045
1925 to 1974 segment:
Lower   1929<-0.041   1965<-0.040   1973<-0.035   1939<-0.033   1927<-0.017   1955<-0.015  Higher   1948 0.045   1960 0.041
====================================================================================================================================
WG251     1954 to  2022      69 years                                                                                    Series 206
[A] Segment   High   -10   -9   -8   -7   -6   -5   -4   -3   -2   -1    0   +1   +2   +3   +4   +5   +6   +7   +8   +9  +10
---------  ----   ---  ---  ---  ---  ---  ---  ---  ---  ---  ---  ---  ---  ---  ---  ---  ---  ---  ---  ---  ---  ---
1954 2003  -10    .35* .06 -.12 -.17  .08  .11 -.17 -.26 -.25  .12  .34|-.25 -.01 -.10  .23  .20  .12 -.09  .01  .13 -.02
[B] Entire series, effect on correlation ( 0.384) is:
Lower   2004>-0.041   1976>-0.040   1960>-0.033   1993<-0.021   1954<-0.018   1995>-0.014  Higher   1996 0.068   2016 0.038
1954 to 2003 segment:
Lower   1976>-0.047   1960>-0.046   1993<-0.030   1954<-0.026   1995>-0.018   1971<-0.016  Higher   1996 0.118   1986 0.025
====================================================================================================================================
WG252     1938 to  2022      85 years                                                                                    Series 207
[A] Segment   High   -10   -9   -8   -7   -6   -5   -4   -3   -2   -1    0   +1   +2   +3   +4   +5   +6   +7   +8   +9  +10
---------  ----   ---  ---  ---  ---  ---  ---  ---  ---  ---  ---  ---  ---  ---  ---  ---  ---  ---  ---  ---  ---  ---
1938 1987    0    .01  .01 -.01 -.02  .10  .07  .14 -.05  .01 -.03  .25*-.16 -.32 -.07  .22 -.02 -.03  .10  .20  .03  .20
1950 1999    4    .17  .04 -.05 -.08  .07  .21 -.17 -.09 -.11  .22  .20|-.12 -.08 -.21  .23* .10  .09 -.08  .09  .19  .14
[B] Entire series, effect on correlation ( 0.383) is:
Lower   1976>-0.026   1960>-0.018   1953<-0.017   2004>-0.016   1959>-0.016   1980>-0.013  Higher   1948 0.046   2016 0.045
1938 to 1987 segment:
Lower   1953<-0.033   1960>-0.031   1959>-0.030   1976>-0.029   1941<-0.024   1980>-0.022  Higher   1948 0.141   1944 0.068
1950 to 1999 segment:
Lower   1976>-0.038   1960>-0.038   1959>-0.036   1953<-0.031   1980>-0.028   1990>-0.019  Higher   1996 0.161   1986 0.070
====================================================================================================================================
WG261     1962 to  2022      61 years                                                                                    Series 208
[A] Segment   High   -10   -9   -8   -7   -6   -5   -4   -3   -2   -1    0   +1   +2   +3   +4   +5   +6   +7   +8   +9  +10
---------  ----   ---  ---  ---  ---  ---  ---  ---  ---  ---  ---  ---  ---  ---  ---  ---  ---  ---  ---  ---  ---  ---
1962 2011   -1    .24 -.24 -.05 -.17  .12 -.10  .00 -.06  .19  .41* .08|-.14 -.13 -.12  .10  .01 -.07 -.38  .16  .16  .19
1973 2022   -1    .29 -.08 -.11 -.25  .07  .04 -.18 -.02  .06  .36* .20|   -    -    -    -    -    -    -    -    -    -    -
[B] Entire series, effect on correlation ( 0.138) is:
Lower   2008<-0.047   1976>-0.041   2004>-0.037   1964<-0.028   1962<-0.024   1995>-0.022  Higher   2016 0.090   1986 0.056
1962 to 2011 segment:
Lower   2008<-0.058   2004>-0.044   1976>-0.040   1964<-0.033   1962<-0.029   1995>-0.025  Higher   1986 0.079   1996 0.062
1973 to 2022 segment:
Lower   2008<-0.062   1976>-0.055   2004>-0.045   1995>-0.027   2021<-0.025   2011>-0.024  Higher   2016 0.095   1986 0.060
====================================================================================================================================
WG262     1964 to  2022      59 years                                                                                    Series 209
[A] Segment   High   -10   -9   -8   -7   -6   -5   -4   -3   -2   -1    0   +1   +2   +3   +4   +5   +6   +7   +8   +9  +10
---------  ----   ---  ---  ---  ---  ---  ---  ---  ---  ---  ---  ---  ---  ---  ---  ---  ---  ---  ---  ---  ---  ---
1964 2013   -6   -.06  .14 -.18 -.03  .33* .06  .01  .05  .05 -.11  .29|-.02 -.12 -.11  .28 -.03  .08 -.21 -.21 -.06    -
1973 2022   -6   -.07  .24 -.15 -.06  .37* .06 -.01 -.06  .03 -.16  .24|   -    -    -    -    -    -    -    -    -    -    -
[B] Entire series, effect on correlation ( 0.228) is:
Lower   1982<-0.043   2016>-0.040   1995>-0.028   2000>-0.020   2011>-0.017   2018>-0.015  Higher   2007 0.048   1976 0.045
1964 to 2013 segment:
Lower   1982<-0.050   1995>-0.035   2000>-0.025   2011>-0.021   1992<-0.015   1970>-0.012  Higher   2007 0.046   1976 0.039
1973 to 2022 segment:
Lower   1982<-0.048   2016>-0.045   1995>-0.031   2000>-0.022   2011>-0.019   2018>-0.016  Higher   2007 0.046   1976 0.045
====================================================================================================================================
WG271     1953 to  2022      70 years                                                                                    Series 210
[A] Segment   High   -10   -9   -8   -7   -6   -5   -4   -3   -2   -1    0   +1   +2   +3   +4   +5   +6   +7   +8   +9  +10
---------  ----   ---  ---  ---  ---  ---  ---  ---  ---  ---  ---  ---  ---  ---  ---  ---  ---  ---  ---  ---  ---  ---
1953 2002   -3   -.22  .07 -.21  .22 -.29  .01  .18  .32*-.22 -.07  .29| .23  .01 -.07  .15  .17  .05 -.05 -.14 -.10 -.13
1973 2022    0   -.02  .11 -.28 -.03 -.15  .08  .25  .16 -.21 -.14  .31*   -    -    -    -    -    -    -    -    -    -    -
[B] Entire series, effect on correlation ( 0.289) is:
Lower   1955<-0.057   2015<-0.040   1999<-0.034   1986>-0.033   1983<-0.021   1981<-0.018  Higher   1976 0.037   1980 0.026
1953 to 2002 segment:
Lower   1955<-0.074   1986>-0.048   1999<-0.043   1983<-0.026   1981<-0.022   1975<-0.021  Higher   1976 0.047   1980 0.034
1973 to 2022 segment:
Lower   2015<-0.053   1986>-0.046   1999<-0.041   1983<-0.029   1981<-0.025   1975<-0.020  Higher   1976 0.039   1988 0.029
```

```
===========================================================================================================================
WG272     1954 to  2022      69 years                                                                           Series 211
[A] Segment   High   -10   -9   -8   -7   -6   -5   -4   -3   -2   -1    0   +1   +2   +3   +4   +5   +6   +7   +8   +9  +10
---------  ----   ---  ---  ---  ---  ---  ---  ---  ---  ---  ---  ---  ---  ---  ---  ---  ---  ---  ---  ---  ---  ---
1954 2003   -9    .02  .50*-.14 -.24 -.30  .15  .17 -.17 -.24  .11  .30| .06  .13 -.13 -.02  .16  .27 -.02 -.14  .02 -.09
[B] Entire series, effect on correlation ( 0.385) is:
Lower   1986>-0.048   1959>-0.045   1976>-0.033   1957<-0.026   1955<-0.015   1981<-0.011  Higher   2016 0.042   2000 0.024
1954 to 2003 segment:
Lower   1986>-0.060   1959>-0.059   1976>-0.032   1957<-0.025   1955<-0.018   1981<-0.013  Higher   2000 0.039   1988 0.032
===========================================================================================================================
WG281     1960 to  2022      63 years                                                                           Series 212
[B] Entire series, effect on correlation ( 0.527) is:
Lower   2022<-0.016   1986>-0.015   1982<-0.014   2007<-0.014   2018>-0.013   2013>-0.012  Higher   1976 0.040   1960 0.021
===========================================================================================================================
WG282     1964 to  2022      59 years                                                                           Series 213
[A] Segment   High   -10   -9   -8   -7   -6   -5   -4   -3   -2   -1    0   +1   +2   +3   +4   +5   +6   +7   +8   +9  +10
---------  ----   ---  ---  ---  ---  ---  ---  ---  ---  ---  ---  ---  ---  ---  ---  ---  ---  ---  ---  ---  ---  ---
1973 2022    0    .00 -.12 -.11  .09  .13  .07  .01 -.26 -.10  .21  .28*   -    -    -    -    -    -    -    -    -    -
[B] Entire series, effect on correlation ( 0.266) is:
Lower   2016>-0.035   2001<-0.031   1970<-0.027   2015<-0.024   1996>-0.024   1987<-0.017  Higher   1976 0.072   1988 0.029
1973 to 2022 segment:
Lower   2001<-0.051   2016>-0.040   2015<-0.035   1987<-0.032   1996>-0.026   2014<-0.017  Higher   1976 0.095   1988 0.036
===========================================================================================================================
WG291     1920 to  2022     103 years                                                                           Series 214
[A] Segment   High   -10   -9   -8   -7   -6   -5   -4   -3   -2   -1    0   +1   +2   +3   +4   +5   +6   +7   +8   +9  +10
---------  ----   ---  ---  ---  ---  ---  ---  ---  ---  ---  ---  ---  ---  ---  ---  ---  ---  ---  ---  ---  ---  ---
1920 1969    8   -.19  .00  .08 -.16  .05 -.24  .13  .03 -.05 -.19  .33|-.14 -.18 -.09  .07 -.27 -.14  .06  .36*-.03  .11
[B] Entire series, effect on correlation ( 0.427) is:
Lower   1944>-0.035   1970<-0.024   1986>-0.024   1968<-0.023   1959>-0.016   1936<-0.013  Higher   1948 0.037   2016 0.027
1920 to 1969 segment:
Lower   1944>-0.059   1968<-0.046   1959>-0.032   1936<-0.027   1923>-0.023   1942>-0.021  Higher   1948 0.119   1960 0.024
===========================================================================================================================
WG292     1933 to  2022      90 years                                                                           Series 215
[B] Entire series, effect on correlation ( 0.474) is:
Lower   1956>-0.031   1986>-0.023   1944>-0.021   2007>-0.014   1990<-0.011   1942>-0.010  Higher   2016 0.032   1948 0.028
===========================================================================================================================
WG31      1967 to  2022      56 years                                                                           Series 216
[B] Entire series, effect on correlation ( 0.435) is:
Lower   1976>-0.057   2004>-0.029   1967<-0.022   1974<-0.012   1998<-0.011   2019<-0.010  Higher   2016 0.074   1996 0.041
===========================================================================================================================
WG32      1961 to  2022      62 years                                                                           Series 217
[B] Entire series, effect on correlation ( 0.361) is:
Lower   1976>-0.042   2011>-0.041   1975<-0.023   1974<-0.021   1961<-0.020   1990>-0.019  Higher   1996 0.081   2016 0.040
===========================================================================================================================
WG301     1969 to  2022      54 years                                                                           Series 218
[A] Segment   High   -10   -9   -8   -7   -6   -5   -4   -3   -2   -1    0   +1   +2   +3   +4   +5   +6   +7   +8   +9  +10
---------  ----   ---  ---  ---  ---  ---  ---  ---  ---  ---  ---  ---  ---  ---  ---  ---  ---  ---  ---  ---  ---  ---
1973 2022    0    .02  .06 -.12 -.03  .10 -.07 -.07 -.27  .31  .05  .32*   -    -    -    -    -    -    -    -    -    -
[B] Entire series, effect on correlation ( 0.330) is:
Lower   1978<-0.261   1976>-0.025   1980>-0.013   2007>-0.012   1992<-0.010   1990>-0.006  Higher   2016 0.054   2004 0.019
1973 to 2022 segment:
Lower   1978<-0.273   1976>-0.025   1980>-0.013   2007>-0.013   1992<-0.010   1990>-0.006  Higher   2016 0.055   1982 0.019
[C] Year-to-year changes diverging by over 4.0 std deviations:
1977 1978  -4.2 SD
[E] Outliers     1   3.0 SD above or -4.5 SD below mean for year
1978 -5.9 SD
===========================================================================================================================
WG302     1960 to  2022      63 years                                                                           Series 219
[A] Segment   High   -10   -9   -8   -7   -6   -5   -4   -3   -2   -1    0   +1   +2   +3   +4   +5   +6   +7   +8   +9  +10
---------  ----   ---  ---  ---  ---  ---  ---  ---  ---  ---  ---  ---  ---  ---  ---  ---  ---  ---  ---  ---  ---  ---
1960 2009    0    .00  .01 -.02 -.04  .09  .10 -.04 -.28 -.01  .17  .32*-.18  .12 -.01  .07 -.09 -.10 -.12 -.02  .26 -.08
[B] Entire series, effect on correlation ( 0.422) is:
Lower   1960>-0.057   2007>-0.032   1977<-0.018   1976>-0.016   1961<-0.016   1964<-0.015  Higher   2016 0.071   1996 0.033
1960 to 2009 segment:
Lower   1960>-0.073   2007>-0.041   1977<-0.023   1961<-0.022   1964<-0.021   1993<-0.019  Higher   1996 0.059   2004 0.047
[E] Outliers     1   3.0 SD above or -4.5 SD below mean for year
1960 +3.0 SD
===========================================================================================================================
WG311     1964 to  2022      59 years                                                                           Series 220
[A] Segment   High   -10   -9   -8   -7   -6   -5   -4   -3   -2   -1    0   +1   +2   +3   +4   +5   +6   +7   +8   +9  +10
---------  ----   ---  ---  ---  ---  ---  ---  ---  ---  ---  ---  ---  ---  ---  ---  ---  ---  ---  ---  ---  ---  ---
1973 2022    0   -.03  .14 -.23  .04  .05  .13 -.14  .15 -.14 -.04  .32*   -    -    -    -    -    -    -    -    -    -
[B] Entire series, effect on correlation ( 0.323) is:
Lower   2016>-0.021   2011>-0.020   1990>-0.016   1980>-0.016   2018>-0.015   1986>-0.013  Higher   2007 0.032   1996 0.024
1973 to 2022 segment:
Lower   2016>-0.023   2011>-0.022   1980>-0.017   1990>-0.017   2018>-0.015   1986>-0.015  Higher   2007 0.028   1996 0.024
[E] Outliers     1   3.0 SD above or -4.5 SD below mean for year
2007 -4.5 SD
===========================================================================================================================
WG312     1963 to  2022      60 years                                                                           Series 221
[B] Entire series, effect on correlation ( 0.325) is:
Lower   2020<-0.074   2016>-0.049   2014<-0.016   2011>-0.014   1984<-0.014   1963>-0.012  Higher   1976 0.060   2007 0.031
===========================================================================================================================
WG321     1967 to  2022      56 years                                                                           Series 222
[A] Segment   High   -10   -9   -8   -7   -6   -5   -4   -3   -2   -1    0   +1   +2   +3   +4   +5   +6   +7   +8   +9  +10
---------  ----   ---  ---  ---  ---  ---  ---  ---  ---  ---  ---  ---  ---  ---  ---  ---  ---  ---  ---  ---  ---  ---
1967 2016    0    .12  .15 -.31 -.09  .10  .06 -.25 -.06 -.12 -.21  .28*-.03 -.08  .12  .07  .00 -.09    -    -    -    -
1973 2022    0    .15  .10 -.26 -.07  .04  .15 -.26 -.07 -.16 -.21  .28*   -    -    -    -    -    -    -    -    -    -
[B] Entire series, effect on correlation ( 0.273) is:
Lower   1973<-0.070   1988<-0.022   2004>-0.021   1976<-0.020   1993<-0.018   2011>-0.016  Higher   2016 0.115   1995 0.043
1967 to 2016 segment:
Lower   1973<-0.075   1976>-0.024   1988<-0.023   2004>-0.023   1993<-0.019   2011>-0.018  Higher   2016 0.119   1995 0.044
```

```
1973 to 2022 segment:
Lower   1973<-0.079   1988<-0.024   2004>-0.022   1976>-0.020   1993<-0.020   2011>-0.017  Higher   2016 0.119   1995 0.043
[E] Outliers     1   3.0 SD above or -4.5 SD below mean for year
1973 -4.7 SD
==================================================================================================================================
WG322     1959 to  2022      64 years                                                                                    Series 223
[A] Segment   High   -10   -9   -8   -7   -6   -5   -4   -3   -2   -1    0   +1   +2   +3   +4   +5   +6   +7   +8   +9  +10
---------  ----   ---  ---  ---  ---  ---  ---  ---  ---  ---  ---  ---  ---  ---  ---  ---  ---  ---  ---  ---  ---  ---
1959 2008    5    .15  .06 -.11 -.17  .12 -.05 -.08 -.17 -.14 -.18  .05| .11  .04  .18 -.07  .20*-.02  .02 -.14  .13  .01
1973 2022    0    .08  .13 -.22 -.15  .10  .08 -.17 -.16 -.17 -.18  .21*   -    -    -    -    -    -    -    -    -    -
[B] Entire series, effect on correlation ( 0.182) is:
Lower   1976>-0.082   1973<-0.030   2004>-0.022   1959>-0.017   1962<-0.016   2007>-0.014  Higher   2016 0.093   1995 0.071
1959 to 2008 segment:
Lower   1976>-0.085   1973<-0.033   2004>-0.024   1959>-0.019   1962<-0.018   2007>-0.015  Higher   1995 0.146   1996 0.058
1973 to 2022 segment:
Lower   1976>-0.100   1973<-0.035   2004>-0.026   1986>-0.017   2007>-0.017   1979>-0.013  Higher   2016 0.102   1995 0.072
[C] Year-to-year changes diverging by over 4.0 std deviations:
1975 1976   4.8 SD
==================================================================================================================================
WG331     1942 to  2022      81 years                                                                                    Series 224
[A] Segment   High   -10   -9   -8   -7   -6   -5   -4   -3   -2   -1    0   +1   +2   +3   +4   +5   +6   +7   +8   +9  +10
---------  ----   ---  ---  ---  ---  ---  ---  ---  ---  ---  ---  ---  ---  ---  ---  ---  ---  ---  ---  ---  ---  ---
1942 1991    1   -.04  .07 -.10  .00 -.16 -.02 -.22  .10 -.21 -.05 -.02| .25* .12  .17  .15  .13  .04  .08 -.30 -.12  .00
[B] Entire series, effect on correlation ( 0.205) is:
Lower   1948>-0.082   1944>-0.055   1960>-0.032   1955<-0.017   1980>-0.011   1975<-0.010  Higher   1976 0.075   2004 0.047
1942 to 1991 segment:
Lower   1948>-0.122   1944>-0.069   1960>-0.050   1955<-0.026   1980>-0.017   1975<-0.016  Higher   1976 0.197   1988 0.028
[C] Year-to-year changes diverging by over 4.0 std deviations:
1943 1944   4.3 SD    1947 1948   4.7 SD
[E] Outliers     1   3.0 SD above or -4.5 SD below mean for year
1948 +3.2 SD
==================================================================================================================================
WG332     1945 to  2022      78 years                                                                                    Series 225
[A] Segment   High   -10   -9   -8   -7   -6   -5   -4   -3   -2   -1    0   +1   +2   +3   +4   +5   +6   +7   +8   +9  +10
---------  ----   ---  ---  ---  ---  ---  ---  ---  ---  ---  ---  ---  ---  ---  ---  ---  ---  ---  ---  ---  ---  ---
1973 2022   -4   -.09  .13 -.07 -.20 -.16 -.10  .39*-.19  .05 -.34  .38|   -    -    -    -    -    -    -    -    -    -
[B] Entire series, effect on correlation ( 0.379) is:
Lower   2016>-0.030   1955<-0.024   1986>-0.020   1973<-0.020   2015<-0.015   1960>-0.015  Higher   2004 0.027   1976 0.017
1973 to 2022 segment:
Lower   2016>-0.045   1986>-0.032   1973<-0.026   2015<-0.020   1982<-0.019   2007>-0.018  Higher   2004 0.041   2000 0.024
==================================================================================================================================
WG41      1963 to  2022      60 years                                                                                    Series 226
[A] Segment   High   -10   -9   -8   -7   -6   -5   -4   -3   -2   -1    0   +1   +2   +3   +4   +5   +6   +7   +8   +9  +10
---------  ----   ---  ---  ---  ---  ---  ---  ---  ---  ---  ---  ---  ---  ---  ---  ---  ---  ---  ---  ---  ---  ---
1963 2012    9   -.10 -.04 -.11  .17  .00  .08  .01  .00 -.21  .09  .34|-.15 -.03 -.07  .08  .05  .01 -.30 -.22  .38* .10
[B] Entire series, effect on correlation ( 0.344) is:
Lower   2000>-0.028   2004>-0.026   2008<-0.026   1993<-0.014   1979>-0.013   1967<-0.011  Higher   1976 0.051   1982 0.021
1963 to 2012 segment:
Lower   2000>-0.032   2004>-0.030   2008<-0.029   1993<-0.016   1979>-0.014   1967<-0.012  Higher   1976 0.057   1982 0.023
[E] Outliers     1   3.0 SD above or -4.5 SD below mean for year
2007 -5.2 SD
==================================================================================================================================
WG42      1961 to  2022      62 years                                                                                    Series 227
[B] Entire series, effect on correlation ( 0.315) is:
Lower   1993<-0.035   1986>-0.030   2011>-0.028   2000>-0.015   2019<-0.013   2002>-0.013  Higher   1980 0.044   1976 0.032
==================================================================================================================================
WG51      1958 to  2022      65 years                                                                                    Series 228
[B] Entire series, effect on correlation ( 0.412) is:
Lower   1958<-0.062   2016>-0.048   1960>-0.028   2017<-0.026   1980>-0.023   2006<-0.014  Higher   1976 0.083   1996 0.023
==================================================================================================================================
WG52      1965 to  2022      58 years                                                                                    Series 229
[A] Segment   High   -10   -9   -8   -7   -6   -5   -4   -3   -2   -1    0   +1   +2   +3   +4   +5   +6   +7   +8   +9  +10
---------  ----   ---  ---  ---  ---  ---  ---  ---  ---  ---  ---  ---  ---  ---  ---  ---  ---  ---  ---  ---  ---  ---
1973 2022    0    .07  .15 -.20 -.04  .23  .09  .00 -.35 -.06  .01  .28*   -    -    -    -    -    -    -    -    -    -
[B] Entire series, effect on correlation ( 0.267) is:
Lower   2022<-0.101   1976>-0.030   1980>-0.020   1965<-0.015   2004>-0.013   2008<-0.012  Higher   1996 0.031   2007 0.024
1973 to 2022 segment:
Lower   2022<-0.127   1976>-0.034   1980>-0.022   2004>-0.014   2008<-0.013   2000>-0.013  Higher   1996 0.031   1988 0.024
==================================================================================================================================
WG61      1925 to  2022      98 years                                                                                    Series 230
[B] Entire series, effect on correlation ( 0.615) is:
Lower   1948>-0.040   1969<-0.018   1994<-0.017   1960>-0.013   1950<-0.010   1939<-0.010  Higher   1944 0.078   1976 0.056
==================================================================================================================================
WG62      1933 to  2022      90 years                                                                                    Series 231
[B] Entire series, effect on correlation ( 0.439) is:
Lower   1948>-0.039   2007>-0.031   1973<-0.015   1985>-0.013   1954<-0.011   2008<-0.010  Higher   1976 0.097   2016 0.019
==================================================================================================================================
WG71      1940 to  2022      83 years                                                                                    Series 232
[A] Segment   High   -10   -9   -8   -7   -6   -5   -4   -3   -2   -1    0   +1   +2   +3   +4   +5   +6   +7   +8   +9  +10
---------  ----   ---  ---  ---  ---  ---  ---  ---  ---  ---  ---  ---  ---  ---  ---  ---  ---  ---  ---  ---  ---  ---
1940 1989    4    .08  .03 -.01  .05  .00  .03  .04 -.05 -.13 -.07  .30|-.21 -.09 -.19  .42* .13 -.01 -.23  .21  .22 -.10
1950 1999    5    .07  .19 -.05  .09 -.08  .20 -.19 -.16 -.09  .17  .19|-.17 -.02 -.03  .22  .28*-.06 -.14 -.13  .27 -.03
1973 2022   -5    .03  .04 -.33  .01  .12  .39* .07 -.33 -.02  .08  .36|   -    -    -    -    -    -    -    -    -    -
[B] Entire series, effect on correlation ( 0.384) is:
Lower   1981<-0.052   1976>-0.048   1940<-0.023   1959>-0.016   1967<-0.013   1997<-0.010  Higher   1948 0.072   2016 0.014
1940 to 1989 segment:
Lower   1976>-0.065   1981<-0.064   1940<-0.025   1959>-0.021   1967<-0.016   1955<-0.011  Higher   1948 0.120   1943 0.016
1950 to 1999 segment:
Lower   1976>-0.079   1981<-0.059   1959>-0.029   1967<-0.017   1997<-0.015   1955<-0.014  Higher   1986 0.024   1996 0.022
1973 to 2022 segment:
Lower   1981<-0.115   1976>-0.090   1997<-0.020   1984<-0.011   2022<-0.010   2004>-0.009  Higher   2016 0.031   2011 0.025
==================================================================================================================================
```

```
WG72      1941 to  2022      82 years                                                                                    Series 233
[A] Segment   High   -10   -9   -8   -7   -6   -5   -4   -3   -2   -1    0   +1   +2   +3   +4   +5   +6   +7   +8   +9  +10
---------  ----   ---  ---  ---  ---  ---  ---  ---  ---  ---  ---  ---  ---  ---  ---  ---  ---  ---  ---  ---  ---  ---
1950 1999    0    .16  .06 -.25 -.17 -.08 -.09 -.05 -.07  .00  .10  .25*-.03  .00  .07  .17  .12  .24 -.10 -.15 -.04 -.05
1973 2022    0   -.05 -.07 -.46  .12  .25  .13  .06 -.21  .05 -.14  .29*   -    -    -    -    -    -    -    -    -    -
[B] Entire series, effect on correlation ( 0.348) is:
Lower   2002<-0.068   1976>-0.045   1973<-0.025   1995>-0.014   1958<-0.011   1941<-0.011  Higher   1944 0.030   1948 0.025
1950 to 1999 segment:
Lower   1976>-0.091   1973<-0.057   1995>-0.029   1958<-0.023   1950<-0.021   1963>-0.016  Higher   1960 0.039   1988 0.029
1973 to 2022 segment:
Lower   2002<-0.091   1976>-0.079   1973<-0.038   1995>-0.022   2004>-0.010   1982<-0.007  Higher   1988 0.023   2011 0.019
===================================================================================================================================
WG81      1963 to  2022      60 years                                                                                    Series 234
[B] Entire series, effect on correlation ( 0.369) is:
Lower   2016>-0.058   2004>-0.033   2000>-0.021   2015<-0.018   1984<-0.015   1975<-0.015  Higher   1996 0.050   2011 0.031
===================================================================================================================================
WG82      1958 to  2022      65 years                                                                                    Series 235
[A] Segment   High   -10   -9   -8   -7   -6   -5   -4   -3   -2   -1    0   +1   +2   +3   +4   +5   +6   +7   +8   +9  +10
---------  ----   ---  ---  ---  ---  ---  ---  ---  ---  ---  ---  ---  ---  ---  ---  ---  ---  ---  ---  ---  ---  ---
1958 2007    0    .20  .12 -.14  .00  .23  .20 -.28 -.06 -.09  .05  .30*-.07 -.08 -.21  .29  .10 -.05 -.22 -.22  .25  .15
1973 2022    0    .20 -.08 -.11  .00  .18  .26 -.23 -.16 -.13  .29  .30*   -    -    -    -    -    -    -    -    -    -
[B] Entire series, effect on correlation ( 0.255) is:
Lower   1976>-0.084   2011>-0.022   1960>-0.021   2016>-0.018   2012<-0.014   1980>-0.013  Higher   1996 0.043   2007 0.039
1958 to 2007 segment:
Lower   1976>-0.117   1960>-0.029   1980>-0.018   1984<-0.012   1990>-0.012   1966<-0.012  Higher   1996 0.047   1986 0.039
1973 to 2022 segment:
Lower   1976>-0.104   2011>-0.026   2016>-0.022   2012<-0.018   1980>-0.016   2008<-0.014  Higher   1996 0.047   1986 0.041
[E] Outliers     1   3.0 SD above or -4.5 SD below mean for year
2007 -5.4 SD
===================================================================================================================================
WG91      1940 to  2022      83 years                                                                                    Series 236
[A] Segment   High   -10   -9   -8   -7   -6   -5   -4   -3   -2   -1    0   +1   +2   +3   +4   +5   +6   +7   +8   +9  +10
---------  ----   ---  ---  ---  ---  ---  ---  ---  ---  ---  ---  ---  ---  ---  ---  ---  ---  ---  ---  ---  ---  ---
1940 1989    7   -.30 -.03 -.32  .10 -.06 -.06  .12  .27  .03 -.13  .16|-.08 -.26  .24 -.05 -.12 -.11  .41*-.19 -.08 -.17
1950 1999   -3   -.17 -.01 -.29  .02  .01  .05  .14  .25* .00 -.34  .20|-.09 -.18 -.07  .00 -.04 -.02  .21 -.13 -.02 -.12
[B] Entire series, effect on correlation ( 0.297) is:
Lower   1941<-0.030   1963<-0.022   1986>-0.020   1980>-0.019   1973<-0.014   2007>-0.010  Higher   2016 0.038   2011 0.016
1940 to 1989 segment:
Lower   1941<-0.038   1980>-0.025   1986>-0.025   1973<-0.020   1951<-0.013   1963<-0.011  Higher   1976 0.033   1944 0.028
1950 to 1999 segment:
Lower   1980>-0.036   1986>-0.036   1973<-0.024   1951<-0.015   1977>-0.015   1964<-0.011  Higher   1976 0.039   1955 0.025
===================================================================================================================================
WG92      1941 to  2022      82 years                                                                                    Series 237
[A] Segment   High   -10   -9   -8   -7   -6   -5   -4   -3   -2   -1    0   +1   +2   +3   +4   +5   +6   +7   +8   +9  +10
---------  ----   ---  ---  ---  ---  ---  ---  ---  ---  ---  ---  ---  ---  ---  ---  ---  ---  ---  ---  ---  ---  ---
1941 1990    0    .02  .03 -.28  .05 -.13  .15 -.04  .20 -.12  .02  .32*-.16 -.25 -.03  .22 -.09 -.11  .03 -.11 -.03 -.01
[B] Entire series, effect on correlation ( 0.440) is:
Lower   1980>-0.029   2007>-0.023   1960>-0.016   1994<-0.015   2000>-0.015   1973<-0.014  Higher   1944 0.033   2016 0.021
1941 to 1990 segment:
Lower   1980>-0.046   1945<-0.025   1973<-0.025   1960>-0.023   1951<-0.019   1972<-0.019  Higher   1944 0.093   1986 0.043
===================================================================================================================================
PART 7:  DESCRIPTIVE STATISTICS:                                                                     00:00   00     0000  Page  10
-----------------------------------------------------------------------------------------------------------------------------------
Corr   //-------- Unfiltered --------
//---- Filtered -----

No.    No.    No.    with   Mean   Max     Std   Auto   Mean   Max     Std   Auto  AR
Seq Series   Interval   Years  Segmt  Flags   Master  msmt   msmt    dev   corr   sens  value    dev   corr  ()
--- -------- ---------  -----  -----  -----   ------ -----  -----  -----  -----  -----  -----  -----  -----  --
1 L10011   1804 2005    202      8      0   0.565   1.93   3.87  0.921  0.747  0.330   2.54  0.317  0.021   2
2 L10012   1797 1807     11      1      0   0.740   2.56   3.99  0.757 -0.341  0.390   2.62  0.826 -0.024   1
3 L10012   1810 2013    204      8      0   0.650   1.91   4.67  0.852  0.680  0.345   2.48  0.398  0.009   2
4 L10021   1818 2018    201      8      0   0.595   2.17   6.70  1.047  0.767  0.286   2.69  0.441  0.011   1
5 L10022   1826 2019    194      7      2   0.483   2.53   7.41  1.045  0.765  0.260   2.55  0.360 -0.005   1
6 L10061   1799 2019    221      9      1   0.493   1.24   5.40  1.097  0.863  0.426   2.81  0.518 -0.003   1
7 L10062   1812 2019    208      8      1   0.559   1.34   4.18  1.002  0.830  0.399   2.71  0.405  0.002   1
8 L10071   1822 2018    197      8      0   0.615   1.66   5.68  0.967  0.760  0.368   2.47  0.334 -0.023   1
9 L10072   1803 2018    216      8      3   0.482   1.18   3.64  0.761  0.725  0.353   2.77  0.383 -0.009   1
10 L10081   1846 2020    175      7      0   0.629   1.01   2.55  0.557  0.616  0.435   2.73  0.433 -0.005   1
11 L10082   1808 2020    213      8      1   0.549   1.16   2.95  0.697  0.771  0.346   2.89  0.432  0.039   1
12 L10101   1825 2020    196      7      0   0.473   1.76   3.90  0.900  0.681  0.365   2.80  0.390  0.043   1
13 L10102   1814 2020    207      8      1   0.557   2.07   4.26  0.888  0.728  0.304   2.66  0.329 -0.011   1
14 L10131   1804 2018    215      8      0   0.634   2.04   4.97  1.119  0.834  0.309   2.59  0.346 -0.006   2
15 L10132   1881 2018    138      5      0   0.753   2.14   4.72  1.073  0.811  0.308   2.43  0.380  0.043   1
16 L10141   1809 2018    210      8      0   0.612   1.93   3.91  0.812  0.749  0.289   2.59  0.437 -0.012   3
17 L10142   1822 2017    196      8      0   0.540   2.02   4.48  0.979  0.708  0.340   2.61  0.438 -0.028   2
18 L10151   1796 2019    224      9      0   0.668   2.22   6.26  1.285  0.848  0.290   2.51  0.305 -0.013   1
19 L10152   1788 2019    232      9      0   0.672   2.36   7.40  1.210  0.786  0.264   2.49  0.341 -0.017   2
20 L10171   1847 2018    172      7      1   0.674   2.01   5.28  0.940  0.701  0.288   2.64  0.374  0.001   1
21 L10172   1834 2018    185      7      0   0.632   1.67   3.44  0.668  0.523  0.300   2.52  0.421 -0.041   1
22 L10181   1916 2019    104      4      0   0.682   2.19   5.01  0.837  0.301  0.395   2.63  0.375 -0.007   1
23 L10182   1800 2019    220      8      0   0.690   1.71   4.24  0.830  0.675  0.375   2.63  0.414 -0.022   1
24 L10183   1818 2019    202      8      0   0.699   1.97   4.48  0.799  0.663  0.296   2.55  0.395  0.000   1
25 L10331   1808 2018    211      8      0   0.706   2.34   8.88  1.274  0.739  0.338   2.68  0.372  0.005   1
26 L10332   1975 2018     44      1      0   0.693   1.92   4.20  0.996  0.332  0.554   2.60  0.529  0.024   1
27 L7221    1800 2001    202      8      0   0.671   2.10   7.52  1.767  0.877  0.316   2.54  0.324 -0.010   1
28 L7222    1794 1997    204      8      0   0.717   2.05   6.16  1.417  0.826  0.333   2.50  0.354 -0.042   1
29 L7231    1792 1995    204      8      2   0.506   1.99   4.50  0.913  0.766  0.249   2.46  0.295  0.009   1
30 L7271    1916 2003     88      4      2   0.371   1.25   3.07  0.714  0.541  0.508   2.70  0.524 -0.027   1
31 L7272    1815 1992    178      7      3   0.356   1.37   3.59  0.774  0.663  0.471   2.57  0.404  0.016   1
32 L7273    1798 2007    210      9      5   0.353   1.46   5.77  1.053  0.814  0.443   2.56  0.424  0.003   3
33 L7301    1809 2016    208      8      0   0.613   1.95   3.81  0.676  0.667  0.246   2.55  0.382 -0.017   1
```

Seq	Series	Interval	Years	Segmt	Flags	Master	msmt	msmt	dev	corr	sens	value	dev	corr	()
34	L7302	1799 2012	214	9	0	0.658	1.92	4.25	0.595	0.429	0.265	2.51	0.340	-0.027	1
35	SD1330	1792 2021	230	9	0	0.529	1.69	4.02	0.777	0.539	0.369	2.68	0.457	-0.004	1
36	SD1331	1804 2021	218	8	0	0.525	1.83	4.72	0.905	0.639	0.328	2.43	0.290	0.000	1
37	SD1401	1870 2021	152	6	0	0.468	4.34	9.75	2.141	0.878	0.177	2.47	0.309	0.008	1
38	SD1402	1845 2021	177	7	0	0.567	3.00	6.52	1.203	0.721	0.233	2.76	0.417	-0.012	1
39	SD1621	1786 2021	236	9	0	0.573	1.65	5.55	1.183	0.757	0.427	2.62	0.371	0.000	1
40	SD1622	1786 2021	236	9	1	0.487	1.73	6.13	1.210	0.735	0.437	2.64	0.340	0.065	1
41	SD1650	1804 2021	218	8	0	0.609	2.11	6.21	0.948	0.702	0.277	2.77	0.436	-0.038	2
42	SD1651	1798 2021	224	9	0	0.725	1.68	5.04	0.839	0.689	0.298	2.60	0.459	0.011	1
43	SD1720	1937 2021	85	3	0	0.666	3.24	6.22	1.588	0.676	0.356	2.51	0.467	-0.078	2
44	SD1721	1934 2021	88	3	0	0.719	2.45	7.34	1.560	0.795	0.350	2.61	0.414	-0.034	2
45	SD1750	1792 2021	230	9	0	0.645	1.90	4.41	0.679	0.503	0.269	2.56	0.431	-0.011	1
46	SD1751	1813 2021	209	8	0	0.651	1.77	3.53	0.615	0.527	0.285	2.45	0.320	0.003	1
47	SD1752	1899 2021	123	5	0	0.616	2.16	4.03	0.723	0.557	0.259	2.53	0.349	0.039	1
48	SD1753	1806 2021	216	8	0	0.561	2.41	5.32	0.870	0.594	0.273	2.58	0.410	-0.030	1

PART 7: DESCRIPTIVE STATISTICS: 00:00 00 0000 Page 11

Corr //-------- Unfiltered --------
//---- Filtered -----

No. Seq	No. Series	No. Interval	with Years	Mean Segmt	Max Flags	Std Master	Auto msmt	Mean msmt	Max dev	Std corr	Auto sens	AR value	dev	corr	()
49	SD1754	1805 2021	217	8	0	0.662	2.15	5.93	1.016	0.767	0.293	2.65	0.379	0.001	1
50	SD1755	1783 2021	239	9	0	0.592	1.84	5.53	1.385	0.820	0.378	2.46	0.298	-0.021	2
51	SD1920	1841 2021	181	7	0	0.687	2.17	4.78	0.861	0.599	0.300	2.71	0.417	-0.009	1
52	SD1921	1798 2021	224	9	1	0.661	2.23	5.40	0.944	0.576	0.311	2.68	0.375	0.004	1
53	SD1930	1835 2021	187	7	0	0.572	2.36	5.59	0.949	0.704	0.252	2.88	0.497	-0.015	1
54	SD1931	1798 2021	224	9	0	0.664	2.65	9.77	1.442	0.684	0.306	2.56	0.396	-0.003	1
55	SD1970	1774 2021	248	9	0	0.643	1.82	4.65	1.117	0.680	0.429	2.60	0.355	-0.022	2
56	SD1971	1788 2021	234	9	2	0.536	1.61	5.83	1.085	0.703	0.411	2.61	0.297	0.009	1
57	SD1990	1800 2021	222	8	0	0.497	1.71	4.90	0.920	0.787	0.306	2.68	0.385	-0.018	1
58	SD1991	1802 2021	220	8	0	0.565	1.76	4.33	1.060	0.808	0.300	2.58	0.317	-0.021	1
59	SD2210	1796 2021	226	9	0	0.619	2.15	6.58	1.461	0.827	0.360	2.60	0.395	0.056	1
60	SD2211	1800 2018	219	8	0	0.570	2.14	8.11	1.433	0.795	0.307	2.51	0.286	-0.025	2
61	SD2230	1776 2021	246	9	0	0.567	2.18	6.35	1.164	0.659	0.397	2.48	0.369	-0.037	1
62	SD2231	1798 2021	224	9	0	0.608	2.20	5.51	1.291	0.701	0.403	2.56	0.299	-0.018	2
63	SD2240	1795 2021	227	9	0	0.594	1.30	4.39	0.917	0.845	0.316	2.56	0.351	-0.002	1
64	SD2241	1779 2021	243	9	1	0.571	1.33	4.33	0.762	0.743	0.322	2.64	0.255	-0.043	1
65	SD2311	1777 2021	245	9	0	0.704	1.71	4.67	0.898	0.734	0.361	2.47	0.339	-0.011	1
66	SD2312	1794 2021	228	9	0	0.687	2.00	4.85	0.956	0.678	0.331	2.54	0.312	-0.061	1
67	SD2501	1825 2021	197	7	1	0.535	1.55	6.46	1.147	0.864	0.337	2.52	0.334	0.017	1
68	SD2502	1797 2021	225	9	1	0.498	2.57	8.64	1.522	0.847	0.321	2.55	0.328	-0.015	1
69	SD2560	1802 2021	220	8	0	0.650	1.36	4.79	0.887	0.790	0.380	2.58	0.340	-0.040	1
70	SD2561	1813 2021	209	8	0	0.644	1.85	4.78	1.063	0.640	0.416	2.49	0.355	-0.013	1
71	SD2580	1804 2021	218	8	1	0.604	1.91	5.01	0.934	0.620	0.329	2.57	0.418	0.018	1
72	SD2581	1792 2021	230	9	2	0.517	1.96	6.56	1.120	0.656	0.371	2.59	0.494	-0.045	2
73	SD2760	1793 2021	229	9	3	0.466	1.28	4.46	0.957	0.726	0.415	2.62	0.407	-0.014	1
74	SD2761	1788 2021	234	9	3	0.382	1.34	4.83	1.022	0.769	0.396	2.60	0.381	-0.076	2
75	SD2780	1816 2021	206	8	0	0.605	2.33	5.52	0.907	0.673	0.261	2.54	0.450	-0.042	1
76	SD2781	1833 2014	182	7	0	0.673	2.14	4.77	0.889	0.663	0.274	2.68	0.414	0.018	1
77	SD2791	1804 2021	218	8	0	0.532	1.22	4.24	0.919	0.876	0.339	2.68	0.435	-0.018	2
78	SD2792	1833 2021	189	7	0	0.600	1.95	4.85	1.032	0.820	0.273	2.47	0.299	-0.048	2
79	SD2801	1824 2021	198	8	0	0.603	1.31	4.23	0.672	0.720	0.317	2.70	0.390	-0.004	1
80	SD2802	1823 2021	199	8	1	0.466	1.58	5.00	0.882	0.855	0.252	2.55	0.369	-0.054	1
81	SD2840	1827 2021	195	7	0	0.629	2.51	5.47	1.105	0.697	0.323	2.48	0.371	-0.010	1
82	SD2841	1822 2021	200	8	0	0.706	1.94	3.89	0.729	0.558	0.306	2.52	0.317	-0.026	1
83	SD2851	1795 2021	227	9	0	0.625	1.78	5.25	1.142	0.746	0.402	2.52	0.298	0.018	1
84	SD2852	1815 2021	207	8	0	0.578	2.17	6.41	1.119	0.751	0.332	2.53	0.382	-0.053	2
85	SD2930	1833 2021	189	7	0	0.612	2.34	4.73	0.821	0.564	0.269	2.46	0.333	0.010	1
86	SD2931	1805 2021	217	8	0	0.609	2.21	4.36	0.832	0.653	0.288	2.57	0.408	-0.026	1
87	SD3771	1818 2021	204	8	5	0.189	0.96	7.31	0.868	0.761	0.319	2.73	0.383	-0.075	1
88	SD3772	1785 2021	237	9	7	0.231	0.94	5.04	0.852	0.747	0.415	2.56	0.305	-0.063	1
89	SD4361	1846 2021	176	7	0	0.621	1.53	4.84	1.146	0.849	0.315	2.43	0.291	-0.015	1
90	SD4362	1878 2021	144	5	1	0.535	1.80	5.18	1.203	0.831	0.299	2.68	0.432	0.007	1
91	SD4521	1813 2021	209	8	0	0.639	1.70	4.61	0.972	0.844	0.280	2.52	0.391	-0.012	2
92	SD4522	1797 2021	225	9	0	0.622	1.76	4.82	0.928	0.618	0.364	2.70	0.457	-0.038	1
93	SD4531	1829 2021	193	7	2	0.399	1.83	5.30	1.215	0.777	0.384	2.60	0.412	-0.021	2
94	SD4532	1871 2021	151	6	1	0.499	2.23	4.37	0.863	0.478	0.366	2.61	0.353	0.018	1
95	SD4551	1819 2017	199	8	0	0.721	2.31	5.59	1.027	0.676	0.267	2.60	0.403	-0.037	1
96	SD4581	1797 2017	221	9	4	0.387	1.38	6.32	1.337	0.835	0.361	2.53	0.368	-0.040	1

PART 7: DESCRIPTIVE STATISTICS: 00:00 00 0000 Page 12

Corr //-------- Unfiltered --------
//---- Filtered -----

No. Seq	No. Series	No. Interval	with Years	Mean Segmt	Max Flags	Std Master	Auto msmt	Mean msmt	Max dev	Std corr	Auto sens	AR value	dev	corr	()
97	SD5341	1803 2017	215	8	2	0.483	1.83	4.39	0.845	0.658	0.334	2.72	0.416	-0.031	1
98	SD6311	1856 2016	161	6	0	0.700	3.20	5.91	1.271	0.701	0.289	2.56	0.410	0.016	1
99	SD6681	1851 2021	171	6	0	0.773	2.01	4.30	0.897	0.717	0.312	2.44	0.353	-0.017	1
100	SD6682	1848 2021	174	7	0	0.693	1.65	4.73	0.804	0.741	0.319	2.61	0.376	0.011	1
101	SD7221	1795 2021	227	9	3	0.487	1.73	5.02	0.870	0.705	0.289	2.46	0.389	0.033	1
102	SD7222	1780 2021	242	9	0	0.648	1.56	3.55	0.652	0.574	0.282	2.54	0.409	-0.004	1
103	SD81	1873 2021	149	6	0	0.661	1.97	7.00	1.092	0.875	0.220	2.61	0.349	-0.055	1
104	SD82	1841 2021	181	7	1	0.568	2.48	5.74	1.003	0.793	0.212	2.50	0.395	-0.013	1
105	SD83	1843 2017	175	7	0	0.570	2.47	4.63	0.833	0.738	0.205	2.45	0.313	-0.035	1
106	SD8601	1777 2017	241	9	6	0.349	0.90	3.95	0.712	0.835	0.301	2.71	0.318	0.007	1
107	SD9531	1811 2021	211	8	2	0.465	2.40	4.38	0.777	0.493	0.276	2.47	0.313	-0.056	2
108	SD9532	1849 2021	173	7	0	0.565	2.71	5.52	0.901	0.460	0.297	2.51	0.408	-0.046	1
109	NG1070	1941 2021	81	3	0	0.494	2.42	7.03	1.311	0.818	0.294	2.61	0.395	0.003	1

Seq	Series	Interval	Years	Segmt	Flags	Corr with Master	Mean msmt	Max msmt	Std dev	Auto corr	Mean sens	Max value	Std dev	Auto corr	AR ()
110	NG1071	1941 2021	81	3	0	0.566	2.29	5.49	0.824	0.672	0.228	2.74	0.530	-0.070	1
111	NG1110	1956 2021	66	2	0	0.372	2.51	4.24	0.984	0.904	0.168	2.51	0.408	-0.049	2
112	NG1111	1965 2021	57	2	2	-0.001	2.93	5.34	1.374	0.863	0.259	2.74	0.569	0.018	1
113	NG1120	1952 2021	70	2	1	0.349	2.30	4.58	1.126	0.774	0.300	2.98	0.630	-0.001	2
114	NG1121	1943 2021	79	3	2	0.341	1.84	4.18	0.923	0.692	0.354	2.63	0.574	0.047	1
115	NG1180	1961 2021	61	2	2	0.199	2.16	3.28	0.653	0.575	0.224	2.53	0.513	-0.006	1
116	NG1181	1963 2021	59	2	2	0.185	1.93	3.14	0.595	0.572	0.234	2.47	0.425	-0.044	1
117	NG1182	1963 2021	59	2	2	0.291	2.23	3.68	0.729	0.501	0.294	2.54	0.502	-0.085	2
118	NG1183	1968 2021	54	2	1	0.331	2.21	3.48	0.677	0.615	0.253	2.55	0.500	0.028	1
119	NG1190	1964 2021	58	2	0	0.525	2.23	4.57	1.168	0.824	0.327	2.74	0.637	-0.008	1
120	NG1191	1957 2021	65	2	0	0.436	2.34	4.44	0.985	0.807	0.228	2.82	0.539	-0.044	1
121	NG1280	1971 2021	51	2	2	0.322	1.82	5.75	0.830	0.630	0.275	2.49	0.393	0.057	1
122	NG1281	1972 2021	50	1	1	0.258	1.75	4.10	0.944	0.665	0.346	2.42	0.426	-0.026	1
123	NG1282	1962 2022	61	2	2	0.272	1.49	3.54	0.781	0.674	0.357	2.66	0.458	-0.024	1
124	NG1283	1976 2022	47	1	0	0.333	2.17	4.30	0.922	0.726	0.296	2.68	0.648	0.191	1
125	NG1290	1961 2022	62	2	0	0.551	3.65	5.72	1.090	0.798	0.148	2.61	0.528	0.023	1
126	NG1291	1965 2022	58	2	0	0.519	3.72	6.82	1.107	0.786	0.167	2.56	0.440	-0.041	1
127	NG1320	1964 2021	58	2	0	0.539	2.57	4.72	0.828	0.690	0.203	2.56	0.551	0.004	1
128	NG1321	1966 2021	56	2	1	0.219	2.98	4.88	0.894	0.709	0.186	2.54	0.428	0.007	2
129	NG1400	1968 2022	55	2	2	0.168	3.84	6.16	0.858	0.412	0.178	2.50	0.461	-0.024	1
130	NG1401	1967 2022	56	2	1	0.372	3.24	4.80	0.999	0.802	0.179	2.37	0.384	-0.007	1
131	NG1420	1968 2022	55	2	2	0.307	2.30	8.17	1.908	0.902	0.380	2.83	0.496	0.001	2
132	NG1421	1969 2022	54	2	0	0.494	2.59	8.30	1.946	0.892	0.232	2.38	0.440	-0.024	1
133	NG1422	1969 2022	54	2	0	0.411	2.91	7.20	1.803	0.929	0.205	2.75	0.675	0.086	2
134	NG1423	1971 2022	52	2	0	0.372	2.46	6.15	1.769	0.930	0.293	2.73	0.598	-0.051	1
135	NG1430	1968 2022	55	2	0	0.487	4.53	7.86	1.454	0.823	0.165	2.59	0.511	-0.044	1
136	NG1431	1958 2022	65	2	1	0.325	4.51	8.68	1.865	0.886	0.176	2.95	0.655	0.002	1
137	NG1440	1959 2022	64	2	2	0.276	2.58	4.67	0.957	0.851	0.184	2.52	0.415	0.062	1
138	NG1441	1968 2022	55	2	2	0.322	3.07	5.57	0.833	0.747	0.149	2.61	0.493	-0.017	1
139	NG1470	1975 2022	48	1	1	0.158	3.49	6.75	1.237	0.781	0.199	2.56	0.538	-0.123	2
140	NG1471	1966 2022	57	2	2	0.268	3.44	8.06	1.398	0.846	0.179	2.59	0.413	0.066	1
141	NG151	1968 2017	50	1	1	0.202	2.26	4.50	1.136	0.799	0.247	2.51	0.562	0.014	2
142	NG1580	1962 2021	60	2	2	0.269	2.70	6.29	1.444	0.810	0.285	2.57	0.554	0.122	1
143	NG1581	1967 2021	55	2	2	0.189	2.28	5.42	1.243	0.816	0.323	2.45	0.440	-0.032	1
144	NG2640	1958 2021	64	2	0	0.409	1.99	3.96	0.852	0.669	0.275	2.40	0.375	-0.005	1

Corr //-------- Unfiltered --------
//---- Filtered -----

No. Seq	No. Series	No. Interval	with Years	Mean Segmt	Max Flags	Std Master	Auto msmt	Mean msmt	Max dev	Std corr	Auto sens	AR value	dev	corr	()
145	NG2641	1959 2021	63	2	0	0.443	2.24	3.50	0.753	0.749	0.197	2.33	0.383	0.055	3
146	NG2650	1952 2021	70	2	1	0.198	1.73	4.14	1.078	0.828	0.356	2.49	0.436	-0.049	2
147	NG2651	1962 2021	60	2	0	0.401	1.75	3.80	0.815	0.735	0.283	2.46	0.437	0.098	2
148	NG2710	1968 2021	54	2	0	0.344	2.63	6.96	0.976	0.622	0.232	2.71	0.442	0.012	2
149	NG2711	1967 2021	55	2	0	0.397	1.60	3.52	0.738	0.648	0.376	2.65	0.586	-0.052	2
150	NG2712	1970 2021	52	2	0	0.336	2.73	4.31	0.805	0.416	0.277	2.60	0.521	-0.027	2
151	NG2713	1965 2021	57	2	0	0.478	3.07	5.34	1.107	0.590	0.270	2.73	0.546	0.086	1
152	NG2800	1967 2021	55	2	1	0.321	2.42	5.35	1.033	0.798	0.260	2.56	0.469	-0.057	1
153	NG2801	1958 2021	64	2	1	0.394	2.17	4.46	1.149	0.827	0.310	2.63	0.482	-0.070	1
154	NG2900	1948 2021	74	3	3	0.188	2.23	6.39	1.496	0.876	0.265	3.18	0.575	0.039	1
155	NG2901	1960 2021	62	2	0	0.293	2.61	6.09	1.420	0.805	0.263	2.89	0.551	0.098	1
156	NG2940	1957 2021	65	2	2	0.169	1.98	4.26	1.060	0.881	0.274	2.45	0.402	0.007	1
157	NG2941	1957 2020	64	2	1	0.253	1.87	3.89	0.964	0.823	0.273	2.64	0.423	0.022	1
158	NG3020	1955 2021	67	2	0	0.420	2.63	5.42	1.268	0.791	0.285	2.45	0.387	0.089	1
159	NG3021	1951 2021	71	2	0	0.370	2.73	5.22	0.986	0.697	0.244	2.54	0.443	0.099	1
160	NG3040	1960 2021	62	2	1	0.230	2.13	3.78	0.792	0.555	0.314	2.52	0.450	0.002	1
161	NG3041	1972 2021	50	1	1	0.173	1.79	3.68	0.828	0.708	0.352	2.52	0.510	-0.044	1
162	NG3051	1960 2021	62	2	0	0.381	2.50	6.54	1.498	0.851	0.297	2.45	0.404	-0.085	1
163	NG3052	1953 2021	69	2	0	0.370	2.77	6.68	1.475	0.833	0.317	2.33	0.330	-0.034	1
164	NG3101	1951 2021	71	2	1	0.262	3.36	6.25	1.835	0.925	0.209	2.64	0.413	0.113	1
165	NG3102	1956 2021	66	2	0	0.416	3.18	6.15	1.678	0.880	0.263	2.50	0.447	0.024	1
166	NG3571	1983 2021	39	1	0	0.374	2.71	4.82	1.113	0.702	0.246	2.53	0.570	-0.029	1
167	NG3572	1991 2021	31	1	1	0.274	3.26	6.21	1.790	0.844	0.308	2.51	0.510	-0.012	1
168	NG3581	1970 2021	52	2	0	0.363	4.66	8.21	1.880	0.836	0.189	2.73	0.686	0.056	1
169	NG3582	1975 2021	47	1	1	0.187	4.33	7.69	1.751	0.733	0.290	2.60	0.626	0.017	1
170	NG4141	1946 2020	75	3	1	0.507	1.68	3.33	0.811	0.719	0.338	2.67	0.500	-0.046	1
171	NG4142	1950 2021	72	2	0	0.367	1.61	3.65	0.812	0.664	0.335	2.87	0.556	0.037	1
172	WG11	1964 2022	59	2	0	0.521	4.36	6.32	0.992	0.726	0.142	2.57	0.550	0.101	1
173	WG12	1968 2022	55	2	0	0.380	3.96	5.71	0.818	0.492	0.167	2.73	0.554	0.079	1
174	WG101	1964 2022	59	2	0	0.307	4.21	7.99	1.731	0.822	0.178	2.63	0.560	0.025	1
175	WG102	1961 2022	62	2	2	0.206	3.93	7.29	1.666	0.789	0.225	2.54	0.474	-0.027	1
176	WG111	1928 2022	95	3	0	0.398	1.94	5.43	1.120	0.671	0.377	2.80	0.536	-0.007	1
177	WG112	1933 2022	90	3	1	0.360	2.05	4.63	1.050	0.746	0.320	2.79	0.545	0.009	1
178	WG121	1962 2022	61	2	1	0.321	3.14	5.44	0.999	0.580	0.232	2.57	0.378	-0.013	2
179	WG122	1973 2022	50	1	0	0.421	3.35	5.02	0.952	0.505	0.253	2.53	0.465	0.036	4
180	WG131	1966 2022	57	2	0	0.430	4.17	6.59	1.106	0.570	0.207	2.37	0.360	-0.083	2
181	WG132	1959 2022	64	2	1	0.435	3.61	6.01	1.021	0.601	0.210	2.38	0.376	-0.042	2
182	WG141	1971 2022	52	2	2	0.191	3.26	5.61	1.041	0.704	0.217	2.55	0.557	0.017	1
183	WG142	1963 2022	60	2	2	0.223	4.27	7.41	1.517	0.773	0.231	2.67	0.558	0.006	1
184	WG151	1959 2022	64	2	0	0.418	3.61	5.58	0.839	0.568	0.178	2.43	0.319	-0.006	1
185	WG152	1961 2022	62	2	1	0.329	3.56	5.38	0.673	0.350	0.175	2.56	0.431	-0.042	1
186	WG161	1971 2022	52	2	2	0.197	3.41	5.16	0.681	0.625	0.129	2.74	0.568	-0.047	1
187	WG162	1963 2022	60	2	0	0.433	3.44	5.04	0.745	0.564	0.170	2.67	0.581	-0.030	1
188	WG171	1957 2022	66	2	1	0.399	2.67	5.35	1.026	0.587	0.278	2.86	0.585	-0.019	1
189	WG172	1963 2022	60	2	0	0.403	2.43	4.73	1.150	0.705	0.335	2.69	0.467	-0.023	1
190	WG181	1927 2022	96	3	0	0.611	2.10	4.53	0.971	0.497	0.415	2.52	0.312	-0.013	1
191	WG182	1921 2022	102	4	3	0.447	2.68	6.19	1.340	0.737	0.342	2.70	0.611	0.006	2
192	WG191	1964 2022	59	2	2	0.350	2.81	4.47	0.874	0.744	0.195	2.52	0.376	-0.040	2

Corr //-------- Unfiltered --------
//---- Filtered -----

No. Seq	No. Series	No. Interval	with Years	Mean Segmt	Max Flags	Std Master	Auto msmt	Mean msmt	Max dev	Std corr	Auto sens	AR value	dev	corr	()
193	WG192	1967 2022	56	2	0	0.390	2.90	5.55	1.227	0.851	0.209	2.45	0.446	-0.034	2
194	WG21	1959 2022	64	2	2	0.074	2.77	5.99	1.309	0.844	0.230	2.51	0.464	0.016	1
195	WG22	1959 2022	64	2	1	0.378	3.18	5.86	1.053	0.769	0.163	2.53	0.524	0.010	3
196	WG201	1972 2022	51	2	0	0.521	2.84	4.59	0.955	0.721	0.200	2.50	0.432	0.004	1
197	WG202	1960 2022	63	2	1	0.382	2.53	4.11	0.681	0.644	0.178	2.59	0.488	-0.039	1
198	WG211	1957 2022	66	2	2	0.166	2.81	5.13	1.156	0.678	0.264	2.57	0.406	-0.003	1
199	WG212	1957 2022	66	2	0	0.407	3.52	6.06	1.333	0.845	0.190	2.49	0.511	0.019	1
200	WG221	1927 2022	96	3	1	0.438	3.32	8.85	1.841	0.661	0.368	2.60	0.417	-0.009	1
201	WG222	1946 2022	77	3	0	0.508	3.31	8.50	1.757	0.534	0.358	2.55	0.441	0.015	1
202	WG231	1961 2022	62	2	0	0.585	2.87	4.54	0.733	0.426	0.226	2.53	0.493	0.035	1
203	WG232	1962 2022	61	2	0	0.567	2.94	4.90	0.793	0.636	0.198	2.44	0.470	0.030	1
204	WG241	1916 2022	107	4	3	0.331	1.95	4.31	0.738	0.691	0.271	2.47	0.463	-0.004	2
205	WG242	1914 2022	109	4	2	0.404	2.27	4.25	0.778	0.644	0.250	2.44	0.393	-0.081	2
206	WG251	1954 2022	69	2	1	0.384	2.53	5.60	1.093	0.876	0.181	2.48	0.469	-0.056	1
207	WG252	1938 2022	85	3	2	0.383	1.82	4.26	0.805	0.757	0.270	2.46	0.394	-0.079	4
208	WG261	1962 2022	61	2	2	0.138	2.55	5.13	1.114	0.737	0.335	2.69	0.640	-0.045	1
209	WG262	1964 2022	59	2	2	0.228	2.70	5.81	1.337	0.847	0.281	2.54	0.514	-0.080	1
210	WG271	1953 2022	70	2	2	0.289	2.22	4.83	0.901	0.651	0.249	2.84	0.683	0.073	2
211	WG272	1954 2022	69	2	1	0.385	2.10	3.93	0.654	0.567	0.206	2.93	0.540	0.163	1
212	WG281	1960 2022	63	2	0	0.527	2.56	4.51	0.713	0.807	0.131	2.58	0.441	-0.071	1
213	WG282	1964 2022	59	2	1	0.266	2.19	4.08	0.746	0.806	0.142	2.66	0.465	-0.063	1
214	WG291	1920 2022	103	4	1	0.427	2.79	5.64	1.340	0.768	0.275	2.63	0.490	-0.052	2
215	WG292	1933 2022	90	3	0	0.474	2.66	6.18	1.073	0.646	0.276	2.79	0.523	-0.036	1
216	WG31	1967 2022	56	2	0	0.435	3.97	6.02	1.092	0.614	0.197	2.59	0.407	0.017	3
217	WG32	1961 2022	62	2	0	0.361	3.52	5.95	1.163	0.752	0.189	2.61	0.500	0.130	1
218	WG301	1969 2022	54	2	1	0.330	4.39	5.63	0.755	0.333	0.162	2.29	0.335	-0.015	1
219	WG302	1960 2022	63	2	1	0.422	4.15	7.84	1.298	0.710	0.212	2.46	0.447	-0.042	1
220	WG311	1964 2022	59	2	1	0.323	3.11	5.45	0.905	0.794	0.172	2.52	0.458	0.053	1
221	WG312	1963 2022	60	2	0	0.325	3.49	4.82	0.861	0.762	0.142	2.52	0.436	-0.003	1
222	WG321	1967 2022	56	2	2	0.273	3.75	7.39	1.605	0.717	0.295	2.44	0.452	-0.026	2
223	WG322	1959 2022	64	2	2	0.182	3.28	6.52	1.354	0.749	0.276	2.40	0.401	0.086	1
224	WG331	1942 2022	81	3	1	0.205	3.33	6.10	1.261	0.737	0.246	2.39	0.358	0.031	1
225	WG332	1945 2022	78	3	1	0.379	3.00	5.79	1.446	0.842	0.272	2.37	0.413	-0.071	2
226	WG41	1963 2022	60	2	1	0.344	3.56	5.65	0.994	0.397	0.247	2.46	0.349	0.055	1
227	WG42	1961 2022	62	2	0	0.315	3.37	7.67	1.195	0.596	0.259	2.61	0.402	-0.063	2
228	WG51	1958 2022	65	2	0	0.412	2.38	4.95	1.017	0.796	0.252	2.46	0.502	0.007	2
229	WG52	1965 2022	58	2	1	0.267	2.17	4.38	0.880	0.790	0.280	2.51	0.361	-0.003	1
230	WG61	1925 2022	98	3	0	0.615	2.35	5.05	1.055	0.765	0.263	2.78	0.423	-0.024	1
231	WG62	1933 2022	90	3	0	0.439	3.02	6.43	1.277	0.806	0.230	2.63	0.426	-0.037	1
232	WG71	1940 2022	83	3	3	0.384	2.77	5.87	1.420	0.862	0.256	2.55	0.477	-0.001	1
233	WG72	1941 2022	82	3	2	0.348	3.18	7.21	1.758	0.899	0.249	2.37	0.339	-0.034	3
234	WG81	1963 2022	60	2	0	0.369	2.23	6.11	1.178	0.777	0.352	2.64	0.526	0.014	3
235	WG82	1958 2022	65	2	2	0.255	2.86	6.25	1.275	0.744	0.297	2.61	0.407	0.127	1
236	WG91	1940 2022	83	3	2	0.297	3.28	6.78	1.425	0.687	0.247	2.50	0.436	-0.043	1
237	WG92	1941 2022	82	3	1	0.440	3.46	6.94	1.539	0.737	0.246	2.70	0.582	0.023	1
Total or mean:			29807	1103	187	0.517	2.15	9.77	1.034	0.716	0.307	3.18	0.404	-0.009	

- = [COFECHA ALL COF] = -

Data S2: COFECHA output of tree ring width from the old trees. The dating statistics were calculated by COFECHA for all trees older than 240 years. This is sections 1 to 5 of the raw output of the program using the default settings.

```
[]  Dendrochronology Program Library                                        Run OLD    Program COF  00:00   00     0000  Page   1
[]
[]  P R O G R A M      C O F E C H A                                                                       Version 6.06P      -30
------------------------------------------------------------------------------------------------------------------------------------
QUALITY CONTROL AND DATING CHECK OF TREE-RING MEASUREMENTS
File of DATED series:   TRWOLD.rwl
CONTENTS:
Part 1:  Title page, options selected, summary, absent rings by series
Part 2:  Histogram of time spans
Part 3:  Master series with sample depth and absent rings by year
Part 4:  Bar plot of Master Dating Series
Part 5:  Correlation by segment of each series with Master
Part 6:  Potential problems: low correlation, divergent year-to-year changes, absent rings, outliers
Part 7:  Descriptive statistics
RUN CONTROL OPTIONS SELECTED                             VALUE
1  Cubic smoothing spline 50                                                           32 years
2  Segments examined are                       50 years lagged successively by  25 years
3  Autoregressive model applied                    A  Residuals are used in master dating series and testing
4  Series transformed to logarithms                Y  Each series log-transformed for master dating series and testing
5  CORRELATION is Pearson (parametric, quantitative)
Critical correlation, 99        6  Master dating series saved                      N
7  Ring measurements listed                        N
8  Parts printed                              1234567
9  Absent rings are omitted from master series and segment correlations  (Y)
Time span of Master dating series is  1774 to  2021   248 years
Continuous time span is               1774 to  2021   248 years
Portion with two or more series is    1776 to  2021   246 years
****************************************
*C* Number of dated series       108 *C*
*O* Master series 1774 2021  248 yrs *O*
*F* Total rings in all series  21470 *F*
*E* Total dated rings checked  21468 *E*
*C* Series intercorrelation    0.602 *C*
*H* Average mean sensitivity   0.328 *H*
*A* Segments, possible problems   72 *A*
*** Mean length of series      198.8 ***
****************************************
ABSENT RINGS listed by SERIES:            (See Master Dating Series for absent rings listed by year)
No ring measurements of zero value
PART 2:  TIME PLOT OF TREE-RING SERIES:                                                       00:00   00     0000  Page   2
------------------------------------------------------------------------------------------------------------------------------------
1050 1100 1150 1200 1250 1300 1350 1400 1450 1500 1550 1600 1650 1700 1750 1800 1850 1900 1950 2000 2050 Ident     Seq Time-span  Yrs
:    :    :    :    :    :    :    :    :    :    :    :    :    :    :    :    :    :    :    :    :    -------- --- ---- ---- ----
.    .    .    .    .    .    .    .    .    .    .    .    .    .    .    <===================>    . L10011     1 1804 2005  202
.    .    .    .    .    .    .    .    .    .    .    .    .    .    .   <>    .    .    .    .    . L10012     2 1797 1807   11
.    .    .    .    .    .    .    .    .    .    .    .    .    .    .    .<===================>   . L10012     3 1810 2013  204
.    .    .    .    .    .    .    .    .    .    .    .    .    .    .    .<===================>   . L10021     4 1818 2018  201
.    .    .    .    .    .    .    .    .    .    .    .    .    .    .    . <==================>   . L10022     5 1826 2019  194
.    .    .    .    .    .    .    .    .    .    .    .    .    .    .   <=====================>   . L10061     6 1799 2019  221
.    .    .    .    .    .    .    .    .    .    .    .    .    .    .    .<===================>   . L10062     7 1812 2019  208
.    .    .    .    .    .    .    .    .    .    .    .    .    .    .    . <==================>   . L10071     8 1822 2018  197
.    .    .    .    .    .    .    .    .    .    .    .    .    .    .    <====================>   . L10072     9 1803 2018  216
.    .    .    .    .    .    .    .    .    .    .    .    .    .    .    .   <=================>  . L10081    10 1846 2020  175
.    .    .    .    .    .    .    .    .    .    .    .    .    .    .    <=====================>  . L10082    11 1808 2020  213
.    .    .    .    .    .    .    .    .    .    .    .    .    .    .    . <===================>  . L10101    12 1825 2020  196
.    .    .    .    .    .    .    .    .    .    .    .    .    .    .    .<====================>  . L10102    13 1814 2020  207
.    .    .    .    .    .    .    .    .    .    .    .    .    .    .    <====================>   . L10131    14 1804 2018  215
.    .    .    .    .    .    .    .    .    .    .    .    .    .    .    .    .  <============>   . L10132    15 1881 2018  138
.    .    .    .    .    .    .    .    .    .    .    .    .    .    .    <====================>   . L10141    16 1809 2018  210
.    .    .    .    .    .    .    .    .    .    .    .    .    .    .    . <==================>   . L10142    17 1822 2017  196
.    .    .    .    .    .    .    .    .    .    .    .    .    .    .   <=====================>   . L10151    18 1796 2019  224
.    .    .    .    .    .    .    .    .    .    .    .    .    .    .  <======================>   . L10152    19 1788 2019  232
.    .    .    .    .    .    .    .    .    .    .    .    .    .    .    .   <================>   . L10171    20 1847 2018  172
.    .    .    .    .    .    .    .    .    .    .    .    .    .    .    .  <=================>   . L10172    21 1834 2018  185
.    .    .    .    .    .    .    .    .    .    .    .    .    .    .    .    .    .<=========>   . L10181    22 1916 2019  104
.    .    .    .    .    .    .    .    .    .    .    .    .    .    .    <====================>   . L10182    23 1800 2019  220
.    .    .    .    .    .    .    .    .    .    .    .    .    .    .    .<===================>   . L10183    24 1818 2019  202
.    .    .    .    .    .    .    .    .    .    .    .    .    .    .    <====================>   . L10331    25 1808 2018  211
.    .    .    .    .    .    .    .    .    .    .    .    .    .    .    .    .    .    . <===>   . L10332    26 1975 2018   44
.    .    .    .    .    .    .    .    .    .    .    .    .    .    .    <===================>    . L7221     27 1800 2001  202
.    .    .    .    .    .    .    .    .    .    .    .    .    .    .   <===================>.    . L7222     28 1794 1997  204
.    .    .    .    .    .    .    .    .    .    .    .    .    .    .   <===================>.    . L7231     29 1792 1995  204
.    .    .    .    .    .    .    .    .    .    .    .    .    .    .    .    .    .<========>    . L7271     30 1916 2003   88
.    .    .    .    .    .    .    .    .    .    .    .    .    .    .    .<=================>.    . L7272     31 1815 1992  178
.    .    .    .    .    .    .    .    .    .    .    .    .    .    .   <====================>    . L7273     32 1798 2007  210
.    .    .    .    .    .    .    .    .    .    .    .    .    .    .    <====================>   . L7301     33 1809 2016  208
.    .    .    .    .    .    .    .    .    .    .    .    .    .    .   <=====================>   . L7302     34 1799 2012  214
.    .    .    .    .    .    .    .    .    .    .    .    .    .    .   <======================>  . SD1330    35 1792 2021  230
.    .    .    .    .    .    .    .    .    .    .    .    .    .    .    <=====================>  . SD1331    36 1804 2021  218
.    .    .    .    .    .    .    .    .    .    .    .    .    .    .    .    . <==============>  . SD1401    37 1870 2021  152
.    .    .    .    .    .    .    .    .    .    .    .    .    .    .    .   <=================>  . SD1402    38 1845 2021  177
.    .    .    .    .    .    .    .    .    .    .    .    .    .    .  <=======================>  . SD1621    39 1786 2021  236
.    .    .    .    .    .    .    .    .    .    .    .    .    .    .  <=======================>  . SD1622    40 1786 2021  236
.    .    .    .    .    .    .    .    .    .    .    .    .    .    .    <=====================>  . SD1650    41 1804 2021  218
.    .    .    .    .    .    .    .    .    .    .    .    .    .    .   <======================>  . SD1651    42 1798 2021  224
.    .    .    .    .    .    .    .    .    .    .    .    .    .    .    .    .    .  <========>  . SD1720    43 1937 2021   85
.    .    .    .    .    .    .    .    .    .    .    .    .    .    .    .    .    .  <========>  . SD1721    44 1934 2021   88
```

```
.    .    .    .    .    .    .    .    .    .    .    .    .    .    .   <======================>  . SD1750    45 1792 2021  230
.    .    .    .    .    .    .    .    .    .    .    .    .    .    .    .<====================>  . SD1751    46 1813 2021  209
.    .    .    .    .    .    .    .    .    .    .    .    .    .    .    .    .   <============>  . SD1752    47 1899 2021  123
.    .    .    .    .    .    .    .    .    .    .    .    .    .    .    <=====================>  . SD1753    48 1806 2021  216
.    .    .    .    .    .    .    .    .    .    .    .    .    .    .    <=====================>  . SD1754    49 1805 2021  217
.    .    .    .    .    .    .    .    .    .    .    .    .    .    .  <=======================>  . SD1755    50 1783 2021  239
:    :    :    :    :    :    :    :    :    :    :    :    :    :    :    :    :    :    :    :    :
1050 1100 1150 1200 1250 1300 1350 1400 1450 1500 1550 1600 1650 1700 1750 1800 1850 1900 1950 2000 2050
PART 2:  TIME PLOT OF TREE-RING SERIES:                                                 00:00   00     0000  Page   3
-----------------------------------------------------------------------------------------------------------------------------------
1050 1100 1150 1200 1250 1300 1350 1400 1450 1500 1550 1600 1650 1700 1750 1800 1850 1900 1950 2000 2050 Ident   Seq Time-span  Yrs
:    :    :    :    :    :    :    :    :    :    :    :    :    :    :    :    :    :    :    :    :    : -------- --- ---- ---- ----
.    .    .    .    .    .    .    .    .    .    .    .    .    .    .    .   <=================>  . SD1920    51 1841 2021  181
.    .    .    .    .    .    .    .    .    .    .    .    .    .    .   <======================>  . SD1921    52 1798 2021  224
.    .    .    .    .    .    .    .    .    .    .    .    .    .    .    .  <==================>  . SD1930    53 1835 2021  187
.    .    .    .    .    .    .    .    .    .    .    .    .    .    .   <======================>  . SD1931    54 1798 2021  224
.    .    .    .    .    .    .    .    .    .    .    .    .    .    . <========================>  . SD1970    55 1774 2021  248
.    .    .    .    .    .    .    .    .    .    .    .    .    .    .  <=======================>  . SD1971    56 1788 2021  234
.    .    .    .    .    .    .    .    .    .    .    .    .    .    .    <=====================>  . SD1990    57 1800 2021  222
.    .    .    .    .    .    .    .    .    .    .    .    .    .    .    <=====================>  . SD1991    58 1802 2021  220
.    .    .    .    .    .    .    .    .    .    .    .    .    .    .   <======================>  . SD2210    59 1796 2021  226
.    .    .    .    .    .    .    .    .    .    .    .    .    .    .    <====================>   . SD2211    60 1800 2018  219
.    .    .    .    .    .    .    .    .    .    .    .    .    .    . <========================>  . SD2230    61 1776 2021  246
.    .    .    .    .    .    .    .    .    .    .    .    .    .    .   <======================>  . SD2231    62 1798 2021  224
.    .    .    .    .    .    .    .    .    .    .    .    .    .    .   <======================>  . SD2240    63 1795 2021  227
.    .    .    .    .    .    .    .    .    .    .    .    .    .    . <========================>  . SD2241    64 1779 2021  243
.    .    .    .    .    .    .    .    .    .    .    .    .    .    . <========================>  . SD2311    65 1777 2021  245
.    .    .    .    .    .    .    .    .    .    .    .    .    .    .   <======================>  . SD2312    66 1794 2021  228
.    .    .    .    .    .    .    .    .    .    .    .    .    .    .    . <===================>  . SD2501    67 1825 2021  197
.    .    .    .    .    .    .    .    .    .    .    .    .    .    .   <======================>  . SD2502    68 1797 2021  225
.    .    .    .    .    .    .    .    .    .    .    .    .    .    .    <=====================>  . SD2560    69 1802 2021  220
.    .    .    .    .    .    .    .    .    .    .    .    .    .    .    .<====================>  . SD2561    70 1813 2021  209
.    .    .    .    .    .    .    .    .    .    .    .    .    .    .    <=====================>  . SD2580    71 1804 2021  218
.    .    .    .    .    .    .    .    .    .    .    .    .    .    .   <======================>  . SD2581    72 1792 2021  230
.    .    .    .    .    .    .    .    .    .    .    .    .    .    .   <======================>  . SD2760    73 1793 2021  229
.    .    .    .    .    .    .    .    .    .    .    .    .    .    .  <=======================>  . SD2761    74 1788 2021  234
.    .    .    .    .    .    .    .    .    .    .    .    .    .    .    .<====================>  . SD2780    75 1816 2021  206
.    .    .    .    .    .    .    .    .    .    .    .    .    .    .    .  <=================>   . SD2781    76 1833 2014  182
.    .    .    .    .    .    .    .    .    .    .    .    .    .    .    <=====================>  . SD2791    77 1804 2021  218
.    .    .    .    .    .    .    .    .    .    .    .    .    .    .    .  <==================>  . SD2792    78 1833 2021  189
.    .    .    .    .    .    .    .    .    .    .    .    .    .    .    . <===================>  . SD2801    79 1824 2021  198
.    .    .    .    .    .    .    .    .    .    .    .    .    .    .    . <===================>  . SD2802    80 1823 2021  199
.    .    .    .    .    .    .    .    .    .    .    .    .    .    .    . <===================>  . SD2840    81 1827 2021  195
.    .    .    .    .    .    .    .    .    .    .    .    .    .    .    . <===================>  . SD2841    82 1822 2021  200
.    .    .    .    .    .    .    .    .    .    .    .    .    .    .   <======================>  . SD2851    83 1795 2021  227
.    .    .    .    .    .    .    .    .    .    .    .    .    .    .    .<====================>  . SD2852    84 1815 2021  207
.    .    .    .    .    .    .    .    .    .    .    .    .    .    .    .  <==================>  . SD2930    85 1833 2021  189
.    .    .    .    .    .    .    .    .    .    .    .    .    .    .    <=====================>  . SD2931    86 1805 2021  217
.    .    .    .    .    .    .    .    .    .    .    .    .    .    .    .<====================>  . SD3771    87 1818 2021  204
.    .    .    .    .    .    .    .    .    .    .    .    .    .    .  <=======================>  . SD3772    88 1785 2021  237
.    .    .    .    .    .    .    .    .    .    .    .    .    .    .    .   <=================>  . SD4361    89 1846 2021  176
.    .    .    .    .    .    .    .    .    .    .    .    .    .    .    .    . <==============>  . SD4362    90 1878 2021  144
.    .    .    .    .    .    .    .    .    .    .    .    .    .    .    .<====================>  . SD4521    91 1813 2021  209
.    .    .    .    .    .    .    .    .    .    .    .    .    .    .   <======================>  . SD4522    92 1797 2021  225
.    .    .    .    .    .    .    .    .    .    .    .    .    .    .    . <===================>  . SD4531    93 1829 2021  193
.    .    .    .    .    .    .    .    .    .    .    .    .    .    .    .    . <==============>  . SD4532    94 1871 2021  151
.    .    .    .    .    .    .    .    .    .    .    .    .    .    .    .<===================>   . SD4551    95 1819 2017  199
.    .    .    .    .    .    .    .    .    .    .    .    .    .    .   <=====================>   . SD4581    96 1797 2017  221
.    .    .    .    .    .    .    .    .    .    .    .    .    .    .    <====================>   . SD5341    97 1803 2017  215
.    .    .    .    .    .    .    .    .    .    .    .    .    .    .    .    <===============>   . SD6311    98 1856 2016  161
.    .    .    .    .    .    .    .    .    .    .    .    .    .    .    .    <================>  . SD6681    99 1851 2021  171
.    .    .    .    .    .    .    .    .    .    .    .    .    .    .    .   <=================>  . SD6682   100 1848 2021  174
:    :    :    :    :    :    :    :    :    :    :    :    :    :    :    :    :    :    :    :    :
1050 1100 1150 1200 1250 1300 1350 1400 1450 1500 1550 1600 1650 1700 1750 1800 1850 1900 1950 2000 2050
PART 2:  TIME PLOT OF TREE-RING SERIES:                                                 00:00   00     0000  Page   4
-----------------------------------------------------------------------------------------------------------------------------------
1050 1100 1150 1200 1250 1300 1350 1400 1450 1500 1550 1600 1650 1700 1750 1800 1850 1900 1950 2000 2050 Ident   Seq Time-span  Yrs
:    :    :    :    :    :    :    :    :    :    :    :    :    :    :    :    :    :    :    :    :    : -------- --- ---- ---- ----
.    .    .    .    .    .    .    .    .    .    .    .    .    .    .   <======================>  . SD7221   101 1795 2021  227
.    .    .    .    .    .    .    .    .    .    .    .    .    .    .  <=======================>  . SD7222   102 1780 2021  242
.    .    .    .    .    .    .    .    .    .    .    .    .    .    .    .    . <==============>  . SD81     103 1873 2021  149
.    .    .    .    .    .    .    .    .    .    .    .    .    .    .    .   <=================>  . SD82     104 1841 2021  181
.    .    .    .    .    .    .    .    .    .    .    .    .    .    .    .   <================>   . SD83     105 1843 2017  175
.    .    .    .    .    .    .    .    .    .    .    .    .    .    . <=======================>   . SD8601   106 1777 2017  241
.    .    .    .    .    .    .    .    .    .    .    .    .    .    .    .<====================>  . SD9531   107 1811 2021  211
.    .    .    .    .    .    .    .    .    .    .    .    .    .    .    .   <=================>  . SD9532   108 1849 2021  173
:    :    :    :    :    :    :    :    :    :    :    :    :    :    :    :    :    :    :    :    :
1050 1100 1150 1200 1250 1300 1350 1400 1450 1500 1550 1600 1650 1700 1750 1800 1850 1900 1950 2000 2050
PART 3:  Master Dating Series:                                                          00:00   00     0000  Page   5
-----------------------------------------------------------------------------------------------------------------------------------
Year  Value  No Ab    Year  Value  No Ab    Year  Value  No Ab    Year  Value  No Ab    Year  Value  No Ab    Year  Value  No Ab
------------------    ------------------    ------------------    ------------------    ------------------    ------------------

1800 -0.037  40       1850  0.368  94       1900 -1.294 102       1950  0.904 106       2000 -0.940 104
1801  0.569  40       1851  1.330  95       1901 -0.045 102       1951  1.048 106       2001  0.643 104
1802 -3.902  42       1852  0.138  95       1902  0.230 102       1952  0.790 106       2002 -0.710 103
1803 -0.342  44       1853  0.735  95       1903  0.128 102       1953  1.253 106       2003  0.722 103
1804  1.249  50       1854  0.064  95       1904  0.501 102       1954  0.647 106       2004 -1.363 102
1805  0.290  52       1855  0.668  95       1905  1.017 102       1955  1.297 106       2005 -0.445 102
1806  0.107  53       1856  0.341  96       1906  0.783 102       1956 -0.348 106       2006 -0.719 101
1807  0.997  53       1857 -1.259  96       1907  1.073 102       1957 -0.435 106       2007  0.609 101
1808  1.013  54       1858 -2.440  96       1908  0.145 102       1958 -0.012 106       2008  1.655 100
1809  0.641  56       1859  0.384  96       1909 -2.378 102       1959 -1.116 106       2009  0.569 100
```

```
1810  0.448  57       1860  0.789  96       1910 -1.688 102       1960 -1.973 106       2010  0.692 100
1811  1.009  58       1861  1.186  96       1911  0.467 102       1961 -0.508 106       2011 -1.404 100
1812 -0.179  59       1862  0.249  96       1912  0.060 102       1962  0.870 106       2012  0.258 100
1813  0.624  62       1863  1.157  96       1913  0.233 102       1963  0.027 106       2013 -0.125  99
1814 -0.266  63       1864 -0.027  96       1914  0.797 102       1964  0.168 106       2014  0.303  98
1815 -3.435  65       1865 -2.239  96       1915 -0.037 102       1965  1.054 106       2015  1.140  97
1816 -1.062  66       1866 -0.950  96       1916  0.800 104       1966  0.552 106       2016 -0.919  97
1817  0.786  66       1867  0.524  96       1917  0.056 104       1967  0.796 106       2017 -0.378  95
1818  0.104  69       1868 -0.917  96       1918 -1.178 104       1968  0.100 106       2018 -1.064  89
1819 -0.026  70       1869 -0.188  96       1919 -1.097 104       1969  0.129 106       2019  0.914  78
1820 -0.993  70       1870 -0.040  97       1920  0.265 104       1970 -0.452 106       2020 -0.170  70
1821  0.277  70       1871  0.811  98       1921 -0.613 104       1971  0.421 106       2021  0.904  66
1822 -0.275  73       1872  0.829  98       1922 -0.714 104       1972  0.222 106
1823 -1.256  74       1873  0.585  99       1923 -0.732 104       1973  1.025 106
1774  0.166   1       1824 -0.030  75       1874  0.216  99       1924 -0.467 104       1974  0.045 106
1775 -5.472   1       1825  0.407  77       1875  1.206  99       1925  0.388 104       1975  0.784 107
1776  0.726   2       1826  0.431  78       1876  0.556  99       1926  0.324 104       1976 -3.524 107
1777  1.179   4       1827  0.472  79       1877 -2.688  99       1927  0.759 104       1977 -1.259 107
1778  0.256   4       1828  1.089  79       1878  0.206 100       1928  0.080 104       1978  0.595 107
1779 -0.937   5       1829  0.897  80       1879  0.666 100       1929  0.593 104       1979 -0.203 107
1780 -0.869   6       1830  0.589  80       1880 -0.273 100       1930  0.106 104       1980 -0.944 107
1781 -0.275   6       1831 -0.809  80       1881 -0.514 101       1931  1.398 104       1981  0.042 107
1782  0.144   6       1832 -0.457  80       1882  0.516 101       1932  0.363 104       1982  0.896 107
1783  0.711   7       1833  0.295  83       1883  0.586 101       1933  0.726 104       1983  0.416 107
1784  0.344   7       1834  1.244  84       1884  0.571 101       1934 -0.373 105       1984  1.128 107
1785  0.546   8       1835 -0.069  85       1885 -0.275 101       1935  0.035 105       1985  0.923 107
1786  0.226  10       1836  0.255  85       1886  0.725 101       1936  0.469 105       1986 -1.800 107
1787  0.405  10       1837  1.131  85       1887  0.663 101       1937  0.001 106       1987 -0.512 107
1788 -0.273  13       1838 -0.975  85       1888 -1.164 101       1938 -0.125 106       1988  0.776 107
1789 -0.468  13       1839  0.272  85       1889 -0.738 101       1939  0.546 106       1989  0.224 107
1790  0.611  13       1840  0.078  85       1890 -0.574 101       1940  0.040 106       1990 -1.466 107
1791 -0.172  13       1841 -2.420  87       1891 -0.641 101       1941  0.358 106       1991 -0.001 107
1792  0.352  17       1842  0.035  87       1892 -0.220 101       1942 -0.449 106       1992  0.205 107
1793 -0.146  18       1843 -1.074  88       1893 -1.589 101       1943  1.259 106       1993  1.508 106
1794 -0.442  20       1844 -0.415  88       1894  0.384 101       1944 -2.249 106       1994  1.939 106
1795 -0.688  23       1845 -0.203  89       1895  1.518 101       1945 -0.414 106       1995 -0.499 106
1796  1.133  25       1846  0.168  91       1896 -0.727 101       1946 -0.311 106       1996 -1.407 105
1797  0.953  29       1847 -1.076  92       1897  0.496 101       1947  0.077 106       1997 -0.089 105
1798  0.775  34       1848  0.821  93       1898  0.771 101       1948 -3.045 106       1998  0.005 104
1799 -1.223  36       1849  0.839  94       1899  0.829 102       1949 -0.423 106       1999  1.148 104
------------------------------------------------------------------------------------------------------------------------------------
PART 4:  Master Bar Plot:                                                                          00:00   00     0000  Page   6
------------------------------------------------------------------------------------------------------------------------------------
Year Rel value  Year Rel value  Year Rel value  Year Rel value  Year Rel value  Year Rel value  Year Rel value  Year Rel value
1800----@       1850------A     1900-e          1950---------D  2000-d
1801-------B    1851----------E 1901----@       1951---------D  2001--------C
1802p           1852-----A      1902-----A      1952--------C   2002-c
1803--a         1853--------C   1903-----A      1953----------E 2003--------C
1804----------E 1854----@       1904-------B    1954--------C   2004-e
1805------A     1855--------C   1905---------D  1955----------E 2005--b
1806-----@      1856------A     1906--------C   1956--a         2006-c
1807---------D  1857-e          1907---------D  1957--b         2007-------B
1808---------D  1858j           1908-----A      1958----@       2008----------G
1809--------C   1859------B     1909j           1959-d          2009-------B
1810------B     1860--------C   1910g           1960h           2010--------C
1811---------D  1861----------E 1911-------B    1961--b         2011-f
1812---a        1862-----A      1912----@       1962---------C  2012-----A
1813-------B    1863----------E 1913-----A      1963----@       2013---@
1814---a        1864----@       1914--------C   1964-----A      2014------A
1815n           1865i           1915----@       1965---------D  2015----------E
1816-d          1866-d          1916--------C   1966-------B    2016-d
1817--------C   1867-------B    1917----@       1967--------C   2017--b
1818-----@      1868-d          1918-e          1968-----@      2018-d
1819----@       1869---a        1919-d          1969-----A      2019---------D
1820-d          1870----@       1920------A     1970--b         2020---a
1821------A     1871--------C   1921--b         1971------B     2021---------D
1822---a        1872---------C  1922-c          1972-----A
1823-e          1873-------B    1923-c          1973---------D
1774-----A      1824----@       1874-----A      1924--b         1974----@
1775v           1825------B     1875----------E 1925------B     1975--------C
1776--------C   1826------B     1876-------B    1926------A     1976n
1777----------E 1827-------B    1877k           1927--------C   1977-e
1778-----A      1828----------D 1878-----A      1928----@       1978-------B
1779-d          1829---------D  1879--------C   1929-------B    1979---a
1780-c          1830-------B    1880---a        1930-----@      1980-d
1781---a        1831-c          1881--b         1931----------F 1981----@
1782-----A      1832--b         1882-------B    1932------A     1982---------D
1783--------C   1833------A     1883-------B    1933--------C   1983------B
1784------A     1834----------E 1884-------B    1934--a         1984----------E
1785-------B    1835----@       1885---a        1935----@       1985---------D
1786-----A      1836-----A      1886--------C   1936-------B    1986g
1787------B     1837----------E 1887--------C   1937----@       1987--b
1788---a        1838-d          1888-e          1938---a        1988--------C
1789--b         1839------A     1889-c          1939-------B    1989-----A
1790-------B    1840----@       1890--b         1940----@       1990f
1791---a        1841j           1891--c         1941------A     1991----@
1792------A     1842----@       1892---a        1942--b         1992-----A
1793---a        1843-d          1893f           1943----------E 1993----------F
1794--b         1844--b         1894------B     1944i           1994----------H
1795--c         1845---a        1895----------F 1945--b         1995--b
1796----------E 1846-----A      1896-c          1946---a        1996-f
1797---------D  1847-d          1897-------B    1947----@       1997---@
```

1798--------C   1848---------C 1898--------C   1948l   1998----@
1799-e   1849---------C 1899---------C 1949--b   1999----------E

PART 5: CORRELATION OF SERIES BY SEGMENTS: 00:00   00   0000 Page   5

Correlations of 50-year dated segments, lagged 25 years
Flags: A = correlation under 0.3281 but highest as dated; B = correlation higher at other than dated position

Seq	Series	Time span		1775 1824	1800 1849	1825 1874	1850 1899	1875 1924	1900 1949	1925 1974	1950 1999	1975 2024
1	L10011	1804	2005		.59	.69	.74	.70	.51	.50	.55	.47
2	L10012	1797	1807	.74								
3	L10012	1810	2013		.63	.65	.78	.74	.58	.58	.80	.74
4	L10021	1818	2018		.50	.52	.63	.56	.69	.73	.78	.78
5	L10022	1826	2019			.59	.50	.41	.68	.72	.34	.40
6	L10061	1799	2019	.40	.37	.43	.32B	.47	.69	.64	.58	.62
7	L10062	1812	2019		.32A	.35	.74	.81	.64	.57	.66	.74
8	L10071	1822	2018		.58	.56	.60	.56	.76	.74	.71	.66
9	L10072	1803	2018		.26B	.34	.72	.73	.71	.55	.28B	.27A
10	L10081	1846	2020			.73	.71	.71	.65	.52	.53	.61
11	L10082	1808	2020		.59	.64	.77	.83	.68	.60	.43	.25A
12	L10101	1825	2020			.62	.48	.40	.49	.49	.72	.52
13	L10102	1814	2020		.64	.69	.70	.59	.62	.65	.66	.40
14	L10131	1804	2018		.59	.52	.65	.66	.70	.57	.70	.71
15	L10132	1881	2018					.74	.77	.75	.84	.85
16	L10141	1809	2018		.58	.36	.58	.67	.69	.64	.67	.80
17	L10142	1822	2017		.41	.41	.59	.70	.48	.45	.84	.78
18	L10151	1796	2019	.71	.72	.61	.79	.79	.58	.54	.89	.84
19	L10152	1788	2019	.64	.69	.67	.82	.73	.70	.68	.78	.82
20	L10171	1847	2018			.83	.82	.76	.60	.45B	.57	.79
21	L10172	1834	2018			.60	.54	.48	.56	.56	.80	.90
22	L10181	1916	2019						.72	.71	.70	.79
23	L10182	1800	2019		.60	.60	.75	.78	.71	.80	.80	.85
24	L10183	1818	2019		.59	.64	.78	.82	.71	.69	.68	.79
25	L10331	1808	2018		.63	.76	.81	.79	.78	.73	.73	.79
26	L10332	1975	2018									.86
27	L7221	1800	2001		.54	.58	.72	.76	.70	.70	.84	.85
28	L7222	1794	1997	.76	.76	.68	.76	.76	.61	.62	.80	
29	L7231	1792	1995	.71	.73	.62	.52	.33A	.16B	.25A	.41	
30	L7271	1916	2003						.65	.62	.37B	.17B
31	L7272	1815	1992		.05B	.09B	.18B	.49	.71	.79	.60	
32	L7273	1798	2007	.23B	.33B	.10B	.10B	.49	.76	.78	.58	.40
33	L7301	1809	2016		.68	.75	.67	.43	.44	.63	.71	.77
34	L7302	1799	2012	.72	.71	.80	.76	.54	.45	.59	.85	.88
35	SD1330	1792	2021	.45	.44	.59	.48	.53	.64	.60	.61	.72
36	SD1331	1804	2021		.43	.63	.52	.50	.54	.55	.68	.79
37	SD1401	1870	2021				.35	.36	.57	.60	.50	.45
38	SD1402	1845	2021			.59	.61	.56	.45	.52	.72	.80
39	SD1621	1786	2021	.44	.58	.55	.55	.67	.65	.59	.70	.67
40	SD1622	1786	2021	.40	.55	.44	.31A	.48	.51	.55	.66	.66
41	SD1650	1804	2021		.66	.69	.66	.50	.52	.63	.72	.80
42	SD1651	1798	2021	.76	.74	.76	.67	.64	.73	.78	.74	.84
43	SD1720	1937	2021							.76	.72	.71
44	SD1721	1934	2021							.85	.83	.81
45	SD1750	1792	2021	.58	.62	.70	.66	.62	.65	.76	.79	.80
46	SD1751	1813	2021		.54	.66	.75	.72	.67	.69	.72	.69
47	SD1752	1899	2021					.62	.61	.70	.74	.71
48	SD1753	1806	2021		.58	.58	.42	.47	.59	.63	.84	.80
49	SD1754	1805	2021		.65	.71	.71	.59	.55	.59	.76	.83
50	SD1755	1783	2021	.56	.67	.76	.74	.70	.56	.37	.53	.69
51	SD1920	1841	2021			.64	.69	.79	.69	.70	.89	.87
52	SD1921	1798	2021	.69	.69	.61	.76	.69	.51	.47B	.71	.73
53	SD1930	1835	2021			.71	.73	.62	.62	.47	.45	.53
54	SD1931	1798	2021	.63	.66	.60	.78	.71	.53	.54	.78	.79
55	SD1970	1774	2021	.58	.72	.56	.61	.68	.74	.72	.80	.80
56	SD1971	1788	2021	.60	.66	.55	.23B	.14B	.53	.64	.85	.84
57	SD1990	1800	2021		.46	.36	.41	.42	.63	.63	.64	.62
58	SD1991	1802	2021		.50	.44	.41	.47	.75	.76	.73	.72
59	SD2210	1796	2021	.53	.55	.58	.69	.79	.65	.64	.78	.76
60	SD2211	1800	2018		.54	.69	.75	.76	.59	.56	.74	.53
61	SD2230	1776	2021	.55	.71	.45	.60	.57	.56	.72	.69	.61
62	SD2231	1798	2021	.64	.68	.45	.63	.53	.54	.66	.78	.82
63	SD2240	1795	2021	.80	.77	.58	.60	.43	.62	.72	.57	.48
64	SD2241	1779	2021	.68	.79	.62	.71	.52	.37	.48	.51	.56
65	SD2311	1777	2021	.63	.68	.74	.83	.83	.75	.67	.81	.80
66	SD2312	1794	2021	.73	.72	.75	.68	.67	.79	.75	.84	.86
67	SD2501	1825	2021			.62	.68	.56	.49	.65	.53	.43
68	SD2502	1797	2021	.26A	.33	.72	.68	.55	.59	.60	.60	.54
69	SD2560	1802	2021		.68	.66	.68	.54	.60	.66	.73	.76
70	SD2561	1813	2021		.68	.68	.60	.70	.71	.53	.72	.86
71	SD2580	1804	2021		.55	.57	.73	.66	.46	.32A	.70	.78
72	SD2581	1792	2021	.27B	.30B	.56	.53	.45	.70	.71	.80	.80
73	SD2760	1793	2021	.42	.49	.74	.57	.25A	.30A	.28B	.50	.71
74	SD2761	1788	2021	.22B	.40	.48	.14B	.23B	.39	.31B	.53	.75
75	SD2780	1816	2021		.77	.74	.62	.55	.73	.78	.58	.44
76	SD2781	1833	2014			.62	.63	.58	.73	.78	.79	.75
77	SD2791	1804	2021		.66	.60	.40	.38	.61	.56	.71	.68
78	SD2792	1833	2021			.66	.52	.58	.67	.63	.72	.69
79	SD2801	1824	2021		.58	.54	.46	.40	.67	.69	.69	.83
80	SD2802	1823	2021		.52	.55	.43	.54	.38	.06B	.34	.67
81	SD2840	1827	2021			.73	.68	.50	.37	.52	.80	.82
82	SD2841	1822	2021		.73	.77	.79	.69	.68	.70	.79	.82
83	SD2851	1795	2021	.68	.67	.57	.48	.56	.68	.68	.70	.70

84	SD2852	1815	2021		.54	.59	.58	.64	.71	.66	.60	.49
85	SD2930	1833	2021			.54	.69	.69	.54	.54	.82	.82
86	SD2931	1805	2021		.59	.61	.59	.57	.57	.58	.69	.79
87	SD3771	1818	2021		-.20B	-.17B	-.05B	-.06B	.29B	.63	.46	.49
88	SD3772	1785	2021	.37B	.16B	-.03B	.05B	.05B	.30A	.30B	.21B	.32A
89	SD4361	1846	2021			.64	.67	.57	.50	.58	.75	.79
90	SD4362	1878	2021					.33	.43	.55	.66	.66
91	SD4521	1813	2021		.62	.62	.71	.82	.74	.64	.62	.46
92	SD4522	1797	2021	.60	.60	.64	.70	.72	.66	.65	.57	.67
93	SD4531	1829	2021			.00B	.13B	.36	.58	.63	.51	.63
94	SD4532	1871	2021				.41	.44	.38B	.42	.71	.87
95	SD4551	1819	2017		.73	.73	.68	.74	.76	.75	.84	.86
96	SD4581	1797	2017	.31B	.33B	.57	.62	.40	-.05B	.06B	.40	.60
97	SD5341	1803	2017		.28A	.17B	.33	.45	.66	.55	.75	.86
98	SD6311	1856	2016				.68	.63	.75	.79	.64	.73
99	SD6681	1851	2021				.86	.85	.77	.69	.83	.87
100	SD6682	1848	2021			.71	.71	.71	.73	.71	.80	.81
101	SD7221	1795	2021	.68	.65	.59	.53	.23A	.26A	.20B	.32A	.44
102	SD7222	1780	2021	.76	.70	.54	.65	.58	.61	.62	.60	.67
103	SD81	1873	2021				.60	.60	.81	.76	.68	.62
104	SD82	1841	2021			.32B	.38	.53	.72	.72	.75	.76
105	SD83	1843	2017			.38	.52	.57	.61	.56	.76	.75
106	SD8601	1777	2017	.68	.65	.31B	.10B	.03B	.07B	.09B	.20B	.29A
107	SD9531	1811	2021		.30A	.46	.48	.26B	.42	.65	.75	.65
108	SD9532	1849	2021			.68	.66	.47	.39	.56	.69	.68
Av segment correlation				0.57	0.56	0.57	0.59	0.57	0.59	0.60	0.67	0.69

Data S3: COFECHA output of tree ring width from the younger trees. The dating statistics were calculated by COFECHA for all trees younger than 120 years. This is sections 1 to 5 of the raw output of the program using the default settings.

```
[]  Dendrochronology Program Library                                        Run YOUNG  Program COF  00:00   00     0000  Page   1
[]
[]  P R O G R A M      C O F E C H A                                                                     Version 6.06P      -30
------------------------------------------------------------------------------------------------------------------------------------
QUALITY CONTROL AND DATING CHECK OF TREE-RING MEASUREMENTS
File of DATED series:   TRWYOUNG.rwl
CONTENTS:
Part 1:  Title page, options selected, summary, absent rings by series
Part 2:  Histogram of time spans
Part 3:  Master series with sample depth and absent rings by year
Part 4:  Bar plot of Master Dating Series
Part 5:  Correlation by segment of each series with Master
Part 6:  Potential problems: low correlation, divergent year-to-year changes, absent rings, outliers
Part 7:  Descriptive statistics
RUN CONTROL OPTIONS SELECTED                             VALUE
1  Cubic smoothing spline 50                                                         32 years
2  Segments examined are                       50 years lagged successively by  25 years
3  Autoregressive model applied                     A  Residuals are used in master dating series and testing
4  Series transformed to logarithms                 Y  Each series log-transformed for master dating series and testing
5  CORRELATION is Pearson (parametric, quantitative)
Critical correlation, 99        6  Master dating series saved                       N
7  Ring measurements listed                         N
8  Parts printed                              1234567
9  Absent rings are omitted from master series and segment correlations  (Y)
Time span of Master dating series is  1914 to  2022   109 years
Continuous time span is               1914 to  2022   109 years
Portion with two or more series is    1916 to  2022   107 years
****************************************
*C* Number of dated series       129 *C*
*O* Master series 1914 2022  109 yrs *O*
*F* Total rings in all series   8337 *F*
*E* Total dated rings checked   8335 *E*
*C* Series intercorrelation    0.424 *C*
*H* Average mean sensitivity   0.253 *H*
*A* Segments, possible problems   64 *A*
*** Mean length of series       64.6 ***
****************************************
ABSENT RINGS listed by SERIES:            (See Master Dating Series for absent rings listed by year)
No ring measurements of zero value
PART 2:  TIME PLOT OF TREE-RING SERIES:                                                    00:00   00     0000  Page   2
------------------------------------------------------------------------------------------------------------------------------------
1050 1100 1150 1200 1250 1300 1350 1400 1450 1500 1550 1600 1650 1700 1750 1800 1850 1900 1950 2000 2050 Ident    Seq Time-span  Yrs
:    :    :    :    :    :    :    :    :    :    :    :    :    :    :    :    :    :    :    :    : -------- --- ---- ---- ----
.    .    .    .    .    .    .    .    .    .    .    .    .    .    .    .    .    .   <=======>  . NG1070     1 1941 2021   81
.    .    .    .    .    .    .    .    .    .    .    .    .    .    .    .    .    .   <=======>  . NG1071     2 1941 2021   81
.    .    .    .    .    .    .    .    .    .    .    .    .    .    .    .    .    .    <======>  . NG1110     3 1956 2021   66
.    .    .    .    .    .    .    .    .    .    .    .    .    .    .    .    .    .    .<=====>  . NG1111     4 1965 2021   57
.    .    .    .    .    .    .    .    .    .    .    .    .    .    .    .    .    .    <======>  . NG1120     5 1952 2021   70
.    .    .    .    .    .    .    .    .    .    .    .    .    .    .    .    .    .   <=======>  . NG1121     6 1943 2021   79
.    .    .    .    .    .    .    .    .    .    .    .    .    .    .    .    .    .    .<=====>  . NG1180     7 1961 2021   61
.    .    .    .    .    .    .    .    .    .    .    .    .    .    .    .    .    .    .<=====>  . NG1181     8 1963 2021   59
.    .    .    .    .    .    .    .    .    .    .    .    .    .    .    .    .    .    .<=====>  . NG1182     9 1963 2021   59
.    .    .    .    .    .    .    .    .    .    .    .    .    .    .    .    .    .    .<=====>  . NG1183    10 1968 2021   54
.    .    .    .    .    .    .    .    .    .    .    .    .    .    .    .    .    .    .<=====>  . NG1190    11 1964 2021   58
.    .    .    .    .    .    .    .    .    .    .    .    .    .    .    .    .    .    <======>  . NG1191    12 1957 2021   65
.    .    .    .    .    .    .    .    .    .    .    .    .    .    .    .    .    .    . <====>  . NG1280    13 1971 2021   51
.    .    .    .    .    .    .    .    .    .    .    .    .    .    .    .    .    .    . <====>  . NG1281    14 1972 2021   50
.    .    .    .    .    .    .    .    .    .    .    .    .    .    .    .    .    .    .<=====>  . NG1282    15 1962 2022   61
.    .    .    .    .    .    .    .    .    .    .    .    .    .    .    .    .    .    . <====>  . NG1283    16 1976 2022   47
.    .    .    .    .    .    .    .    .    .    .    .    .    .    .    .    .    .    .<=====>  . NG1290    17 1961 2022   62
.    .    .    .    .    .    .    .    .    .    .    .    .    .    .    .    .    .    .<=====>  . NG1291    18 1965 2022   58
.    .    .    .    .    .    .    .    .    .    .    .    .    .    .    .    .    .    .<=====>  . NG1320    19 1964 2021   58
.    .    .    .    .    .    .    .    .    .    .    .    .    .    .    .    .    .    .<=====>  . NG1321    20 1966 2021   56
.    .    .    .    .    .    .    .    .    .    .    .    .    .    .    .    .    .    .<=====>  . NG1400    21 1968 2022   55
.    .    .    .    .    .    .    .    .    .    .    .    .    .    .    .    .    .    .<=====>  . NG1401    22 1967 2022   56
.    .    .    .    .    .    .    .    .    .    .    .    .    .    .    .    .    .    .<=====>  . NG1420    23 1968 2022   55
.    .    .    .    .    .    .    .    .    .    .    .    .    .    .    .    .    .    .<=====>  . NG1421    24 1969 2022   54
.    .    .    .    .    .    .    .    .    .    .    .    .    .    .    .    .    .    .<=====>  . NG1422    25 1969 2022   54
.    .    .    .    .    .    .    .    .    .    .    .    .    .    .    .    .    .    . <====>  . NG1423    26 1971 2022   52
.    .    .    .    .    .    .    .    .    .    .    .    .    .    .    .    .    .    .<=====>  . NG1430    27 1968 2022   55
.    .    .    .    .    .    .    .    .    .    .    .    .    .    .    .    .    .    <======>  . NG1431    28 1958 2022   65
.    .    .    .    .    .    .    .    .    .    .    .    .    .    .    .    .    .    <======>  . NG1440    29 1959 2022   64
.    .    .    .    .    .    .    .    .    .    .    .    .    .    .    .    .    .    .<=====>  . NG1441    30 1968 2022   55
.    .    .    .    .    .    .    .    .    .    .    .    .    .    .    .    .    .    . <====>  . NG1470    31 1975 2022   48
.    .    .    .    .    .    .    .    .    .    .    .    .    .    .    .    .    .    .<=====>  . NG1471    32 1966 2022   57
.    .    .    .    .    .    .    .    .    .    .    .    .    .    .    .    .    .    .<====>   . NG151     33 1968 2017   50
.    .    .    .    .    .    .    .    .    .    .    .    .    .    .    .    .    .    .<=====>  . NG1580    34 1962 2021   60
.    .    .    .    .    .    .    .    .    .    .    .    .    .    .    .    .    .    .<=====>  . NG1581    35 1967 2021   55
.    .    .    .    .    .    .    .    .    .    .    .    .    .    .    .    .    .    <======>  . NG2640    36 1958 2021   64
.    .    .    .    .    .    .    .    .    .    .    .    .    .    .    .    .    .    <======>  . NG2641    37 1959 2021   63
.    .    .    .    .    .    .    .    .    .    .    .    .    .    .    .    .    .    <======>  . NG2650    38 1952 2021   70
.    .    .    .    .    .    .    .    .    .    .    .    .    .    .    .    .    .    .<=====>  . NG2651    39 1962 2021   60
.    .    .    .    .    .    .    .    .    .    .    .    .    .    .    .    .    .    .<=====>  . NG2710    40 1968 2021   54
.    .    .    .    .    .    .    .    .    .    .    .    .    .    .    .    .    .    .<=====>  . NG2711    41 1967 2021   55
.    .    .    .    .    .    .    .    .    .    .    .    .    .    .    .    .    .    . <====>  . NG2712    42 1970 2021   52
.    .    .    .    .    .    .    .    .    .    .    .    .    .    .    .    .    .    .<=====>  . NG2713    43 1965 2021   57
.    .    .    .    .    .    .    .    .    .    .    .    .    .    .    .    .    .    .<=====>  . NG2800    44 1967 2021   55
```

```
.    .    .    .    .    .    .    .    .    .    .    .    .    .    .    .    .    .    <======>  . NG2801    45 1958 2021   64
.    .    .    .    .    .    .    .    .    .    .    .    .    .    .    .    .    .   <=======>  . NG2900    46 1948 2021   74
.    .    .    .    .    .    .    .    .    .    .    .    .    .    .    .    .    .    .<=====>  . NG2901    47 1960 2021   62
.    .    .    .    .    .    .    .    .    .    .    .    .    .    .    .    .    .    <======>  . NG2940    48 1957 2021   65
.    .    .    .    .    .    .    .    .    .    .    .    .    .    .    .    .    .    <======>  . NG2941    49 1957 2020   64
.    .    .    .    .    .    .    .    .    .    .    .    .    .    .    .    .    .    <======>  . NG3020    50 1955 2021   67
:    :    :    :    :    :    :    :    :    :    :    :    :    :    :    :    :    :    :    :    :
1050 1100 1150 1200 1250 1300 1350 1400 1450 1500 1550 1600 1650 1700 1750 1800 1850 1900 1950 2000 2050
PART 2:  TIME PLOT OF TREE-RING SERIES:                                                   00:00   00     0000  Page   3
-------------------------------------------------------------------------------------------------------------------------
1050 1100 1150 1200 1250 1300 1350 1400 1450 1500 1550 1600 1650 1700 1750 1800 1850 1900 1950 2000 2050 Ident   Seq Time-span  Yrs
:    :    :    :    :    :    :    :    :    :    :    :    :    :    :    :    :    :    :    :    : -------- --- ---- ---- ----
.    .    .    .    .    .    .    .    .    .    .    .    .    .    .    .    .    .    <======>  . NG3021    51 1951 2021   71
.    .    .    .    .    .    .    .    .    .    .    .    .    .    .    .    .    .    .<=====>  . NG3040    52 1960 2021   62
.    .    .    .    .    .    .    .    .    .    .    .    .    .    .    .    .    .    . <====>  . NG3041    53 1972 2021   50
.    .    .    .    .    .    .    .    .    .    .    .    .    .    .    .    .    .    .<=====>  . NG3051    54 1960 2021   62
.    .    .    .    .    .    .    .    .    .    .    .    .    .    .    .    .    .    <======>  . NG3052    55 1953 2021   69
.    .    .    .    .    .    .    .    .    .    .    .    .    .    .    .    .    .    <======>  . NG3101    56 1951 2021   71
.    .    .    .    .    .    .    .    .    .    .    .    .    .    .    .    .    .    <======>  . NG3102    57 1956 2021   66
.    .    .    .    .    .    .    .    .    .    .    .    .    .    .    .    .    .    .  <===>  . NG3571    58 1983 2021   39
.    .    .    .    .    .    .    .    .    .    .    .    .    .    .    .    .    .    .   <==>  . NG3572    59 1991 2021   31
.    .    .    .    .    .    .    .    .    .    .    .    .    .    .    .    .    .    . <====>  . NG3581    60 1970 2021   52
.    .    .    .    .    .    .    .    .    .    .    .    .    .    .    .    .    .    . <====>  . NG3582    61 1975 2021   47
.    .    .    .    .    .    .    .    .    .    .    .    .    .    .    .    .    .   <=======>  . NG4141    62 1946 2020   75
.    .    .    .    .    .    .    .    .    .    .    .    .    .    .    .    .    .    <======>  . NG4142    63 1950 2021   72
.    .    .    .    .    .    .    .    .    .    .    .    .    .    .    .    .    .    .<=====>  . WG11      64 1964 2022   59
.    .    .    .    .    .    .    .    .    .    .    .    .    .    .    .    .    .    .<=====>  . WG12      65 1968 2022   55
.    .    .    .    .    .    .    .    .    .    .    .    .    .    .    .    .    .    .<=====>  . WG101     66 1964 2022   59
.    .    .    .    .    .    .    .    .    .    .    .    .    .    .    .    .    .    .<=====>  . WG102     67 1961 2022   62
.    .    .    .    .    .    .    .    .    .    .    .    .    .    .    .    .    . <=========>  . WG111     68 1928 2022   95
.    .    .    .    .    .    .    .    .    .    .    .    .    .    .    .    .    .  <========>  . WG112     69 1933 2022   90
.    .    .    .    .    .    .    .    .    .    .    .    .    .    .    .    .    .    .<=====>  . WG121     70 1962 2022   61
.    .    .    .    .    .    .    .    .    .    .    .    .    .    .    .    .    .    . <====>  . WG122     71 1973 2022   50
.    .    .    .    .    .    .    .    .    .    .    .    .    .    .    .    .    .    .<=====>  . WG131     72 1966 2022   57
.    .    .    .    .    .    .    .    .    .    .    .    .    .    .    .    .    .    <======>  . WG132     73 1959 2022   64
.    .    .    .    .    .    .    .    .    .    .    .    .    .    .    .    .    .    . <====>  . WG141     74 1971 2022   52
.    .    .    .    .    .    .    .    .    .    .    .    .    .    .    .    .    .    .<=====>  . WG142     75 1963 2022   60
.    .    .    .    .    .    .    .    .    .    .    .    .    .    .    .    .    .    <======>  . WG151     76 1959 2022   64
.    .    .    .    .    .    .    .    .    .    .    .    .    .    .    .    .    .    .<=====>  . WG152     77 1961 2022   62
.    .    .    .    .    .    .    .    .    .    .    .    .    .    .    .    .    .    . <====>  . WG161     78 1971 2022   52
.    .    .    .    .    .    .    .    .    .    .    .    .    .    .    .    .    .    .<=====>  . WG162     79 1963 2022   60
.    .    .    .    .    .    .    .    .    .    .    .    .    .    .    .    .    .    <======>  . WG171     80 1957 2022   66
.    .    .    .    .    .    .    .    .    .    .    .    .    .    .    .    .    .    .<=====>  . WG172     81 1963 2022   60
.    .    .    .    .    .    .    .    .    .    .    .    .    .    .    .    .    . <=========>  . WG181     82 1927 2022   96
.    .    .    .    .    .    .    .    .    .    .    .    .    .    .    .    .    . <=========>  . WG182     83 1921 2022  102
.    .    .    .    .    .    .    .    .    .    .    .    .    .    .    .    .    .    .<=====>  . WG191     84 1964 2022   59
.    .    .    .    .    .    .    .    .    .    .    .    .    .    .    .    .    .    .<=====>  . WG192     85 1967 2022   56
.    .    .    .    .    .    .    .    .    .    .    .    .    .    .    .    .    .    <======>  . WG21      86 1959 2022   64
.    .    .    .    .    .    .    .    .    .    .    .    .    .    .    .    .    .    <======>  . WG22      87 1959 2022   64
.    .    .    .    .    .    .    .    .    .    .    .    .    .    .    .    .    .    . <====>  . WG201     88 1972 2022   51
.    .    .    .    .    .    .    .    .    .    .    .    .    .    .    .    .    .    .<=====>  . WG202     89 1960 2022   63
.    .    .    .    .    .    .    .    .    .    .    .    .    .    .    .    .    .    <======>  . WG211     90 1957 2022   66
.    .    .    .    .    .    .    .    .    .    .    .    .    .    .    .    .    .    <======>  . WG212     91 1957 2022   66
.    .    .    .    .    .    .    .    .    .    .    .    .    .    .    .    .    . <=========>  . WG221     92 1927 2022   96
.    .    .    .    .    .    .    .    .    .    .    .    .    .    .    .    .    .   <=======>  . WG222     93 1946 2022   77
.    .    .    .    .    .    .    .    .    .    .    .    .    .    .    .    .    .    .<=====>  . WG231     94 1961 2022   62
.    .    .    .    .    .    .    .    .    .    .    .    .    .    .    .    .    .    .<=====>  . WG232     95 1962 2022   61
.    .    .    .    .    .    .    .    .    .    .    .    .    .    .    .    .    .<==========>  . WG241     96 1916 2022  107
.    .    .    .    .    .    .    .    .    .    .    .    .    .    .    .    .    .<==========>  . WG242     97 1914 2022  109
.    .    .    .    .    .    .    .    .    .    .    .    .    .    .    .    .    .    <======>  . WG251     98 1954 2022   69
.    .    .    .    .    .    .    .    .    .    .    .    .    .    .    .    .    .  <========>  . WG252     99 1938 2022   85
.    .    .    .    .    .    .    .    .    .    .    .    .    .    .    .    .    .    .<=====>  . WG261    100 1962 2022   61
:    :    :    :    :    :    :    :    :    :    :    :    :    :    :    :    :    :    :    :    :
1050 1100 1150 1200 1250 1300 1350 1400 1450 1500 1550 1600 1650 1700 1750 1800 1850 1900 1950 2000 2050
PART 2:  TIME PLOT OF TREE-RING SERIES:                                                   00:00   00     0000  Page   4
-------------------------------------------------------------------------------------------------------------------------
1050 1100 1150 1200 1250 1300 1350 1400 1450 1500 1550 1600 1650 1700 1750 1800 1850 1900 1950 2000 2050 Ident   Seq Time-span  Yrs
:    :    :    :    :    :    :    :    :    :    :    :    :    :    :    :    :    :    :    :    : -------- --- ---- ---- ----
.    .    .    .    .    .    .    .    .    .    .    .    .    .    .    .    .    .    .<=====>  . WG262    101 1964 2022   59
.    .    .    .    .    .    .    .    .    .    .    .    .    .    .    .    .    .    <======>  . WG271    102 1953 2022   70
.    .    .    .    .    .    .    .    .    .    .    .    .    .    .    .    .    .    <======>  . WG272    103 1954 2022   69
.    .    .    .    .    .    .    .    .    .    .    .    .    .    .    .    .    .    .<=====>  . WG281    104 1960 2022   63
.    .    .    .    .    .    .    .    .    .    .    .    .    .    .    .    .    .    .<=====>  . WG282    105 1964 2022   59
.    .    .    .    .    .    .    .    .    .    .    .    .    .    .    .    .    . <=========>  . WG291    106 1920 2022  103
.    .    .    .    .    .    .    .    .    .    .    .    .    .    .    .    .    .  <========>  . WG292    107 1933 2022   90
.    .    .    .    .    .    .    .    .    .    .    .    .    .    .    .    .    .    .<=====>  . WG31     108 1967 2022   56
.    .    .    .    .    .    .    .    .    .    .    .    .    .    .    .    .    .    .<=====>  . WG32     109 1961 2022   62
.    .    .    .    .    .    .    .    .    .    .    .    .    .    .    .    .    .    .<=====>  . WG301    110 1969 2022   54
.    .    .    .    .    .    .    .    .    .    .    .    .    .    .    .    .    .    .<=====>  . WG302    111 1960 2022   63
.    .    .    .    .    .    .    .    .    .    .    .    .    .    .    .    .    .    .<=====>  . WG311    112 1964 2022   59
.    .    .    .    .    .    .    .    .    .    .    .    .    .    .    .    .    .    .<=====>  . WG312    113 1963 2022   60
.    .    .    .    .    .    .    .    .    .    .    .    .    .    .    .    .    .    .<=====>  . WG321    114 1967 2022   56
.    .    .    .    .    .    .    .    .    .    .    .    .    .    .    .    .    .    <======>  . WG322    115 1959 2022   64
.    .    .    .    .    .    .    .    .    .    .    .    .    .    .    .    .    .   <=======>  . WG331    116 1942 2022   81
.    .    .    .    .    .    .    .    .    .    .    .    .    .    .    .    .    .   <=======>  . WG332    117 1945 2022   78
.    .    .    .    .    .    .    .    .    .    .    .    .    .    .    .    .    .    .<=====>  . WG41     118 1963 2022   60
.    .    .    .    .    .    .    .    .    .    .    .    .    .    .    .    .    .    .<=====>  . WG42     119 1961 2022   62
.    .    .    .    .    .    .    .    .    .    .    .    .    .    .    .    .    .    <======>  . WG51     120 1958 2022   65
.    .    .    .    .    .    .    .    .    .    .    .    .    .    .    .    .    .    .<=====>  . WG52     121 1965 2022   58
.    .    .    .    .    .    .    .    .    .    .    .    .    .    .    .    .    . <=========>  . WG61     122 1925 2022   98
.    .    .    .    .    .    .    .    .    .    .    .    .    .    .    .    .    .  <========>  . WG62     123 1933 2022   90
.    .    .    .    .    .    .    .    .    .    .    .    .    .    .    .    .    .   <=======>  . WG71     124 1940 2022   83
```

```
 .    .    .    .    .    .    .    .    .    .    .    .    .    .    .    .    .    .  <=======>  . WG72     125 1941 2022   82
 .    .    .    .    .    .    .    .    .    .    .    .    .    .    .    .    .    .   .<=====>  . WG81     126 1963 2022   60
 .    .    .    .    .    .    .    .    .    .    .    .    .    .    .    .    .    .   <======>  . WG82     127 1958 2022   65
 .    .    .    .    .    .    .    .    .    .    .    .    .    .    .    .    .    .  <=======>  . WG91     128 1940 2022   83
 .    .    .    .    .    .    .    .    .    .    .    .    .    .    .    .    .    .  <=======>  . WG92     129 1941 2022   82
 :    :    :    :    :    :    :    :    :    :    :    :    :    :    :    :    :    :    :    :    :
1050 1100 1150 1200 1250 1300 1350 1400 1450 1500 1550 1600 1650 1700 1750 1800 1850 1900 1950 2000 2050
PART 3:  Master Dating Series:                                                                      00:00   00     0000  Page   5
-------------------------------------------------------------------------------------------------------------------------------------
Year  Value  No Ab    Year  Value  No Ab    Year  Value  No Ab    Year  Value  No Ab    Year  Value  No Ab    Year  Value  No Ab
------------------    ------------------    ------------------    ------------------    ------------------    ------------------
1950  0.497  25       2000 -0.227 129
1951 -0.150  27       2001 -0.572 129
1952  0.384  29       2002  0.026 129
1953  0.799  31       2003  0.951 129
1954  0.271  33       2004 -0.292 129
1955  0.973  34       2005 -0.112 129
1956 -0.571  36       2006  0.193 129
1957 -0.387  42       2007 -2.457 129
1958  0.667  47       2008 -0.260 129
1959  0.019  54       2009  1.173 129
1960 -0.884  60       2010  0.473 129
1961  0.680  67       2011 -0.339 129
1962  0.146  73       2012  0.178 129
1963 -0.494  81       2013 -0.654 129
1914  1.480   1       1964  0.473  89       2014  0.778 129
1915 -1.209   1       1965 -0.151  93       2015  0.279 129
1916 -1.925   2       1966  0.317  96       2016 -1.567 129
1917 -0.912   2       1967  0.033 103       2017 -1.393 129
1918  1.360   2       1968  0.773 111       2018 -0.748 128
1919  2.272   2       1969  0.338 114       2019  1.193 128
1920  1.397   3       1970  0.084 116       2020  0.883 128
1921  0.686   4       1971  0.406 120       2021  0.462 126
1922 -0.271   4       1972 -0.730 123       2022  0.035  82
1923  1.109   4       1973  0.432 124
1924  1.170   4       1974  0.019 124
1925  0.952   5       1975  0.781 126
1926  0.182   5       1976 -1.141 127
1927 -1.239   7       1977 -0.630 127
1928 -1.454   8       1978 -0.832 127
1929 -1.083   8       1979 -0.718 127
1930  0.304   8       1980 -1.807 127
1931  2.100   8       1981 -0.494 127
1932  1.082   8       1982  1.159 127
1933  0.845  11       1983  0.455 128
1934 -0.200  11       1984  0.133 128
1935 -0.216  11       1985 -1.549 128
1936 -0.318  11       1986 -2.218 128
1937 -1.757  11       1987 -0.858 128
1938 -0.567  12       1988  1.178 128
1939 -0.986  12       1989  1.104 128
1940 -1.483  14       1990  1.231 128
1941 -1.123  18       1991 -0.014 129
1942  1.374  19       1992  1.124 129
1943  1.271  20       1993  0.568 129
1944 -2.164  20       1994  1.531 129
1945 -0.303  21       1995 -0.286 129
1946  0.662  23       1996 -2.485 129
1947  0.857  23       1997 -0.023 129
1948 -1.533  24       1998  0.129 129
1949  0.122  24       1999  1.236 129
-------------------------------------------------------------------------------------------------------------------------------------
PART 4:  Master Bar Plot:                                                                           00:00   00     0000  Page   6
-------------------------------------------------------------------------------------------------------------------------------------
Year Rel value  Year Rel value  Year Rel value  Year Rel value  Year Rel value  Year Rel value  Year Rel value  Year Rel value
1950-------B    2000----a
1951----a       2001--b
1952------B     2002-----@
1953--------C   2003--------D
1954------A     2004---a
1955--------D   2005----@
1956--b         2006-----A
1957---b        2007j
1958-------C    2008---a
1959-----@      2009---------E
1960--d         2010-------B
1961--------C   2011---a
1962-----A      2012-----A
1963---b        2013--c
1914----------F 1964-------B    2014--------C
1915-e          1965----a       2015------A
1916h           1966------A     2016f
1917--d         1967-----@      2017-f
1918----------E 1968--------C   2018--c
1919----------I 1969------A     2019---------E
1920----------F 1970-----@      2020--------D
1921--------C   1971-------B    2021-------B
1922---a        1972--c         2022-----@
1923---------D  1973-------B
1924---------E  1974-----@
1925--------D   1975--------C
1926-----A      1976-e
```

```
1927-e          1977--c
1928-f          1978--c
1929-d          1979--c
1930------A     1980g
1931----------H 1981---b
1932---------D  1982---------E
1933--------C   1983-------B
1934----a       1984-----A
1935----a       1985f
1936---a        1986i
1937g           1987--c
1938--b         1988---------E
1939-d          1989---------D
1940f           1990---------E
1941-d          1991-----@
1942----------E 1992---------D
1943---------E  1993-------B
1944i           1994----------F
1945---a        1995---a
1946-------C    1996j
1947--------C   1997-----@
1948f           1998-----A
1949-----@      1999---------E
PART 5:  CORRELATION OF SERIES BY SEGMENTS:                                                        00:00   00     0000  Page   5
-----------------------------------------------------------------------------------------------------------------------------------
Correlations of  50-year dated segments, lagged  25 years
Flags:  A = correlation under  0.3281 but highest as dated;  B = correlation higher at other than dated position
Seq Series  Time span   1900 1925 1950 1975
1949 1974 1999 2024
--- -------- ---------  ---- ---- ---- ---- ---- ---- ---- ---- ---- ---- ---- ---- ---- ---- ---- ---- ---- ---- ---- ----
1 NG1070   1941 2021        .57  .61  .78
2 NG1071   1941 2021        .68  .56  .51
3 NG1110   1956 2021             .34  .22B
4 NG1111   1965 2021            -.03B .00B
5 NG1120   1952 2021             .29A .29A
6 NG1121   1943 2021        .45  .28B .31A
7 NG1180   1961 2021             .36  .31A
8 NG1181   1963 2021             .30A .27B
9 NG1182   1963 2021             .46  .48
10 NG1183   1968 2021             .46  .47
11 NG1190   1964 2021             .57  .61
12 NG1191   1957 2021             .42  .37
13 NG1280   1971 2021             .38  .38
14 NG1281   1972 2021             .34
15 NG1282   1962 2022             .48  .47
16 NG1283   1976 2022                  .52
17 NG1290   1961 2022             .67  .76
18 NG1291   1965 2022             .50  .54
19 NG1320   1964 2021             .51  .63
20 NG1321   1966 2021             .42  .32A
21 NG1400   1968 2022             .24B .22B
22 NG1401   1967 2022             .37  .41
23 NG1420   1968 2022             .47  .45
24 NG1421   1969 2022             .59  .61
25 NG1422   1969 2022             .53  .55
26 NG1423   1971 2022             .36  .35
27 NG1430   1968 2022             .58  .58
28 NG1431   1958 2022             .52  .63
29 NG1440   1959 2022             .36  .32A
30 NG1441   1968 2022             .31A .32A
31 NG1470   1975 2022                  .35
32 NG1471   1966 2022             .37  .43
33 NG151    1968 2017             .39
34 NG1580   1962 2021             .36  .45
35 NG1581   1967 2021             .31A .37
36 NG2640   1958 2021             .56  .60
37 NG2641   1959 2021             .51  .55
38 NG2650   1952 2021             .49  .31B
39 NG2651   1962 2021             .41  .34
40 NG2710   1968 2021             .56  .50
41 NG2711   1967 2021             .52  .53
42 NG2712   1970 2021             .53  .52
43 NG2713   1965 2021             .58  .62
44 NG2800   1967 2021             .38  .36
45 NG2801   1958 2021             .46  .32B
46 NG2900   1948 2021        .28A .41  .52
47 NG2901   1960 2021             .56  .58
48 NG2940   1957 2021             .29A .43
49 NG2941   1957 2020             .45  .38
50 NG3020   1955 2021             .58  .55
51 NG3021   1951 2021             .44  .51
52 NG3040   1960 2021             .68  .45
53 NG3041   1972 2021             .29B
54 NG3051   1960 2021             .61  .63
55 NG3052   1953 2021             .48  .63
56 NG3101   1951 2021             .48  .38
57 NG3102   1956 2021             .58  .64
58 NG3571   1983 2021                  .53
59 NG3572   1991 2021                  .56
60 NG3581   1970 2021             .49  .49
61 NG3582   1975 2021                  .33A
62 NG4141   1946 2020        .60  .54  .50
```

63	NG4142	1950	2021			.44	.37
64	WG11	1964	2022			.56	.58
65	WG12	1968	2022			.51	.60
66	WG101	1964	2022			.42	.49
67	WG102	1961	2022			.40	.46
68	WG111	1928	2022		.28A	.36	.47
69	WG112	1933	2022		.26A	.38	.42
70	WG121	1962	2022			.51	.61
71	WG122	1973	2022			.64	
72	WG131	1966	2022			.70	.70
73	WG132	1959	2022			.65	.72
74	WG141	1971	2022			.41	.35
75	WG142	1963	2022			.48	.44
76	WG151	1959	2022			.63	.61
77	WG152	1961	2022			.52	.55
78	WG161	1971	2022			.41	.40
79	WG162	1963	2022			.65	.64
80	WG171	1957	2022			.38	.60
81	WG172	1963	2022			.64	.68
82	WG181	1927	2022		.54	.43	.47
83	WG182	1921	2022	.31A	.33	.28B	.41
84	WG191	1964	2022			.47	.51
85	WG192	1967	2022			.46	.48
86	WG21	1959	2022			.33B	.30A
87	WG22	1959	2022			.46	.45
88	WG201	1972	2022			.45	.46
89	WG202	1960	2022			.39	.46
90	WG211	1957	2022			.27A	.27B
91	WG212	1957	2022			.58	.50
92	WG221	1927	2022		.34	.33	.38B
93	WG222	1946	2022		.41	.49	.41
94	WG231	1961	2022			.45	.58
95	WG232	1962	2022			.60	.54
96	WG241	1916	2022	.36	.30A	.12B	.26A
97	WG242	1914	2022	.47	.43	.44	.41B
98	WG251	1954	2022			.48	.64
99	WG252	1938	2022		.32A	.35	.58
100	WG261	1962	2022			.21B	.34B
101	WG262	1964	2022			.46	.41
102	WG271	1953	2022			.29B	.34
103	WG272	1954	2022			.37	.63
104	WG281	1960	2022			.68	.61
105	WG282	1964	2022			.38	.34
106	WG291	1920	2022	.27A	.30A	.35	.42
107	WG292	1933	2022		.35	.28A	.38
108	WG31	1967	2022			.54	.54
109	WG32	1961	2022			.39	.47
110	WG301	1969	2022			.36	.35
111	WG302	1960	2022			.21A	.45
112	WG311	1964	2022			.55	.48
113	WG312	1963	2022			.57	.38
114	WG321	1967	2022			.26B	.25B
115	WG322	1959	2022			.04B	.18B
116	WG331	1942	2022		-.18B	.22A	.24A
117	WG332	1945	2022		.20B	.21B	.19B
118	WG41	1963	2022			.61	.59
119	WG42	1961	2022			.60	.57
120	WG51	1958	2022			.34	.44
121	WG52	1965	2022			.54	.37
122	WG61	1925	2022		.60	.44	.53
123	WG62	1933	2022		.47	.34	.25A
124	WG71	1940	2022		.37B	.30A	.35
125	WG72	1941	2022		.41	.23B	.21B
126	WG81	1963	2022			.52	.45
127	WG82	1958	2022			.60	.58
128	WG91	1940	2022		.17B	.17A	.24B
129	WG92	1941	2022		.22A	.34	.31B
Av segment correlation				0.35	0.36	0.44	0.45
